# Strategies for involving patients and the public in scaling initiatives in health and social services: A scoping review

**DOI:** 10.1111/hex.14086

**Published:** 2024-06-05

**Authors:** Roberta de Carvalho Corôa, Ali Ben Charif, Vincent Robitaille, Diogo G. V. Mochcovitch, Mamane Abdoulaye Samri, Talagbe Gabin Akpo, Amédé Gogovor, Virginie Blanchette, Lucas Gomes Souza, Kathy Kastner, Amélie M. Achim, Robert K. D. McLean, Andrew Milat, France Légaré, The RePOS Network

**Affiliations:** ^1^ VITAM—Centre de recherche en santé durable Centre intégré universitaire de santé et services sociaux de la Capitale‐Nationale Quebec City Quebec Canada; ^2^ Unité de soutien au système de santé apprenant Québec Quebec City Quebec Canada; ^3^ Faculty of Medicine Université Laval Quebec City Quebec Canada; ^4^ CubecXpert Quebec City Quebec Canada; ^5^ Faculty of Nursing Université Laval Quebec City Quebec Canada; ^6^ Faculty of Business Administration Université Laval Quebec City Quebec Canada; ^7^ Institut National de la Recherche Scientifique Université Laval Quebec City Quebec Canada; ^8^ Department of Human Kinetics and Podiatric Medicine Université du Québec à Trois‐Rivières Trois‐Rivières Quebec Canada; ^9^ Best Endings Winnipeg Manitoba Canada; ^10^ CERVO Brain Research Centre Quebec City Quebec Canada; ^11^ International Development Research Centre Ottawa Ontario Canada; ^12^ Faculty of Medicine and Health Sciences Stellenbosch University Stellenbosch Western Cape South Africa; ^13^ School of Public Health University of Sydney Sydney New South Wales Australia; ^14^ Sydney Health Partners Sydney New South Wales Australia; ^15^ Centre de Recherche du CHU de Québec ‐ Université Laval Quebec City Quebec Canada

**Keywords:** co‐construction, health and social services, implementation science, participatory research, patient and public involvement and engagement (PPIE), scalability, scaling

## Abstract

**Background:**

Scaling in health and social services (HSS) aims to increase the intended impact of proven effective interventions. Patient and public involvement (PPI) is critical for ensuring that scaling beneficiaries’ interests are served. We aimed to identify PPI strategies and their characteristics in the science and practice of scaling in HSS.

**Methods:**

In this scoping review, we included any scaling initiative in HSS that used PPI strategies and reported PPI methods and outcomes. We searched electronic databases (e.g., Medline) from inception to 5 February 2024, and grey literature (e.g., Google). Paired reviewers independently selected and extracted eligible reports. A narrative synthesis was performed and we used the PRISMA for Scoping Reviews and the Guidance for Reporting Involvement of Patients and the Public (GRIPP2).

**Findings:**

We included 110 unique reports out of 24,579 records. In the past 5 years, the evidence on PPI in scaling has increased faster than in any previous period. We found 236 mutually nonexclusive PPI strategies among 120 scaling initiatives. Twenty‐four initiatives did not target a specific country; but most of those that did so (*n* = 96) occurred in higher‐income countries (*n* = 51). Community‐based primary health care was the most frequent level of care (*n* = 103). Mostly, patients and the public were involved throughout all scaling phases (*n* = 46) and throughout the continuum of collaboration (*n* = 45); the most frequently reported ethical lens regarding the rationale for PPI was consequentialist‐utilitarian (*n* = 96). Few papers reported PPI recruitment processes (*n* = 31) or incentives used (*n* = 18). PPI strategies occurred mostly in direct care (*n* = 88). Patient and public education was the PPI strategy most reported (*n* = 31), followed by population consultations (*n* = 30).

**Conclusions:**

PPI in scaling is increasing in HSS. Further investigation is needed to better document the PPI experience in scaling and ensure that it occurs in a meaningful and equitable way.

**Patient and Public Contribution:**

Two patients were involved in this review. They shared decisions on review questions, data collection instruments, protocol design, and findings dissemination.

**Review Registration:**

Open Science Framework on 19 August 2020 (https://osf.io/zqpx7/).

## 1 BACKGROUND

Health and social care systems worldwide are facing significant resource shortages and an increase in noncommunicable diseases.^1^ The COVID‐19 pandemic highlighted the importance of *ubuntu*,^2^ an African humanist philosophy based on the notion that for their own survival, people connect with one another and with systems through relations of mutual respect, dignity, solidarity and compassion.^3^ *Ubuntu* supports the idea that interdependence and co‐construction are essential for sustaining health and social care system recovery efforts.^2^, ^4^ Patient and public involvement (PPI) in health and social services (HSS) could foster this kind of dignity and respect by integrating the experiential knowledge of patients and the public in efforts to improve health and social care systems.^5^, ^6^ Indeed, the United Nations has emphasized the need for more collaborative and interdisciplinary initiatives to achieve all its sustainable development goals.^7^

Recently, the World Health Organization (WHO) declared ‘safe and scalable’ care as one of the pillars of a new strategy aimed at supporting countries as they transitioned from an emergency COVID‐19 response to longer‐term sustained prevention, control, and management.^8^ The concept of ‘scaling’ emerged in HSS in the 2000s in the fields of implementation of science and knowledge mobilization as the process of transferring an intervention from a controlled setting into practical implementation, which usually requires a change of scale, or ‘scaling’.^9^, ^10^ A series of WHO publications^11^ established milestones in the scaling field by defining more specific terms and goals. They describe it as an empirical phenomenon distinct from the transfer of research findings and other evidence‐based practices into routines in HSS as described by implementation science.^12^ Scaling goes beyond implementation by (1) expanding tested interventions,^13^ and (2) increasing their impact.^14^ Scaling as a practice can also be found in the literature as a synonym of ‘spread’, meaning replicating an intervention elsewhere.^10^, ^15^ Scaling has become increasingly urgent in the 21st century as pilot projects proliferate in higher‐income countries (HICs)^16^ while their health and social care systems break down, and low‐ and middle‐income countries (LMICs) struggle to expand the coverage of their HSS.^17^

Based on the specialized literature on the topic, we define scaling in HSS as a systematic evidence‐informed and ethically congruent process whose aim is to increase the intended impacts of an intervention that has proven effective for improving quality in care and the equitable welfare of individuals and populations.^13^, ^14^, ^15^, ^18^ Scaling in HSS may also aim to increase equity impacts, that is, ensure that not just a larger but a more diverse population has access to the benefits of an intervention.^14^

The WHO recommends involving a broad range of stakeholders in scaling, including scaling beneficiaries.^13^ This increases commitment to a project, ensures that the scaling impacts are relevant, patient‐centred and inclusive, and that the interests of people who experience its impacts are respected.^13^, ^19^, ^20^ A previous knowledge synthesis showed that an absence of PPI, that is, failure to recognize the agency of patients and the public as main actors in health and social care systems or to integrate their knowledge, experiences and values, is one of the barriers to successful scaling.^21^ However, scaling frameworks in HSS, while emphasizing that stakeholder involvement is a critical success factor,^13^, ^18^, ^20^ often lack information on strategies and methods for working with users and beneficiaries. We define PPI in the science and practice of scaling in HSS as a partnership that scaling practitioners (e.g., researchers, policymakers, health professionals) establish with patients, citizens, communities and other civil entities to scale effective health and social interventions (whether in research, direct care, training, or policy making) and increase their impact.^5^, ^6^, ^13^, ^14^, ^15^, ^22^, ^23^, ^24^, ^25^, ^26^, ^27^, ^28^

Although scaling guidelines recognize the potential of PPI to enhance scaling impact,^13^, ^20^, ^29^ to the best of our knowledge no reviews have examined PPI strategies in scaling initiatives in HSS as a whole.^15^ In this review, our research question^30^ was therefore ‘what strategies have been used for involving patients and the public in scaling initiatives in HSS?’ Our specific objectives were as follows: (1) review existing literature on PPI in any scaling initiative in HSS; (2) identify strategies for involving patients and the public in the science and practice of scaling in HSS, and 3) describe the characteristics of these strategies.

## 2 METHODS

We conducted a scoping review to identify PPI scaling strategies and their characteristics in HSS. Scoping reviews allow the mapping of concepts and practices drawn from a wide range of sources,^31^, ^32^ and are useful for exploring theoretical and empirical evidence on emerging complex social phenomena that have not yet been systematically explored in the literature.^33^ We followed the Joanna Briggs Institute (JBI) Methods Manual for scoping reviews^31^ and used the Preferred Reporting Items for Systematic reviews and the Meta‐Analyses extension for Scoping Reviews (PRISMA‐ScR)^34^ checklist (Additional File 1). We considered the Sex and Gender Equity in Research (SAGER) guidelines^35^ when extracting, analyzing and reporting data (Additional File 2). The protocol was registered with the Open Science Framework on August 19, 2020 (registration identifier: https://osf.io/zqpx7/) and published in 2021.^36^

### 2.1 Patient or public contribution

Through our research and practice networks we recruited two patient instructors, a woman and a man, as members of the research team. We used the Guidance for Reporting Involvement of Patients and the Public (GRIPP2)^37^ to report on PPI characteristics and outcomes (Additional File 3). Patient partners shared decisions with researchers about the review questions and data collection instruments, revising the protocol and findings and disseminating study results. Partners participated through a steering committee, virtual meetings, emails, and newsletters. One partner presented the preliminary review results alongside the first author at an international conference. We reimbursed them for the hours they worked, and acknowledged their contributions in results dissemination including as publication authors.

### 2.2 Diversity, equity and inclusion statement

The present authors are a diverse group of females and males from various backgrounds, including patients, clinicians, researchers, students and policymakers. They span multiple disciplines, including sociology, epidemiology, medicine, philosophy, psychology and administration. They hail from countries with differing income levels, including Brazil, Comoros, Canada, Togo, Benin, Cameroon, and Australia.

### 2.3 Search strategy

We searched published studies in the following electronic bibliographic databases: MEDLINE via Ovid, Embase via Elsevier, Web of Science, CINAHL via EBSCOhost, PsycINFO via Ovid, ERIC via EBSCO, the Cochrane Library, Sociological Abstract via ProQuest, and Academic Search Premier via EBSCO. There were no restrictions regarding language, publication date, or record type. We searched databases from inception to 5 February 2024.

The Ovid‐MEDLINE search strategy was developed and performed by an information specialist with input from the research team and reviewed by a second information specialist using the Peer Review of Electronic Search Strategies (PRESS) guideline.^38^ Search terms reflected the following main concepts: ‘patient and public involvement’, ‘scaling’, and ‘health and social services’. These terms were adapted to the above‐mentioned databases. The search strategy was then translated into other electronic bibliographic databases.

We followed a systematic approach recommended by the Canadian Agency for Drugs and Technologies in Health^39^ to search the grey literature. We searched Google and the websites of 12 major national and international healthcare improvement agencies. We retrieved the first 100 reports for each term, but because it was feasible, we retrieved all reports available in the ExpandNet website. The grey literature search was performed on various dates in February 2022 (Additional file 4).

### 2.4 Eligibility criteria

**Figure 1:**
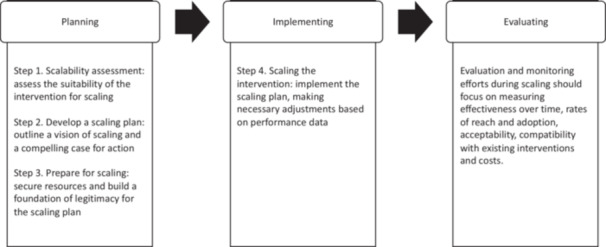
Scaling phases. Adpated from: Milat et al.^18^

**Figure 2:**
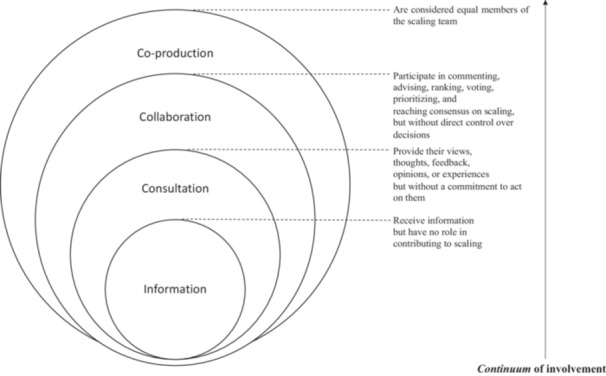
Continuum of involvement. Adapted from: Carman et al.^22^ and Pomey et al.^5^

We translated our review question into PICOS components, that is, population (patients and the public), intervention (PPI strategies), comparator (with/without), outcome (impact of PPI), and setting (HSS) to clarify our eligibility criteria.^40^ Like many other scoping reviews on PPI,^41^, ^42^, ^43^, ^44^ we used the PICOS framework instead of PCC (Population, Concept, and Context) as recommended by the JBI^31^ because we were seeking interventions reporting outcomes.^45^ Considering outcomes was a recommendation from our patient instructor, who noted that PPI can have measurable effects on people's health, well‐being, and experiences with health systems.^46^ Our inclusion criteria were:

1. *Population (P)*. We considered records that included any type of stakeholder involved in PPI strategies in scaling in HSS. They could be involved in any phase of scaling, that is, planning, implementing or evaluation^18^ (Figure 1). We defined stakeholders as any person or group with an interest in or a role in a scaling initiative or affected by its impact.^45^, ^47^ Records had to include patients as stakeholders (or their families and representatives) or the public overall (e.g., beneficiaries, civil society organizations, users); and could include providers (e.g., nurses, physicians, community‐based workers); funders (e.g., employers, governments); policy makers (e.g., governments and professional associations); product makers (e.g., drug and device manufacturers); researchers (e.g., academic institutions); and the press (e.g., publishers and news media). We excluded reports that did not include patients or the public as stakeholders.
2. *Intervention (I)*. We included records reporting any strategy proposed for involving patients or the public, alone or with other stakeholders, in scaling initiatives in HSS. We considered ‘scaling’ as inclusive of all variants of spread, scale or scaling up, scaling out, scaling in, or scaling deep, horizontal, or vertical.^15^ Scaling initiatives could be in scaling science, that is, in research generating new scaling evidence through reflecting on a scaling process (e.g., observational studies, clinical trials), or in scaling practice, that is, intention to scale or act of scaling an intervention, with or without research (e.g., public policies, frameworks). We considered PPI to include terms such as community participation, cooperation, engagement or participatory research. We sought PPI strategies at all points in the involvement *continuum*, from receiving information to co‐construction of scaling initiatives.^5^, ^22^ (Figure 2). Strategies require methods, that is, ways of enacting the strategy. For example, the method for the strategy of involving patients in a policy advisory group might be to form a joint steering committee. We excluded records that did not report any scaling objectives, methods and/or outcomes; that reported PPI strategies but did not report any method employed; or that reported patients or the public as the target population of a scaling intervention but did not involve them as described above.
3. *Comparison(s) (C)*: We included both studies with comparison groups and studies without.
4. *Outcomes (O)*: We included records that reported at least one of the following PPI outcomes^45^ in scaling initiatives: (1) internal outcomes, that is, impact on the involved patients or public participants themselves (e.g., skills, trust), impact on services provided (e.g., efficiency, cost‐effectiveness), and impact on the organization or system (e.g., knowledge of health issues, public support); (2) external outcomes, that is, influence on the broader public (e.g., health system accountability, policy changes, decision‐making methods) and influence on population health generally (equity, health status); and (3) aggregate outcomes, for example, the overall cost‐effectiveness of PPI.
5. *Types of Source*. We included case studies, qualitative studies, expert panels (e.g., Delphi studies), experimental studies (trials), mixed methods, and observational studies (cohorts, case‐controls, and cross‐sectionals). We also included guides, frameworks, reports and tools if they reported the aim and the method. We excluded withdrawn publications, study protocols, editorial material (e.g., blogs, interviews, letters, newsletters), opinion texts, nonauthored records (anonymous), nontraceable appendices or parts of documents, and video or audio records and presentations. We also excluded reviews due to their lack of primary data. No restrictions were placed on the language or date of publication. We planned to use DeepL translator for sources written in languages other than English, French, Spanish or Portuguese.^48^
6. *Setting*. We included records that referred to any type of HSS, with no geographical restrictions. We considered ‘health and social services’ as services provided in institutional or community settings aiming to promote and protect physical, mental, and social well‐being among individuals.^49^, ^50^

See Additional file 5 for further details of eligibility criteria.

### 2.5 Selection process

Records retrieved were stored in an EndNote X9 library for reference management and duplicate removal^51^ and then exported to the Internet‐based system Covidence^52^ for the selection process. We operationalized our inclusion criteria using questions based on our PICOS elements. We conducted a pilot screening of the titles and abstracts with paired independent reviewers using a random sample of 5% of the records to ensure a common understanding of the selection criteria. Agreement among the evaluators during the pilot screening was assessed using Gwet's Kappa matrix. This showed an average of 0.92 (0.03) for all evaluators. Proceeding with the selection process, if information presented in the titles and abstracts was inconclusive, we retained the record for full‐text selection by paired independent reviewers. Throughout the selection process, any doubts or disagreements were resolved through consensus between the paired reviewers and, if necessary, a third party. All assessed records, along with reasons for exclusion and decisions regarding inclusion, were recorded in Excel.

### 2.6 Data extraction

**Figure 3:**
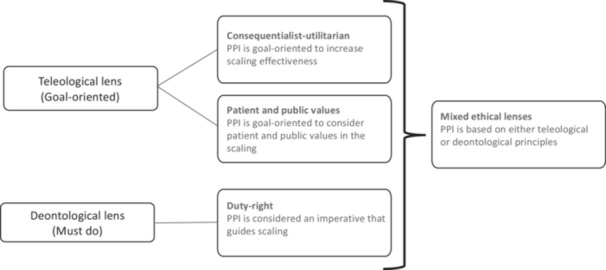
Ethical lenses regarding rationale for PPI. Adapted from: Bégin.^55^

We conducted a pilot using Excel with paired independent reviewers on three included records to discuss data items and achieve a common understanding of the data‐charting form. After this calibration exercise, data extraction by paired independent reviewers proceeded using Covidence, and any disagreements were resolved as above. Our extraction followed the following data themes:

1. Bibliographic characteristics: journal; authors; corresponding author; country of corresponding author; year of publication; language; aim of the study; study design or other types of source (e.g., tool, guide); funding.
2. Characteristics of scaling initiatives: title; description; country; income levels of targeted country (HICs, or high‐income and upper‐middle‐income countries, LMICs, or lower‐middle income and low‐income countries, and not specified); developers (e.g., patients or the public, providers); targeted level of care (e.g., community‐based primary health care, secondary care); health and social issues addressed (e.g., HIV, violence against children); targeted setting (e.g., community, health care system); sex‐ or gender‐sensitivity (e.g., initiative focused on particular groups that are vulnerable due to their gender and/or sex‐based roles in society).
3. PPI strategies: We used a PPI model adapted from previous conceptual frameworks on PPI in social and health domains,^5^, ^22^, ^46^, ^53^ all based on Arnstein's widely quoted ‘ladder of citizen participation’.^53^, ^54^ This shows a vertical axis representing the *continuum* of PPI (from information to co‐construction) and a horizontal axis representing system levels where the initiative occurs (from direct care to policy making). Within this model, we considered 27 potential PPI strategies (e.g., patient education, population consultation) identified in a systematic review on mental health care,^26^ and identified the methods used to enact the strategy (e.g., organizing workshops, field visits and interviews) as an open‐text variable. We developed our own PPI model because none of our reference frameworks addressed scaling specifically. See Additional File 7 for detailed definitions of model components.
4. Characteristics of PPI strategies: scaling phase^18^ in which the involvement occurred (e.g., planning, implementing, evaluating); ethical lenses^55^ in the rationale^56^ for PPI, that is, the ethical reasoning reported for PPI in scaling, whether stated or implied, as interpreted by our reviewers and validated by the team ethicist (Figure 3); recruitment process; patient and public recruitment profile; incentivization; use of an involvement framework or guide (yes/no); other stakeholders involved; PPI outcomes^45^; sex‐ or gender‐sensitivity (yes/no).
5. PROGRESS framework^57^ factors: patient and public participants’ place of residence, race/ethnicity/culture/language, occupation, gender/sex, religion, education, socioeconomic status, and social capital (Additional File 6).

### 2.7 Quality assessment of PPI reporting

We collected data on the quality of PPI reporting in the reports included in our review using an adapted version of the GRIPP2 checklist^37^ to enhance the quality, transparency, and consistency of the PPI evidence base. The checklist comes in two formats: a long‐form list with 15 items and a short‐form list with 5 items. We used an intermediate version with 8 items, as we found the short‐form version sufficiently comprehensive for our purposes, but we included three additional items from the long‐form version. It was important to include items on PPI definitions and theoretical influences because a previous review^15^ identified the lack of definitions of scaling concepts, such as PPI in scaling, as a barrier to advancing the evidence base in this domain. Additionally, we included input on the description of patients and the public involved because this is necessary for transparency.

### 2.8 Data analysis

The selection process was summarized using the 2020 PRISMA Flow Diagram.^58^ All results were exported to Excel and the data was synthesized using frequencies. For open‐text data, we grouped information into categories (e.g., both breastfeeding and newborn mortality papers were categorized under ‘Maternal and child health’). We used ‘Not reported’ for missing information. We used ‘Not specified’ for generic information (e.g., reports that did not focus on a specific health issue). The data was mainly analysed in a mutually exclusive format; except for (a) ethical lenses regarding the rationale for PPI, (b) patient and public recruited profile, (c) other stakeholders involved, (d) PPI strategies and methods, and (e) PPI outcomes, for which we proceeded with nonmutually exclusive (multilabel) classes. The input for the quality assessment of PPI reporting using GRIPP2 was binary (Yes/No), and no score was calculated. We did not exclude reports based on quality assessment results. We present and discuss our results in the form of a narrative synthesis.

We deviated from the protocol^36^ in the following ways: we did not conduct searches in citations and references of included reports. We conducted a pilot on 10% of the included reports, and we found that this additional search did not yield significant new data. Second, we did not contact authors to request missing information or additional data because the research team considered that we already had a relevant body of evidence from data extraction to address the questions and objectives of the review. Third, we did not include systematic reviews because they rarely provide primary study data on PPI methods.

## 3 RESULTS

### 3.1 Bibliographic characteristics

**Figure 4:**
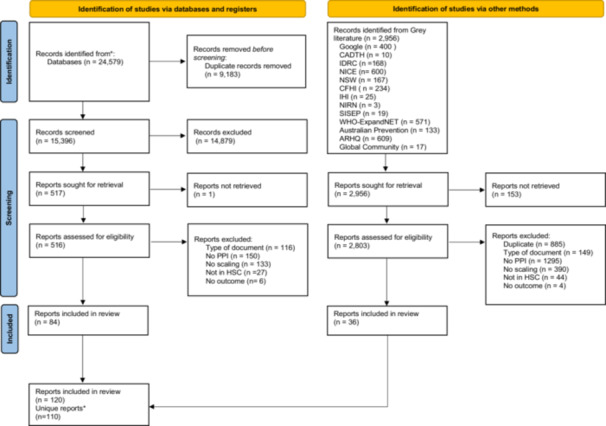
PRISMA flow. *HSC is for ‘health and social care’. *10 Associated reports. From: Page et al.^58^ For more information, visit: http://www.prisma-statement.org/.

We identified 24,579 records, of which 84 met the eligibility criteria (Figure 4). The grey literature search identified 2956 records, of which 36 were included in the review. We did not assess 163 grey literature reports that were unavailable through the access links provided. We included 110 unique reports and 10 associated reports. Three reports from the grey literature were associated with the peer‐reviewed documents identified through the database searches. Reports were mostly excluded because they did not mention any PPI strategy in the scaling (*n* = 1445). The list of included reports is presented in Additional File 8, while a list of excluded reports with reasons for their exclusion is presented in Additional File 9.

**Figure 5:**
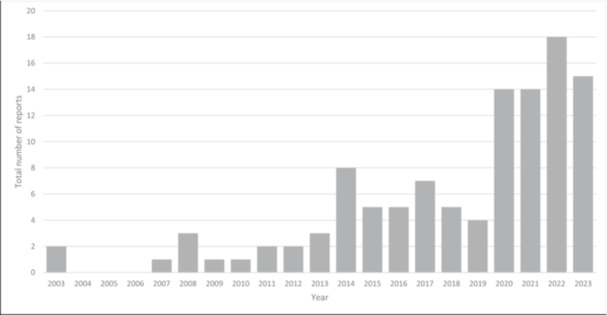
Reports distribution per year.

All 110 unique included reports were published in English between 2003 and 2023. In the last 5 years (2019–2023) there was a 144.4% (*n* = 65) increase in the number of publications compared with the total increase in all previous years (*n* = 45) (Figure 5). The corresponding authors were primarily from HICs (*n* = 80), with the United States (*n* = 40), the United Kingdom (*n* = 12), and Canada (*n* = 9) at the top of the list. Eighty reports were classified as studies, with case studies the most frequent (*n* = 27), and 30 reports were identified as other types of source, mostly guides or frameworks (*n* = 22) (Table 1).

**Table 1:** Bibliographic characteristics.

	*n* = 120	*%*
*Country of corresponding author*		
Higher‐income countries (HICs)	80	66.7
United States	40	33.3
United Kingdom	12	10.0
Canada	9	7.5
Australia	5	4.2
Switzerland	3	2.5
Ireland	2	1.7
Argentina	1	0.8
Botsawana	1	0.8
Brazil; Chile	1	0.8
Denmark	1	0.8
Estonia	1	0.8
Guatemala	1	0.8
Portugal	1	0.8
South Africa	1	0.8
Uruguay	1	0.8
Low‐ and middle‐income countries (LMICs)	24	20.0
India	5	4.2
Nigeria	4	3.3
Ethiopia	2	1.7
Ghana	2	1.7
Bangladesh	1	0.8
Democratic Republic of the Congo	1	0.8
Kenya	1	0.8
Malawi	1	0.8
Samoa	1	0.8
Senegal	1	0.8
Tanzania	1	0.8
Uganda	1	0.8
Vietnam	1	0.8
Zambia and Kenya	1	0.8
Zimbabwe	1	0.8
Both HICs and LMICs	3	2.5
Bangladesh; Burkina Faso; Ethiopia; India; Nigeria; Vietnam; United States	1	0.8
Egypt; Germany; Sweden	1	0.8
Kenya, Rwanda and Canada	1	0.8
Not reported	3	2.5
*Types of sources*		
Study design	80	66.7
Case study	27	22.5
Qualitative study	24	20.0
Observational study (cohort, case‐control, and cross‐sectional	12	10.0
Mixed methods	10	8.3
Experimental study (trial)	7	5.8
Others	30	25.0
Guide or framework	22	18.3
Report	7	5.8
Tool	1	0.8

### 3.2 Critical appraisal

Fourteen reports provided the PPI definition used in scaling and 64 presented a theoretical rationale or influences. Document aim was defined in 109 reports. As expected, due to our eligibility criteria, all 110 reports provided a description of the patients and the public involved, detailed involvement methods, and presented PPI outcomes. A total of 106 reports commented on how PPI influenced scaling overall, and 99 included a critical discussion of the scaling. The completed GRIPP2 form is in Additional File 10 and the data is summarized in Table 2.

**Table 2:** Guidance for Reporting Involvement of Patients and the Public (GRIPP2).

	*n* = 110	*%*
*GRIPP2 Items*		
Definition of PPI	14	12.7
PPI theoretical rationale	64	58.2
Aim of the report	109	99.1
Clear description of PPI methods	110	100.0
Description of patients and the public involved	110	100.0
Reported the results of PPI in the scaling	110	100.0
Commented on how PPI influenced the scaling	106	96.4
Commented critically on the scaling	99	90.0

Abbreviation: PPI, patient and public involvement.

### 3.3 Characteristics of scaling initiatives

The 110 unique reports covered 120 scaling initiatives. Four reports addressed multiple scaling initiatives. Twenty‐four scaling initiatives did not aim to target a specific country; however, among those that did so (*n* = 96), HICs (*n* = 51) were more frequently targeted. Thirty‐two scaling initiatives were developed by patients and the public, 27 of which were with other stakeholders. Community‐based primary health care was the most frequently targeted level of care (*n* = 103). Scaling initiatives mostly addressed targeted populations (e.g., women's health, older adults’ health) (n = 26), infectious diseases (*n* = 22), and health interventions (*n* = 20), most of which focused on HIV/AIDS (*n* = 15), maternal and child health (*n* = 13), and e‐Health (*n* = 12). The community setting was the most frequently reported target setting for the scaling initiative (*n* = 90), and 34 of the scaling initiatives were reported as sex‐ or gender‐sensitive (Table 3).

**Table 3:** Characteristics of scaling initiatives.

	*n* = 120	*%*
*Income level of targeted countries*		
Higher income	51	42.5
Low and middle income	43	35.8
Not specified	24	20.0
Both higher income and low and middle income	2	1.7
*Developers*		
Other stakeholders	88	73.3
Patients and the public with other stakeholders	27	22.5
Patients and the public	5	4.2
*Levels of care*		
Community‐based primary health care (CBPHC)	103	85.8
Tertiary care	4	3.3
Secondary care	2	1.7
Secondary care and tertiary care	1	0.8
Not specified	10	8.3
*Health and social issues*		
Populations	26	21.7
Maternal and child health	13	10.8
Child and adolescent health	6	5.0
Women's health	4	3.3
Older adults’ health	2	1.7
Men's health	1	0.8
Infectious diseases	22	18.3
HIV/AIDS	15	12.5
COVID‐19	3	2.5
Malaria	2	1.7
Dengue, Chikungunya and Zika	1	0.8
Tuberculosis	1	0.8
Health interventions	20	16.7
e‐Health	12	10.0
Health innovation regardless of disease	5	4.2
Palliative care	1	0.8
Decision Aids	1	0.8
Complementary and alternative medicine (CAM)	1	0.8
Health promotion	13	10.8
Physical activity	4	3.3
Healthy nutrition	2	1.7
Sustainable health	1	0.8
Occupational health	1	0.8
Sexual and reproductive health	5	4.2
Health and social services improvement	12	10.0
Health care improvement and/or quality and/or access	7	5.8
Patient safety and quality	3	2.5
Community engagement, accountability and responsiveness	2	1.7
Mental health	10	8.3
Chronic diseases	5	4.2
Cardiovascular diseases	3	2.5
Diabetes	1	0.8
Chronic Obstructive Pulmonary Disease (COPD)	1	0.8
Not specified	12	10.0
*Setting of the initiative*		
Community	90	75.0
Health care system	24	20.0
Workplace	1	0.8
Not specified	5	4.2
*Sex‐ or gender‐sensitive scaling*		
Not reported	86	71.7
Yes	34	28.3

### 3.4 PPI strategies

**Figure 6:**
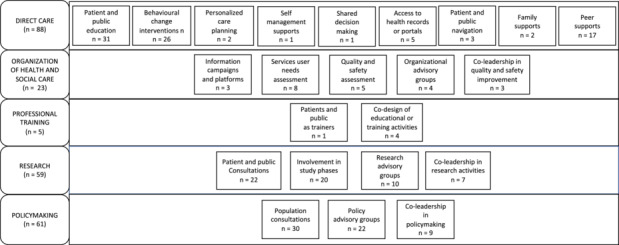
Patient and public involvement strategies. Adapted from: Menear et al.^26^

Twenty‐three PPI strategies were found to be employed 236 times in the 120 scaling initiatives (Figure 6).

#### 3.4.1 Health and social care system level

The strategies were mostly found at the direct care level, that is, through direct interactions with health professionals in the delivery of care (*n* = 88). A significant number of PPI strategies were also found at the policy‐making level (*n* = 61). Patients and the public were least involved at the level of professional training, that is, participating in the training of health professionals in scaling (*n* = 5), and at the level of organization of health and social care, that is, in the planning, governance or assessment of health and social care (*n* = 23).

#### 3.4.2 Type of PPI strategy

Overall, patient and public education was the PPI strategy most reported (e.g., educational resources helping to increase patient and public knowledge about a specific topic related to the scaling) (*n* = 31), followed by population consultations (e.g., surveys or interviews to inform a health policy) (*n* = 30), behavioural change interventions (e.g., actions aimed at increasing patient and public motivation on a topic related to the scaling) (*n* = 26), and policy advisory groups (e.g., committees in which patients and the public provide guidance on system‐level scaling policies) (*n* = 21). A comprehensive list of identified PPI strategies and their respective methods is provided in Additional file 11.

### 3.5 Characteristics of PPI strategies

#### 3.5.1 Continuum of involvement

Patients and the public were involved, in order of frequency, at the levels of collaboration (*n* = 45), consultation (*n* = 38), co‐production (*n* = 24) and information (*n* = 13).

#### 3.5.2 Scaling phase

Most scaling initiatives involved patients and the public throughout all phases of the scaling (*n* = 46).

#### 3.5.3 Ethical lenses in the rationale for PPI

Most scaling initiatives presented a teleological ethical lens (*n* = 96), that is, a ‘goal‐oriented’ lens, within which the consequentialist‐utilitarian goal of increasing scaling effectiveness was the most frequent (*n* = 93); while eight scaling initiatives presented a deontological ethical lens, that is, a ‘must do’ lens, and 16 presented both simultaneously.

#### 3.5.4 Recruitment and incentives

Thirty‐one scaling initiatives reported on the patient and public recruitment process, and 18 reported on the types of incentive used. Targeted groups and/or beneficiary populations was the profile most frequently recruited (*n* = 57), followed by community members (*n* = 44) (Additional File 12).

#### 3.5.5 Frameworks

Sixty‐seven scaling initiatives used a framework for involving patients and the public (Additional File 13).

#### 3.5.6 Stakeholders involved in PPI

Healthcare providers (*n* = 95), policymakers (*n* = 83) and researchers (*n* = 54) were the stakeholders most involved along with patients and the public.

#### 3.5.7 PPI outcomes

PPI outcomes most reported were impacts on services provided (*n* = 99), followed by impacts on PPI participants (*n* = 94) and impacts on the broader public (*n* = 75). A sex‐ or gender‐sensitive PPI strategy was identified in 23 scaling initiatives. Characteristics of PPI strategies are presented in Table 4.

**Table 4:** Characteristics of patient and public involvement (PPI) strategies.

	*n* = 120	*%*
Continuum of involvement		
Collaboration	45	37.5
Consultation	38	31.7
Co‐production	24	20.0
Information	13	10.8
Scaling phases (planning, implementing, and evaluating)		
All scaling phases	46	38.3
Implementing	29	24.2
Evaluating	13	10.8
Implementing and evaluating	11	9.2
Planning. and implementing	10	8.3
Planning	9	7.5
Planning. and evaluating	2	1.7
Ethical lenses regarding rationale for PPI^a^		
Teleological (goal‐oriented)	96	80.0
Consequentialist‐utilitarianism	93	77.5
Patient/public values	3	2.5
Mixed ethical lenses	16	13.3
Consequentialist‐utilitarianism and patient and public values	9	7.5
Duty‐rights and patient and public values	5	4.2
Consequentialist‐utilitarianism, patient and public values, and duty‐rights	2	1.7
Deontological (must do)	8	6.7
Duty‐rights	8	6.7
Type of incentivization		
Not reported	102	85.0
Financial compensation^b^	16	13.3
Nonmaterial incentives^c^	2	1.7
Recruitment		
Not reported	89	74.2
Recruited by health and social services^d^	10	8.3
Recruited by community services^e^	8	6.7
Recruited by communication media^f^	7	5.8
Recruited by scaling team network^g^	6	5.0
Patient and public recruitment profile^a^		
Targeted groups and/or beneficiary populations	57	47.5
Community members	44	36.7
Patients	25	20.8
Civil society organizations	25	20.8
Leaders	23	19.2
Volunteers	10	8.3
Users	7	5.8
Citizens	3	2.5
The public	2	1.7
Involvement framework or guide		
Not reported	67	55.8
Yes	53	44.2
Other stakeholders involved^a^		
Providers	95	79.2
Policy makers	83	69.2
Researchers	54	45.0
Funders	21	17.5
The press	18	15.0
Product makers	7	5.8
PPI outcomes		
Services provided^h^	99	82.5
PPI participants^i^	94	78.3
Broader public^j^	75	62.5
Organization or system^k^	69	57.5
Population health^l^	62	51.7
Overall cost‐effectiveness of involvement^m^	26	21.7
Sex‐ or gender‐sensitive PPI strategy		
Non reported	92	76.7
Yes	28	23.3

^a^ Mutually nonexclusive.

^b^ For example, payments, gift cards, grants, salaries.

^c^ For example, social recognition, authorship.

^d^ For example, clinics, ambulatories.

^e^ For example, community‐based organizations, community groups.

^f^ For example, radio, social media.

^g^ For example, emails, snowball sampling.

^h^ Efficiency and cost‐effectiveness of services, service availability, services quality and safety, services responsiveness to needs, utilization of services.

^i^ Knowledge, skills, empowerment, satisfaction, trust.

^j^ Accountability of organization to patient and public served, staff views on involvement, formal (written) organization or system policies, explicit change to organization or system process of decision‐making, additional connections or partnerships with other groups or organizations, funding and resources availability, visibility of organization.

^k^ Awareness or knowledge of health issues, support of the organization or system.

^l^ Level of health inequalities, population health status.

^m^ Overall cost‐effectiveness of involvement from the standpoint of the healthcare organization or system.

### 3.6 PROGRESS factors

Few PROGRESS factors were reported. Race, ethnicity or culture of the patients and public involved were reported for only 47 scaling initiatives, and socioeconomic status for only nine. For 10 scaling initiatives, sex at birth was reported, for 24 gender was reported, and for two, sexual orientation (Table 5 and Additional File 14).

**Table 5:** PROGRESS factors reporting.

	*n* = 110	*%*
*PROGRESS Framework factors*		
Place of residence	33	30.0
Race/ethnicity/culture	47	42.7
Occupation	12	10.9
Gender	24	21.8
Sex at birth	10	9.1
Socioeconomic status	9	8.2
Education	5	4.5
Social capital and networks	1	0.9
Age	17	15.5

## 4 DISCUSSION

We conducted a scoping review to identify PPI strategies and their characteristics in the science and practice of scaling in HSS. We identified a total of 110 unique reports, of which 36 were in the grey literature. We observed a marked increase in the number of publications reporting PPI in scaling over the 5‐year period from 2019 to 2023. Guides and frameworks were mostly found in the grey literature. We found 120 scaling initiatives involving patients and the public, most occurring in HICs. Items of the GRIPP2 checklist were well reported, although very few authors provided a clear definition of PPI. Most scaling initiatives were found in community‐based primary healthcare, particularly in community settings. PPI strategies were mostly found at the direct care level, with patient and public education being the most frequent. However, PPI in scaling was also significant at the policymaking level. Patients and the public were involved throughout all phases of scaling, but less so in the co‐production and information interactions of the involvement *continuum*. As for the rationale for PPI, most reports presented a consequentialist‐utilitarian ethical lens for PPI, that is, the PPI was ethically orientated by the goal of enhancing scaling effectiveness. Fewer studies reported the rationale for PPI as valuing the knowledge and experiences of patients and the public, or respecting their right or duty to participate in such initiatives. Notably, PROGRESS factors and other critical elements, such as incentivization and the recruitment process, were poorly reported. Based on these findings, we make the following observations.

First, we highlight that our review included a substantial number of reports, and a marked increase in publication in the last 5 years, revealing the recent rapid progress of evidence on PPI in scaling. In contrast, a 2017 systematic review found little evidence about PPI in scaling,^21^ and another in 2019 observed that a pitfall of the bottom‐up approach to scaling is that local PPI is difficult to replicate at scale.^59^ In spite of this recent increase in evidence, the number of records we excluded for not reporting any PPI strategies in their scaling initiatives suggests that a significant number still either do not involve patients and the public at all or, if they do, fail to report it.

Also, guides and frameworks were mostly found in the grey literature rather than published in peer‐reviewed journals. They reflect scaling initiatives that are occurring in other settings than research projects, many of them sponsored by international agencies (e.g., World Bank, World Health Organization) and designed to guide public policies. This finding can be explained by (1) the relative novelty of the domain, and (2) the importance given to both PPI and scaling at the political level by macro‐actors, who hold out high hopes that they will alleviate world health problems. The findings in the grey literature confirmed that scoping reviews are an effective knowledge synthesis strategy^60^, ^61^ for gathering a broad range of evidence on PPI in scaling, as they provide a state‐of‐the‐art overview of a domain for which the evidence is disseminated through a variety of knowledge bases (e.g., implementation reports and policy guides).^62^, ^63^ However, while scoping reviews are increasingly accepted as valid sources of scientific evidence, they rarely comply with available guidelines, lack critical appraisal, are often reported with a lack of transparency, and are unlikely to have accessible protocols.^62^, ^64^ Our study suggests scoping reviews should be taken seriously and conducted with full scientific rigour.

Second, while a recent umbrella review on evidence on scaling found that it mostly occurs in LMICs,^15^ we found that scaling that involves patients and the public mostly occurred in HICs. In HICs, the pioneering PPI experiences in HSS were associated with the patient‐centred movement originating in the United States and the United Kingdom,^5^ which might explain why these countries are at the top of the list. However, PPI in LMICs is shaped by different historical contexts and processes, and may take a different form and using different terms than those disseminated in HICs.^65^ For example, in Brazil, PPI may be referred to as ‘social participation’ and has been integral to the healthcare system since its establishment in 1988.^66^ On the other hand, researchers may consider patient involvement ‘less urgent’ in LMICs that lack basic HSS; also poverty, cultural stigma around illness, and patients’ not valuing their own voice or experience all prevent more patient involvement in research.^67^ Patient and public mobilization, leveraging partnerships for financial support, peer‐to‐peer mentorship and psychosocial support and education could increase PPI in LMICs.^67^ Furthermore, south‐north collaborations have been identified as a cornerstone in scaling science^15^ and could enhance equity in PPI worldwide.^67^ Nevertheless, further research is required to determine whether it is the structural and situational constraints in LMICs that hinder the PPI in scaling in these contexts, or whether PPI exists in scaling initiatives in these regions but is reported using different terminology^65^ and so was not retrieved by our review.

Third, based on the GRIPP2 checklist, few scaling initiatives provided a definition for PPI, confirming that there is a need for better definition of key concepts in the literature on scaling. However, in the majority of cases, the primary focus of the authors was not on reporting PPI but rather on some other aspect of the scaling initiative. Even the most prominent frameworks^15^, ^18^ for scaling did not include a definition of PPI, despite recognizing stakeholder involvement as a key practice for successful scaling initiatives and using PPI strategies in their own internal scaling initiatives.^68^ Given the increasing interest in PPI and its importance in scaling initiatives,^32^, ^69^ we highlight the importance of providing a definition of PPI when reporting future research.

Fourth, PPI strategies in scaling were found predominantly in the context of community‐based primary health care. Most were related to direct care activities, with patient education and behaviour change interventions being the most frequent. This finding is coherent with a global trend in scaling initiatives that mostly focus on infectious diseases and crisis contexts,^15^ such as HIV and maternal and child health, both most reported health issues in this review. With these health issues, scaling requires direct care level PPI strategies, such as the use of educational resources to increase knowledge about washing hands to prevent virus contamination (maternal and child health); or strategies aimed at increasing group commitment to changing a specific behaviour, such as the use of condoms (HIV). Methods for enacting these PPI strategies might consist of organizing information workshops for community leaders or using mass media to deliver prevention messages – typical traditional public health practices in community settings.^70^ In addition, implementation of HSS in community‐based primary healthcare initiatives are known for involving the communities in question, even if they have never named it PPI.^66^ That PPI in scaling is found predominantly at the primary care level also suggests a lack of skills necessary for PPI at the secondary and tertiary levels of care, such as in hospitals and diagnostic centres. These are usually more specialized and distant from both the community and policy decision‐making contexts, and an important barrier in these contexts is competing organizational priorities.^71^ In addition, the nature of hospital and specialist care is less person‐centred, tending to focus more on diseases and on parts of bodies and less on public health and on the whole person.

Additionally, the significant frequency with which the PPI strategies in scaling occurred at the policymaking level shows a marked difference with reports of PPI strategies found in other kinds of health and social interventions. In a review of 139 reviews on patient and family involvement, 131 reviews focused on strategies at the level of direct patient care, only five reviews focused on the healthcare and organization level, and none at all at the policy level.^72^ Our finding of a significant number of strategies occurring at the policy level is encouraging, and reveals the potential for PPI in councils or committees to develop system‐level scaling policies and priorities, as suggested by WHO guidelines.^13^

Fifth, patients and the public are increasingly involved in all phases of scaling. This is grounds for optimism. However, co‐production, the most involved form of PPI, is not yet a well‐established practice in scaling. In our experience, difficulties in communicating in plain language can be a barrier to PPI.^67^, ^73^ Often new sciences develop increasingly specialized vocabularies that become less and less accessible to laypeople. Scaling scientists should take care not to indulge in creating new vocabularies that increase the distance between them and the people they are trying to benefit. Reaching a consensus on scaling terms both in scientific publishing and for lay people may help the dialogue and provide further incentive for PPI in scaling initiatives. Also, PPI requires time and funding^74^ that is often not anticipated, involving planning, infrastructure, training, resources, and other contextual factors.^75^

In addition, the consequentialist‐utilitarian ethical lens for PPI, that is, a goal‐oriented ethical rationale for PPI, was more frequent than other ethical lenses. While increased effectiveness is undoubtedly an important outcome, ethical reasoning based on patient and public values is also fundamental because it acknowledges that patients’ knowledge and backgrounds are essential for justifying scaling^14^, ^55^ and for eventually achieving the desired impacts. Duty‐rights perspectives are also important, that is, including PPI in scaling because it is both a duty to patients and the public and their right to be involved. We suggest that a mix of ethical lenses in approaching patient involvement is required, fostering an understanding of PPI as both an instrument of effectiveness and an ethical imperative.^76^ In scaling science, the different ethical lenses underlying PPI have an impact on study design, including on decisions such as who to involve, in what areas, how, and when, and could explain why expectations for PPI are frequently not met.^56^, ^77^ Explicitly identifying the ethical lenses that justify PPI could help manage the PPI process, keep goals realistic, and guide selection of appropriate approaches.

Lastly, the lack of information on incentivization and recruitment processes of patients and the public underlines a need for improved systematization and transparency in reporting PPI strategies in scaling. Clear and detailed documentation of these critical requirements will not only enhance the overall understanding of the phenomena but also facilitate the replication and adaptation of successful strategies in diverse contexts, ultimately contributing to the progress of knowledge in this field. Also, this may help scaling practitioners to better plan PPI in scaling initiatives, taking into account the extra time, infrastructure and funding that is needed.^74^

This review provides evidence on where and how PPI strategies in scaling in HSS are occurring and being reported, that is, which countries are involved in this practice, in which settings, and what PPI strategies are being used. This information on PPI elsewhere could inspire scaling practitioners to fully explore the potential of co‐construction in their own contexts, and to adopt an ethical approach to scaling that incorporates the knowledge and experience of patients and the public and respects their right to participate. Future qualitative research on real experiences should also be encouraged to document the local challenges of PPI in scaling as experienced on the ground.^78^

Our review has several limitations. Our eligibility criteria, which were based on reported methods and outcomes, served as a high‐quality threshold for selecting evidence, but may have resulted in some missed records. On the other hand, this rigorous approach ensured that the evidence was based on documented outcomes. Finally, we did not contact authors to ask for potentially available information that may have simply been missing from the report.

## 5 CONCLUSIONS

We found a significant body of evidence on PPI in scaling, showing a sharp increase in the last few years. Nevertheless, the topic of PPI in scaling deserves further investigation as a distinct domain. Trends in PPI in scaling will not necessarily follow scaling trends. For example, while a previous systematic review showed that scaling initiatives mostly occur in LMICs, our review found that PPI in scaling mostly occurs in HICs. More research is needed to investigate how both scaling and PPI are occurring and being communicated in LMICs, where they have long been practiced.^15^ Likewise, trends in PPI in scaling will not necessarily follow trends in PPI. For example, another review showed PPI occurred very rarely in the context of policymaking, while our review showed it occurred frequently in that context. Also, our findings demonstrate that scaling initiatives in HSS are often misreported. This was especially evident in the lack of PPI definitions as well as in the lack of reported PPI recruitment and incentivization processes. Additional efforts are warranted to document scaling participants’ experiences in PPI and to determine how we can meaningfully and equitably involve patients and the public in both the science and practice of scaling HSS in all contexts, especially those where it is currently neglected.

## AUTHOR CONTRIBUTIONS

**Roberta de Carvalho Corôa**: Conceptualization; investigation; writing—original draft; methodology; validation; visualization; writing—review and editing; project administration; data curation; supervision; software; formal analysis; resources. **Ali Ben Charif**: Conceptualization; funding acquisition; investigation; writing—review and editing; methodology; supervision; software; validation; visualization; formal analysis; resources; data curation; project administration. **Vincent Robitaille**: Investigation; validation; writing—review and editing; formal analysis; visualization. **Diogo Gonçalves Vianna Mochcovitch**: Investigation; validation; visualization; writing—review and editing; formal analysis; writing—original draft. **Mamane Abdoulaye Samri**: Methodology; validation; investigation; visualization; writing—review and editing; formal analysis. **Talagbé Gabin Akpo**: Investigation; methodology; validation; visualization; writing—review and editing; formal analysis; software. **Amédé Gogovor**: Conceptualization; Investigation; methodology; writing—review and editing; validation; visualization. **Virginie Blanchette**: Investigation; methodology; writing—review and editing; validation; visualization. **Lucas Gomes Souza**: Investigation; validation; visualization; writing—review and editing; software; formal analysis. **Kathy Kastner**: Conceptualization; investigation; validation; methodology; visualization; writing—review and editing. **Amélie M. Achim**: Methodology; validation; visualization; writing—review and editing; investigation. **Robert K. D. McLean**: Conceptualization; investigation; funding acquisition; methodology; validation; visualization; writing—review and editing. **Andrew Milat**: Conceptualization; investigation; funding acquisition; methodology; validation; visualization; writing—review and editing. **France Légaré**: Conceptualization; Investigation; funding acquisition; writing—original draft; methodology; visualization; validation; writing—review and editing; project administration; data curation; supervision; resources; formal analysis.

## Research on Patient‐Oriented Scaling‐up (RePOS) Network

**Table:** 

**Last name, first name**	**Country**	**Sex**	**Type of stakeholder**	**Email**
Abdoulaye Samri, Mamane	Canada	M	Trainee, investigator	mamane.abdoulaye-samri.1@ulaval.ca
Achim, Amélie M.	Canada	F	Investigator	amelie.achim@fmed.ulaval.ca
Beleno, Ron	Canada	M	Patient instructor	ron@rb33.com
Ben Charif, Ali	Canada, Comoros	M	Investigator	ali.bencharif@cubecxpert.ca
Blair, Louisa	Canada	F	Patient, Editor	louisablair@outlook.com
Blanchette, Virginie	Canada	F	Investigator	virginie.blanchette@uqtr.ca
Corôa, Roberta de Carvalho	Brazil, Canada	F	Trainee, investigator	roberta.de-carvalho-coroa.1@ulaval.ca
Akpo, Talagb Gabin	Canada	M	Trainee	talagbe-gabin.akpo.1@ulaval.ca
Geiger, Friedemann	Germany	M	Provider, Investigator	f.geiger@uksh.de
Ghiron, Laura	USA	F	Policy Maker	ljghiron@umich.edu
Gogovor, Amédé	Canada	M	Investigator	amede.gogovor@gmail.com
Gomes Souza, Lucas	Brazil, Canada	M	Provider, Investigator	lucas.gomes-souza.1@ulaval.ca
Guay‐Bélanger, Sabrina	Canada	F	Investigator	sabrina.guay-belanger.ciussscn@ssss.gouv.qc.ca
Kastner, Kathy	Canada	F	Patient instructor	kathyk@bestendings.com
Légaré, France	Canada	F	Provider, Investigator	france.legare@mfa.ulaval.ca
McLean, Robert	Canada	M	Policy Maker, Investigator	rmclean@idrc.ca
Milat, Andrew J	Australia	M	Policy Maker, Investigator	andrew.milat@sydney.edu.au
Mochcovitch, Diogo Gonçalves Vianna	Brazil, Canada	M	Investigator	diogo.mochcovitch.ciussscn@ssss.gouv.qc.ca
Paquette, Jean‐Sébastien	Canada	M	Provider, Investigator	jspaquette.lab@gmail.com
Plourde, Karine V.	Canada	F	Knowledge user	karine.plourde.1@ulaval.ca
Robitaille, Vincent	Canada	M	Student	vincent.robitaille.9@ulaval.ca
Straus, Sharon	Canada	F	Provider, Investigator	sharon.straus@utoronto.ca
Wolfenden, Luke	Australia	M	Investigator	luke.wolfenden@hnehealth.nsw.gov.au
Zomahoun, Hervé Tchala Vignon	Canada	M	Investigator	herve.zomahoun.ciussscn@ssss.gouv.qc.ca

## CONFLICT OF INTEREST STATEMENT

The authors declare no conflict of interest.

## Supporting information

Supporting information.

Supporting information.

Supporting information.

Supporting information.

Supporting information.

Supporting information.

Supporting information.

Supporting information.

Supporting information.

Supporting information.

Supporting information.

Supporting information.

Supporting information.

Supporting information.

## Data Availability

The data that supports the findings of this study are available in the Supporting Infomation of this article.
