# Supplementary material for: Strategies for involving patients and the public in scaling initiatives in health and social services: A scoping review
Source: Health Expect. 2024 Jun 5;27(3):e14086. doi: 10.1111/hex.14086 (PMC11150745; doi:10.1111/hex.14086)
Supplement: Supplementary file 2 — Supporting information. [file HEX-27-e14086-s005.docx]

**Additional File 2 - SAGER guidelines**

NA – Not applicable

| Research approaches ✓ | |  |
| --- | --- | --- |
|  | ✓ Are the concepts of gender and/or sex used in your research project? | Yes |
|  | ✓ If yes, have you explicitly defined the concepts of gender and/or sex? Is it clear what aspects of gender and/or sex are being examined in your study? | Yes, Additional File 6 |
|  | ✓ If no, do you consider this to be a significant limitation? Given existing knowledge in the relevant literature, are there plausible gender and/or sex factors that should have been considered? If you consider sex and/or gender to be highly relevant to your proposed research, the research design should reflect this | NA |
| Research questions and hypotheses | |  |
|  | ✓ Does your research question(s) or hypothesis/es make reference to gender and/or sex, or relevant groups or phenomena? (e.g., differences between males and females, differences among women, seeking to understand a gendered phenomenon such as masculinity) | No |
| Literature review | |  |
|  | ✓ Does your literature review cite prior studies that support the existence (or lack) of significant differences between women and men, boys and girls, or males and females? | No, because our research question/ hypothesis makes no reference to gender and/or sex, or relevant groups or phenomena |
|  | ✓ Does your literature review point to the extent to which past research has taken gender or sex into account? | Yes |
| Research methods | |  |
|  | ✓ Is your sample appropriate to capture gender and/or sex-based factors? | Yes |
|  | ✓ Is it possible to collect data that are disaggregated by sex and/or gender? | Yes, but we did not proceed with disaggregated data, since research on sex and gender was not included in our main research question |
|  | ✓ Are the inclusion and exclusion criteria well justified with respect to sex and/or gender? (Note: this pertains to human and animal subjects and biological systems that are not whole organisms) | NA |
|  | ✓ Is the data collection method proposed in your study appropriate for investigation of sex and/or gender? | Yes |
|  | ✓ Is your analytic approach appropriate and rigorous enough to capture gender and/or sex-based factors? | Yes |
| Ethics | |  |
|  | ✓ Does your study design account for the relevant ethical issues that might have particular significance with respect to gender and/or sex? (e.g., inclusion of pregnant women in clinical trials) | Yes |
| *Source: Adapted from Canadian Institutes of Health Research.* | |  |
