# Supplementary material for: Strategies for involving patients and the public in scaling initiatives in health and social services: A scoping review
Source: Health Expect. 2024 Jun 5;27(3):e14086. doi: 10.1111/hex.14086 (PMC11150745; doi:10.1111/hex.14086)
Supplement: Supplementary file 3 — Supporting information. [file HEX-27-e14086-s007.docx]

**Additional File 3 – GRIPP2 for the study**

| **Section and topic** | **Item** | **Reported on page No** |
| --- | --- | --- |
| 1: Background | Report the definition of PPI used in the study and how it links to comparable studies | p. 5 |
|  | Report the theoretical rationale and any theoretical influences relating to PPI in the study | p.4 – p.6 |
| 2. Aim | Report the aim of the study | p.6 |
| 2: Methods | Does the paper provide a clear description of methods by which patients and the public were involved? | p.6 – p.7 |
|  | Provide a description of patients, carers, and the public involved with the PPI activity in the study | p.6 – p.7, and The RePOS Network list in the end of the article |
| 4: Discussion and conclusions | Report the results of PPI in the study, including both positive and negative outcomes | p.6 – p.7, and p.13 |
|  | Comment on how PPI influenced the study overall. Describe positive and negative effects | p.6 – p 7 |
| 5: Reflections/critical perspective | Critical comment on the study, a reflection on the things that went well and those that did not, so that others can learn from it | p.24 |
