# Supplementary material for: Strategies for involving patients and the public in scaling initiatives in health and social services: A scoping review
Source: Health Expect. 2024 Jun 5;27(3):e14086. doi: 10.1111/hex.14086 (PMC11150745; doi:10.1111/hex.14086)
Supplement: Supplementary file 4 — Supporting information. [file HEX-27-e14086-s014.docx]

**Additional File 4 – Search Strategy**

**Databases**

1. Ovid Medline
2. Embase
3. Web of Science
4. Cinahl
5. PsycINFO
6. ERIC
7. Cochrane Library
8. Sociological Abstract
9. Academic Search Premier

**Gray Literature**

1. Google
2. CADTH
3. IDRC
4. NICE
5. NSW
6. CFHI
7. IHI
8. NIRN
9. SISEP
10. WHO-ExpandNET
11. Australian Prevention
12. ARHQ
13. Global Community

### Medline-Ovid- (2020-09-28)

| **Concepts** | **Search strategy keywords** | **Search** | **# Results** |
| --- | --- | --- | --- |
| Scaling (Controlled Vocabulary) | "diffusion of innovation"/ or Organizational Innovation/ | #1 | 40 860 |
| Scaling (Free text) | ("scal* up" or "scal* out").ab,kf,kw,ti. | #2 | 20 048 |
|  | (("scaling" or widespread or spread? or spreading or "rolling out" or "roll out" or "rolls out" or "rolled out" or upscaling or scalability or scalable) adj5 (innovation? or intervention? or technolog* or practice* or care or initiative* or program* or product? or therap* or service* or strateg* or change? or proces*)).ab,kf,kw,ti. | #3 | 31 997 |
|  | ((bring* or brought or taking or take* or increas* or going or implement* or econom*) adj5 scal* adj5 (innovation? or intervention? or technolog* or practice* or care or initiative* or program* or product? or therap* or service* or strateg* or change? or proces*)).ab,kf,kw,ti. | #4 | 3 096 |
| Scaling (Free text) | #2 or #3 or #4 | #5 | 52 204 |
| Scaling | #1 or #5 | #6 | 92 598 |

| Patient partner (Controlled vocabulary) | exp Community-Based Participatory Research/ or exp Community Participation/ or stakeholder participation/ | #7 | 48 156 |
| --- | --- | --- | --- |
| Patient partner in research (Free text) | ((caregiver* or care-giver* or citizen* or client* or communit* or consumer* or "family carer*" or lay or patient* or "peer helper*" or public or stakeholder* or survivor* or user*) adj2 (collaborat* or cooperat* or co-operat* or coproduc* or co-produc* or engag* or includ* or involve* or "joint effort*" or mobilis* or mobiliz* or participat* or partner* or "work together" or "working together")).ab,kf,kw,ti | #8 | 382 738 |
| Research co-building (Free text) | (Co-build* or co-constr* or co-creation or coproduc* or co-produc* ).ab,kf,kw,ti. | #9 | 5 662 |
| Patient partner (Free text) | #8 or #9 | #10 | 387 793 |
| Patient partner | #7 or #10 | #11 | 422 269 |
| Strategy (Controlled vocabulary) | Methods/ or exp Models, Theoretical/ | #12 | 2 006 293 |
| Strategy (Free text) | (Action or actions or approach* or example* or "frame work*" or Framework* or guidance* or guide or guides or guideline* or how or hows or methodolog* or model or models or paradigm* or practice* or principle* or process or recommend* or stratagem* or strateg* or tool or toolkit* or tools).ab,kf,kw,ti. | #13 | 8 937 894 |
| Strategy | #12 or #13 | #14 | 9 791 826 |
| Total Result | #6 and #11 and #14 | #15 | 3 046 |

### Medline-Ovid- (2024-02-05)

| 1 | diffusion of innovation/ or Organizational Innovation/ | 42305 |
| --- | --- | --- |
| 2 | ("scal* up" or "scal* out").ab,kf,kw,ti. | 29833 |
| 3 | (("scaling" or widespread or spread? or spreading or "rolling out" or "roll out" or "rolls out" or "rolled out" or upscaling or scalability or scalable) adj5 (innovation? or intervention? or technolog* or practice* or care or initiative* or program* or product? or therap* or service* or strateg* or change? or proces*)).ab,kf,kw,ti. | 46621 |
| 4 | ((bring* or brought or taking or take* or increas* or going or implement* or econom*) adj5 scal* adj5 (innovation? or intervention? or technolog* or practice* or care or initiative* or program* or product? or therap* or service* or strateg* or change? or proces*)).ab,kf,kw,ti. | 4788 |
| 5 | 2 or 3 or 4 | 76520 |
| 6 | 1 or 5 | 118318 |
| 7 | exp Community-Based Participatory Research/ or exp Community Participation/ or stakeholder participation/ |  |
| 8 | ((caregiver* or care-giver* or citizen* or client* or communit* or consumer* or "family carer*" or lay or patient* or "peer helper*" or public or stakeholder* or survivor* or user*) adj2 (collaborat* or cooperat* or co-operat* or coproduc* or co-produc* or engag* or includ* or involve* or "joint effort*" or mobilis* or mobiliz* or participat* or partner* or "work together" or "working together")).ab,kf,kw,ti. | 55458 |
| 9 | (Co-build* or co-constr* or co-creation or coproduc* or co-produc*).ab,kf,kw,ti. | 9674 |
| 10 | 8 or 9 | 580566 |
| 11 | 7 or 10 | 618089 |
| 12 | Methods/ or exp Models, Theoretical/ | 2191647 |
| 13 | (Action or actions or approach* or example* or "frame work*" or Framework* or guidance* or guide or guides or guideline* or how or hows or methodolog* or model or models or paradigm* or practice* or principle* or process or recommend* or stratagem* or strateg* or tool or toolkit* or tools).ab,kf,kw,ti. | 11611793 |
| 14 | 12 or 13 | 12510097 |
| 15 | 6 and 11 and 14 | 4399 |
| 16 | limit 15 to ed=20200928-20240205 | 1108 |
| FINAL | limit 15 to ed=20200928-20240205 | 1108 |

###

### Embase-Elsevier (2020-09-29)

| **Concepts** | **Search strategy keywords** | **Search** | **# Results** |
| --- | --- | --- | --- |
| Scaling (Controlled Vocabulary) | 'scale up'/exp or 'scaling'/exp or 'scalability'/exp | #1 | 11 581 |
| Scaling (Free text) | ("scal* up" or "scal* out"):ti,ab,kw | #2 | 25 680 |
|  | (("scaling" or widespread or spread$ or spreading or "rolling out" or "roll out" or "rolls out" or "rolled out" or upscaling or scalability or scalable) NEAR/5 (innovation$ or intervention$ or technolog* or practice* or care or initiative* or program* or product$ or therap* or service* or strateg* or change$ or proces*)):ti,ab,kw | #3 | 40 332 |
|  | ((bring* or brought or taking or take* or increas* or going or implement* or econom*) NEAR/5 scal* NEAR/5 (innovation$ or intervention$ or technolog* or practice* or care or initiative* or program* or product$ or therap* or service* or strateg* or change$ or proces*)):ti,ab,kw | #4 | 3 842 |
| Scaling (Free text) | #2 or #3 or #4 | #5 | 66 054 |
| Scaling | #1 or #5 | #6 | 69 238 |

| Patient partner (Controlled vocabulary) | 'community participation'/exp or 'stakeholder engagement'/exp | #7 | 5 268 |
| --- | --- | --- | --- |
| Patient partner in research (Free text) | ((caregiver* or care-giver* or citizen* or client* or communit* or consumer* or "family carer*" or lay or patient* or "peer helper*" or public or stakeholder* or survivor* or user*) NEAR/2 (collaborat* or cooperat* or co-operat* or coproduc* or co-produc* or engag* or includ* or involve* or "joint effort*" or mobilis* or mobiliz* or participat* or partner* or "work together" or "working together")):ti,ab,kw | #8 | 670 226 |
| Research co-building (Free text) | (Co-build* or co-constr* or co-creation or coproduc* or co-produc*):ti,ab,kw | #9 | 6 852 |
| Patient partner (Free text) | #8 or #9 | #10 | 676 325 |
| Patient partner | #7 or #10 | #11 | 681 778 |
| Strategy (Controlled vocabulary) | 'action'/exp or 'framework'/exp or 'guidance'/exp or 'guide'/exp or 'guideline'/exp or 'methodology'/de or 'model'/de or 'practice guideline'/exp or 'process'/exp or 'recommendations'/exp or 'strategy'/exp or 'tool'/exp | #12 | 2 650 958 |
| Strategy (Free text) | (Action or actions or approach* or example* or "frame work*" or Framework* or guidance* or guide or guides or guideline* or how or hows or methodolog* or model or models or paradigm* or practice* or principle* or process or recommend* or stratagem* or strateg* or tool or toolkit* or tools):ti,ab,kw | #13 | 11 486 072 |
| Strategy | #12 or #13 | #14 | 12 557 303 |
| Total Result | #6 and #11 and #14 | #15 | 2 391 |

### Embase-Elsevier (2024-02-05)

| 1 | scale up'/exp OR 'scale up' OR 'scaling'/exp OR 'scaling' OR 'scalability'/exp OR 'scalability' | 104856 |
| --- | --- | --- |
| 2 | scal* up':ti,ab,kw OR 'scal* out':ti,ab,kw | 36593 |
| 3 | (('scaling' OR widespread OR spread$ OR spreading OR 'rolling out' OR 'roll out' OR 'rolls out' OR 'rolled out' OR upscaling OR scalability OR scalable) NEAR/5 (innovation$ OR intervention$ OR technolog* OR practice* OR care OR initiative* OR program* OR product$ OR therap* OR service* OR strateg* OR change$ OR proces*)):ti,ab,kw | 57421 |
| 4 | ((bring* OR brought OR taking OR take* OR increas* OR going OR implement* OR econom*) NEAR/5 scal* NEAR/5 (innovation$ OR intervention$ OR technolog* OR practice* OR care OR initiative* OR program* OR product$ OR therap* OR service* OR strateg* OR change$ OR proces*)):ti,ab,kw | 5769 |
| 5 | #2 OR #3 OR #4 | 93850 |
| 6 | #1 OR #5 | 163340 |
| 7 | community participation'/exp OR 'stakeholder engagement'/exp | 14079 |
| 8 | ((caregiver* OR 'care giver*' OR citizen* OR client* OR communit* OR consumer* OR 'family carer*' OR lay OR patient* OR 'peer helper*' OR public OR stakeholder* OR survivor* OR user*) NEAR/2 (collaborat* OR cooperat* OR 'co operat*' OR coproduc* OR 'co produc*' OR engag* OR includ* OR involve* OR 'joint effort*' OR mobilis* OR mobiliz* OR participat* OR partner* OR 'work together' OR 'working together')):ti,ab,kw | 983571 |
| 9 | co build*':ti,ab,kw OR 'co constr*':ti,ab,kw OR 'co creation':ti,ab,kw OR coproduc*:ti,ab,kw OR 'co produc*':ti,ab,kw | 11381 |
| 10 | #8 OR #9 | 993107 |
| 11 | #7 OR #10 | 1000298 |
| 12 | action'/exp OR 'framework'/exp OR 'guidance'/exp OR 'guide'/exp OR 'guideline'/exp OR 'methodology'/de OR 'model'/de OR 'practice guideline'/exp OR 'process'/exp OR 'recommendations'/exp OR 'strategy'/exp OR 'tool'/exp | 16797225 |
| 13 | action:ti,ab,kw OR actions:ti,ab,kw OR approach*:ti,ab,kw OR example*:ti,ab,kw OR 'frame work*':ti,ab,kw OR framework*:ti,ab,kw OR guidance*:ti,ab,kw OR guide:ti,ab,kw OR guides:ti,ab,kw OR guideline*:ti,ab,kw OR how:ti,ab,kw OR hows:ti,ab,kw OR methodolog*:ti,ab,kw OR model:ti,ab,kw OR models:ti,ab,kw OR paradigm*:ti,ab,kw OR practice*:ti,ab,kw OR principle*:ti,ab,kw OR process:ti,ab,kw OR recommend*:ti,ab,kw OR stratagem*:ti,ab,kw OR strateg*:ti,ab,kw OR tool:ti,ab,kw OR toolkit*:ti,ab,kw OR tools:ti,ab,kw | 14786675 |
| 14 | #12 OR #13 | 20598336 |
| 15 | #6 AND #11 AND #14 | 5524 |
| 16 | [29-09-2020]/sd NOT [06-02-2024]/sd | 7273086 |
| FINAL | #15 AND #16 | 2388 |

### Web of Science (2020-09-29)

| **Concepts** | **Search strategy keywords** | **Search** | **# Results** |
| --- | --- | --- | --- |
| Scaling (Free text) | TS=("scal* up" or "scal* out") | #1 | 53 735 |
|  | TS=(("scaling" or widespread or spread$ or spreading or "rolling out" or "roll out" or "rolls out" or "rolled out" or upscaling or scalability or scalable) NEAR/4 (innovation$ or intervention$ or technolog* or practice* or care or initiative* or program* or product$ or therap* or service* or strateg* or change$ or proces*)) | #2 | 81 086 |
|  | TS=((bring* or brought or taking or take* or increas* or going or implement* or econom*) NEAR/4 scal* NEAR/4 (innovation$ or intervention$ or technolog* or practice* or care or initiative* or program* or product$ or therap* or service* or strateg* or change$ or proces*)) | #3 | 10 694 |
|  | #1 or #2 or #3 | #4 | 138 841 |

| Patient partner in research (Free text) | TS=((caregiver* or care-giver* or citizen* or client* or communit* or consumer* or "family carer*" or lay or patient* or "peer helper*" or public or stakeholder* or survivor* or user*) NEAR/1 (collaborat* or cooperat* or co-operat* or coproduc* or co-produc* or engag* or includ* or involve* or "joint effort*" or mobilis* or mobiliz* or participat* or partner* or "work together" or "working together")) | #5 | 459 323 |
| --- | --- | --- | --- |
| Research co-building (Free text) | TS=(Co-build* or co-constr* or co-creation or coproduc* or co-produc*) | #6 | 28 652 |
| Patient partner | #5 or #6 | #7 | 485 071 |
| Strategy (Free text) | TS=(Action or actions or approach* or example* or "frame work$" or Framework$ or guidance$ or guide$ or guideline$ or how$ or methodolog* or model$ or paradigm* or practice$ or principle$ or process or recommend* or stratagem* or strateg* or toolkit$ or tool$) | #8 | 24 102 703 |
| Health and Social Services (Free text) | TS=(care or chiroprati* or clinic* or communit* or dentist* or diagnos* or disease* or drug* or healing* or health* or illness or infection* or injur* or medicin* or medica* or nurs* or nutrition* or optometr* or orthoptic* or patholog* or patient or pharma* or pharmaceutic* or pill* or placebo* or poverty or pregnan* or prevention* or psychiatr* or psycholog* or psychosocial* or remed* or social* or surger* or therap* or treatment* or unemploy* or violence* or wound*) | #9 | 21 808 607 |
| Total Result | #4 and #7 and #9 | #10 | 2 097 |

### Web of Science (2024-02-05)

| 1 | TS=(("scal* up" or "scal* out")) | 77006 |
| --- | --- | --- |
| 2 | TS=(("scaling" or widespread or spread$ or spreading or "rolling out" or "roll out" or "rolls out" or "rolled out" or upscaling or scalability or scalable) NEAR/4 (innovation$ or intervention$ or technolog* or practice* or care or initiative* or program* or product$ or therap* or service* or strateg* or change$ or proces*)) | 115669 |
| 3 | TS=((bring* or brought or taking or take* or increas* or going or implement* or econom*) NEAR/4 scal* NEAR/4 (innovation$ or intervention$ or technolog* or practice* or care or initiative* or program* or product$ or therap* or service* or strateg* or change$ or proces*)) | 14950 |
| 4 | #1 OR #2 OR #3 | 197453 |
| 5 | TS=((caregiver* or care-giver* or citizen* or client* or communit* or consumer* or "family carer*" or lay or patient* or "peer helper*" or public or stakeholder* or survivor* or user*) NEAR/1 (collaborat* or cooperat* or co-operat* or coproduc* or co-produc* or engag* or includ* or involve* or "joint effort*" or mobilis* or mobiliz* or participat* or partner* or "work together" or "working together")) | 700973 |
| 6 | TS=(Co-build* or co-constr* or co-creation or coproduc* or co-produc*) | 47534 |
| 7 | #5 OR #6 | 742703 |
| 8 | TS=(Action or actions or approach* or example* or "frame work$" or Framework$ or guidance$ or guide$ or guideline$ or how$ or methodolog* or model$ or paradigm* or practice$ or principle$ or process or recommend* or stratagem* or strateg* or toolkit$ or tool$) | 31379729 |
| 9 | TS=(care or chiroprati* or clinic* or communit* or dentist* or diagnos* or disease* or drug* or healing* or health* or illness or infection* or injur* or medicin* or medica* or nurs* or nutrition* or optometr* or orthoptic* or patholog* or patient or pharma* or pharmaceutic* or pill* or placebo* or poverty or pregnan* or prevention* or psychiatr* or psycholog* or psychosocial* or remed* or social* or surger* or therap* or treatment* or unemploy* or violence* or wound*) | 27769329 |
| 10 | #4 AND #7 AND #9 | 4056 |
| 11 | LD=2020-09-29/2024-02-05 | 12022490 |
| 12 | #10 AND #11 | 1614 |
| FINAL | #10 AND #11 |  |

### Cinahl-EBSCO-(2020-09-29)

| **Concepts** | **Search strategy keywords** | **Search** | **# Results** |
| --- | --- | --- | --- |
| Scaling (Controlled Vocabulary) | (MH "Diffusion of Innovation+") | #1 | 15 354 |
| Scaling (Free text) | TI ( ("scal* up" or "scal* out") )  OR AB ( ("scal* up" or "scal* out") )  OR SU ( ("scal* up" or "scal* out" ) ) | #2 | 4 031 |
|  | TI (("scaling" or widespread or spread# or spreading or "rolling out" or "roll out" or "rolls out" or "rolled out" or upscaling or scalability or scalable) N4 (innovation# or intervention# or technolog* or practice* or care or initiative* or program* or product# or therap* or service* or strateg* or change# or proces*))  OR AB (("scaling" or widespread or spread# or spreading or "rolling out" or "roll out" or "rolls out" or "rolled out" or upscaling or scalability or scalable) N4 (innovation# or intervention# or technolog* or practice* or care or initiative* or program* or product# or therap* or service* or strateg* or change# or proces*))  OR SU (("scaling" or widespread or spread# or spreading or "rolling out" or "roll out" or "rolls out" or "rolled out" or upscaling or scalability or scalable) N4 (innovation# or intervention# or technolog* or practice* or care or initiative* or program* or product# or therap* or service* or strateg* or change# or proces*) ) | #3 | 7 796 |
|  | TI ( (bring* or brought or taking or take* or increas* or going or implement* or econom*) N4 scal* N4 (innovation# or intervention# or technolog* or practice* or care or initiative* or program* or product# or therap* or service* or strateg* or change# or proces*) )  OR AB ( (bring* or brought or taking or take* or increas* or going or implement* or econom*) N4 scal* N4 (innovation# or intervention# or technolog* or practice* or care or initiative* or program* or product# or therap* or service* or strateg* or change# or proces*) )  OR SU ( (bring* or brought or taking or take* or increas* or going or implement* or econom*) N4 scal* N4 (innovation# or intervention# or technolog* or practice* or care or initiative* or program* or product# or therap* or service* or strateg* or change# or proces*) ) | #4 | 1 172 |
| Scaling (Free text) | S2 OR S3 OR S4 | #5 | 11 932 |
| Scaling | S1 OR S5 | #6 | 27 074 |

| Patient partner (Controlled vocabulary) | (MH "Consumer Participation") OR (MH "Stakeholder Participation") | #7 | 21 341 |
| --- | --- | --- | --- |
| Patient partner in research (Free text) | TI ((caregiver* or care-giver* or citizen* or client* or communit* or consumer* or "family carer*" or lay or patient* or "peer helper*" or public or stakeholder* or survivor* or user*) N1 (collaborat* or cooperat* or co-operat* or coproduc* or co-produc* or engag* or includ* or involve* or "joint effort*" or mobilis* or mobiliz* or participat* or partner* or "work together" or "working together"))  OR AB ((caregiver* or care-giver* or citizen* or client* or communit* or consumer* or "family carer*" or lay or patient* or "peer helper*" or public or stakeholder* or survivor* or user*) N1 (collaborat* or cooperat* or co-operat* or coproduc* or co-produc* or engag* or includ* or involve* or "joint effort*" or mobilis* or mobiliz* or participat* or partner* or "work together" or "working together"))  OR SU ((caregiver* or care-giver* or citizen* or client* or communit* or consumer* or "family carer*" or lay or patient* or "peer helper*" or public or stakeholder* or survivor* or user*) N1 (collaborat* or cooperat* or co-operat* or coproduc* or co-produc* or engag* or includ* or involve* or "joint effort*" or mobilis* or mobiliz* or participat* or partner* or "work together" or "working together") ) | #8 | 155 996 |
| Research co-building (Free text) | TI ( Co-build* or co-constr* or co-creation or coproduc* or co-produc* )  OR AB ( Co-build* or co-constr* or co-creation or coproduc* or co-produc* )  OR SU ( Co-build* or co-constr* or co-creation or coproduc* or co-produc* ) | #9 | 1 744 |
| Patient partner (Free text) | S8 OR S9 | #10 | 157 259 |
| Patient partner | S7 OR S10 | #11 | 157 259 |
| Strategy (Controlled vocabulary) | (MH "Conceptual Framework") OR (MH "Models, Theoretical+") OR (MH "Practice Guidelines") OR (MH "Research Methodology") OR (MH "Study Methods") | #12 | 304 730 |
| Strategy (Free text) | TI ( Action or actions or approach* or example*or "frame work*" or Framework* or guidance* or guide or guides or guideline* or how or hows or methodolog* or model or models or paradigm* or practice* or principle* or process or recommend* or stratagem* or strateg* or tool or toolkit* or tools )  OR AB ( Action or actions or approach* or example* or "frame work*" or Framework* or guidance* or guide or guides or guideline* or how or hows or methodolog* or model or models or paradigm* or practice* or principle* or process or recommend* or stratagem* or strateg* or tool or toolkit* or tools )  OR SU ( Action or actions or approach* or example* or "frame work*" or Framework* or guidance* or guide or guides or guideline* or how or hows or methodolog* or model or models or paradigm* or practice* or principle* or process or recommend* or stratagem* or strateg* or tool or toolkit* or tools ) | #13 | 2 457 890 |
| Strategy | S12 or S13 | #14 | 2 459 433 |
| Total Result | S6 and S11 and S14 | #15 | 1 309 |

### Cinahl-EBSCO-(2024-02-05)

|  |  |  |
| --- | --- | --- |
| 1 | (MH "Diffusion of Innovation+") | 5,948 |
| 2 | TI ( ("scal* up" or "scal* out") ) OR AB ( ("scal* up" or "scal* out") ) OR SU ( ("scal* up" or "scal* out" ) ) | 5,948 |
| 3 | TI (("scaling" or widespread or spread# or spreading or "rolling out" or "roll out" or "rolls out" or "rolled out" or upscaling or scalability or scalable) N4 (innovation# or intervention# or technolog* or practice* or care or initiative* or program* or product# or therap* or service* or strateg* or change# or proces*)) OR AB (("scaling" or widespread or spread# or spreading or "rolling out" or "roll out" or "rolls out" or "rolled out" or upscaling or scalability or scalable) N4 (innovation# or intervention# or technolog* or practice* or care or initiative* or program* or product# or therap* or service* or strateg* or change# or proces*)) OR SU (("scaling" or widespread or spread# or spreading or "rolling out" or "roll out" or "rolls out" or "rolled out" or upscaling or scalability or scalable) N4 (innovation# or intervention# or technolog* or practice* or care or initiative* or program* or product# or therap* or service* or strateg* or change# or proces*) ) | 10,78 |
| 4 | TI ( (bring* or brought or taking or take* or increas* or going or implement* or econom*) N4 scal* N4 (innovation# or intervention# or technolog* or practice* or care or initiative* or program* or product# or therap* or service* or strateg* or change# or proces*) ) OR AB ( (bring* or brought or taking or take* or increas* or going or implement* or econom*) N4 scal* N4 (innovation# or intervention# or technolog* or practice* or care or initiative* or program* or product# or therap* or service* or strateg* or change# or proces*) ) OR SU ( (bring* or brought or taking or take* or increas* or going or implement* or econom*) N4 scal* N4 (innovation# or intervention# or technolog* or practice* or care or initiative* or program* or product# or therap* or service* or strateg* or change# or proces*) ) | 1,73 |
| 5 | (S2 OR S3 OR S4) | 16,758 |
| 6 | (S1 OR S5) | 36,68 |
| 7 | (MH "Consumer Participation") OR (MH "Stakeholder Participation") | 27,814 |
| 8 | TI ((caregiver* or care-giver* or citizen* or client* or communit* or consumer* or "family carer*" or lay or patient* or "peer helper*" or public or stakeholder* or survivor* or user*) N1 (collaborat* or cooperat* or co-operat* or coproduc* or co-produc* or engag* or includ* or involve* or "joint effort*" or mobilis* or mobiliz* or participat* or partner* or "work together" or "working together")) OR AB ((caregiver* or care-giver* or citizen* or client* or communit* or consumer* or "family carer*" or lay or patient* or "peer helper*" or public or stakeholder* or survivor* or user*) N1 (collaborat* or cooperat* or co-operat* or coproduc* or co-produc* or engag* or includ* or involve* or "joint effort*" or mobilis* or mobiliz* or participat* or partner* or "work together" or "working together")) OR SU ((caregiver* or care-giver* or citizen* or client* or communit* or consumer* or "family carer*" or lay or patient* or "peer helper*" or public or stakeholder* or survivor* or user*) N1 (collaborat* or cooperat* or co-operat* or coproduc* or co-produc* or engag* or includ* or involve* or "joint effort*" or mobilis* or mobiliz* or participat* or partner* or "work together" or "working together") ) | 215,549 |
| 9 | TI ( Co-build* or co-constr* or co-creation or coproduc* or co-produc* ) OR AB ( Co-build* or co-constr* or co-creation or coproduc* or co-produc* ) OR SU ( Co-build* or co-constr* or co-creation or coproduc* or co-produc* ) | 3,079 |
| 10 | (S8 OR S9) | 217,661 |
| 11 | (S7 OR S10) | 217,661 |
| 12 | (MH "Conceptual Framework") OR (MH "Models, Theoretical+") OR (MH "Practice Guidelines") OR (MH "Research Methodology") OR (MH "Study Methods") | 355,19 |
| 13 | TI ( Action or actions or approach* or example*or "frame work*" or Framework* or guidance* or guide or guides or guideline* or how or hows or methodolog* or model or models or paradigm* or practice* or principle* or process or recommend* or stratagem* or strateg* or tool or toolkit* or tools ) OR AB ( Action or actions or approach* or example* or "frame work*" or Framework* or guidance* or guide or guides or guideline* or how or hows or methodolog* or model or models or paradigm* or practice* or principle* or process or recommend* or stratagem* or strateg* or tool or toolkit* or tools ) OR SU ( Action or actions or approach* or example* or "frame work*" or Framework* or guidance* or guide or guides or guideline* or how or hows or methodolog* or model or models or paradigm* or practice* or principle* or process or recommend* or stratagem* or strateg* or tool or toolkit* or tools ) | 3,038,576 |
| 14 | (S12 OR S13) | 3,040,402 |
| 15 | (S6 AND S11 AND S14) | 2,244 |
| 16 | EM 20200929-20240205 | 1,061,830 |
| 17 | (EM 20200929-20240205) AND (S15 AND S16) | 858 |
| FINAL | (EM 20200929-20240205) AND (S15 AND S16) | 858 |

### PsycINFO-Ovid- (2020-09-29)

| **Concepts** | **Search strategy keywords** | **Search** | **# Results** |
| --- | --- | --- | --- |
| Scaling (Controlled Vocabulary) | Not available |  |  |
| Scaling (Free text) | ("scal* up" or "scal* out").ti,ab,hw,id,jx. | #1 | 2 316 |
|  | (("scaling" or widespread or spread? or spreading or "rolling out" or "roll out" or "rolls out" or "rolled out" or upscaling or scalability or scalable) adj5 (innovation? or intervention? or technolog* or practice* or care or initiative* or program* or product? or therap* or service* or strateg* or change? or proces*)).ti,ab,hw,id,jx. | #2 | 7 270 |
|  | ((bring* or brought or taking or take* or increas* or going or implement* or econom*) adj5 scal* adj5 (innovation? or intervention? or technolog* or practice* or care or initiative* or program* or product? or therap* or service* or strateg* or change? or proces*)).ti,ab,hw,id,jx. | #3 | 1 125 |
| Scaling | #1 or #2 or #3 | #4 | 10 038 |

| Patient partner (Controlled vocabulary) | exp client participation/ or exp community involvement/ | #5 | 7 680 |
| --- | --- | --- | --- |
| Patient partner in research (Free text) | ((caregiver* or care-giver* or citizen* or client* or communit* or consumer* or "family carer*" or lay or patient* or "peer helper*" or public or stakeholder* or survivor* or user*) adj2 (collaborat* or cooperat* or co-operat* or coproduc* or co-produc* or engag* or includ* or involve* or "joint effort*" or mobilis* or mobiliz* or participat* or partner* or "work together" or "working together")).ti,ab,hw,id,jx. | #6 | 81 467 |
| Research co-building (Free text) | (Co-build* or co-constr* or co-creation or coproduc* or co-produc*).ti,ab,hw,id,jx. | #7 | 5 007 |
| Patient partner (Free text) | #6 or #7 | #8 | 85 889 |
| Patient partner | #5 or #8 | #9 | 86 326 |
| Strategy (Controlled vocabulary) | methodology/ or exp practice/ | #10 | 47 051 |
| Strategy (Free text) | (Action or actions or approach* or example* or "frame work*" or Framework* or guidance* or guide or guides or guideline* or how or hows or methodolog* or model or models or paradigm* or practice* or principle* or process or recommend* or stratagem* or strateg* or tool or toolkit* or tools).ti,ab,hw,id,jx. | #11 | 2 662 123 |
| Strategy | #10 or #11 | #12 | 2 662 123 |
| Total Result | #4 and #9 and #12 | #13 | 471 |

### PsycINFO-Ovid- (2024-02-05)

| 1 | ("scal* up" or "scal* out").ti,ab,hw,id,jx. | 3194 |
| --- | --- | --- |
| 2 | (("scaling" or widespread or spread? or spreading or "rolling out" or "roll out" or "rolls out" or "rolled out" or upscaling or scalability or scalable) adj5 (innovation? or intervention? or technolog* or practice* or care or initiative* or program* or product? or therap* or service* or strateg* or change? or proces*)).ti,ab,hw,id,jx. | 9471 |
| 3 | ((bring* or brought or taking or take* or increas* or going or implement* or econom*) adj5 scal* adj5 (innovation? or intervention? or technolog* or practice* or care or initiative* or program* or product? or therap* or service* or strateg* or change? or proces*)).ti,ab,hw,id,jx. | 1489 |
| 4 | 1 or 2 or 3 | 13165 |
| 5 | exp client participation/ or exp community involvement/ | 10658 |
| 6 | ((caregiver* or care-giver* or citizen* or client* or communit* or consumer* or "family carer*" or lay or patient* or "peer helper*" or public or stakeholder* or survivor* or user*) adj2 (collaborat* or cooperat* or co-operat* or coproduc* or co-produc* or engag* or includ* or involve* or "joint effort*" or mobilis* or mobiliz* or participat* or partner* or "work together" or "working together")).ti,ab,hw,id,jx. | 104211 |
| 7 | (Co-build* or co-constr* or co-creation or coproduc* or co-produc*).ti,ab,hw,id,jx. | 7245 |
| 8 | 6 or 7 | 110372 |
| 9 | 5 or 8 | 110855 |
| 10 | methodology/ or exp practice/ | 52360 |
| 11 | (Action or actions or approach* or example* or "frame work*" or Framework* or guidance* or guide or guides or guideline* or how or hows or methodolog* or model or models or paradigm* or practice* or principle* or process or recommend* or stratagem* or strateg* or tool or toolkit* or tools).ti,ab,hw,id,jx. | 3132006 |
| 12 | 10 or 11 | 3132006 |
| 13 | 4 and 9 and 12 | 688 |
| 14 | limit 13 to up=20200929-20240205 | 219 |
| Final | limit 13 to up=20200929-20240205 | 219 |

### ERIC-EBSCO-(2020-09-29)

| **Concepts** | **Search strategy keywords** | **Search** | **# Results** |
| --- | --- | --- | --- |
| Scaling (Controlled Vocabulary) | Not available | - | - |
| Scaling (Free text) | TI ( ("scal* up" or "scal* out") )  OR AB ( ("scal* up" or "scal* out") )  OR SU ( ("scal* up" or "scal* out") ) | #1 | 947 |
|  | TI ( ("scaling" or widespread or spread# or spreading or "rolling out" or "roll out" or "rolls out" or "rolled out" or upscaling or scalability or scalable) N4 (innovation# or intervention# or technolog* or practice* or care or initiative* or program* or product# or therap* or service* or strateg* or change# or proces*) )  OR AB ( ("scaling" or widespread or spread# or spreading or "rolling out" or "roll out" or "rolls out" or "rolled out" or upscaling or scalability or scalable) N4 (innovation# or intervention# or technolog* or practice* or care or initiative* or program* or product# or therap* or service* or strateg* or change# or proces*) )  OR SU ( ("scaling" or widespread or spread# or spreading or "rolling out" or "roll out" or "rolls out" or "rolled out" or upscaling or scalability or scalable) N4 (innovation# or intervention# or technolog* or practice* or care or initiative* or program* or product# or therap* or service* or strateg* or change# or proces*) ) | #2 | 2 338 |
|  | TI ( (bring* or brought or taking or take* or increas* or going or implement* or econom*) N4 scal* N4 (innovation# or intervention# or technolog* or practice* or care or initiative* or program* or product# or therap* or service* or strateg* or change# or proces*) )  OR AB ( (bring* or brought or taking or take* or increas* or going or implement* or econom*) N4 scal* N4 (innovation# or intervention# or technolog* or practice* or care or initiative* or program* or product# or therap* or service* or strateg* or change# or proces*) )  OR SU ( (bring* or brought or taking or take* or increas* or going or implement* or econom*) N4 scal* N4 (innovation# or intervention# or technolog* or practice* or care or initiative* or program* or product# or therap* or service* or strateg* or change# or proces*) ) | #3 | 559 |
| Scaling (Free text) | S1 OR S2 OR S3 | #4 | 3 544 |

| Patient partner (Controlled vocabulary) | DE "Citizen Participation" OR DE "Community Cooperation" OR DE "Community Involvement" | #5 | 16 395 |
| --- | --- | --- | --- |
| Patient partner in research (Free text) | TI ( (caregiver* or care-giver* or citizen* or client* or communit* or consumer* or "family carer*" or lay or patient* or "peer helper*" or public or stakeholder* or survivor* or user*) N1 (collaborat* or cooperat* or co-operat* or coproduc* or co-produc* or engag* or includ* or involve* or "joint effort*" or mobilis* or mobiliz* or participat* or partner* or "work together" or "working together") )  OR AB ( (caregiver* or care-giver* or citizen* or client* or communit* or consumer* or "family carer*" or lay or patient* or "peer helper*" or public or stakeholder* or survivor* or user*) N1 (collaborat* or cooperat* or co-operat* or coproduc* or co-produc* or engag* or includ* or involve* or "joint effort*" or mobilis* or mobiliz* or participat* or partner* or "work together" or "working together") )  OR SU ( (caregiver* or care-giver* or citizen* or client* or communit* or consumer* or "family carer*" or lay or patient* or "peer helper*" or public or stakeholder* or survivor* or user*) N1 (collaborat* or cooperat* or co-operat* or coproduc* or co-produc* or engag* or includ* or involve* or "joint effort*" or mobilis* or mobiliz* or participat* or partner* or "work together" or "working together") ) | #6 | 41 457 |
| Research co-building (Free text) | TI ( Co-build* or co-constr* or co-creation or coproduc* or co-produc* )  OR AB ( Co-build* or co-constr* or co-creation or coproduc* or co-produc* )  OR SU ( Co-build* or co-constr* or co-creation or coproduc* or co-produc* ) | #7 | 1 895 |
| Patient partner (Free text) | S6 OR S7 | #8 | 43 215 |
| Patient partner | S5 OR S8 | #9 | 43 215 |
| Strategy (Controlled vocabulary) | DE "Best Practices" OR DE "Change Strategies" OR DE "Guidelines" OR DE "Methods" OR DE "Models" OR DE "Research Methodology" OR DE "Scientific Methodology" | #10 | 166 624 |
| Strategy (Free text) | TI ( Action or actions or approach* or "frame work*" or Framework* or guidance* or guide or guides or guideline* or how or hows or methodolog* or model or models or practice* or principle* or process or recommend* or stratagem* or strateg* or tool or toolkit* or tools )  OR AB ( Action or actions or approach* or "frame work*" or Framework* or guidance* or guide or guides or guideline* or how or hows or methodolog* or model or models or practice* or principle* or process or recommend* or stratagem* or strateg* or tool or toolkit* or tools )  OR SU ( Action or actions or approach* or "frame work*" or Framework* or guidance* or guide or guides or guideline* or how or hows or methodolog* or model or models or practice* or principle* or process or recommend* or stratagem* or strateg* or tool or toolkit* or tools ) | #11 | 1 148 522 |
| Strategy | S10 or S11 | #12 | 1 149 396 |
|  | DE "Access to Health Care" OR DE "Allied Health Personnel" OR DE "Anesthesiology" OR DE "Audiology" OR DE "Biomedicine" OR DE "Child Health" OR DE "Clinical Diagnosis" OR DE "Community Health Services" OR DE "Dentistry" OR DE "Dietetics" OR DE "Disease Control" OR DE "Disease Incidence" OR DE "Drug Use" OR DE "Epidemiology" OR DE "Family Practice (Medicine)" OR DE "Geriatrics" OR DE "Gynecology" OR DE "Health" OR DE "Health Care Costs" OR DE "Health Personnel" OR DE "Health Facilities" OR DE "Health Insurance" OR DE "Health Sciences" OR DE "Health Services" OR DE "Hospices (Terminal Care)" OR DE "Internal Medicine" OR DE "Medical Services" OR DE "Medical Care Evaluation" OR DE "Medicine" OR DE "Mental Health" OR DE "Mental Health Workers" OR DE "Neurology" OR DE "Nurses" OR DE "Nursing" OR DE "Nursing Homes" OR DE "Nutrition" OR DE "Obstetrics" OR DE "Occupational Safety and Health" OR DE "Oncology" OR DE "Ophthalmology" OR DE "Optometry" OR DE "Pathology" OR DE "Patient Education" OR DE "Pediatrics" OR DE "Pharmacology" OR DE "Pharmacy" OR DE "Physical Health" OR DE "Physicians" OR DE "Physician Patient Relationship" OR DE "Podiatry" OR DE "Pregnancy" OR DE "Prenatal Care" OR DE "Preventive Medicine" OR DE "Primary Health Care" OR DE "Psychiatry" OR DE "Psychologists" OR DE "Public Health" OR DE "School Health Services" OR DE "Sports Medicine" OR DE "Surgery" OR DE "Toxicology" OR DE "Trauma" OR DE "Wellness" OR DE "Disabilities" OR DE "Adventitious Impairments" OR DE "Attention Deficit Disorders" OR DE "Behavior Disorders" OR DE "Communication Disorders" OR DE "Congenital Impairments" OR DE "Developmental Disabilities" OR DE "Diseases" OR DE "Hearing Impairments" OR DE "Injuries" OR DE "Intellectual Disability" OR DE "Language Impairments" OR DE "Learning Disabilities" OR DE "Mental Disorders" OR DE "Mild Disabilities" OR DE "Multiple Disabilities" OR DE "Perceptual Impairments" OR DE "Physical Disabilities" OR DE "Severe Disabilities" OR DE "Special Health Problems" OR DE "Speech Impairments" OR DE "Visual Impairments" OR DE "Diseases" OR DE "Alcoholism" OR DE "Allergy" OR DE "Alzheimers Disease" OR DE "Cancer" OR DE "Chronic Illness" OR DE "Communicable Diseases" OR DE "Diabetes" OR DE "Drug Addiction" OR DE "Eating Disorders" OR DE "Fetal Alcohol Syndrome" OR DE "Genetic Disorders" OR DE "Hypertension" OR DE "Obesity" OR DE "Occupational Diseases" OR DE "Poisoning" OR DE "Seizures" OR DE "Terminal Illness" OR DE "Therapy" OR DE "Art Therapy" OR DE "Bibliotherapy" OR DE "Drug Therapy" OR DE "Educational Therapy" OR DE "Group Therapy" OR DE "Hearing Therapy" OR DE "Music Therapy" OR DE "Occupational Therapy" OR DE "Physical Therapy" OR DE "Psychotherapy" OR DE "Speech Therapy" OR DE "Therapeutic Recreation" OR DE "Medical Education" OR DE "Graduate Medical Education" OR DE "Nursing Education" OR DE "Pharmaceutical Education" OR DE "Veterinary Medical Education" OR DE "Psychology" OR DE "Behaviorism" OR DE "Child Psychology" OR DE "Clinical Psychology" OR DE "Cognitive Psychology" OR DE "Counseling Psychology" OR DE "Developmental Psychology" OR DE "Educational Psychology" OR DE "Experimental Psychology" OR DE "Individual Psychology" OR DE "Industrial Psychology" OR DE "Neuropsychology" OR DE "Psychometrics" OR DE "Psychopathology" OR DE "Psychophysiology" OR DE "School Psychology" OR DE "Social Psychology" OR DE "Sport Psychology" | #13 | 240 722 |
|  | TI ( care or chiroprati* or clinic* or communit* or dentist* or diagnos* or disease* or drug* or healing* or health* or illness or infection* or injur* or medicin* or medica* or nurs* or nutrition* or optometr* or orthoptic* or patholog* or patient or pharma* or pharmaceutic* or pill* or placebo* or poverty or pregnan* or prevention* or psychiatr* or psycholog* or psychosocial* or remed* or social* or surger* or therap* or treatment* or unemploy* or violence* or wound* )  OR AB ( care or chiroprati* or clinic* or communit* or dentist* or diagnos* or disease* or drug* or healing* or health* or illness or infection* or injur* or medicin* or medica* or nurs* or nutrition* or optometr* or orthoptic* or patholog* or patient or pharma* or pharmaceutic* or pill* or placebo* or poverty or pregnan* or prevention* or psychiatr* or psycholog* or psychosocial* or remed* or social* or surger* or therap* or treatment* or unemploy* or violence* or wound* )  OR SU ( care or chiroprati* or clinic* or communit* or dentist* or diagnos* or disease* or drug* or healing* or health* or illness or infection* or injur* or medicin* or medica* or nurs* or nutrition* or optometr* or orthoptic* or patholog* or patient or pharma* or pharmaceutic* or pill* or placebo* or poverty or pregnan* or prevention* or psychiatr* or psycholog* or psychosocial* or remed* or social* or surger* or therap* or treatment* or unemploy* or violence* or wound* ) | #14 | 771 882 |
|  | S13 OR S14 | #15 | 824 483 |
| Total Result | S4 and S9 and S12 and S15 | #16 | 127 |

### ERIC-EBSCO-(2024-02-05)

| 1 | TI ( ("scal* up" or "scal* out") ) OR AB ( ("scal* up" or "scal* out") ) OR SU ( ("scal* up" or "scal* out") ) | 1,259 |
| --- | --- | --- |
| 2 | TI ( ("scaling" or widespread or spread# or spreading or "rolling out" or "roll out" or "rolls out" or "rolled out" or upscaling or scalability or scalable) N4 (innovation# or intervention# or technolog* or practice* or care or initiative* or program* or product# or therap* or service* or strateg* or change# or proces*) ) OR AB ( ("scaling" or widespread or spread# or spreading or "rolling out" or "roll out" or "rolls out" or "rolled out" or upscaling or scalability or scalable) N4 (innovation# or intervention# or technolog* or practice* or care or initiative* or program* or product# or therap* or service* or strateg* or change# or proces*) ) OR SU ( ("scaling" or widespread or spread# or spreading or "rolling out" or "roll out" or "rolls out" or "rolled out" or upscaling or scalability or scalable) N4 (innovation# or intervention# or technolog* or practice* or care or initiative* or program* or product# or therap* or service* or strateg* or change# or proces*) ) | 2,969 |
| 3 | TI ( (bring* or brought or taking or take* or increas* or going or implement* or econom*) N4 scal* N4 (innovation# or intervention# or technolog* or practice* or care or initiative* or program* or product# or therap* or service* or strateg* or change# or proces*) ) OR AB ( (bring* or brought or taking or take* or increas* or going or implement* or econom*) N4 scal* N4 (innovation# or intervention# or technolog* or practice* or care or initiative* or program* or product# or therap* or service* or strateg* or change# or proces*) ) OR SU ( (bring* or brought or taking or take* or increas* or going or implement* or econom*) N4 scal* N4 (innovation# or intervention# or technolog* or practice* or care or initiative* or program* or product# or therap* or service* or strateg* or change# or proces*) ) | 702 |
| 4 | (S1 OR S2 OR S3) | 4,505 |
| 5 | DE "Citizen Participation" OR DE "Community Cooperation" OR DE "Community Involvement" | 17,921 |
| 6 | TI ( (caregiver* or care-giver* or citizen* or client* or communit* or consumer* or "family carer*" or lay or patient* or "peer helper*" or public or stakeholder* or survivor* or user*) N1 (collaborat* or cooperat* or co-operat* or coproduc* or co-produc* or engag* or includ* or involve* or "joint effort*" or mobilis* or mobiliz* or participat* or partner* or "work together" or "working together") ) OR AB ( (caregiver* or care-giver* or citizen* or client* or communit* or consumer* or "family carer*" or lay or patient* or "peer helper*" or public or stakeholder* or survivor* or user*) N1 (collaborat* or cooperat* or co-operat* or coproduc* or co-produc* or engag* or includ* or involve* or "joint effort*" or mobilis* or mobiliz* or participat* or partner* or "work together" or "working together") ) OR SU ( (caregiver* or care-giver* or citizen* or client* or communit* or consumer* or "family carer*" or lay or patient* or "peer helper*" or public or stakeholder* or survivor* or user*) N1 (collaborat* or cooperat* or co-operat* or coproduc* or co-produc* or engag* or includ* or involve* or "joint effort*" or mobilis* or mobiliz* or participat* or partner* or "work together" or "working together") ) | 46,611 |
| 7 | TI ( Co-build* or co-constr* or co-creation or coproduc* or co-produc* ) OR AB ( Co-build* or co-constr* or co-creation or coproduc* or co-produc* ) OR SU ( Co-build* or co-constr* or co-creation or coproduc* or co-produc* ) | 2,852 |
| 8 | (S6 OR S7) | 49,225 |
| 9 | (S5 OR S8) | 49,225 |
| 10 | DE "Best Practices" OR DE "Change Strategies" OR DE "Guidelines" OR DE "Methods" OR DE "Models" OR DE "Research Methodology" OR DE "Scientific Methodology" | 177,829 |
| 11 | TI ( Action or actions or approach* or "frame work*" or Framework* or guidance* or guide or guides or guideline* or how or hows or methodolog* or model or models or practice* or principle* or process or recommend* or stratagem* or strateg* or tool or toolkit* or tools ) OR AB ( Action or actions or approach* or "frame work*" or Framework* or guidance* or guide or guides or guideline* or how or hows or methodolog* or model or models or practice* or principle* or process or recommend* or stratagem* or strateg* or tool or toolkit* or tools ) OR SU ( Action or actions or approach* or "frame work*" or Framework* or guidance* or guide or guides or guideline* or how or hows or methodolog* or model or models or practice* or principle* or process or recommend* or stratagem* or strateg* or tool or toolkit* or tools ) | 1,280,860 |
| 12 | (S10 or S11) | 1,281,770 |
| 13 | DE "Access to Health Care" OR DE "Allied Health Personnel" OR DE "Anesthesiology" OR DE "Audiology" OR DE "Biomedicine" OR DE "Child Health" OR DE "Clinical Diagnosis" OR DE "Community Health Services" OR DE "Dentistry" OR DE "Dietetics" OR DE "Disease Control" OR DE "Disease Incidence" OR DE "Drug Use" OR DE "Epidemiology" OR DE "Family Practice (Medicine)" OR DE "Geriatrics" OR DE "Gynecology" OR DE "Health" OR DE "Health Care Costs" OR DE "Health Personnel" OR DE "Health Facilities" OR DE "Health Insurance" OR DE "Health Sciences" OR DE "Health Services" OR DE "Hospices (Terminal Care)" OR DE "Internal Medicine" OR DE "Medical Services" OR DE "Medical Care Evaluation" OR DE "Medicine" OR DE "Mental Health" OR DE "Mental Health Workers" OR DE "Neurology" OR DE "Nurses" OR DE "Nursing" OR DE "Nursing Homes" OR DE "Nutrition" OR DE "Obstetrics" OR DE "Occupational Safety and Health" OR DE "Oncology" OR DE "Ophthalmology" OR DE "Optometry" OR DE "Pathology" OR DE "Patient Education" OR DE "Pediatrics" OR DE "Pharmacology" OR DE "Pharmacy" OR DE "Physical Health" OR DE "Physicians" OR DE "Physician Patient Relationship" OR DE "Podiatry" OR DE "Pregnancy" OR DE "Prenatal Care" OR DE "Preventive Medicine" OR DE "Primary Health Care" OR DE "Psychiatry" OR DE "Psychologists" OR DE "Public Health" OR DE "School Health Services" OR DE "Sports Medicine" OR DE "Surgery" OR DE "Toxicology" OR DE "Trauma" OR DE "Wellness" OR DE "Disabilities" OR DE "Adventitious Impairments" OR DE "Attention Deficit Disorders" OR DE "Behavior Disorders" OR DE "Communication Disorders" OR DE "Congenital Impairments" OR DE "Developmental Disabilities" OR DE "Diseases" OR DE "Hearing Impairments" OR DE "Injuries" OR DE "Intellectual Disability" OR DE "Language Impairments" OR DE "Learning Disabilities" OR DE "Mental Disorders" OR DE "Mild Disabilities" OR DE "Multiple Disabilities" OR DE "Perceptual Impairments" OR DE "Physical Disabilities" OR DE "Severe Disabilities" OR DE "Special Health Problems" OR DE "Speech Impairments" OR DE "Visual Impairments" OR DE "Diseases" OR DE "Alcoholism" OR DE "Allergy" OR DE "Alzheimers Disease" OR DE "Cancer" OR DE "Chronic Illness" OR DE "Communicable Diseases" OR DE "Diabetes" OR DE "Drug Addiction" OR DE "Eating Disorders" OR DE "Fetal Alcohol Syndrome" OR DE "Genetic Disorders" OR DE "Hypertension" OR DE "Obesity" OR DE "Occupational Diseases" OR DE "Poisoning" OR DE "Seizures" OR DE "Terminal Illness" OR DE "Therapy" OR DE "Art Therapy" OR DE "Bibliotherapy" OR DE "Drug Therapy" OR DE "Educational Therapy" OR DE "Group Therapy" OR DE "Hearing Therapy" OR DE "Music Therapy" OR DE "Occupational Therapy" OR DE "Physical Therapy" OR DE "Psychotherapy" OR DE "Speech Therapy" OR DE "Therapeutic Recreation" OR DE "Medical Education" OR DE "Graduate Medical Education" OR DE "Nursing Education" OR DE "Pharmaceutical Education" OR DE "Veterinary Medical Education" OR DE "Psychology" OR DE "Behaviorism" OR DE "Child Psychology" OR DE "Clinical Psychology" OR DE "Cognitive Psychology" OR DE "Counseling Psychology" OR DE "Developmental Psychology" OR DE "Educational Psychology" OR DE "Experimental Psychology" OR DE "Individual Psychology" OR DE "Industrial Psychology" OR DE "Neuropsychology" OR DE "Psychometrics" OR DE "Psychopathology" OR DE "Psychophysiology" OR DE "School Psychology" OR DE "Social Psychology" OR DE "Sport Psychology" | 263,36 |
| 14 | TI ( care or chiroprati* or clinic* or communit* or dentist* or diagnos* or disease* or drug* or healing* or health* or illness or infection* or injur* or medicin* or medica* or nurs* or nutrition* or optometr* or orthoptic* or patholog* or patient or pharma* or pharmaceutic* or pill* or placebo* or poverty or pregnan* or prevention* or psychiatr* or psycholog* or psychosocial* or remed* or social* or surger* or therap* or treatment* or unemploy* or violence* or wound* ) OR AB ( care or chiroprati* or clinic* or communit* or dentist* or diagnos* or disease* or drug* or healing* or health* or illness or infection* or injur* or medicin* or medica* or nurs* or nutrition* or optometr* or orthoptic* or patholog* or patient or pharma* or pharmaceutic* or pill* or placebo* or poverty or pregnan* or prevention* or psychiatr* or psycholog* or psychosocial* or remed* or social* or surger* or therap* or treatment* or unemploy* or violence* or wound* ) OR SU ( care or chiroprati* or clinic* or communit* or dentist* or diagnos* or disease* or drug* or healing* or health* or illness or infection* or injur* or medicin* or medica* or nurs* or nutrition* or optometr* or orthoptic* or patholog* or patient or pharma* or pharmaceutic* or pill* or placebo* or poverty or pregnan* or prevention* or psychiatr* or psycholog* or psychosocial* or remed* or social* or surger* or therap* or treatment* or unemploy* or violence* or wound* ) | 863,106 |
| 15 | S13 OR S14 | 918,883 |
| 16 | S4 and S9 and S12 and S15 | 171 |
| 17 | DT 20200929-20240205 | 138,166 |
| 18 | (S16 AND S17) | 28 |
| FINAL | (S16 AND S17) | 28 |

### Cochrane Library- (2020-09-29)

| **Concepts** | **Search strategy keywords** | **Search** | **# Results** |
| --- | --- | --- | --- |
| Scaling (Controlled Vocabulary) | MeSH descriptor: [Diffusion of Innovation] explode all trees | #1 | 172 |
|  | MeSH descriptor: [Organizational Innovation] this term only | #2 | 105 |
|  | #1 or #2 | #3 | 270 |
| Scaling (Free text) | ("scale up" or "scaling up" or "scale out" or "scaling out"):ti,ab,kw | #4 | 1 054 |
|  | (("scaling" or widespread or spread or spreads or spreading or "rolling out" or "roll out" or "rolls out" or "rolled out" or upscaling or scalability or scalable) NEAR/4 (innovation or innovations or intervention or interventions or technolog* or practice* or care or initiative* or program* or product* or therap* or service* or strateg* or change or changes or proces*)):ti,ab,kw | #5 | 2 754 |
|  | ((bring* or brought or taking or take* or increas* or going or implement* or econom*) NEAR/4 scal* NEAR/4 (innovation or innovations or intervention or interventions or technolog* or practice* or care or initiative* or program* or product or products or therap* or service* or strateg* or change or changes or proces*)):ti,ab,kw | #6 | 365 |
| Scaling (Free text) | #4 or #5 or #6 | #7 | 3 907 |
| Scaling | #3 or #7 | #8 | 4 170 |

| Patient partner (Controlled vocabulary) | MeSH descriptor: [Community-Based Participatory Research] explode all trees | #9 | 245 |
| --- | --- | --- | --- |
|  | MeSH descriptor: [Patient Participation] explode all trees | #10 | 1 344 |
|  | MeSH descriptor: [Community Participation] explode all trees | #11 | 1 621 |
|  | MeSH descriptor: [Stakeholder Participation] this term only | #12 | 13 |
|  | #9 or #10 or #11 or #12 | #13 | 1 860 |
| Patient partner in research (Free text) | ((caregiver* or care-giver* or citizen* or client* or communit* or consumer* or "family carer*" or lay or patient* or "peer helper*" or public or stakeholder* or survivor* or user*) NEAR/1 (collaborat* or cooperat* or co-operat* or coproduc* or co-produc* or engag* or includ* or involve* or "joint effort*" or mobilis* or mobiliz* or participat* or partner* or "work together" or "working together")):ti,ab,kw | #14 | 32 737 |
| Research co-building (Free text) | (Co-build* or co-constr* or co-creation or coproduc* or co-produc*):ti,ab,kw | #15 | 132 |
| Patient partner (Free text) | #14 or #15 | #16 | 32 853 |
| Patient partner | #13 or #16 | #17 | 32 853 |
| Strategy (Controlled vocabulary) | MeSH descriptor: [Methods] this term only | #18 | 1 077 |
|  | MeSH descriptor: [Models, Theoretical] this term only | #19 | 671 |
|  | MeSH descriptor: [Resource Guide] explode all trees | #20 | 0 |
|  | MeSH descriptor: [Study Guide] explode all trees | #21 | 0 |
|  | MeSH descriptor: [Guideline] explode all trees | #22 | 0 |
|  | #18 or #19 or #20 or #21 or #22 | #23 | 1 746 |
| Strategy (Free text) | (Action or actions or approach* or "frame work*" or Framework* or guidance* or guide or guides or guideline* or how or hows or methodolog* or model or models or practice* or principle* or process or recommend* or stratagem* or strateg* or tool or toolkit* or tools):ti,ab,kw | #24 | 473 350 |
| Strategy | #23 or #24 | #24 | 480 255 |
| Total Result | #8 and #17 and #25 | #25 | 244 |

### Cochrane Library- (2024-02-05)

|  |  |  |
| --- | --- | --- |
| #1 | MeSH descriptor: [Diffusion of Innovation] explode all trees | 355 |
| #2 | MeSH descriptor: [Organizational Innovation] this term only | 153 |
| #3 | #1 or #2 | 494 |
| #4 | ("scale up" or "scaling up" or "scale out" or "scaling out"):ti,ab,kw | 1779 |
| #5 | (("scaling" or widespread or spread or spreads or spreading or "rolling out" or "roll out" or "rolls out" or "rolled out" or upscaling or scalability or scalable) NEAR/4 (innovation or innovations or intervention or interventions or technolog* or practice* or care or initiative* or program* or product* or therap* or service* or strateg* or change or changes or proces*)):ti,ab,kw | 4344 |
| #6 | ((bring* or brought or taking or take* or increas* or going or implement* or econom*) NEAR/4 scal* NEAR/4 (innovation or innovations or intervention or interventions or technolog* or practice* or care or initiative* or program* or product or products or therap* or service* or strateg* or change or changes or proces*)):ti,ab,kw | 655 |
| #7 | #4 or #5 or #6 | 6249 |
| #8 | #3 or #7 | 6720 |
| #9 | MeSH descriptor: [Community-Based Participatory Research] explode all trees | 385 |
| #10 | MeSH descriptor: [Patient Participation] explode all trees | 2175 |
| #11 | MeSH descriptor: [Community Participation] explode all trees | 2607 |
| #12 | MeSH descriptor: [Stakeholder Participation] this term only | 72 |
| #13 | #9 or #10 or #11 or #12 | 3018 |
| #14 | ((caregiver* or care-giver* or citizen* or client* or communit* or consumer* or "family carer*" or lay or patient* or "peer helper*" or public or stakeholder* or survivor* or user*) NEAR/1 (collaborat* or cooperat* or co-operat* or coproduc* or co-produc* or engag* or includ* or involve* or "joint effort*" or mobilis* or mobiliz* or participat* or partner* or "work together" or "working together")):ti,ab,kw | 47102 |
| #15 | (Co-build* or co-constr* or co-creation or coproduc* or co-produc*):ti,ab,kw | 310 |
| #16 | #14 or #15 | 47349 |
| #17 | #13 or #16 | 47361 |
| #18 | MeSH descriptor: [Methods] this term only | 1217 |
| #19 | MeSH descriptor: [Models, Theoretical] this term only | 1145 |
| #20 | MeSH descriptor: [Resource Guide] this term only | 0 |
| #21 | MeSH descriptor: [Study Guide] explode all trees | 0 |
| #22 | MeSH descriptor: [Guideline] explode all trees | 0 |
| #23 | #18 or #19 or #20 or #21 or #22 | 2359 |
| #24 | (Action or actions or approach* or "frame work*" or Framework* or guidance* or guide or guides or guideline* or how or hows or methodolog* or model or models or practice* or principle* or process or recommend* or stratagem* or strateg* or tool or toolkit* or tools):ti,ab,kw | 645476 |
| #25 | #23 or #24 | 646465 |
| #26 | #8 and #17 and #25 | 450 |
| #27 | 20200929-20240205 | 197 |
| FINAL | #26 and #27 | 197 |

###

### Sociological Abstract-Proquest (2020-09-29)

| **Concepts** | **Search strategy keywords** | **Search** | **# Results** |
| --- | --- | --- | --- |
| Scaling (Controlled Vocabulary) | Not available | - | - |
| Scaling (Free text) | ti("scal* up" or "scal* out")  OR ab("scal* up" or "scal* out")  OR su("scal* up" or "scal* out" ) | #1 | 606 |
|  | ti(("scaling" or widespread or spread or spreads or spreading or "rolling out" or "roll out" or "rolls out" or "rolled out" or upscaling or scalability or scalable) AND (innovation or innovations or intervention or interventions or technolog* or practice* or care or initiative* or program* or product or products or therap* or service* or strateg* or change or changes or proces*) )  OR ab(("scaling" or widespread or spread or spreads or spreading or "rolling out" or "roll out" or "rolls out" or "rolled out" or upscaling or scalability or scalable) AND (innovation or innovations or intervention or interventions or technolog* or practice* or care or initiative* or program* or product or products or therap* or service* or strateg* or change or changes or proces*))  OR su(("scaling" or widespread or spread or spreads or spreading or "rolling out" or "roll out" or "rolls out" or "rolled out" or upscaling or scalability or scalable) AND (innovation or innovations or intervention or interventions or technolog* or practice* or care or initiative* or program* or product or products or therap* or service* or strateg* or change or changes or proces*) ) | #2 | 17 002 |
|  | ti((bring* or brought or taking or take* or increas* or going or implement* or econom*) AND scal* AND (innovation or innovations or intervention or interventions or technolog* or practice* or care or initiative* or program* or product or products or therap* or service* or strateg* or change or changes or proces*) )  OR ab((bring* or brought or taking or take* or increas* or going or implement* or econom*) AND scal* AND (innovation or innovations or intervention or interventions or technolog* or practice* or care or initiative* or program* or product or products or therap* or service* or strateg* or change or changes or proces*))  OR su((bring* or brought or taking or take* or increas* or going or implement* or econom*) AND scal* AND (innovation or innovations or intervention or interventions or technolog* or practice* or care or initiative* or program* or product or products or therap* or service* or strateg* or change or changes or proces*) ) | #3 | 13 522 |
| Scaling (Free text) | #1 or #2 or #3 | #4 | 29 711 |

| Patient partner (Controlled vocabulary) | MAINSUBJECT.EXACT("Citizen Participation") or MAINSUBJECT.EXACT("Community Involvement") | #5 | 8 732 |
| --- | --- | --- | --- |
| Patient partner in research (Free text) | ti((caregiver* or care-giver* or citizen* or client* or communit* or consumer* or "family carer*" or lay or patient* or "peer helper*" or public or stakeholder* or survivor* or user*) AND (collaborat* or cooperat* or co-operat* or coproduc* or co-produc* or engag* or includ* or involve* or "joint effort*" or mobilis* or mobiliz* or participat* or partner* or "work together" or "working together") )  OR ab((caregiver* or care-giver* or citizen* or client* or communit* or consumer* or "family carer*" or lay or patient* or "peer helper*" or public or stakeholder* or survivor* or user*) AND (collaborat* or cooperat* or co-operat* or coproduc* or co-produc* or engag* or includ* or involve* or "joint effort*" or mobilis* or mobiliz* or participat* or partner* or "work together" or "working together"))  OR su((caregiver* or care-giver* or citizen* or client* or communit* or consumer* or "family carer*" or lay or patient* or "peer helper*" or public or stakeholder* or survivor* or user*) AND (collaborat* or cooperat* or co-operat* or coproduc* or co-produc* or engag* or includ* or involve* or "joint effort*" or mobilis* or mobiliz* or participat* or partner* or "work together" or "working together") ) | #6 | 159 030 |
| Research co-building (Free text) | ti(Co-build* or co-constr* or co-creation or coproduc* or co-produc*)  OR ab(Co-build* or co-constr* or co-creation or coproduc* or co-produc*)  OR su(Co-build* or co-constr* or co-creation or coproduc* or co-produc* ) | #7 | 1 858 |
| Patient partner (Free text) | #6 or #7 | #8 | 160 107 |
| Patient partner | #5 or #8 | #9 | 160 107 |
| Strategy (Controlled vocabulary) | MAINSUBJECT.EXACT.EXPLODE("Action") OR MAINSUBJECT.EXACT("Alternative Approaches") OR MAINSUBJECT.EXACT("Research Methodology") OR MAINSUBJECT.EXACT("Models") OR MAINSUBJECT.EXACT("Paradigms") OR MAINSUBJECT.EXACT.EXPLODE("Principles") OR MAINSUBJECT.EXACT.EXPLODE("Tools") | #10 | 40 958 |
| Strategy (Free text) | ti(Action or actions or approach* or "frame work*" or Framework* or guidance* or guide or guides or guideline* or how or hows or methodolog* or model or models or practice* or principle* or process or recommend* or stratagem* or strateg* or tool or toolkit* or tools)  OR ab(Action or actions or approach* or "frame work*" or Framework* or guidance* or guide or guides or guideline* or how or hows or methodolog* or model or models or practice* or principle* or process or recommend* or stratagem* or strateg* or tool or toolkit* or tools)  OR su(Action or actions or approach* or "frame work*" or Framework* or guidance* or guide or guides or guideline* or how or hows or methodolog* or model or models or practice* or principle* or process or recommend* or stratagem* or strateg* or tool or toolkit* or tools) | #11 | 941 521 |
| Strategy | #10 or #11 | #12 | 942 488 |
|  | MAINSUBJECT.EXACT.EXPLODE("After Care") OR MAINSUBJECT.EXACT("Bioethics") OR MAINSUBJECT.EXACT("Clinics") OR MAINSUBJECT.EXACT("Community Mental Health Centers") OR MAINSUBJECT.EXACT("Dentists") OR MAINSUBJECT.EXACT.EXPLODE("Disorders") OR MAINSUBJECT.EXACT.EXPLODE("Drugs") OR MAINSUBJECT.EXACT.EXPLODE("Handicapped") OR MAINSUBJECT.EXACT.EXPLODE("Health") OR MAINSUBJECT.EXACT.EXPLODE("Health Behavior") OR MAINSUBJECT.EXACT("Health Care Services") OR MAINSUBJECT.EXACT("Health Care Services Policy") OR MAINSUBJECT.EXACT("Health Care Utilization") OR MAINSUBJECT.EXACT("Health Education") OR MAINSUBJECT.EXACT("Health Insurance") OR MAINSUBJECT.EXACT("Health Planning") OR MAINSUBJECT.EXACT("Health Policy") OR MAINSUBJECT.EXACT("Health Problems") OR MAINSUBJECT.EXACT.EXPLODE("Health Professions") OR MAINSUBJECT.EXACT("Health Research") OR MAINSUBJECT.EXACT.EXPLODE("Home Care") OR MAINSUBJECT.EXACT.EXPLODE("Illness") OR MAINSUBJECT.EXACT("Injuries") OR MAINSUBJECT.EXACT.EXPLODE("Medical Decision Making") OR MAINSUBJECT.EXACT("Medical Research") OR MAINSUBJECT.EXACT("Medical Sociology") OR MAINSUBJECT.EXACT.EXPLODE("Medical Technology") OR MAINSUBJECT.EXACT("Medications") OR MAINSUBJECT.EXACT.EXPLODE("Medicine") OR MAINSUBJECT.EXACT("Nurses") OR MAINSUBJECT.EXACT.EXPLODE("Nutrition") OR MAINSUBJECT.EXACT("Optometry") OR MAINSUBJECT.EXACT("Paramedical Personnel") OR MAINSUBJECT.EXACT.EXPLODE("Patients") OR MAINSUBJECT.EXACT("Pharmacists") OR MAINSUBJECT.EXACT("Placebo Effect") OR MAINSUBJECT.EXACT.EXPLODE("Poverty") OR MAINSUBJECT.EXACT("Practitioner Patient Relationship") OR MAINSUBJECT.EXACT.EXPLODE("Pregnancy") OR MAINSUBJECT.EXACT.EXPLODE("Prevention") OR MAINSUBJECT.EXACT.EXPLODE("Psychology") OR MAINSUBJECT.EXACT("Psychosocial Factors") OR MAINSUBJECT.EXACT("Quality of Health Care") OR MAINSUBJECT.EXACT.EXPLODE("Social Work") OR MAINSUBJECT.EXACT("Social Workers") OR MAINSUBJECT.EXACT.EXPLODE("Treatment") OR MAINSUBJECT.EXACT("Treatment Compliance") | #13 | 206 226 |
|  | ti(care or chiroprati* or clinic* or communit* or dentist* or diagnos* or disease* or drug* or healing* or health* or illness or infection* or injur* or medicin* or medica* or nurs* or nutrition* or optometr* or orthoptic* or patholog* or patient or pharma* or pharmaceutic* or pill* or placebo* or poverty or pregnan* or prevention* or psychiatr* or psycholog* or psychosocial* or remed* or social* or surger* or therap* or treatment* or unemploy* or violence* or wound* )  OR ab(care or chiroprati* or clinic* or communit* or dentist* or diagnos* or disease* or drug* or healing* or health* or illness or infection* or injur* or medicin* or medica* or nurs* or nutrition* or optometr* or orthoptic* or patholog* or patient or pharma* or pharmaceutic* or pill* or placebo* or poverty or pregnan* or prevention* or psychiatr* or psycholog* or psychosocial* or remed* or social* or surger* or therap* or treatment* or unemploy* or violence* or wound*)  OR su(care or chiroprati* or clinic* or communit* or dentist* or diagnos* or disease* or drug* or healing* or health* or illness or infection* or injur* or medicin* or medica* or nurs* or nutrition* or optometr* or orthoptic* or patholog* or patient or pharma* or pharmaceutic* or pill* or placebo* or poverty or pregnan* or prevention* or psychiatr* or psycholog* or psychosocial* or remed* or social* or surger* or therap* or treatment* or unemploy* or violence* or wound*) | #14 | 1 191 840 |
|  | #13 or #14 | #15 | 1 197 073 |
| Total Result | #4 and #9 and #12 and #15 | #16 | 4 633 |

### Sociological Abstract-Proquest (2024-02-05)

| 1 | ti("scal* up" or "scal* out") OR ab("scal* up" or "scal* out") OR su("scal* up" or "scal* out" ) | 1 409 |
| --- | --- | --- |
| 2 | (ti("scal* up" OR "scal* out") OR ab("scal* up" OR "scal* out") OR su("scal* up" OR "scal* out")) OR (ti(("scaling" or widespread or spread or spreads or spreading or "rolling out" or "roll out" or "rolls out" or "rolled out" or upscaling or scalability or scalable) AND (innovation or innovations or intervention or interventions or technolog* or practice* or care or initiative* or program* or product or products or therap* or service* or strateg* or change or changes or proces*) ) OR ab(("scaling" or widespread or spread or spreads or spreading or "rolling out" or "roll out" or "rolls out" or "rolled out" or upscaling or scalability or scalable) AND (innovation or innovations or intervention or interventions or technolog* or practice* or care or initiative* or program* or product or products or therap* or service* or strateg* or change or changes or proces*)) OR su(("scaling" or widespread or spread or spreads or spreading or "rolling out" or "roll out" or "rolls out" or "rolled out" or upscaling or scalability or scalable) AND (innovation or innovations or intervention or interventions or technolog* or practice* or care or initiative* or program* or product or products or therap* or service* or strateg* or change or changes or proces*) ) ) | 25 293 |
| 3 | (ti((bring* or brought or taking or take* or increas* or going or implement* or econom*) AND scal* AND (innovation or innovations or intervention or interventions or technolog* or practice* or care or initiative* or program* or product or products or therap* or service* or strateg* or change or changes or proces*) ) OR ab((bring* or brought or taking or take* or increas* or going or implement* or econom*) AND scal* AND (innovation or innovations or intervention or interventions or technolog* or practice* or care or initiative* or program* or product or products or therap* or service* or strateg* or change or changes or proces*)) OR su((bring* or brought or taking or take* or increas* or going or implement* or econom*) AND scal* AND (innovation or innovations or intervention or interventions or technolog* or practice* or care or initiative* or program* or product or products or therap* or service* or strateg* or change or changes or proces*) ) ) OR (ti(("scaling" OR widespread OR spread OR spreads OR spreading OR "rolling out" OR "roll out" OR "rolls out" OR "rolled out" OR upscaling OR scalability OR scalable) AND (innovation OR innovations OR intervention OR interventions OR technolog* OR practice* OR care OR initiative* OR program* OR product OR products OR therap* OR service* OR strateg* OR change OR changes OR proces*)) OR ab(("scaling" OR widespread OR spread OR spreads OR spreading OR "rolling out" OR "roll out" OR "rolls out" OR "rolled out" OR upscaling OR scalability OR scalable) AND (innovation OR innovations OR intervention OR interventions OR technolog* OR practice* OR care OR initiative* OR program* OR product OR products OR therap* OR service* OR strateg* OR change OR changes OR proces*)) OR su(("scaling" OR widespread OR spread OR spreads OR spreading OR "rolling out" OR "roll out" OR "rolls out" OR "rolled out" OR upscaling OR scalability OR scalable) AND (innovation OR innovations OR intervention OR interventions OR technolog* OR practice* OR care OR initiative* OR program* OR product OR products OR therap* OR service* OR strateg* OR change OR changes OR proces*))) | 44 037 |
| 4 | [S1] OR [S2] OR [S3] | 44 440 |
| 5 | MAINSUBJECT.EXACT("Citizen Participation") or MAINSUBJECT.EXACT("Community Involvement") | 14 974 |
| 6 | ti((caregiver* or care-giver* or citizen* or client* or communit* or consumer* or "family carer*" or lay or patient* or "peer helper*" or public or stakeholder* or survivor* or user*) AND (collaborat* or cooperat* or co-operat* or coproduc* or co-produc* or engag* or includ* or involve* or "joint effort*" or mobilis* or mobiliz* or participat* or partner* or "work together" or "working together") ) OR ab((caregiver* or care-giver* or citizen* or client* or communit* or consumer* or "family carer*" or lay or patient* or "peer helper*" or public or stakeholder* or survivor* or user*) AND (collaborat* or cooperat* or co-operat* or coproduc* or co-produc* or engag* or includ* or involve* or "joint effort*" or mobilis* or mobiliz* or participat* or partner* or "work together" or "working together")) OR su((caregiver* or care-giver* or citizen* or client* or communit* or consumer* or "family carer*" or lay or patient* or "peer helper*" or public or stakeholder* or survivor* or user*) AND (collaborat* or cooperat* or co-operat* or coproduc* or co-produc* or engag* or includ* or involve* or "joint effort*" or mobilis* or mobiliz* or participat* or partner* or "work together" or "working together") ) | 263 878 |
| 7 | ti(Co-build* or co-constr* or co-creation or coproduc* or co-produc*) OR ab(Co-build* or co-constr* or co-creation or coproduc* or co-produc*) OR su(Co-build* or co-constr* or co-creation or coproduc* or co-produc* ) | 3 449 |
| 8 | [S6] OR [S7] | 265 690 |
| 9 | [S5] OR [S9] | 265 690 |
| 10 | MAINSUBJECT.EXACT.EXPLODE("Action") OR MAINSUBJECT.EXACT("Alternative Approaches") OR MAINSUBJECT.EXACT("Research Methodology") OR MAINSUBJECT.EXACT("Models") OR MAINSUBJECT.EXACT("Paradigms") OR MAINSUBJECT.EXACT.EXPLODE("Principles") OR MAINSUBJECT.EXACT.EXPLODE("Tools") | 69 796 |
| 11 | ti(Action or actions or approach* or "frame work*" or Framework* or guidance* or guide or guides or guideline* or how or hows or methodolog* or model or models or practice* or principle* or process or recommend* or stratagem* or strateg* or tool or toolkit* or tools) OR ab(Action or actions or approach* or "frame work*" or Framework* or guidance* or guide or guides or guideline* or how or hows or methodolog* or model or models or practice* or principle* or process or recommend* or stratagem* or strateg* or tool or toolkit* or tools) OR su(Action or actions or approach* or "frame work*" or Framework* or guidance* or guide or guides or guideline* or how or hows or methodolog* or model or models or practice* or principle* or process or recommend* or stratagem* or strateg* or tool or toolkit* or tools) | 1 315 940 |
| 12 | [S11] OR [S12] | 1 320 172 |
| 13 | MAINSUBJECT.EXACT("Community mental health services") OR MAINSUBJECT.EXACT.EXPLODE("After care") OR MAINSUBJECT.EXACT("Bioethics") OR MAINSUBJECT.EXACT("Clinics") OR MAINSUBJECT.EXACT.EXPLODE("Disorders") OR MAINSUBJECT.EXACT.EXPLODE("Disabled people") OR MAINSUBJECT.EXACT.EXPLODE("Health behavior") OR MAINSUBJECT.EXACT("Health care policy") OR MAINSUBJECT.EXACT("Health education") OR MAINSUBJECT.EXACT("Health planning") or MAINSUBJECT.EXACT("Health care policy") OR MAINSUBJECT.EXACT("Health problems") OR MAINSUBJECT.EXACT.EXPLODE("Medical personnel") OR MAINSUBJECT.EXACT("Health research") OR MAINSUBJECT.EXACT.EXPLODE("Home health care") OR MAINSUBJECT.EXACT.EXPLODE("Illnesses") OR MAINSUBJECT.EXACT("Injuries") OR MAINSUBJECT.EXACT.EXPLODE("Medical decision making") OR MAINSUBJECT.EXACT("Medical research") OR MAINSUBJECT.EXACT("Medical sociology") OR MAINSUBJECT.EXACT.EXPLODE("Medical technology") OR MAINSUBJECT.EXACT.EXPLODE("Medicine") OR MAINSUBJECT.EXACT("Nurses") OR MAINSUBJECT.EXACT.EXPLODE("Nutrition") OR MAINSUBJECT.EXACT("Optometry") OR MAINSUBJECT.EXACT("Medical personnel") OR MAINSUBJECT.EXACT.EXPLODE("Patients") OR MAINSUBJECT.EXACT("Pharmacists") OR MAINSUBJECT.EXACT("Placebo effect") OR MAINSUBJECT.EXACT.EXPLODE("Poverty") OR MAINSUBJECT.EXACT.EXPLODE("Pregnancy") OR MAINSUBJECT.EXACT.EXPLODE("Prevention") OR MAINSUBJECT.EXACT.EXPLODE("Psychology") OR MAINSUBJECT.EXACT("Psychosocial factors") OR MAINSUBJECT.EXACT("Quality of care") OR MAINSUBJECT.EXACT.EXPLODE("Social work") OR MAINSUBJECT.EXACT("Social workers") OR MAINSUBJECT.EXACT.EXPLODE("Medical treatment") OR MAINSUBJECT.EXACT("Treatment compliance") | 374 710 |
| 14 | ti(care or chiroprati* or clinic* or communit* or dentist* or diagnos* or disease* or drug* or healing* or health* or illness or infection* or injur* or medicin* or medica* or nurs* or nutrition* or optometr* or orthoptic* or patholog* or patient or pharma* or pharmaceutic* or pill* or placebo* or poverty or pregnan* or prevention* or psychiatr* or psycholog* or psychosocial* or remed* or social* or surger* or therap* or treatment* or unemploy* or violence* or wound* ) OR ab(care or chiroprati* or clinic* or communit* or dentist* or diagnos* or disease* or drug* or healing* or health* or illness or infection* or injur* or medicin* or medica* or nurs* or nutrition* or optometr* or orthoptic* or patholog* or patient or pharma* or pharmaceutic* or pill* or placebo* or poverty or pregnan* or prevention* or psychiatr* or psycholog* or psychosocial* or remed* or social* or surger* or therap* or treatment* or unemploy* or violence* or wound*) OR su(care or chiroprati* or clinic* or communit* or dentist* or diagnos* or disease* or drug* or healing* or health* or illness or infection* or injur* or medicin* or medica* or nurs* or nutrition* or optometr* or orthoptic* or patholog* or patient or pharma* or pharmaceutic* or pill* or placebo* or poverty or pregnan* or prevention* or psychiatr* or psycholog* or psychosocial* or remed* or social* or surger* or therap* or treatment* or unemploy* or violence* or wound*) | 1 724 454 |
| 15 | [S13] OR [S14] | 1 733 027 |
| 16 | [4] and [9] and [12] and [15] | 8 034 |
| 17 | [16] and 20200929-202040205 | 1 482 |
| FINAL | [16] and [17] | 1 482 |

### Academic Search Premier-EBSCO-(2020-09-30)

| **Concepts** | **Search strategy keywords** | **Search** | **# Results** |
| --- | --- | --- | --- |
| Scaling (Controlled Vocabulary) | DE "DIFFUSION of innovations" OR DE "ECONOMIES of scale" OR DE "DIFFUSION of innovations theory" OR DE "INNOVATION adoption" OR DE "TECHNOLOGY transfer" | #1 | 11 871 |
| Scaling (Free text) | TI ( ("scal* up" or "scal* out") )  OR AB ( ("scal* up" or "scal* out") )  OR SU ( ("scal* up" or "scal* out") ) | #2 | 23 960 |
|  | TI (("scaling" or widespread or spread# or spreading or "rolling out" or "roll out" or "rolls out" or "rolled out" or upscaling or scalability or scalable) N4 (innovation# or intervention# or technolog* or practice* or care or initiative* or program* or product# or therap* or service* or strateg* or change# or proces*))  OR AB (("scaling" or widespread or spread# or spreading or "rolling out" or "roll out" or "rolls out" or "rolled out" or upscaling or scalability or scalable) N4 (innovation# or intervention# or technolog* or practice* or care or initiative* or program* or product# or therap* or service* or strateg* or change# or proces*))  OR SU (("scaling" or widespread or spread# or spreading or "rolling out" or "roll out" or "rolls out" or "rolled out" or upscaling or scalability or scalable) N4 (innovation# or intervention# or technolog* or practice* or care or initiative* or program* or product# or therap* or service* or strateg* or change# or proces*) ) | #3 | 36 853 |
|  | TI ( (bring* or brought or taking or take* or increas* or going or implement* or econom*) N4 scal* N4 (innovation# or intervention# or technolog* or practice* or care or initiative* or program* or product# or therap* or service* or strateg* or change# or proces*) )  OR AB ( (bring* or brought or taking or take* or increas* or going or implement* or econom*) N4 scal* N4 (innovation# or intervention# or technolog* or practice* or care or initiative* or program* or product# or therap* or service* or strateg* or change# or proces*) )  OR SU ( (bring* or brought or taking or take* or increas* or going or implement* or econom*) N4 scal* N4 (innovation# or intervention# or technolog* or practice* or care or initiative* or program* or product# or therap* or service* or strateg* or change# or proces*) ) | #4 | 4 577 |
| Scaling (Free text) | S2 OR S3 OR S4 | #5 | 62 223 |
| Scaling | S1 OR S5 | #6 | 73 718 |

| Patient partner (Controlled vocabulary) | DE "COMMUNITY-based participatory research" | #7 | 1 658 |
| --- | --- | --- | --- |
| Patient partner in research (Free text) | TI ((caregiver* or care-giver* or citizen* or client* or communit* or consumer* or "family carer*" or lay or patient* or "peer helper*" or public or stakeholder* or survivor* or user*) N1 (collaborat* or cooperat* or co-operat* or coproduc* or co-produc* or engag* or includ* or involve* or "joint effort*" or mobilis* or mobiliz* or participat* or partner* or "work together" or "working together"))  OR AB ((caregiver* or care-giver* or citizen* or client* or communit* or consumer* or "family carer*" or lay or patient* or "peer helper*" or public or stakeholder* or survivor* or user*) N1 (collaborat* or cooperat* or co-operat* or coproduc* or co-produc* or engag* or includ* or involve* or "joint effort*" or mobilis* or mobiliz* or participat* or partner* or "work together" or "working together"))  OR SU ( (caregiver* or care-giver* or citizen* or client* or communit* or consumer* or "family carer*" or lay or patient* or "peer helper*" or public or stakeholder* or survivor* or user*) N1 (collaborat* or cooperat* or co-operat* or coproduc* or co-produc* or engag* or includ* or involve* or "joint effort*" or mobilis* or mobiliz* or participat* or partner* or "work together" or "working together") ) | #8 | 269 727 |
| Research co-building (Free text) | TI ( Co-build* or co-constr* or co-creation or coproduc* or co-produc* )  OR AB ( Co-build* or co-constr* or co-creation or coproduc* or co-produc* )  OR SU ( Co-build* or co-constr* or co-creation or coproduc* or co-produc* ) | #9 | 13 107 |
| Patient partner (Free text) | S8 OR S9 | #10 | 281 896 |
| Patient partner | S7 OR S10 | #11 | 282 111 |
| Strategy (Controlled vocabulary) | DE "GUIDELINES" OR DE "METHODOLOGY" OR DE "TOOLS" | #12 | 56 570 |
| Strategy (Free text) | TI ( Action or actions or approach* or "frame work*" or Framework* or guidance* or guide or guides or guideline* or how or hows or methodolog* or model or models or practice* or principle* or process or recommend* or stratagem* or strateg* or tool or toolkit* or tools )  OR AB ( Action or actions or approach* or "frame work*" or Framework* or guidance* or guide or guides or guideline* or how or hows or methodolog* or model or models or practice* or principle* or process or recommend* or stratagem* or strateg* or tool or toolkit* or tools )  OR SU ( Action or actions or approach* or "frame work*" or Framework* or guidance* or guide or guides or guideline* or how or hows or methodolog* or model or models or practice* or principle* or process or recommend* or stratagem* or strateg* or tool or toolkit* or tools ) | #13 | 11 644 279 |
| Strategy | S12 or S13 | #14 | 11 645 966 |
|  | DE "CAREGIVERS" OR DE "CARE of aging parents" OR DE "CARE of Alzheimer's patients" OR DE "CARE of dementia patients" OR DE "CARE of Parkinson's disease patients" OR DE "CHILD caregivers" OR DE "CLINIC employees" OR DE "DEMENTIA care mapping" OR DE "HEALTH" OR DE "HEALTH facility employees" OR DE "LGBTQ+ caregivers" OR DE "LIBRARIES & caregivers" OR DE "LONG-distance caregivers" OR DE "MALE caregivers" OR DE "MEDICAL personnel-caregiver relationships" OR DE "MENTAL health facility employees" OR DE "NURSING home employees" OR DE "OLDER caregivers" OR DE "RURAL caregivers" OR DE "UTILIZATION of clinics" OR DE "WOMEN caregivers" OR DE "MEDICINE" OR DE "ADOLESCENT medicine" OR DE "ALTERNATIVE medicine" OR DE "ANCIENT medicine" OR DE "ANESTHESIOLOGY" OR DE "AUDIOLOGY" OR DE "AUTOMOTIVE medicine" OR DE "BIOMEDICAL engineering" OR DE "BLOOD as food or medicine" OR DE "BLOODLETTING" OR DE "BUDDHIST medicine" OR DE "BUSINESS & medicine" OR DE "CIRCUMPOLAR medicine" OR DE "CLINICAL medicine" OR DE "COMMUNISM & medicine" OR DE "COMPUTER vision in medicine" OR DE "CREATIVE ability in medicine" OR DE "CRITICAL care medicine" OR DE "CURATIVE medicine" OR DE "DARWINIAN medicine" OR DE "DEFENSIVE medicine" OR DE "DENTISTRY" OR DE "DERMATOLOGY" OR DE "DIAGNOSIS" OR DE "DISASTER medicine" OR DE "ELECTRICITY in medicine" OR DE "EMERGENCY medicine" OR DE "ENDOCRINOLOGY" OR DE "ENVIRONMENTAL medicine" OR DE "FAMILY medicine" OR DE "FUZZY systems in medicine" OR DE "GERIATRICS" OR DE "GYNECOLOGY" OR DE "HEALTH" OR DE "HETEROSEXISM in medicine" OR DE "HOMOPHOBIA in medicine" OR DE "HORMONE therapy" OR DE "INTEGRATIVE medicine" OR DE "INTERNAL medicine" OR DE "INTERNET in medicine" OR DE "LANGUAGE & medicine" OR DE "LITERATURE & medicine" OR DE "MEDICAL advertising" OR DE "MEDICAL climatology" OR DE "MEDICAL communication" OR DE "MEDICAL jurisprudence" OR DE "MEDICAL parasitology" OR DE "MEDICAL robotics" OR DE "MEDICINE & art" OR DE "MEDICINE & philosophy" OR DE "MEDICINE & theater" OR DE "MEDIEVAL medicine" OR DE "MENTORING in medicine" OR DE "MIDWIFERY" OR DE "MILITARY medicine" OR DE "MINORITIES in medicine" OR DE "MISSIONARY medicine" OR DE "MOTION pictures & medicine" OR DE "MOTION pictures in medicine" OR DE "NANOMEDICINE" OR DE "NATIONAL socialism & medicine" OR DE "NAVAL medicine" OR DE "NEUROLOGY" OR DE "NOSOLOGY" OR DE "NURSING" OR DE "OBSTETRICS" OR DE "OCCUPATIONAL medicine" OR DE "ONCOLOGY" OR DE "OPHTHALMOLOGY" OR DE "OPTICAL fibers in medicine" OR DE "ORAL medicine" OR DE "ORTHOPEDICS" OR DE "OSTEOPATHIC medicine" OR DE "OTOLARYNGOLOGY" OR DE "PAIN medicine" OR DE "PEDIATRICS" OR DE "PHARMACY" OR DE "PHYSICAL medicine" OR DE "PLASTICS in medicine" OR DE "PODIATRY" OR DE "PREVENTIVE medicine" OR DE "PSYCHIATRY" OR DE "QUACKS & quackery" OR DE "RACISM in medicine" OR DE "RADIO in medicine" OR DE "REGENERATIVE medicine" OR DE "SEX discrimination in medicine" OR DE "SEXISM in medicine" OR DE "SOUND recordings in medicine" OR DE "SPACE medicine" OR DE "SURGERY" OR DE "TELEMEDICINE" OR DE "TELEVISION in medicine" OR DE "TOXICOLOGY" OR DE "TROPICAL medicine" OR DE "UROLOGY" OR DE "VASCULAR medicine" OR DE "VETERINARY medicine" OR DE "VIDEO recording in medicine" OR DE "VIRTUAL reality in medicine" OR DE "WILDERNESS medicine" OR DE "WIT & humor in medicine" OR DE "WOMEN in medicine" OR DE "MEDICAL personnel" OR DE "ABORIGINAL Australians in medicine" OR DE "ALLIED health personnel" OR DE "AROMATHERAPISTS" OR DE "BIOMEDICAL engineers" OR DE "BLACK people in medicine" OR DE "CHIROPRACTORS" OR DE "DENTAL personnel" OR DE "EMERGENCY medical personnel" OR DE "HEALTH care teams" OR DE "HEALTH occupations students" OR DE "HEALTH practitioners" OR DE "HEALTH services administrators" OR DE "HOSPITAL personnel" OR DE "IMPAIRED medical personnel" OR DE "MEDICAL personnel as patients" OR DE "MEDICAL personnel-caregiver relationships" OR DE "MEDICAL registry personnel" OR DE "MEDICAL research personnel" OR DE "MEDICAL scribes" OR DE "MEDICAL specialties & specialists" OR DE "MEDICAL teaching personnel" OR DE "MENTAL health personnel" OR DE "MIDWIVES" OR DE "MILITARY medical personnel" OR DE "MINORITY medical personnel" OR DE "MULTISKILLED medical personnel" OR DE "NATIVE Americans in medicine" OR DE "NURSES" OR DE "OPERATING room personnel" OR DE "OPTOMETRISTS" OR DE "ORGAN transplant coordinators" OR DE "PATIENT-professional relations" OR DE "PHARMACISTS" OR DE "PHLEBOTOMISTS" OR DE "PHYSICIANS" OR DE "PODIATRISTS" OR DE "PROSTHETISTS" OR DE "PUBLIC health personnel" OR DE "RECOVERY room personnel" OR DE "STUDENT volunteers in medical care" OR DE "TRAVELING medical personnel" OR DE "UNLICENSED medical personnel" OR DE "DIAGNOSIS" OR DE "ALLERGY diagnosis" OR DE "CANCER diagnosis" OR DE "CATHETERIZATION" OR DE "CHIROPRACTIC diagnosis" OR DE "CLINICAL pathology" OR DE "CLINICAL prediction rules" OR DE "COMMUNICABLE disease diagnosis" OR DE "COMPUTER-assisted medical diagnosis" OR DE "COVID-19 testing" OR DE "DIAGNOSTIC errors" OR DE "DIAGNOSTIC lasers" OR DE "DIAGNOSTIC services" OR DE "DIAGNOSTIC sex determination" OR DE "DIAGNOSTIC use of acupuncture points" OR DE "DIAGNOSTIC use of aerosols" OR DE "DIAGNOSTIC use of graphology" OR DE "DIAGNOSTIC use of transluminal angioplasty" OR DE "DIFFERENTIAL diagnosis" OR DE "DISABILITY evaluation" OR DE "DYES in medical diagnosis" OR DE "EARLY diagnosis" OR DE "ELECTRODIAGNOSIS" OR DE "EMERGENCY medical diagnosis" OR DE "ENDOSCOPY" OR DE "FAMILY medical history" OR DE "GENETIC disorder diagnosis" OR DE "GERIATRIC diagnosis" OR DE "GYNECOLOGIC diagnosis" OR DE "HUMAN chromosome abnormality diagnosis" OR DE "INCIDENTAL findings (Medicine)" OR DE "INFANT disease diagnosis" OR DE "INSUFFLATION" OR DE "INVASIVE diagnosis" OR DE "KYMOGRAPHY" OR DE "LYMPHATICS -- Puncture" OR DE "MEDICAL function tests" OR DE "MEDICAL history taking" OR DE "MEDICAL logic" OR DE "MEDICAL self-examination" OR DE "MOLECULAR diagnosis" OR DE "NEUROOPHTHALMOLOGICAL diagnosis" OR DE "NONINVASIVE diagnostic tests" OR DE "NURSING diagnosis" OR DE "OBSTETRICAL diagnosis" OR DE "ORTHODONTIC diagnosis" OR DE "ORTHOPEDIC diagnosis" OR DE "OTOLARYNGOLOGY diagnosis" OR DE "OVERDIAGNOSIS" OR DE "PATIENT monitoring" OR DE "PEDIATRIC cardiology diagnosis" OR DE "PEDIATRIC dentistry diagnosis" OR DE "PEDIATRIC dermatology diagnosis" OR DE "PEDIATRIC diagnosis" OR DE "PEDIATRIC gastroenterology diagnosis" OR DE "PEDIATRIC nephrology diagnosis" OR DE "PEDIATRIC neurology diagnosis" OR DE "PEDIATRIC urology diagnosis" OR DE "PHYSICAL diagnosis" OR DE "POINT-of-care testing" OR DE "PRENATAL diagnosis" OR DE "PROVOCATION tests (Medicine)" OR DE "PULSE diagnosis" OR DE "RADIOISOTOPES in medical diagnosis" OR DE "RADIOSCOPIC diagnosis" OR DE "RETROSPECTIVE diagnosis" OR DE "ROADSIDE sobriety tests" OR DE "ROUTINE diagnostic tests" OR DE "SELF diagnosis" OR DE "STABLE isotopes in medical diagnosis" OR DE "STERNUM -- Puncture" OR DE "SUBSTANCE abuse diagnosis" OR DE "SURGICAL diagnosis" OR DE "TONGUE diagnosis (Chinese medicine)" OR DE "VENOUS thrombosis diagnosis" OR DE "VETERINARY diagnosis" OR DE "VISUAL analog scale" OR DE "MEDICAL sciences" OR DE "BIOCHEMISTRY" OR DE "BIOPHYSICS" OR DE "HUMAN anatomy" OR DE "HUMAN physiology" OR DE "IMMUNOLOGY" OR DE "MEDICAL botany" OR DE "MEDICAL genetics" OR DE "MEDICAL geology" OR DE "MEDICAL microbiology" OR DE "MEDICAL parasitology" OR DE "NANOTECHNOLOGY & health" OR DE "NEUROSCIENCES" OR DE "OCCUPATIONAL science" OR DE "PATHOLOGY" OR DE "PHARMACOLOGY" OR DE "EPIDEMIOLOGY" OR DE "CLINICAL epidemiology" OR DE "COMMUNICABLE disease epidemiology" OR DE "COMORBIDITY" OR DE "CONTACT tracing (Epidemiology)" OR DE "DISEASE clusters" OR DE "DISEASE incidence" OR DE "DISEASE prevalence" OR DE "ENDEMIC infections" OR DE "EPIDEMICS" OR DE "FORENSIC epidemiology" OR DE "HEALTH transition" OR DE "HYPERTENSION epidemiology" OR DE "INFECTIOUS disease transmission" OR DE "MEDICAL record linkage" OR DE "MOLECULAR epidemiology" OR DE "PANDEMICS" OR DE "PEDIATRIC epidemiology" OR DE "PHARMACOEPIDEMIOLOGY" OR DE "PLANT epidemiology" OR DE "PSYCHIATRIC epidemiology" OR DE "PUBLIC health surveillance" OR DE "SEROPREVALENCE" OR DE "VETERINARY epidemiology" OR DE "DECISION making in clinical medicine" OR DE "CLINICAL prediction rules" OR DE "DISEASE management" OR DE "EVALUATION of dental services" OR DE "EVIDENCE-based medicine" OR DE "SPEECH -- Evaluation" OR DE "HEALTH services administration" OR DE "CLINICAL supervision" OR DE "COMMUNITY mental health service administration" OR DE "DISEASE management" OR DE "ENVIRONMENTAL health administration" OR DE "HEALTH facility administration" OR DE "HOSPITAL administration" OR DE "MEDICAL center administration" OR DE "NURSE-patient ratio" OR DE "NURSING care facility administration" OR DE "NURSING service administration" OR DE "PATIENT-centered care" OR DE "PHARMACY management" OR DE "RELATIVE value scales (Medical care)" OR DE "VALUE-based purchasing (Medical care)" OR DE "DISEASE complications" OR DE "ANOREXIA nervosa complications" OR DE "CANCER complications" OR DE "DIABETES complications" OR DE "HEART disease complications" OR DE "LUPUS erythematosus complications" OR DE "OBESITY complications" OR DE "SHINGLES complications" OR DE "DISEASES" OR DE "ACUTE diseases" OR DE "AGE factors in disease" OR DE "AIDS-related opportunistic infections" OR DE "ANIMAL diseases" OR DE "ATTITUDES toward disease" OR DE "AUTISM" OR DE "BLOOD diseases" OR DE "BOVINE spongiform encephalopathy" OR DE "BRAIN diseases" OR DE "CANCER" OR DE "CARDIOVASCULAR diseases" OR DE "CATASTROPHIC illness" OR DE "CENTRAL nervous system diseases" OR DE "CHRONIC diseases" OR DE "COMMUNICABLE diseases" OR DE "COMMUNICATIVE disorders" OR DE "CONGENITAL disorders" OR DE "CONSTITUTIONAL diseases" OR DE "DENTAL pathology" OR DE "DIGESTIVE system diseases" OR DE "DISABILITIES" OR DE "DISEASE duration" OR DE "DISEASE exacerbation" OR DE "DISEASE progression" OR DE "DISEASE relapse" OR DE "DISEASE remission" OR DE "DISEASES in alcoholics" OR DE "DISEASES in men" OR DE "DISEASES in women" OR DE "ENDOCRINE diseases" OR DE "ENVIRONMENTALLY induced diseases" OR DE "EYE diseases" OR DE "FAMILIAL diseases" OR DE "GENETIC disorders" OR DE "GENITOURINARY diseases" OR DE "HEMORRHAGIC diseases" OR DE "IATROGENIC diseases" OR DE "IMMUNOLOGIC diseases" OR DE "INCURABLE diseases" OR DE "JUVENILE diseases" OR DE "LIVER diseases" OR DE "MEDICAL emergencies" OR DE "MEMBRANE disorders" OR DE "MENTAL illness" OR DE "METABOLIC disorders" OR DE "MUSCULOSKELETAL system diseases" OR DE "NEUROLOGICAL disorders" OR DE "NON-communicable diseases" OR DE "NUTRITION disorders" OR DE "OCCUPATIONAL diseases" OR DE "PERIODIC diseases" OR DE "RARE diseases" OR DE "RESPIRATORY diseases" OR DE "RHEUMATISM" OR DE "SEXUALLY transmitted diseases" OR DE "SKIN diseases" OR DE "SUBCUTANEOUS emphysema" OR DE "SUBSTANCE-induced disorders" OR DE "SURGICAL diseases" OR DE "SYMPTOMS" OR DE "SYNDROMES" OR DE "TUMORS" OR DE "OPTOMETRY" OR DE "BEHAVIORAL optometry" OR DE "OPHTHALMIC lenses" OR DE "OPTICIANRY" OR DE "PEDIATRIC optometry" OR DE "VISION testing" OR DE "PATIENTS" OR DE "AGITATED patients" OR DE "ALLIED health personnel & patient" OR DE "AMNESIACS" OR DE "ARTHRITIS patients" OR DE "AUDIOLOGIST & patient" OR DE "BURN patients" OR DE "CANCER patients" OR DE "DENTAL personnel & patient" OR DE "DENTIST-patient relationship" OR DE "EMERGENCY medical technician & patient" OR DE "GYNECOLOGIST & patient" OR DE "HEALTH maintenance organization patients" OR DE "HEMODIALYSIS patients" OR DE "HIV-positive persons" OR DE "HOSPITAL patients" OR DE "IMMUNOCOMPROMISED patients" OR DE "LARYNGECTOMEES" OR DE "MEDICAL personnel as patients" OR DE "MENTAL health personnel & patient" OR DE "MUSIC therapist & patient" OR DE "NURSE-patient relationships" OR DE "NURSES as patients" OR DE "NURSING home patients" OR DE "OLDER patients" OR DE "PATIENT care conferences" OR DE "PATIENT dropouts" OR DE "PATIENT-family relations" OR DE "PATIENT-professional relations" OR DE "PATIENTS' families" OR DE "PHARMACIST-patient relationships" OR DE "PHYSICIAN-patient relations" OR DE "PHYSICIANS as patients" OR DE "PROBLEM patients" OR DE "PSYCHOTHERAPIST-patient relations" OR DE "PSYCHOTHERAPY patients" OR DE "SEXUALLY abused patients" OR DE "SIMULATED patients" OR DE "SPEECH therapist & patient" OR DE "TERMINALLY ill" OR DE "TRACHEOTOMY -- Patients" OR DE "WOMEN patients" OR DE "PREGNANCY" OR DE "AMNIOTIC liquid" OR DE "DURATION of pregnancy" OR DE "EXTRAMARITAL pregnancy" OR DE "FIRST pregnancy" OR DE "GRAVID uterus" OR DE "HIGH-risk pregnancy" OR DE "LABOR (Obstetrics)" OR DE "MALE pregnancy" OR DE "MATERNAL-fetal exchange" OR DE "METABOLISM in pregnancy" OR DE "MISCARRIAGE" OR DE "MULTIPLE pregnancy" OR DE "OVUM implantation" OR DE "PARITY (Obstetrics)" OR DE "PREGNANCY in mentally ill women" OR DE "PREGNANT women" OR DE "PRENATAL influences" OR DE "PSEUDOCYESIS" OR DE "SUBSEQUENT pregnancy" OR DE "TEENAGE pregnancy" OR DE "UNPLANNED pregnancy" OR DE "UNWANTED pregnancy" OR DE "PUBLIC health" OR DE "BIOSURVEILLANCE" OR DE "COMMUNITY health services" OR DE "DAIRY inspection" OR DE "DENTAL public health" OR DE "DISEASE eradication" OR DE "ECOSOCIAL theory (Social medicine)" OR DE "ENVIRONMENTAL health" OR DE "EPIDEMIOLOGY" OR DE "FOOD inspection" OR DE "HEALTH boards" OR DE "HEALTH facilities" OR DE "HEALTH impact assessment" OR DE "HEALTH planning" OR DE "HEALTH risk assessment" OR DE "HOUSING & health" OR DE "INTERMENT" OR DE "LABOR unions & public health" OR DE "MASS media & public health" OR DE "MEDICAL care" OR DE "MENTAL health" OR DE "POPULATION health" OR DE "PUBLIC health communication" OR DE "QUARANTINE" OR DE "REGIONAL medical programs" OR DE "RURAL health" OR DE "SANITARY districts" OR DE "SANITARY engineering" OR DE "SCHOOL hygiene" OR DE "SOCIAL distancing" OR DE "SOCIAL epidemiology" OR DE "SOCIAL medicine" OR DE "STAY-at-home orders" OR DE "UNIVERSAL precautions (Health)" OR DE "URBAN health" OR DE "VETERINARY public health" OR DE "VOLUNTEER workers in public health" OR DE "WORLD health" | #15 | 3 254 615 |
|  | TI ( care or chiroprati* or clinic* or communit* or dentist* or diagnos* or disease* or drug* or healing* or health* or illness or infection* or injur* or medicin* or medica* or nurs* or nutrition* or optometr* or orthoptic* or patholog* or patient or pharma* or pharmaceutic* or pill* or placebo* or poverty or pregnan* or prevention* or psychiatr* or psycholog* or psychosocial* or remed* or social* or surger* or therap* or treatment* or unemploy* or violence* or wound* )  OR AB ( care or chiroprati* or clinic* or communit* or dentist* or diagnos* or disease* or drug* or healing* or health* or illness or infection* or injur* or medicin* or medica* or nurs* or nutrition* or optometr* or orthoptic* or patholog* or patient or pharma* or pharmaceutic* or pill* or placebo* or poverty or pregnan* or prevention* or psychiatr* or psycholog* or psychosocial* or remed* or social* or surger* or therap* or treatment* or unemploy* or violence* or wound* )  OR SU ( care or chiroprati* or clinic* or communit* or dentist* or diagnos* or disease* or drug* or healing* or health* or illness or infection* or injur* or medicin* or medica* or nurs* or nutrition* or optometr* or orthoptic* or patholog* or patient or pharma* or pharmaceutic* or pill* or placebo* or poverty or pregnan* or prevention* or psychiatr* or psycholog* or psychosocial* or remed* or social* or surger* or therap* or treatment* or unemploy* or violence* or wound* ) | #16 | 12 259 500 |
|  | S15 or S16 | #17 | 12 442 309 |
| Total Result | S6 and S11 and S14 and S17 | #18 | 1 425 |

### Academic Search Premier-EBSCO-(2024-02-05)

| 1 | DE "DIFFUSION of innovations" OR DE "ECONOMIES of scale" OR DE "DIFFUSION of innovations theory" OR DE "INNOVATION adoption" OR DE "TECHNOLOGY transfer" | 17001 |
| --- | --- | --- |
| 2 | TI ( ("scal* up" or "scal* out") ) OR AB ( ("scal* up" or "scal* out") ) OR SU ( ("scal* up" or "scal* out") ) | 34720 |
| 3 | TI (("scaling" or widespread or spread# or spreading or "rolling out" or "roll out" or "rolls out" or "rolled out" or upscaling or scalability or scalable) N4 (innovation# or intervention# or technolog* or practice* or care or initiative* or program* or product# or therap* or service* or strateg* or change# or proces*)) OR AB (("scaling" or widespread or spread# or spreading or "rolling out" or "roll out" or "rolls out" or "rolled out" or upscaling or scalability or scalable) N4 (innovation# or intervention# or technolog* or practice* or care or initiative* or program* or product# or therap* or service* or strateg* or change# or proces*)) OR SU (("scaling" or widespread or spread# or spreading or "rolling out" or "roll out" or "rolls out" or "rolled out" or upscaling or scalability or scalable) N4 (innovation# or intervention# or technolog* or practice* or care or initiative* or program* or product# or therap* or service* or strateg* or change# or proces*) ) | 51929 |
| 4 | TI ( (bring* or brought or taking or take* or increas* or going or implement* or econom*) N4 scal* N4 (innovation# or intervention# or technolog* or practice* or care or initiative* or program* or product# or therap* or service* or strateg* or change# or proces*) ) OR AB ( (bring* or brought or taking or take* or increas* or going or implement* or econom*) N4 scal* N4 (innovation# or intervention# or technolog* or practice* or care or initiative* or program* or product# or therap* or service* or strateg* or change# or proces*) ) OR SU ( (bring* or brought or taking or take* or increas* or going or implement* or econom*) N4 scal* N4 (innovation# or intervention# or technolog* or practice* or care or initiative* or program* or product# or therap* or service* or strateg* or change# or proces*) ) | 6496 |
| 5 | S2 OR S3 OR S4 | 88267 |
| 6 | (S1 OR S5) | 104702 |
| 7 | DE "COMMUNITY-based participatory research" | 2955 |
| 8 | TI ((caregiver* or care-giver* or citizen* or client* or communit* or consumer* or "family carer*" or lay or patient* or "peer helper*" or public or stakeholder* or survivor* or user*) N1 (collaborat* or cooperat* or co-operat* or coproduc* or co-produc* or engag* or includ* or involve* or "joint effort*" or mobilis* or mobiliz* or participat* or partner* or "work together" or "working together")) OR AB ((caregiver* or care-giver* or citizen* or client* or communit* or consumer* or "family carer*" or lay or patient* or "peer helper*" or public or stakeholder* or survivor* or user*) N1 (collaborat* or cooperat* or co-operat* or coproduc* or co-produc* or engag* or includ* or involve* or "joint effort*" or mobilis* or mobiliz* or participat* or partner* or "work together" or "working together")) OR SU ( (caregiver* or care-giver* or citizen* or client* or communit* or consumer* or "family carer*" or lay or patient* or "peer helper*" or public or stakeholder* or survivor* or user*) N1 (collaborat* or cooperat* or co-operat* or coproduc* or co-produc* or engag* or includ* or involve* or "joint effort*" or mobilis* or mobiliz* or participat* or partner* or "work together" or "working together") ) | 388307 |
| 9 | TI ( Co-build* or co-constr* or co-creation or coproduc* or co-produc* ) OR AB ( Co-build* or co-constr* or co-creation or coproduc* or co-produc* ) OR SU ( Co-build* or co-constr* or co-creation or coproduc* or co-produc* ) | 19925 |
| 10 | S8 OR S9 | 406399 |
| 11 | (S7 OR S10) | 406700 |
| 12 | DE "GUIDELINES" OR DE "METHODOLOGY" OR DE "TOOLS" | 61930 |
| 13 | TI ( Action or actions or approach* or "frame work*" or Framework* or guidance* or guide or guides or guideline* or how or hows or methodolog* or model or models or practice* or principle* or process or recommend* or stratagem* or strateg* or tool or toolkit* or tools ) OR AB ( Action or actions or approach* or "frame work*" or Framework* or guidance* or guide or guides or guideline* or how or hows or methodolog* or model or models or practice* or principle* or process or recommend* or stratagem* or strateg* or tool or toolkit* or tools ) OR SU ( Action or actions or approach* or "frame work*" or Framework* or guidance* or guide or guides or guideline* or how or hows or methodolog* or model or models or practice* or principle* or process or recommend* or stratagem* or strateg* or tool or toolkit* or tools ) | 14814895 |
| 14 | (S12 OR S13) | 14816812 |
| 15 | DE "CAREGIVERS" OR DE "CARE of aging parents" OR DE "CARE of Alzheimer's patients" OR DE "CARE of dementia patients" OR DE "CARE of Parkinson's disease patients" OR DE "CHILD caregivers" OR DE "CLINIC employees" OR DE "DEMENTIA care mapping" OR DE "HEALTH" OR DE "HEALTH facility employees" OR DE "LGBTQ+ caregivers" OR DE "LIBRARIES & caregivers" OR DE "LONG-distance caregivers" OR DE "MALE caregivers" OR DE "MEDICAL personnel-caregiver relationships" OR DE "MENTAL health facility employees" OR DE "NURSING home employees" OR DE "OLDER caregivers" OR DE "RURAL caregivers" OR DE "UTILIZATION of clinics" OR DE "WOMEN caregivers" OR DE "MEDICINE" OR DE "ADOLESCENT medicine" OR DE "ALTERNATIVE medicine" OR DE "ANCIENT medicine" OR DE "ANESTHESIOLOGY" OR DE "AUDIOLOGY" OR DE "AUTOMOTIVE medicine" OR DE "BIOMEDICAL engineering" OR DE "BLOOD as food or medicine" OR DE "BLOODLETTING" OR DE "BUDDHIST medicine" OR DE "BUSINESS & medicine" OR DE "CIRCUMPOLAR medicine" OR DE "CLINICAL medicine" OR DE "COMMUNISM & medicine" OR DE "COMPUTER vision in medicine" OR DE "CREATIVE ability in medicine" OR DE "CRITICAL care medicine" OR DE "CURATIVE medicine" OR DE "DARWINIAN medicine" OR DE "DEFENSIVE medicine" OR DE "DENTISTRY" OR DE "DERMATOLOGY" OR DE "DIAGNOSIS" OR DE "DISASTER medicine" OR DE "ELECTRICITY in medicine" OR DE "EMERGENCY medicine" OR DE "ENDOCRINOLOGY" OR DE "ENVIRONMENTAL medicine" OR DE "FAMILY medicine" OR DE "FUZZY systems in medicine" OR DE "GERIATRICS" OR DE "GYNECOLOGY" OR DE "HEALTH" OR DE "HETEROSEXISM in medicine" OR DE "HOMOPHOBIA in medicine" OR DE "HORMONE therapy" OR DE "INTEGRATIVE medicine" OR DE "INTERNAL medicine" OR DE "INTERNET in medicine" OR DE "LANGUAGE & medicine" OR DE "LITERATURE & medicine" OR DE "MEDICAL advertising" OR DE "MEDICAL climatology" OR DE "MEDICAL communication" OR DE "MEDICAL jurisprudence" OR DE "MEDICAL parasitology" OR DE "MEDICAL robotics" OR DE "MEDICINE & art" OR DE "MEDICINE & philosophy" OR DE "MEDICINE & theater" OR DE "MEDIEVAL medicine" OR DE "MENTORING in medicine" OR DE "MIDWIFERY" OR DE "MILITARY medicine" OR DE "MINORITIES in medicine" OR DE "MISSIONARY medicine" OR DE "MOTION pictures & medicine" OR DE "MOTION pictures in medicine" OR DE "NANOMEDICINE" OR DE "NATIONAL socialism & medicine" OR DE "NAVAL medicine" OR DE "NEUROLOGY" OR DE "NOSOLOGY" OR DE "NURSING" OR DE "OBSTETRICS" OR DE "OCCUPATIONAL medicine" OR DE "ONCOLOGY" OR DE "OPHTHALMOLOGY" OR DE "OPTICAL fibers in medicine" OR DE "ORAL medicine" OR DE "ORTHOPEDICS" OR DE "OSTEOPATHIC medicine" OR DE "OTOLARYNGOLOGY" OR DE "PAIN medicine" OR DE "PEDIATRICS" OR DE "PHARMACY" OR DE "PHYSICAL medicine" OR DE "PLASTICS in medicine" OR DE "PODIATRY" OR DE "PREVENTIVE medicine" OR DE "PSYCHIATRY" OR DE "QUACKS & quackery" OR DE "RACISM in medicine" OR DE "RADIO in medicine" OR DE "REGENERATIVE medicine" OR DE "SEX discrimination in medicine" OR DE "SEXISM in medicine" OR DE "SOUND recordings in medicine" OR DE "SPACE medicine" OR DE "SURGERY" OR DE "TELEMEDICINE" OR DE "TELEVISION in medicine" OR DE "TOXICOLOGY" OR DE "TROPICAL medicine" OR DE "UROLOGY" OR DE "VASCULAR medicine" OR DE "VETERINARY medicine" OR DE "VIDEO recording in medicine" OR DE "VIRTUAL reality in medicine" OR DE "WILDERNESS medicine" OR DE "WIT & humor in medicine" OR DE "WOMEN in medicine" OR DE "MEDICAL personnel" OR DE "ABORIGINAL Australians in medicine" OR DE "ALLIED health personnel" OR DE "AROMATHERAPISTS" OR DE "BIOMEDICAL engineers" OR DE "BLACK people in medicine" OR DE "CHIROPRACTORS" OR DE "DENTAL personnel" OR DE "EMERGENCY medical personnel" OR DE "HEALTH care teams" OR DE "HEALTH occupations students" OR DE "HEALTH practitioners" OR DE "HEALTH services administrators" OR DE "HOSPITAL personnel" OR DE "IMPAIRED medical personnel" OR DE "MEDICAL personnel as patients" OR DE "MEDICAL personnel-caregiver relationships" OR DE "MEDICAL registry personnel" OR DE "MEDICAL research personnel" OR DE "MEDICAL scribes" OR DE "MEDICAL specialties & specialists" OR DE "MEDICAL teaching personnel" OR DE "MENTAL health personnel" OR DE "MIDWIVES" OR DE "MILITARY medical personnel" OR DE "MINORITY medical personnel" OR DE "MULTISKILLED medical personnel" OR DE "NATIVE Americans in medicine" OR DE "NURSES" OR DE "OPERATING room personnel" OR DE "OPTOMETRISTS" OR DE "ORGAN transplant coordinators" OR DE "PATIENT-professional relations" OR DE "PHARMACISTS" OR DE "PHLEBOTOMISTS" OR DE "PHYSICIANS" OR DE "PODIATRISTS" OR DE "PROSTHETISTS" OR DE "PUBLIC health personnel" OR DE "RECOVERY room personnel" OR DE "STUDENT volunteers in medical care" OR DE "TRAVELING medical personnel" OR DE "UNLICENSED medical personnel" OR DE "DIAGNOSIS" OR DE "ALLERGY diagnosis" OR DE "CANCER diagnosis" OR DE "CATHETERIZATION" OR DE "CHIROPRACTIC diagnosis" OR DE "CLINICAL pathology" OR DE "CLINICAL prediction rules" OR DE "COMMUNICABLE disease diagnosis" OR DE "COMPUTER-assisted medical diagnosis" OR DE "COVID-19 testing" OR DE "DIAGNOSTIC errors" OR DE "DIAGNOSTIC lasers" OR DE "DIAGNOSTIC services" OR DE "DIAGNOSTIC sex determination" OR DE "DIAGNOSTIC use of acupuncture points" OR DE "DIAGNOSTIC use of aerosols" OR DE "DIAGNOSTIC use of graphology" OR DE "DIAGNOSTIC use of transluminal angioplasty" OR DE "DIFFERENTIAL diagnosis" OR DE "DISABILITY evaluation" OR DE "DYES in medical diagnosis" OR DE "EARLY diagnosis" OR DE "ELECTRODIAGNOSIS" OR DE "EMERGENCY medical diagnosis" OR DE "ENDOSCOPY" OR DE "FAMILY medical history" OR DE "GENETIC disorder diagnosis" OR DE "GERIATRIC diagnosis" OR DE "GYNECOLOGIC diagnosis" OR DE "HUMAN chromosome abnormality diagnosis" OR DE "INCIDENTAL findings (Medicine)" OR DE "INFANT disease diagnosis" OR DE "INSUFFLATION" OR DE "INVASIVE diagnosis" OR DE "KYMOGRAPHY" OR DE "LYMPHATICS -- Puncture" OR DE "MEDICAL function tests" OR DE "MEDICAL history taking" OR DE "MEDICAL logic" OR DE "MEDICAL self-examination" OR DE "MOLECULAR diagnosis" OR DE "NEUROOPHTHALMOLOGICAL diagnosis" OR DE "NONINVASIVE diagnostic tests" OR DE "NURSING diagnosis" OR DE "OBSTETRICAL diagnosis" OR DE "ORTHODONTIC diagnosis" OR DE "ORTHOPEDIC diagnosis" OR DE "OTOLARYNGOLOGY diagnosis" OR DE "OVERDIAGNOSIS" OR DE "PATIENT monitoring" OR DE "PEDIATRIC cardiology diagnosis" OR DE "PEDIATRIC dentistry diagnosis" OR DE "PEDIATRIC dermatology diagnosis" OR DE "PEDIATRIC diagnosis" OR DE "PEDIATRIC gastroenterology diagnosis" OR DE "PEDIATRIC nephrology diagnosis" OR DE "PEDIATRIC neurology diagnosis" OR DE "PEDIATRIC urology diagnosis" OR DE "PHYSICAL diagnosis" OR DE "POINT-of-care testing" OR DE "PRENATAL diagnosis" OR DE "PROVOCATION tests (Medicine)" OR DE "PULSE diagnosis" OR DE "RADIOISOTOPES in medical diagnosis" OR DE "RADIOSCOPIC diagnosis" OR DE "RETROSPECTIVE diagnosis" OR DE "ROADSIDE sobriety tests" OR DE "ROUTINE diagnostic tests" OR DE "SELF diagnosis" OR DE "STABLE isotopes in medical diagnosis" OR DE "STERNUM -- Puncture" OR DE "SUBSTANCE abuse diagnosis" OR DE "SURGICAL diagnosis" OR DE "TONGUE diagnosis (Chinese medicine)" OR DE "VENOUS thrombosis diagnosis" OR DE "VETERINARY diagnosis" OR DE "VISUAL analog scale" OR DE "MEDICAL sciences" OR DE "BIOCHEMISTRY" OR DE "BIOPHYSICS" OR DE "HUMAN anatomy" OR DE "HUMAN physiology" OR DE "IMMUNOLOGY" OR DE "MEDICAL botany" OR DE "MEDICAL genetics" OR DE "MEDICAL geology" OR DE "MEDICAL microbiology" OR DE "MEDICAL parasitology" OR DE "NANOTECHNOLOGY & health" OR DE "NEUROSCIENCES" OR DE "OCCUPATIONAL science" OR DE "PATHOLOGY" OR DE "PHARMACOLOGY" OR DE "EPIDEMIOLOGY" OR DE "CLINICAL epidemiology" OR DE "COMMUNICABLE disease epidemiology" OR DE "COMORBIDITY" OR DE "CONTACT tracing (Epidemiology)" OR DE "DISEASE clusters" OR DE "DISEASE incidence" OR DE "DISEASE prevalence" OR DE "ENDEMIC infections" OR DE "EPIDEMICS" OR DE "FORENSIC epidemiology" OR DE "HEALTH transition" OR DE "HYPERTENSION epidemiology" OR DE "INFECTIOUS disease transmission" OR DE "MEDICAL record linkage" OR DE "MOLECULAR epidemiology" OR DE "PANDEMICS" OR DE "PEDIATRIC epidemiology" OR DE "PHARMACOEPIDEMIOLOGY" OR DE "PLANT epidemiology" OR DE "PSYCHIATRIC epidemiology" OR DE "PUBLIC health surveillance" OR DE "SEROPREVALENCE" OR DE "VETERINARY epidemiology" OR DE "DECISION making in clinical medicine" OR DE "CLINICAL prediction rules" OR DE "DISEASE management" OR DE "EVALUATION of dental services" OR DE "EVIDENCE-based medicine" OR DE "SPEECH -- Evaluation" OR DE "HEALTH services administration" OR DE "CLINICAL supervision" OR DE "COMMUNITY mental health service administration" OR DE "DISEASE management" OR DE "ENVIRONMENTAL health administration" OR DE "HEALTH facility administration" OR DE "HOSPITAL administration" OR DE "MEDICAL center administration" OR DE "NURSE-patient ratio" OR DE "NURSING care facility administration" OR DE "NURSING service administration" OR DE "PATIENT-centered care" OR DE "PHARMACY management" OR DE "RELATIVE value scales (Medical care)" OR DE "VALUE-based purchasing (Medical care)" OR DE "DISEASE complications" OR DE "ANOREXIA nervosa complications" OR DE "CANCER complications" OR DE "DIABETES complications" OR DE "HEART disease complications" OR DE "LUPUS erythematosus complications" OR DE "OBESITY complications" OR DE "SHINGLES complications" OR DE "DISEASES" OR DE "ACUTE diseases" OR DE "AGE factors in disease" OR DE "AIDS-related opportunistic infections" OR DE "ANIMAL diseases" OR DE "ATTITUDES toward disease" OR DE "AUTISM" OR DE "BLOOD diseases" OR DE "BOVINE spongiform encephalopathy" OR DE "BRAIN diseases" OR DE "CANCER" OR DE "CARDIOVASCULAR diseases" OR DE "CATASTROPHIC illness" OR DE "CENTRAL nervous system diseases" OR DE "CHRONIC diseases" OR DE "COMMUNICABLE diseases" OR DE "COMMUNICATIVE disorders" OR DE "CONGENITAL disorders" OR DE "CONSTITUTIONAL diseases" OR DE "DENTAL pathology" OR DE "DIGESTIVE system diseases" OR DE "DISABILITIES" OR DE "DISEASE duration" OR DE "DISEASE exacerbation" OR DE "DISEASE progression" OR DE "DISEASE relapse" OR DE "DISEASE remission" OR DE "DISEASES in alcoholics" OR DE "DISEASES in men" OR DE "DISEASES in women" OR DE "ENDOCRINE diseases" OR DE "ENVIRONMENTALLY induced diseases" OR DE "EYE diseases" OR DE "FAMILIAL diseases" OR DE "GENETIC disorders" OR DE "GENITOURINARY diseases" OR DE "HEMORRHAGIC diseases" OR DE "IATROGENIC diseases" OR DE "IMMUNOLOGIC diseases" OR DE "INCURABLE diseases" OR DE "JUVENILE diseases" OR DE "LIVER diseases" OR DE "MEDICAL emergencies" OR DE "MEMBRANE disorders" OR DE "MENTAL illness" OR DE "METABOLIC disorders" OR DE "MUSCULOSKELETAL system diseases" OR DE "NEUROLOGICAL disorders" OR DE "NON-communicable diseases" OR DE "NUTRITION disorders" OR DE "OCCUPATIONAL diseases" OR DE "PERIODIC diseases" OR DE "RARE diseases" OR DE "RESPIRATORY diseases" OR DE "RHEUMATISM" OR DE "SEXUALLY transmitted diseases" OR DE "SKIN diseases" OR DE "SUBCUTANEOUS emphysema" OR DE "SUBSTANCE-induced disorders" OR DE "SURGICAL diseases" OR DE "SYMPTOMS" OR DE "SYNDROMES" OR DE "TUMORS" OR DE "OPTOMETRY" OR DE "BEHAVIORAL optometry" OR DE "OPHTHALMIC lenses" OR DE "OPTICIANRY" OR DE "PEDIATRIC optometry" OR DE "VISION testing" OR DE "PATIENTS" OR DE "AGITATED patients" OR DE "ALLIED health personnel & patient" OR DE "AMNESIACS" OR DE "ARTHRITIS patients" OR DE "AUDIOLOGIST & patient" OR DE "BURN patients" OR DE "CANCER patients" OR DE "DENTAL personnel & patient" OR DE "DENTIST-patient relationship" OR DE "EMERGENCY medical technician & patient" OR DE "GYNECOLOGIST & patient" OR DE "HEALTH maintenance organization patients" OR DE "HEMODIALYSIS patients" OR DE "HIV-positive persons" OR DE "HOSPITAL patients" OR DE "IMMUNOCOMPROMISED patients" OR DE "LARYNGECTOMEES" OR DE "MEDICAL personnel as patients" OR DE "MENTAL health personnel & patient" OR DE "MUSIC therapist & patient" OR DE "NURSE-patient relationships" OR DE "NURSES as patients" OR DE "NURSING home patients" OR DE "OLDER patients" OR DE "PATIENT care conferences" OR DE "PATIENT dropouts" OR DE "PATIENT-family relations" OR DE "PATIENT-professional relations" OR DE "PATIENTS' families" OR DE "PHARMACIST-patient relationships" OR DE "PHYSICIAN-patient relations" OR DE "PHYSICIANS as patients" OR DE "PROBLEM patients" OR DE "PSYCHOTHERAPIST-patient relations" OR DE "PSYCHOTHERAPY patients" OR DE "SEXUALLY abused patients" OR DE "SIMULATED patients" OR DE "SPEECH therapist & patient" OR DE "TERMINALLY ill" OR DE "TRACHEOTOMY -- Patients" OR DE "WOMEN patients" OR DE "PREGNANCY" OR DE "AMNIOTIC liquid" OR DE "DURATION of pregnancy" OR DE "EXTRAMARITAL pregnancy" OR DE "FIRST pregnancy" OR DE "GRAVID uterus" OR DE "HIGH-risk pregnancy" OR DE "LABOR (Obstetrics)" OR DE "MALE pregnancy" OR DE "MATERNAL-fetal exchange" OR DE "METABOLISM in pregnancy" OR DE "MISCARRIAGE" OR DE "MULTIPLE pregnancy" OR DE "OVUM implantation" OR DE "PARITY (Obstetrics)" OR DE "PREGNANCY in mentally ill women" OR DE "PREGNANT women" OR DE "PRENATAL influences" OR DE "PSEUDOCYESIS" OR DE "SUBSEQUENT pregnancy" OR DE "TEENAGE pregnancy" OR DE "UNPLANNED pregnancy" OR DE "UNWANTED pregnancy" OR DE "PUBLIC health" OR DE "BIOSURVEILLANCE" OR DE "COMMUNITY health services" OR DE "DAIRY inspection" OR DE "DENTAL public health" OR DE "DISEASE eradication" OR DE "ECOSOCIAL theory (Social medicine)" OR DE "ENVIRONMENTAL health" OR DE "EPIDEMIOLOGY" OR DE "FOOD inspection" OR DE "HEALTH boards" OR DE "HEALTH facilities" OR DE "HEALTH impact assessment" OR DE "HEALTH planning" OR DE "HEALTH risk assessment" OR DE "HOUSING & health" OR DE "INTERMENT" OR DE "LABOR unions & public health" OR DE "MASS media & public health" OR DE "MEDICAL care" OR DE "MENTAL health" OR DE "POPULATION health" OR DE "PUBLIC health communication" OR DE "QUARANTINE" OR DE "REGIONAL medical programs" OR DE "RURAL health" OR DE "SANITARY districts" OR DE "SANITARY engineering" OR DE "SCHOOL hygiene" OR DE "SOCIAL distancing" OR DE "SOCIAL epidemiology" OR DE "SOCIAL medicine" OR DE "STAY-at-home orders" OR DE "UNIVERSAL precautions (Health)" OR DE "URBAN health" OR DE "VETERINARY public health" OR DE "VOLUNTEER workers in public health" OR DE "WORLD health" | 3926804 |
| 16 | TI ( care or chiroprati* or clinic* or communit* or dentist* or diagnos* or disease* or drug* or healing* or health* or illness or infection* or injur* or medicin* or medica* or nurs* or nutrition* or optometr* or orthoptic* or patholog* or patient or pharma* or pharmaceutic* or pill* or placebo* or poverty or pregnan* or prevention* or psychiatr* or psycholog* or psychosocial* or remed* or social* or surger* or therap* or treatment* or unemploy* or violence* or wound* ) OR AB ( care or chiroprati* or clinic* or communit* or dentist* or diagnos* or disease* or drug* or healing* or health* or illness or infection* or injur* or medicin* or medica* or nurs* or nutrition* or optometr* or orthoptic* or patholog* or patient or pharma* or pharmaceutic* or pill* or placebo* or poverty or pregnan* or prevention* or psychiatr* or psycholog* or psychosocial* or remed* or social* or surger* or therap* or treatment* or unemploy* or violence* or wound* ) OR SU ( care or chiroprati* or clinic* or communit* or dentist* or diagnos* or disease* or drug* or healing* or health* or illness or infection* or injur* or medicin* or medica* or nurs* or nutrition* or optometr* or orthoptic* or patholog* or patient or pharma* or pharmaceutic* or pill* or placebo* or poverty or pregnan* or prevention* or psychiatr* or psycholog* or psychosocial* or remed* or social* or surger* or therap* or treatment* or unemploy* or violence* or wound* | 15142404 |
| 17 | (S15 OR S16) | 15355329 |
| 18 | S6 AND S11 AND S14 AND S17 | 2363 |
| 19 | S18 AND Limiters - Publication Date: 20200901-20240231 | 942 |
| FINAL | S18 AND Limiters - Publication Date: 20200901-20240231 | 942 |

**GREY LITERATURE**

**Google (2022-02-07)**

<https://www.google.com/advanced_search>

| **Search strategy keywords** | **Search** | **# Results** |
| --- | --- | --- |
| “scaling up” AND “patient OR public” AND “involvement OR engagement” filetype:pdf” | #1 | 1 880 000 |
| “scaling out” AND “patient OR public” AND “involvement OR engagement” filetype:pdf | #2 | 25 900 |
| “scale out”AND“patient OR public” “involvement OR engagement” filetype:pdf | #3 | 62 200 |
| “Scale up” AND “patient OR public” AND “involvement OR engagement” filetype:pdf” | #4 | 27 700 000 |

**Canadian Agency for Drugs and Technologies in Health (CADTH) (2022-02-04)**

<https://www.cadth.ca/resources>

| **Search strategy keywords** | **Search** | **# Results** |
| --- | --- | --- |
| scaling | #1 | 8 |
| scale-up | #2 | 2 |
| scale-out | #3 | 0 |
| échelle | #4 | 0 |

**International Development Research Centre (IDRC) (2022-02-04)**

<https://www.idrc.ca/en>

*Filter topics: health et social policy

| **Search strategy keywords** | **Search** | **# Results** |
| --- | --- | --- |
| scaling | #1 | 119 |
| “scale-up” | #2 | 48 |
| “scale-out” | #3 | 18 |
| “mise à l'échelle” | #4 | 1 |
| "passage à l’échelle" | #5 | 1 |

**National Institute for Health and Care Excellence (NICE) (2022-02-07)**

<https://www.nice.org.uk/>

*Filter: secondary evidence

| **Search strategy keywords** | **Search** | **# Results** |
| --- | --- | --- |
| scaling engagement | #1 | 583 |
| scaling involvement | #2 | 535 |
| “scale up” engagement | #3 | 3 164 |
| “scale up” involvement | #4 | 3 496 |
| “scale out” engagement | #5 | 3 147 |
| “scale out” involvement | #6 | 3 381 |

**Australia NSW Government (NSW Heath) (2022-02-04)**

<https://www.health.nsw.gov.au/Pages/default.aspx>

| **Search strategy keywords** | **Search** | **# Results** |
| --- | --- | --- |
| scaling | #1 | 115 |
| “scale up” | #2 | 67 |
| “scale out” | #3 | 0 |

**The Canadian Foundation for Healthcare Improvement (CFHI) (2022-02-04)**

<https://www.cfhi-fcass.ca/>

| **Search strategy keywords** | **Search** | **# Results** |
| --- | --- | --- |
| scaling | #1 | 34 |
| “scale up” | #2 | 108 |
| scale up | #3 | 108 |
| “scale out” | #4 | 127 |
| échelle | #5 | 0 |
| "accroissement d’échelle" | #6 | 0 |

**Institute for Healthcare Improvement (IHI) (2022-02-04)**

[www.ihi.org](http://www.ihi.org)

*Content type: publications

| **Search strategy keywords** | **Search** | **# Results** |
| --- | --- | --- |
| scaling | #1 | 9 |
| “scale up” | #2 | 16 |
| scale up | #3 | 0 |

**National Implementation Research Network (NIRN) (2022-02-04)**

<https://nirn.fpg.unc.edu/publications-resources>

| **Search strategy keywords** | **Search** | **# Results** |
| --- | --- | --- |
| scaling | #1 | 3 |
| “scaling up” | #2 | 3 |
| “scale up” | #3 | 0 |
| “scale out” | #4 | 0 |

**The State Implementation & Scaling‐up of Evidence‐based Practices Center (SISEP) (2022-02-04)**

<https://nirn.fpg.unc.edu/ai-hub/resources>

| **Search strategy keywords** | **Search** | **# Results** |
| --- | --- | --- |
| scaling | #1 | 8 |
| “scaling up” | #2 | 7 |
| “scale up” | #3 | 2 |
| “scale out” | #4 | 2 |

**WHO/ExpandNet (2022-02-07)**

<http://expandnet.net/biblio/>

| **Search strategy keywords** | **Search** | **# Results** |
| --- | --- | --- |
| Hand search on all website | #1 | 580 |

**Australian Prevention Partnership Centre (2022-02-04)**

<https://preventioncentre.org.au>

| **Search strategy keywords** | **Search** | **# Results** |
| --- | --- | --- |
| scaling | #1 | 33 |
| “scale up” | #2 | 118 |
| “scale out” | #3 | 0 |

**Agency for Healthcare Research and Quality (AHRQ) (2022-02-24)**

<https://www.ahrq.gov>

| **Search strategy keywords** | **Search** | **# Results** |
| --- | --- | --- |
| scaling engagement | #1 | 2 784 |
| scaling involvement | #2 | 10 000 |
| “scale up” engagement | #3 | 2 293 |
| “scale up” involvement | #4 | 4 449 |
| “scale out” engagement | #5 | 1 969 |
| “scale out” involvement | #6 | 3 468 |

**Global community of practice on scaling development outcomes (2022-02-10)**

[www.scalingcommunityofpractice.com](http://www.scalingcommunityofpractice.com)

| **Search strategy keywords** | **Search** | **# Results** |
| --- | --- | --- |
| engagement | #1 | 14 |
| involvement | #2 | 2 |

### Consulted Reference

Allaire, J.-F., St-Martin, K.-A., Massougbodji, J., Zomahoun, H.T.V., Langlois, L. (2018). S’outiller pour favoriser la participation des usagers, des proches, des citoyens et des communautés à l’amélioration continue de la qualité des soins et des services : Recueil d’idées inspirantes. Sous la direction de Paul Morin. Institut universitaire de première ligne en santé et services sociaux du CIUSSS de l’Estrie – CHUS

Chambers, E., Gardiner, C., Thompson, J., & Seymour, J. (2019). Patient and carer involvement in palliative care research: An integrative qualitative evidence synthesis review. *Palliative medicine*, *33*(8), 969-984.

Harrison, J. D., Auerbach, A. D., Anderson, W., Fagan, M., Carnie, M., Hanson, C., ... & Wong, C. (2019). Patient stakeholder engagement in research: a narrative review to describe foundational principles and best practice activities. *Health Expectations*, *22*(3), 307-316.

Petkovic, Jennifer, et al. "Protocol for the development of guidance for stakeholder engagement in health and healthcare guideline development and implementation." *Systematic reviews* 9.1 (2020): 1-11.

Pii, K. H., Schou, L. H., Piil, K., & Jarden, M. (2019). Current trends in patient and public involvement in cancer research: a systematic review. *Health Expectations*, *22*(1), 3-20.

Miah, J., Dawes, P., Edwards, S., Leroi, I., Starling, B., & Parsons, S. (2019). Patient and public involvement in dementia research in the European Union: a scoping review. *BMC geriatrics*, *19*(1), 220.

Vat, L. E., Finlay, T., Jan Schuitmaker‐Warnaar, T., Fahy, N., Robinson, P., Boudes, M., ... & Kürzinger, M. L. (2020). Evaluating the “return on patient engagement initiatives” in medicines research and development: A literature review. *Health Expectations*, *23*(1), 5-18.
