# Supplementary material for: Strategies for involving patients and the public in scaling initiatives in health and social services: A scoping review
Source: Health Expect. 2024 Jun 5;27(3):e14086. doi: 10.1111/hex.14086 (PMC11150745; doi:10.1111/hex.14086)
Supplement: Supplementary file 5 — Supporting information. [file HEX-27-e14086-s008.docx]

**Additional File 5 – Eligibility criteria**

| **Inclusion criteria** | **Exclusion criteria** |
| --- | --- |
| *Population* | |
| - Patients (e.g., representatives, patients, and families) - Targeted-groups and/or beneficiary populations (e.g., women, people living with HIV-AIDS) - Community (e.g., community members, local people) - Civil society organizations (e.g., non-governmental organization, non-profit organizations) - Leaders (e.g., opinion leaders, community leaders, religion leaders) - Users (e.g., clients, service users, Target user, individual consumers) - Citizens, i.e., any individual described as a citizen - Volunteers, i.e., any individual described as a volunteer | - Does not include patients and/or the public as listed besides |
| *Intervention* | |
| Any strategy presenting a general approach to enabling patients and the public to receive information, provide advice, collaborate in or co-construct the scaling initiatives.  We considered as scaling initiatives:   - The act of scaling, spreading, replicating, adapting, expanding, or transferring an intervention that has already been tested in a pilot/experimental/small-scale context to other settings or populations - The act of developing or piloting an intervention with the aim of scaling it using and reporting scaling methods - Research on or for scaling, that uses and reports scaling objectives and methods | - Does not report the PPI method, i.e., does not report the action/activity by which the patients and the public are involved (e.g., interviews, meetings) - Does not involve patients and the public through information, consultation, collaboration, or coproduction process - Patients and/or the public are just the targeted population of the scaling intervention (receiving the scaled intervention) - Report only the implementation of an evidence- based practice - Report only a large-scale implementation of an evidence- based practice, without a previous implementation in a pilot/experimental/small-scale context - Does not report scaling methods, results, and discussion in the text, even if the scaling is announced in the title, abstract or introduction |
| *Comparison(s)* | |
| Include papers with or without a comparator group. | Not applicable. |
| *Outcome* | |
| Internal outcomes:   - Impact on involvement participants themselves (knowledge, skills, empowerment, satisfaction, trust) - Impact on the services provided by the organization or system (efficiency and cost-effectiveness of services, service availability, services quality and safety, services responsiveness to needs, utilization of services) - Impact on the organization or system (Awareness or knowledge of health issues, support of the organization or system)   External outcomes:   - Influence on the broader public (accountability of organization to patient and public served, staff views on involvement, formal (written) organization or system policies, explicit change to organization or system process of decision-making, additional connections or partnerships with other groups or organizations, funding and resources availability, visibility of organization) - Influence on population health, influence on population health generally (level of health inequalities, population health status).   Aggregate outcome:   - Overall cost-effectiveness of involvement from the standpoint of the healthcare organization or system. | - Does not report outcomes associated with PPI. |
| *Setting* | |
| Any type of health or social care setting, with no geographical restrictions, in which services are provided in institutional, or community contexts aiming to promote and protect among individuals, i.e., physical, mental, and social well-being outcomes | - Education outcomes - Economic outcomes - Environmental outcomes |
| *Study design* | |
| - Case study - Expert panel (e.g., Delphi study) - Experimental study (trial) - Qualitative study (e.g., interviews, focus groups) - Mixed methods - Observational study (cohort, case-control, and cross-sectional) | - Withdrawn publications - Project protocols - Reviews |
| *Other types of source* | |
| - Guide or framework - Report - Tool | Does not report the method by which the guide, framework, report, or tool was created.   - Editorial material (e.g. blogs, interviews, letters, newsletters) - Non-authored records (anonymous) - Non-traceable appendices or parts of documents - Videos and audio records - Presentations (e.g., slides) - Opinion texts |
| *Language* | |
| Any language | Not applicable |
| *Data of publication* | |
| Any data of publication | Not applicable |
