# Supplementary material for: Strategies for involving patients and the public in scaling initiatives in health and social services: A scoping review
Source: Health Expect. 2024 Jun 5;27(3):e14086. doi: 10.1111/hex.14086 (PMC11150745; doi:10.1111/hex.14086)
Supplement: Supplementary file 6 — Supporting information. [file HEX-27-e14086-s006.docx]

**Additional File 6 – Data extraction tool variables, codes and definitions**

| **Variable Extracted** | **Codes** | **Definitions** |
| --- | --- | --- |
| ***Bibliographic characteristics*** | | |
| Journal name | - Open-text | Name of the journal in which the article was published. |
| Title | - Open-text | Title of the article. |
| Authors | - Open-text | Authors of the article. |
| Corresponding author | - Open-text | Author to whom an e-mail is provided. |
| Corresponding author author contact | - Open-text | E-mail provided to contact the corresponding author |
| Year of publication | - Open-text | Year that the article was published. |
| Language | - Open-text | Language in which the article was published. |
| Aim of the study | - Open-text | Aim of the study as described by authors. |
| Study design | - Case study - Experimental study (trial) - Qualitative study - Mixed methods - Observational study (cohort, case-control, and cross-sectional) | Methods used in the study. |
| Other types of source | - Tool - Report - Guide or Framework | Records with the aim to present a tool, report* or guide/framework based on previous experience and/or literature but which does not report systematic study design.  *We considered as a “report” a document declared as such by the authors or that describe an initiative that took place without using a study design. |
| Country of corresponding author | - Open-text | We considered it as the country in which the study was led. |
| Funding | - Open-text | Funding declared by the authors for the study. |
| ***Scaling initiatives*** | | |
| Title of scaling initiative | - Open-text | Title of the initiative being scaled as described by the authors. |
| Description of the scaling initiative | - Open-text | Description of the initiative being scaled as stated by the authors. |
| Country in which the scaling was conducted | - Open-text | In the scope of the study, this means where the participants were recruited, where the data was collected, and the country in which the results are relevant. |
| Income levels of targeted country | - Higher income countries (HICs) - Lower-middle income and low-income countries (LMICs) | We based our classification on a World Bank Group^[[1]](#endnote-1)^.  We considered HICs as high-income and upper-middle-income countries; LMICs, as lowe-middle income and low-income countries; and not specified when authors did not target a country. |
| Developers of the scaling initiative | - Patients or public - Providers - Researchers - Policy makers (e.g., governments, ministries, WHO, etc.) - Other: specify (e.g., For-profit corporation) | Individuals, groups or institutions in charge of the scaling conception and development. |
| Targeted level of care | - Community-based primary health care (CBPHC) - Secondary care - Tertiary care | We considered:  Community-based primary health care (CBPHC) as covering the broad range of primary prevention services (including public health) and primary care services within the community, including health promotion and disease prevention; the diagnosis, treatment, and management of chronic and episodic illness; rehabilitation support; and end of life care. CBPHC involves the provision care by a range of health providers, including nurses, social workers, pharmacists, dietitians, public health practitioners, physicians and others in a range of community settings including people's homes, healthcare clinics, physicians' offices, public health units, hospices, and workplaces. We also considered social care services as CBPHC (e.g., initiatives aiming poverty reduction, prevent violence against children)^[[2]](#endnote-2)^.  Secondary care as specialized diagnostic and treatment services, involving specialist consultations, routine surgeries, and more complex medical services.  Tertiary care as highly specialized and advanced medical care provided by specialized hospitals or medical centers.  Social care: We consider social care to be a range of non-medical support services provided to vulnerable groups who need help to achieve well-being and social integration. |
| Health and social issues addressed by the scaling initiative | - Open-text | We consider health and social issues targeted by the scaling.  Health issues refer to a range of medical conditions addressed by health-related services and health care practitioners.  Social issues refer to socials condition of individuals and groups, related to initiatives that assist frail or vulnerable individuals by addressing their needs or empowering them to address needs arising from physical, mental, or emotional impairment^[[3]](#endnote-3)^ ^[[4]](#endnote-4)^. |
| Targeted setting | - Community - Health care system - Not reported - Other: specify (e.g., Workplace) | The setting where the initiative was scaled. |
| Sex- or gender-sensitive scaling initiative | - Yes - No | A scaling initiative is considered sensitive to sex and gender when it meets either of the following criteria: a) it focuses on particular groups that are vulnerable due to their gender and sex-based positions in society; or b) it incorporates specific measures to prevent the exacerbation of gender and sex-based inequalities (for example, by making additional efforts to reach LGBTQ+ groups). |
| ***PPI strategies*** | | |
| Involvement Strategy | Direct care level:   - Patient and public education - Behavioural change interventions - Personalized care planning - Self-management supports - Shared decision making - Access to health records or portals - Patient and public navigation - Family supports - Peer supports   Organization of healthcare:   - Information campaigns and platforms - Service user needs assessment - Quality and safety assurance - Organizational advisory groups - Co-leadership in quality and safety improvement   Professional training level:   - Use of patient and public data in training - Testimonials - Simulated patients and users - Patients and public as trainers - Co-design of educational or training activities   Research level:   - Lay scientific communications - Patient and public consultations - Involvement in study phases - Research advisory groups - Co-leadership in research activities   Policymaking:   - Population consultation - Policy advisory groups - Co-leadership in policymaking - Other: specify   *Non-mutually exclusive (multi-label) classes | We considered a PPI strategy as an approach to PPI in scaling whose goal is to enable patients and the public to receive information, advise, collaborate in or co-construct the scaling process. The level of the health and social care system in which the scaling occurs will determine the type of involvement strategy deployed. See Additional File 7 for Scaling involvement levels and strategy definitions^[[5]](#endnote-5)^. |
| Involvement Method | Open-text | The involvement method refers to a set of actions used to deploy the involvement strategy (e.g., board committees, conferences, focus groups, social media posts). |
| *Continuum* of patient and public involvement | - Information - Consultation - Collaboration - Coproduction | The involvement of patients and the public may be in different degrees: a) information, i.e. patients or the public receive information but have no role in contributing to scaling; b) consultation, when they provide their views, thoughts, feedback, opinions, or experiences but without a commitment to act on them; c) collaboration, when they participate in commenting, advising, ranking, voting, prioritizing, and reaching consensus on scaling, but without direct control over decisions; and d) coproduction, when they are considered equal members of the scaling team^[[6]](#endnote-6)^ ^[[7]](#endnote-7)^. |
| ***Characteristics of PPI strategies*** | | |
| Scaling phase where the involvement occurs | - Planning - Implementing - Evaluating   *Non-mutually exclusive (multi-label) classes | This corresponds to the phase of the scaling in which patients and the public were involved: a) when planning the scaling; b) when scaling; c) when evaluating the scaling (post)^[[8]](#endnote-8)^. |
| Ethical lenses in the rationale for PPI | - Teleological - Consequentialist- utilitarianism - Patient and public values - Deontological - Duty- Right      - Mixed ethical lenses | 1. Teleological (goal-oriented)  1.1 Consequentialist-utilitarian, i.e., when PPI is goal-oriented to increase scaling effectiveness;  1.2. Patient and public values, when PPI is goal-oriented to consider patient and public values in the scaling, i.e., when patients’ or the public's knowledge and background are considered fundamental and the aim of involving them is bringing that knowledge to the scaling;    2. Deontological (must do)  2.2 Duty-right, when PPI is considered a principle that guides scaling, i.e., PPI is either a duty to the patients or a right they deserve.  3. Mixed ethical lenses, PPI is based on either teleological and deontological lenses |
| Incentivization to involve patients and the public | - Open-text | These correspond to the incentivization offered to the public and patients to stimulate their involvement in scaling. This can be financial compensation, such as salaries and gift cards, but also non-material rewards, such as authorship. |
| Patient and public recruitment process | - Open-text | This describes the actions undertaken to recruit patients and the public to be involved in scaling. Example: media campaigns or direct invitation through community-based organizations. |
| Patient and public profile | \| - Targeted groups and/or beneficiary populations (e.g., women, male partners, parents, elderly people, vulnerable groups) \|  \| \| --- \| --- \| \| - Community (e.g., local organizations, community-based organizations, community members) \|  \| \| - Civil society organizations (e.g., NGOs, third sector organizations, trade unions). \|  \| \| - Volunteers (any individual described as volunteer) \|  \| \| - Patients (e.g., patients, patient's family, representatives or proxies) \|  \| \| - Leaders (e.g., religious leaders, opinion leaders, community leaders) \|  \| \| - Users (e.g., service users, clients) \|  \| \| - Citizens (any individual described as citizen) - Public (any group described as public \|  \| | How the authors name and/or describe the groups and individuals engaged in scaling. |
| Sex- or gender-sensitive PPI strategy | - Yes   - Specify - No | A sex and gender sensitive strategy is when additional efforts were deployed to recruit and engage groups that are vulnerable due to their gender and sex-based positions in society. |
| Involvement framework or guide | - Yes   - Specify - Not reported | It states whether a guide or model for patient and public involvement was used in scaling and specifies it. |
| Other stakeholders involved | - Providers (e.g., individuals and organizations that provide care to patients and populations such as nurses, physicians, pharmacists, mental health counselors, and community-based workers); - Funders (e.g., employers, governments, and other entities responsible for underwriting the cost of care); - Policy makers (e.g., policymaking entities such as governments and professional associations); - Product makers (e.g., drug and device manufacturers), - Researchers (e.g., academic institutions, postdoctoral fellows); and - The press (e.g., publisher and news media)   **Non-mutually exclusive (multi-label) classes* | It describes the other stakeholders that were involved in the scaling initiative and the strategies used to involve them. |
| Involvement Outcomes | - Involved participants - Services provided - Organization or system - Broader public - Population health - Overall cost-effectiveness of involvement   **Non-mutually exclusive (multi-label) classes* | We considered^[[9]](#endnote-9)^:  Impact on the involved participants themselves (knowledge, skills, empowerment, satisfaction, trust)  Impact on the services provided by the organization or system (efficiency and cost-effectiveness of services, service availability, services quality and safety, services responsiveness to needs, utilization of services)  Impact on the organization or system itself (awareness or knowledge of health issues, support of the organization or system)  Influence on the broader public (accountability of organization to patient and public served, staff views on involvement, formal (written) organization or system policies, explicit change to organization or system process of decision-making, additional connections or partnerships with other groups or organizations, funding and resources availability, visibility of organization)  Influence on population health, influence on population health generally (level of health inequalities, population health status).  Overall cost-effectiveness of involvement from the standpoint of the healthcare organization or system; could consider all cost changes (positive or negative) throughout all subdomains. |
| PROGRESS-Plus framework | - Place of residence   - Not reported   - Rural   - Urban   - Lower income district - Race/ethnicity/culture   - Not reported   - Yes - specify - Occupation   - Not reported   - Yes - specify - Gender   - Not reported   - Woman   - Men   - Other - specify - Sex at birth   - Not reported   - female   - male - Religion   - Not reported   - Yes - specify - Education   - Not reported   - Low   - Middle   - High - Socioeconomic status   - Not reported   - Yes - specify - Social capital and networks - Age   - Not reported   - Children (00-14 years)   - Youth (15-24 years)   - Adults (25-64 years)   - Seniors (65 years and over)   - Other - specify - Disability   - Not reported   - Yes - specify - Sexual orientation   - Not reported   - Heterosexual   - Gay   - Lesbian   - Bisexual   - Other – specify | PROGRESS-Plus is an acronym used to identify characteristics that stratify health opportunities and outcomes^[[10]](#endnote-10)^. |

1. References:

   World Bank. World Bank Country and Lending Groups [Internet]. The World Bank. 2024. Available from: <https://datahelpdesk.worldbank.org/knowledgebase/articles/906519-world-bank-country-and-lending-groups> [↑](#endnote-ref-1)
2. [Community-Based Primary Health Care - CIHR (cihr-irsc.gc.ca)](https://cihr-irsc.gc.ca/e/43626.html) [↑](#endnote-ref-2)
3. Evans D. The provision of health and social care services for older people by respite providers. Contemp Nurse. 2013;45(2):255-263. https://doi.org/10.5172/conu.2013.45.2.255doi:10.5172/conu.2013.45.2.255. [↑](#endnote-ref-3)
4. Burge P, Netten A, Gallo F. Estimating the value of social care. J Health Econ. 2010;29(6):883-894. https://doi.org/10.1016/j.jhealeco.2010.08.006doi:10.1016/j.jhealeco.2010.08.006. [↑](#endnote-ref-4)
5. Menear M, Dugas M, Careau E, Chouinard MC, Dogba MJ, Gagnon MP, et al. Strategies for engaging patients and families in collaborative care programs for depression and anxiety disorders: A systematic review. Journal of Affective Disorders. 2020 Feb;263(1):528–39. [↑](#endnote-ref-5)
6. Carman KL, Dardess P, Maurer M, Sofaer S, Adams K, Bechtel C, et al. Patient and family involvement: a framework for understanding the elements and

   developing initiatives and policies. Health Aff (Millwood). 2013;32:223–31. [↑](#endnote-ref-6)
7. Pomey M-P, Flora L, Karazivan P, Dumez V, Lebel P, Vanier M-C, et al. The Montreal model: the challenges of a partnership relationship between patients and healthcare professionals. Sante Publique. 2015;27:S41–50. [↑](#endnote-ref-7)
8. Milat, A. J., Newson, R., King, L., Rissel, C., Wolfenden, L., Bauman, A., Redman, S., & Giffin, M. (2016). A guide to scaling up population health interventions. Public health research & practice, 26(1), e2611604. <https://doi.org/10.17061/phrp261160> [↑](#endnote-ref-8)
9. Concannon TW, Meissner P, Grunbaum JA, McElwee N, Guise J-M, Santa J, et al. A new taxonomy for stakeholder engagement in patient-centered outcomes research. J Gen Intern Med. 2012;27:985–91. [↑](#endnote-ref-9)
10. O'Neill J, Tabish H, Welch V, Petticrew M, Pottie K, Clarke M, Evans T, Pardo Pardo J, Waters E, White H, Tugwell P. Applying an equity lens to interventions: using PROGRESS ensures consideration of socially stratifying factors to illuminate inequities in health. Journal of Clinical Epidemiology. 2014, 67 (1), pg. 56-64. doi:10.1016/j.jclinepi.2013.08.005 [↑](#endnote-ref-10)
