# Supplementary material for: Strategies for involving patients and the public in scaling initiatives in health and social services: A scoping review
Source: Health Expect. 2024 Jun 5;27(3):e14086. doi: 10.1111/hex.14086 (PMC11150745; doi:10.1111/hex.14086)
Supplement: Supplementary file 7 — Supporting information. [file HEX-27-e14086-s013.docx]

**Additional File 7 – PPI strategies: levels and definitions**

We considered a PPI strategy as an approach to sustaining patient and public involvement (PPI) in scaling initiatives that may be occurring in scaling science or practice. The goal of a PPI strategy is to enable patients and the public to receive information, advice, collaborate in, and co-construct the scaling of an intervention. Strategies require methods, i.e., a way of enacting this approach. For example, the strategy may be to involve patients and the public in a policy advisory group, and the corresponding method would be to form a steering committee or hold a workshop with patients and policymakers. The health and social care system levels at which the scaling occurs determine the type of involvement strategy deployed. Based on Menear et al. 2020,^[[1]](#endnote-1)^ we considered the following scaling involvement levels and strategies:

**1. Direct care level**: When scaling initiatives occur at the direct care level, patients and the public are directly involved in interactions with professionals and teams in the context of the delivery of care.

| **Involvement strategy** | | **Definition** | **Methods extracted from the scoping review** |
| --- | --- | --- | --- |
| 1.1. | Patient and public education | Involving patients and the public through the use of informational and educational resources to help increase their knowledge about a specific topic. | e.g., Organizing workshops for knowledge building; training people to deliver prevention messages and tools. |
| 1.2 | Access to health records or portals | Involving patients and the public through the improvement of their access to electronic records or online platforms that enable them to communicate with health and social care services. | e.g., patients are encouraged to access their health data through their personal health records. |
| 1.3 | Behavioral change interventions | Involving patients and the public through actions aimed at increasing their motivation, readiness or commitment to change regarding a specific topic. | e.g., involvement of community leaders; comprehensive social and behavioral communications approach, involving mass media and facility- and community-level providers. |
| 1.4 | Patient and public navigation | Involving patients and the public through actions aimed at helping them navigate through health and social services. | e.g., developing a treatment education toolkit. |
| 1.5 | Personalized care planning | Involving patients and the public through anticipatory and negotiated care plans, made with one or more professionals in social and health services. | e.g., discussions on advance care planning and completing a personal directive if desired. |
| 1.6 | Self-management supports | Involving patients and the public through the use of individual or group supports to improve their ability to manage their own health and social conditions. | e.g., providing tools such as books, manuals on skills building, problem solving and positive living. |
| 1.7 | Shared decision making | Involving patients and the public through shared decision making, in which ~~health~~ decisions are made through an interactive process ~~between patients and health professionals~~ that takes into consideration the best available evidence, as well as the patients’ values and preferences. | e.g., in a clinical setting, healthcare teams support patients to make decisions about whether to participate in a scaling project that could potentially improve their well-being. |
| 1.8 | Family supports | Involving patients and the public through actions aiming to inform, consult, support or partner with family members. | e.g., peers come together in a multiple family group structure andprovide culturally acceptable community-level resources for one another. |
| 1.9 | Peer supports | Involving patients and the public through peer support activities, i.e., exchanging experiences with others who have similar backgrounds or challenges. | e.g., involving user groups who release and spread the innovation to introduce it into new user groups within their peer networks; participatory women’s groups. |

**2. Organization of health and social care:** The scaling initiatives may occur at the level of the organization of health and social care, where patients and the public might be involved in planning, governance, improvement, or assessment of programs or services in an organization or system.

| **Involvement strategy** | | | | **Definition** | | **Methods extracted from the scoping review** | |
| --- | --- | --- | --- | --- | --- | --- | --- |
| 2.1 | | Information campaigns and platforms | | Involving patients and the public through communication strategies or platforms designed to raise awareness about a health or social system care topic. | | e.g., mobilizing grassroots campaigns, social marketing, and public relations campaigns targeting stakeholders; policy briefs, engaging the support of opinion leaders and champions to act as spokespersons for scaling up the intervention. | |
| 2.2 | | Service user needs assessment | | Involving patients and the public through systematic assessment of their needs to inform priorities and resources that will help improve the organization of health and social care. | | e.g., focus groups and client surveys; consulting with volunteers. | |
| 2.3 | | Quality and safety assessment | | Involving patients and the public through assessment activities and programs intended to assure or improve the quality or safety of health and social care. | | e.g., collecting patients’ (and carers’) opinions while they are accessing a service; involving patients and the public in detailing safety specifications and assessing equipment; inclusion of patient/carer involvement surveys in post-implementation questionnaires and interviews with stakeholders. | |
| 2.4 | | Organizational advisory groups | | Involving patients and the public in councils or committees established to provide information, advice or guidance on organization of health and social care. | | e.g., steering committee; participatory meetings and events, with the use of the expertise of each partner in the ecosystem to analyze local needs. | |
| 2.5 | | Co-leadership in quality and safety improvement | | Involving patients and the public through shared leadership and responsibility for initiatives aiming to improve the quality and safety in the context of the organization of health and social care. | | Patient chair of a Patient Participation Group (PPG); Actively including community members in Quality Improvement (QI) teams. | |

**3.** **Professional training level:** The scaling initiatives may occur at the professional training level, where patients and the public might participate in the design or delivery of training for professionals, either during their initial professional development or as part of their continuing education.

| **Involvement strategy** | | **Definition** | **Methods extracted from the scoping review** |
| --- | --- | --- | --- |
| 3.1 | Use of patients and the public data in training | Involving patients and the public through data collected directly from them on their characteristics, experiences or perspectives for the purpose of inclusion in professional education or training activities. | Not found. |
| 3.2 | Testimonials | Involving patients and the public through oral, written or video communications (e.g. stories, diaries) prepared by individuals with lived experience intended to help professionals gain insight into their experiences. | Not found. |
| 3.3 | Simulated patients and users | Involving patients and the public through simulation activities in which actors, either lay or with lived experience, receive training to take on the role of patients and users to facilitate learning and help health and social care professionals examine and role play scenarios for training purposes. | Not found. |
| 3.4 | Patients and public as trainers | Involving patients and the public as formal members of the educational or training team to teach about their lived experience. | e.g., train-the-trainer workshops. |
| 3.5 | Co-design of educational or training activities | Involving patients and the public through shared leadership and responsibility for educational or training initiatives. | e.g., creation of training materials containing advice on how the innovation can be used for use in workshops, websites and newsletters; video vignettes developed by the partnership and focused on lived experiences rewritten and produced for broad use within the community. |

**4. Research level:** The scaling initiatives may occur at the research level, where patients and the public might be involved in research on or for scaling.

| **Involvement strategy** | | **Definition** | **Methods extracted from the scoping review** |
| --- | --- | --- | --- |
| 4.1 | Lay scientific communications | Involving patients and the public through the preparation and dissemination of research-related communications to a lay audience. | Not found. |
| 4.2 | Patient and public consultations | Involving patients and the public through consulting them to gather their input on priorities and scaling research methods as well as on research findings and dissemination strategies. | e.g., surveys; consultation workshops. |
| 4.3 | Involvement in study phases | Involving patients and the public through their active collaboration in activities in one or more phases of the scaling research (e.g., identifying evidence, defining problems and setting objectives, conducting participant recruitment and data collection etc.). | e.g., participatory action research; participatory project planning and validation of protocol. |
| 4.4 | Research advisory groups | Involving patients and the public in councils or committees established to provide information, advice or guidance on scaling research. | Community advisory board; Establishment of governance structures with a representative steering committee or decision-making board, with a regular  schedule of meetings, and mechanisms for transparency and accountability. |
| 4.5 | Co-leadership in research activities | Involving patients and the public through shared leadership and responsibility for all phases of the scaling research. | e.g., analyzing and assessing the research process and through all steps. |

**5. Policymaking:** The scaling initiatives may occur at the policymaking level, where patients and the public participate in developing, implementing, and evaluating system-level scaling policies and priorities.

| **Involvement strategy** | | **Definition** | **Methods extracted from the scoping review** |
| --- | --- | --- | --- |
| 5.1 | Population consultations | Involving patients and the public through policymakers gathering input from them on system-level scaling policies and priorities. | e.g., field visits and interviews to understand the local context and local perspectives. |
| 5.2 | Policy advisory groups | Involving patients and the public in councils or committees established to provide information, advice or guidance on system-level scaling policies and priorities | e.g., maintenance and monitoring of interventions by a core group of committed stakeholders, including community advocates, public health officials, practitioners, and scientists; participatory assemblies to review the different stages of the intervention. |
| 5.3 | Co-leadership in policymaking | Involving patients and the public through shared leadership and responsibility in developing, implementing and evaluating system-level scaling policies and priorities | e.g., participating in the decision-making process on national and sub-national levels; community steering committees included in the decision-making processes of the project before it starts |

1. Menear M, Dugas M, Careau E, Chouinard MC, Dogba MJ, Gagnon MP, et al. Strategies for engaging patients and families in collaborative care programs for depression and anxiety disorders: A systematic review. Journal of Affective Disorders. 2020 Feb;263(1):528–39. [↑](#endnote-ref-1)
