# Supplementary material for: Strategies for involving patients and the public in scaling initiatives in health and social services: A scoping review
Source: Health Expect. 2024 Jun 5;27(3):e14086. doi: 10.1111/hex.14086 (PMC11150745; doi:10.1111/hex.14086)
Supplement: Supplementary file 8 — Supporting information. [file HEX-27-e14086-s009.pdf]

Patient and public involvement in scaling in HSS Corôa et al. 2024
Additional File 8 – Included Reports

Title	Authors	Published Year	Journal	DOI
Government Ownership and Adaptation in Scale-Up: Experiences from Community-Based Family Planning Programme in the Democratic Republic of the Congo	Mai, M.; Hassen, E.; Ntabona, A. B.; Bapura, J.; Sarathy, M.; Yodi, R.; Mujani, Z.	2019	Afr J Reprod Health	https://dx.doi.org/10.29063/ajrh2019/v23i4.5
The diffusion of a community-level HIV intervention for women: lessons learned and best practices	King, W.; Nu'Man, J.; Fuller, T. R.; Brown, M.; Smith, S.; Howell, A. V.; Little, S.; Patrick, P.; Glover, L.	2008	J Womens Health (Larchmt)	https://dx.doi.org/10.1089/jwh.2008.1035
Expanding Safer Sex Options: Introducing the Female Condom into National Programmes	Warren, Mitchell; Philpott, Anne	2003	Reproductive Health Matters	
Scaling Up Global Health Interventions: A Proposed Framework for Success	Yamey, Gavin	2011	PLoS Medicine	10.1371/journal.pmed.1001049
Pathways towards scaling up Problem Management Plus in Turkey: a theory of change workshop	Fuhr, Daniela C.; Acarturk, Ceren; Uygun, Ersin; McGrath, Michael; Ilkkursun, Zeynep; Kaykha, Sadaf; Sondorp, Egbert; Sijbrandij, Marit; Ventevogel, Peter; Cuijpers, Pim; Roberts, Bayard	2020	Conflict & Health	10.1186/s13031-020-00278-w
Scaling up breastfeeding policy and programs in Samoa: application of the Becoming Breastfeeding Friendly initiative	Soti-Ulberg, Christina; Hromi-Fiedler, Amber; Hawley, Nicola L.; Naseri, Take; Manuele-Magele, Analosa; Ah-Ching, John; Pérez-Escamilla, Rafael; on behalf of, B. B. F. Samoa Committee; Sanele, Maria; Maualaivao, Namulauulu Tautala; Faaleaga, Visesio; Aiolupotea, Saunimaa; Abraham, Jyothi Alex; Tuautu, Sina; Tamati, Cedrela; Lesatele, Patricia; Harris, Naomi; Moala, Samasoni; Leleimalefaga, Siaosi U.; Faleupolu, Lusi	2020	International Breastfeeding Journal	10.1186/s13006-019-0245-6
Key factors for national spread and scale-up of an eConsult innovation	Moroz, I.; Archibald, D.; Breton, M.; Cote-Boileau, E.; Crowe, L.; Horsley, T.; Hyseni, L.; Johar, G.; Keely, E.; Burns, K. K.; Kuziemsky, C.; Laplante, J.; Mihan, A.; Oppenheimer, L.; Sturge, D.; Tuot, D. S.; Liddy, C.	2020	Health Res Policy Syst	https://dx.doi.org/10.1186/s12961-020-00574-0
Implementation and scale up of population physical activity interventions for clinical and community settings: the PRACTIS guide	Koorts, H.; Eakin, E.; Estabrooks, P.; Timperio, A.; Salmon, J.; Bauman, A.	2018	Int	https://dx.doi.org/10.1186/s12966-018-0678-0
Strengthening scaling up through learning from implementation: comparing experiences from Afghanistan, Bangladesh and Uganda	Bennett, S.; Mahmood, S. S.; Edward, A.; Tetui, M.; Ekirapa-Kiracho, E.	2017	Health Res Policy Syst	https://dx.doi.org/10.1186/s12961-017-0270-0
A model for scale up of family health innovations in low-income and middle-income settings: a mixed methods study	Bradley, E. H.; Curry, L. A.; Taylor, L. A.; Pallas, S. W.; Talbert-Slagle, K.; Yuan, C.; Fox, A.; Minhas, D.; Ciccone, D. K.; Berg, D.; Perez-Escamilla, R.	2012	BMJ Open	https://dx.doi.org/10.1136/bmjopen-2012-000987
Overview of a multi-stakeholder dialogue around Shared Services for Health: the Digital Health Opportunity in Bangladesh	Ashraf, S.; Moore, C.; Gupta, V.; Chowdhury, A.; Azad, A. K.; Singh, N.; Hagan, D.; Labrique, A. B.	2015	Health Res Policy Syst	https://dx.doi.org/10.1186/s12961-015-0063-2
The role of patients and carers in diffusing a health-care innovation: A case study of "My Medication Passport"	Barber, S.; French, C.; Matthews, R.; Lovett, D.; Rollinson, T.; Husson, F.; Turley, M.; Reed, J.	2019	Health Expect	https://dx.doi.org/10.1111/hex.12893
Evidence-based adaptation and scale-up of a mobile phone health information service	L'Engle, K.; Plourde, K. F.; Zan, T.	2017	Mhealth	https://dx.doi.org/10.21037/mhealth.2017.02.06
Dissonances and disconnects: the life and times of community based accountability in the national rural health mission in Tamilnadu, India	Gaitonde, R.; San Sebastian, M.; Hurtig, A. K.	2020	BMC Health Serv Res	https://dx.doi.org/10.1186/s12913-020-4917-0
Scaling up interventions for depression in sub-Saharan Africa: lessons from Zimbabwe	Chibanda, D.; Verhey, R.; Munetsi, E.; Rusakaniko, S.; Cowan, F.; Lund, C.	2016	Glob Ment Health (Camb)	https://dx.doi.org/10.1017/gmh.2016.8
Stakeholder Engagement in the Translation of a Hypertension Control Program to Guatemala's Public Primary Health Care System: Lessons Learned, Challenges, and Opportunities	Fort, M. P.; Paniagua-Avila, A.; Beratarrechea, A.; Cardona, S.; Figueroa, J. C.; Martinez-Folgar, K.; Moyano, D.; Barrios, E.; Mazariegos, B. E.; Palacios, E.; Irazola, V.; He, J.; Ramirez-Zea, M.	2019	Glob Heart	https://dx.doi.org/10.1016/j.gheart.2019.05.005
Dissemination and implementation of an educational tool for veterans on complementary and alternative medicine: a case study	Held, Rachel Forster; Santos, Susan; Marki, Michelle; Helmer, Drew	2016	BMC Complementary & Alternative Medicine	10.1186/s12906-016-1297-4
Factors facilitating and constraining the scaling up of an evidence-based strategy of community-based primary care: management perspectives from northern Ghana	Krumholz, A. R.; Stone, A. E.; Dalaba, M. A.; Phillips, J. F.; Adongo, P. B.	2015	Glob Public Health	https://dx.doi.org/10.1080/17441692.2014.981831
Lessons learned from scaling up a community-based health program in the Upper East Region of northern Ghana	Awoonor-Williams, J. K.; Sory, E. K.; Nyonator, F. K.; Phillips, J. F.; Wang, C.; Schmitt, M. L.	2013	Glob	https://dx.doi.org/10.9745/GHSP-D-12-00012
Adapting and disseminating a community-collaborative, evidence-based HIV/AIDS prevention programme: Lessons from the history of CHAMP	Sperber, Elizabeth; McKay, Mary M.; Bell, Carl C.; Petersen, Inge; Bhana, Arvin; Paikoff, Roberta	2008	Vulnerable Children and Youth Studies	http://dx.doi.org/10.1080/17450120701867561
Community-driven demand creation for the use of routine viral load testing: a model to scale up routine viral load testing	Killingo, B. M.; Taro, T. B.; Mosime, W. N.	2017	J Int AIDS Soc	https://dx.doi.org/10.1002/jia2.25009
Harnessing "Scale-Up and Spread" to Support Community Uptake of the HoMBReS por un Cambio Intervention for Spanish-Speaking Men: Implementation Science Lessons Learned by a CBPR Partnership	Rhodes, S. D.; Mann-Jackson, L.; Alonzo, J.; Nall, J.; Siman, F. M.; Song, E. Y.; Garcia, M.; Tanner, A. E.; Eng, E.	2020	Am j	https://dx.doi.org/10.1177/1557988320938939
Reducing the treatment gap for mental, neurological and substance use disorders in Africa: lessons from the Friendship Bench in Zimbabwe	Chibanda, D.	2017	Epidemiol Psychiatr Sci	https://dx.doi.org/10.1017/S2045796016001128
The costs of scaling up HIV prevention for high risk groups: lessons learned from the Avahan Programme in India	Chandrashekar, S.; Guinness, L.; Pickles, M.; Shetty, G. Y.; Alary, M.; Vickerman, P.; Group, Charme-Evaluation; Vassall, A.	2014	PLoS ONE	https://dx.doi.org/10.1371/journal.pone.0106582
From trial to population: A study of a family-based community intervention for childhood overweight implemented at scale	Fagg, J.; Chadwick, P.; Cole, T.; Cummins, S.; Goldstein, H.; Lewis, H.; Morris, S.; Radley, D.; Sacher, P.; Law, C.	2014	International Journal of Obesity	http://dx.doi.org/10.1038/ijo.2014.103
Stakeholders' participation in operational research on HIV care: insights from Burkina Faso	Desclaux, A.; Kouanda, S.; Obermeyer, C. M.	2010	Aids	https://dx.doi.org/10.1097/01.aids.0000366086.21687.52
A community-based partnership to promote exercise among cancer survivors: lessons learned	Pinto, B. M.; Waldemore, M.; Rosen, R.	2015	Int J Behav Med	https://dx.doi.org/10.1007/s12529-014-9395-5
Beginning with sustainable scale up in mind: initial results from a population, health and environment project in East Africa	Ghiron, L.; Shillingi, L.; Kabiswa, C.; Ogonda, G.; Omimo, A.; Ntabona, A.; Simmons, R.; Fajans, P.	2014	Reprod Health Matters	https://dx.doi.org/10.1016/S0968-8080(14)43761-3
Changing Social Norms: the Importance of "Organized Diffusion" for Scaling Up Community Health Promotion and Women Empowerment Interventions	Cislaghi, B.; Denny, E. K.; Cisse, M.; Gueye, P.; Shrestha, B.; Shrestha, P. N.; Ferguson, G.; Hughes, C.; Clark, C. J.	2019	Prev Sci	https://dx.doi.org/10.1007/s11121-019-00998-3
Interventions in organizational and community context: a framework for building evidence on dissemination and implementation in health services research	Mendel, P.; Meredith, L. S.; Schoenbaum, M.; Sherbourne, C. D.; Wells, K. B.	2008	Adm Policy Ment Health	
Expanding access to primary healthcare for women through a microfinance institution: A case study from rural Guatemala	Colom, M.; Austad, K.; Sacuj, N.; Larson, K.; Rohloff, P.	2018	Healthc (Amst)	https://dx.doi.org/10.1016/j.hjdsi.2017.12.003
Factors that influence the scale up of new interventions in low-income settings: a qualitative case study of the introduction of chlorhexidine cleansing of the umbilical cord in Bangladesh	Callaghan-Koru, J. A.; Islam, M.; Khan, M.; Sowe, A.; Islam, J.; Mannan, II; George, J.; Bangladesh Chlorhexidine Scale Up Study, Group	2020	Health Policy Plan	https://dx.doi.org/10.1093/heapol/czz156
Effectiveness of scaling up the 'three pillars' approach to accelerating MDG 4 progress in Ethiopia	Carnell, M. A.; Dougherty, L.; Pomeroy, A. M.; Karim, A. M.; Mekonnen, Y. M.; Mulligan, B. E.	2014	J Health Popul Nutr	
Scaling Up of an Innovative Intervention to Reduce Risk of Dengue, Chikungunya, and Zika Transmission in Uruguay in the Framework of an Intersectoral Approach with and without Community Participation	Basso, C.; Garcia da Rosa, E.; Lairihoy, R.; Caffera, R. M.; Roche, I.; Gonzalez, C.; da Rosa, R.; Gularte, A.; Alfonso-Sierra, E.; Petzold, M.; Kroeger, A.; Sommerfeld, J.	2017	Am J Trop Med Hyg	https://dx.doi.org/10.4269/ajtmh.17-0061

Formative evaluation of antiretroviral therapy scale-up efficiency in sub-Saharan Africa	Wagner, G.; Ryan, G.; Taylor, S.	2007	AIDS Patient Care STDS	https://dx.doi.org/10.1089/apc.2007.0008
INSPIRED Scale Collaborative	CFHI-4	2021		
REPORT OF THE BETTER TOGETHER POLICY ROUNDTABLE	CFHI-86			
Evaluation of the Huddling Up for Safer Healthcare (HUSH) Scaling Up Project	Google-18	2018		
Strategies for Scaling Up: Promoting Parent Involvement through Family-School-Community Partnerships	Google-28	2016		
Guidance Note. Scaling Up Development Programmes	Google-29	2013		
Citizen workshops in public libraries to disseminate and discuss primary care research results: a scaling-up study	Google-40; Massougbodji, J; Zomahoun, HTV			
Scaling Up—From Vision to Large-Scale Change, 3eEdition	Google-57	2016		
THE JOURNEY TO SCALE. Moving together past digital health pilots	Google-62	2014		
SISCODe co-design for society in innovation and science	Google-180	2020		
Scaling: Where to start?	Google-182	2020		
European Scaling-up Strategy in Active and Healthy Ageing	Google-190			
Implementation, Spread and Impact of the Patient Oriented Discharge Summary (Pods) Across Ontario Hospitals: A Mixed Methods Evaluation	Google-202; Hahn-Goldberg, S; Huynh, T.; Chaput, A.			
Leveraging an Implementation Science Framework to Measure the Impact of Efforts to Scale Out a Total Worker Health® Intervention to Employers	Google-234; Tenney, L.; Huebschmann, A.G.			
COMMUNITY-FACILITY LINKAGES TO SUPPORT THE SCALE UP OF LIFELONG TREATMENT FOR PREGNANT AND BREASTFEEDING WOMEN LIVING WITH HIV	Google-381	2015		
Practical guidance for scaling up health service innovations	IHI-4; WHO-ExpandNet	2009		
Lessons learned from Ghana's Project Fives Alive! A pratical guide for designing and executing large-scale improvement initiatives	IHI-6	2015		
USAID Applying Science to Strengthen and Improve Systems (ASSIST) Project	IHI-12	2020		
Nurturing the seeds of change: Insights from scaling people-powered health and care innovations	NICE-9			
Male engagement in the HIV response —a Platform for Action	Nice-21			
ROADMAP TO IMPLEMENT THE TUBERCULOSIS ACTION PLAN FOR THE WHO EUROPEAN REGION 2016-2020	NICE-167			
Increasing the scale of population health interventions: A Guide	NSW-6			
Evidence-based scaling up of health and family planning service innovations in Bangladesh and Ghana	WHO-3; Phillips, J.F			
Scaling up inclusive approaches for marginalised and vulnerable people Becky	WHO-8; Carter, B			
The Scaling Playbook A Practical Guide for Researchers	WHO-9; Price-Kelly,; van Haeren; McLean	2020		
Scaling-up experimental project success with the Community-based Health Planning and Services initiative in Ghana.	WHO-189; Nyonator, FK.; Akosa, AB.; Awoonor-Williams, JK.; Phillips, JF.; Jones, TC.	2007		
A field-based and participatory approach to supporting the development of scaling-up strategies	WHO-34; ExpandNet	2011		
Scaling Up Community Driven Development Theoretical Underpinnings and Program Design Implications	WHO-11; Binswanger, HP,; Aiyar, SS.	2003		
'The development sector is a graveyard of pilot projects!' Six critical actions for externally funded implementers to foster scale-up of maternal and newborn health innovations in low and middle-income countries	WHO-414; Spicer, N.; Hamza, Y. A.; Berhanu, D.; Gautham, M.; Schellenberg, J.; Tadesse, F.; Umar, N.; Wickremasinghe, D.	2018	Global Health	10.1186/s12992-018-0389-y
INSPIRE Guide to Adaptation and Scale Up	WHO-120; Inspire working group	2021		
Every Newborn: health-systems bottlenecks and strategies to accelerate scale-up in countries	WHO-30; Dickson, K. E.; Simen-Kapeu, A.; Kinney, M. V.; Huicho, L.; Vesel, L.; Lackritz, E.; de Graft Johnson, J.; von Xylander, S.; Rafique, N.; Sylla, M.; Mwansambo, C.; Daelmans, B.; Lawn, J. E.; Lancet Every Newborn Study, Group	2014	Lancet	10.1016/S0140-6736(14)60582-1
Dissemination, diffusion and scale up of family health innovations in low-income countries	WHO-13; Bradley, E.H.; Curry, L.; Perez-Escamilla, R	2011		
Transitioning from a single-site pilot project to a state-wide regional telehealth service: The experience from the Victorian Stroke Telemedicine programme	WHO-553; Bagot, K. L.; Cadilhac, D. A.; Kim, J.; Vu, M.; Savage, M.; Bolitho, L.; Howlett, G.; Rabl, J.; Dewey, H. M.; Hand, P. J.; Denisenko, S.; Donnan, G. A.; Bladin, C. F.; Victorian Stroke Telemedicine Programme, Consortium	2017	Journal of Telemedicine & Telecare	https://dx.doi.org/10.1177/1357633X17734004
'Scaling-up is a craft not a science': Catalysing scale-up of health innovations in Ethiopia, India and Nigeria	WHO-92; Spicer, N.; Bhattacharya, D.; Dimka, R.; Fanta, F.; Mangham-Jefferies, L.; Schellenberg, J.; Tamire-Woldemariam, A.; Walt, G.; Wickremasinghe, D.	2014	Social Science & Medicine	https://dx.doi.org/10.1016/j.socscimed.2014.09.046
Eight Strategies for Research to Practice. Moving Evidence to Action	WHO-179; Canoutas, E.; Harts, L.; Zan, T	2012		
Implementation research in health: a practical	WHO-169; Peters, DH.			
The MAPS Toolkit: mHealth Assessment and Planning for Scale	World Health Organization	2015		
Scale-up of the DMPA-SC in Nigeria: Why policy matters	Akinyemi, Oluwaseun; Danfakha, Nicole; Adefalu, Adewole; Easley, Ebony; Afolabi, Kayode; Latunji, Olajimi	2022	BMC Women's Health	10.1186/s12905-022-02109-x
Stakeholder perceptions on scaling-up community-led interventions for prevention and control of non-communicable diseases in Bangladesh: a qualitative study	Akter, Kohenour; Kuddus, Abdul; Jeny, Tasnova; Nahar, Tasmin; Shaha, Sanjit; Ahmed, Naveed; King, Carina; Pires, Malini; Haghparast-Bidgoli, Hassan; Azad, Kishwar; Fottrell, Edward; Morrison, Joanna	2023	BMC Public Health	10.1186/s12889-023-15551-9
Family-Based Index testing for HIV; a qualitative study of acceptance, barriers/challenges and facilitators among clients in Cape Coast, Ghana	Asamoah Ampofo, Evelyn; Commey Tetteh, Isaac; Adu-Gyamfi, Raphael; Ebu Enyan, Nancy Innocentia; Agyare, Elizabeth; Ayisi Addo, Stephen; Obiri-Yeboah, Dorcas	2022	AIDS Care	10.1080/09540121.2021.1981818
Developing organizational learning for scaling-up community-based primary health care in Ghana	Awoonor-Williams, John Koku; Phillips, James F.	2022	Learning Health Systems	10.1002/lrh2.10282
Scaling-up digital follow-up care services: collaborative development and implementation of Remote Patient Monitoring pilot initiatives to increase access to follow-up care	Azevedo, S.; Guede-Fernández, F.; von Hafe, F.; Dias, P.; Lopes, I.; Cardoso, N.; Coelho, P.; Santos, J.; Fragata, J.; Vital, C.; Semedo, H.; Gualdino, A.; Londral, A.	2022	Frontiers in Digital Health	10.3389/fdgth.2022.1006447
National implementation of a pragmatic quality improvement skills curriculum for urology residents in the UK: Application and results of 'theory-of-change' methodology	Balayah, Z.; Khadjesari, Z.; Keohane, A.; To, W.; Green, J. S. A.; Sevdalis, N.	2021	American Journal of Surgery	10.1016/j.amjsurg.2020.12.007
Using Responsive Feedback in Scaling a Gender Norms-Shifting Adolescent Sexual and Reproductive Health Intervention in the Democratic Republic of Congo	Barker, K. M.; Gayles, J.; Diakité, M.; Diantisa, F. G.; Lundgren, R.	2023	Global health, science and practice	10.9745/GHSP-D-22-00208

Adapting a community pharmacy intervention to improve medication safety	Berbakov, M. E.; Hoffins, E. L.; Stone, J. A.; Gilson, A. M.; Chladek, J. S.; Watterson, T. L.; Lehnbom, E. C.; Moon, J.; Holden, R. J.; Jacobson, N.; Shiyanbola, O. O.; Welch, L. L.; Walker, K. D.; Gollhardt, J. D.; Chui, M. A.	2023	Journal of the American Pharmacists Association	10.1016/j.japh.2023.11.009
A nutrition and lifestyle-focused shared medical appointment in a resource-challenged community setting: a mixed-methods study	Bharmal, N.; Beidelschies, M.; Alejandro-Rodriguez, M.; Alejandro, K.; Guo, N.; Jones, T.; Bradley, E.	2022	BMC Public Health	10.1186/s12889-022-12833-6
Reproducibility and implementation of a rapid, community-based COVID-19 “test and respond” model in low-income, majority-Latino communities in Northern California	Chamie, G.; Prado, P.; Oviedo, Y.; Vizcaíno, T.; Arechiga, C.; Marson, K.; Carrera, O.; Alvarado, M. J.; Corchado, C. G.; Gomez, M.; Mochel, M.; de Leon, I.; Garibay, K. K.; Durazo, A.; De Trinidad Young, M. E.; Yen, I. H.; Sauceda, J.; Rojas, S.; DeRisi, J.; Petersen, M.; Havlir, D. V.; Marquez, C.	2022	PLoS ONE	10.1371/journal.pone.0276257
Lay social workers implementing a task-sharing approach to managing depression in Vietnam	Chau, Leena W.; Murphy, Jill; Nguyen, Vu Cong; Lou, Hayami; Khanh, Huyen; Thu, Trang; Minas, Harry; O'Neil, John	2021	International Journal of Mental Health Systems	10.1186/s13033-021-00478-8
Building sustainable and scalable peer-based programming: promising approaches from TESFA in Ethiopia	Chowdhary, Pari; Mekuria, Feven Tassaw; Tewahido, Dagmawit; Gulema, Hanna; Derni, Ryan; Edmeades, Jeffrey	2022	Reproductive Health	10.1186/s12978-021-01304-7
Development, Adaptation and Scale-up of a Community-wide, Health Behavior Theory-based Stroke Preparedness Intervention	Corches, Casey L.; McBride, A. Camille; Robles, Maria Cielito; Rehman, Narmeen; Bailey, Sarah; Oliver, Alina; Skolarus, Lesli E.	2020	American Journal of Health Behavior	10.5993/AJHB.44.6.1
Implementation of a pilot community-based psychosocial intervention for patients with psychoses in Chile and Brazil: a comparative analysis of users' perspectives	Dev, Saloni; Kankan, Tanvi; Blasco, Drew; Le, PhuongThao D.; Agrest, Martin; Dishy, Gabriella; Mascayano, Franco; Schilling, Sara; Jorquera, María José; Dahl, Catarina; Cavalcanti, Maria Tavares; Price, LeShawndra; Conover, Sarah; Yang, Lawrence H.; Alvarado, Rubén; Susser, Ezra S.	2021	Cambridge Prisms: Global Mental Health	10.1017/gmh.2021.10
Incorporating Community Perspectives to Inform the Scaling-Out of an Evidence-Based Executive Functioning Intervention from Schools to Community Mental Health Settings	Dickson, K. S.; Galligan, M.; Holt, T.; Anthony, L.; Kenworthy, L.; Brookman-Frazee, L.	2023	Evidence-Based Practice in Child and Adolescent Mental Health	10.1080/23794925.2023.2191353
Evaluation of the implementation process of the mobile health platform 'WelTel' in six sites in East Africa and Canada using the modified consolidated framework for implementation research (mCFIR)	El Joueidi, Samia; Bardosh, Kevin; Musoke, Richard; Tilahun, Binyam; Abo Moslim, Maryam; Gourlay, Katie; MacMullin, Alissa; Cook, Victoria J.; Murray, Melanie; Mbaraga, Gilbert; Nsanzimana, Sabin; Lester, Richard	2021	BMC Medical Informatics & Decision Making	10.1186/s12911-021-01644-1
Learning in real world practice: Identifying implementation strategies to integrate health-related social needs screening within a large health system	Fiori, K.; Levano, S.; Haughton, J.; Whiskey-Lalanne, R.; Telzak, A.; Hodgson, S.; Spurrell-Huss, E.; Stark, A.	2023	Journal of Clinical and Translational Science	10.1017/cts.2023.652
Changing maternal, infant and young child nutrition practices through social and behaviour change interventions implemented at scale: Lessons learned from Alive & Thrive	Flax, V. L.; Bose, S.; Escobar-DeMarco, J.; Frongillo, E. A.	2023	Maternal and Child Nutrition	10.1111/mcn.13559
Understanding how context and culture in six communities can shape implementation of a complex intervention: a comparative case study	Gaber, Jessica; Datta, Julie; Clark, Rebecca; Lamarche, Larkin; Parascandalo, Fiona; Di Pelino, Stephanie; Forsyth, Pamela; Oliver, Doug; Mangin, Dee; Price, David	2022	BMC Health Services Research	10.1186/s12913-022-07615-0
Accelerated scale-up of Kangaroo Mother Care: Evidence and experience from an implementation-research initiative in south India	Jayanna, K.; Rao, S.; Kar, A.; Gowda, P. D.; Thomas, T.; Swaroop, N.; Washington, M.; Shashidhar, A. R.; Rai, P.; Chitrapu, S.; Mohan, H. L.; Martines, J.; Mony, P.	2023	Acta Paediatrica, International Journal of Paediatrics	10.1111/apa.16236
Accelerating HIV epidemic control in Benue state, Nigeria, 2019–2021: the APIN program experience	Jwanle, P.; Ibiloye, O.; Obaje, M.; Ngwoke, K.; Usha, T.; Amoo, O.; Ogunsola, O.; Okezie, U.; Olaitan, R.; Ofuche, E.; Onwuatuelo, I.; Samuels, J.; Fagbamigbe, J.; Nwagagbo, F.; Ogbanufe, O.; Okoye, M.; Okonkwo, P.	2023	Therapeutic Advances in Infectious Disease	10.1177/20499361231153549
Designing for Scale and taking scale to account: lessons from a community score card project in Uganda	Kiracho, Elizabeth Ekirapa; Aanyu, Christine; Apolot, Rebecca Racheal; Kiwanuka, Suzanne Namusoke; Paina, Ligia	2021	International Journal for Equity in Health	10.1186/s12939-020-01367-1
Leveraging the Scaling Up Nutrition Movement to Operationalize Stunting Prevention Activities: Implementation Lessons From Rural Malawi	Kodish, S. R.; Farhikhtah, A.; Mlambo, T.; Hambayi, M. N.; Jones, V.; Aburto, N. J.	2022	Food and nutrition bulletin	10.1177/03795721211046140
A scalable health system model to achieve high coverage and quality of Kangaroo mother care in Uttar Pradesh, India	Kumar, A.; Tiwari, M.; Krishna, R.; Singh, P. K.; Sahu, A.; Singh, V.; Mishra, A.; Kumar, P.; Kumar, A.; Darmstadt, G. L.; Kumar, V.	2023	Acta Paediatrica, International Journal of Paediatrics	10.1111/apa.16534
Upscaling HIV and hepatitis C testing in primary healthcare settings: Stigma-sensitive practice	Lenton, E.; Johnson, J.; Brown, G.	2021	Australian Journal of Primary Health	10.1071/PY20176
Enabling patient-centred policy for electronic consultations: A qualitative analysis of discussions from a stakeholder meeting	Liddy, Clare; Hauteclocque, Jennifer; Moroz, Isabella; Oppenheimer, Luis; Sturge, Don; Burns, Katharina Kovacs; Horsley, Tanya; Tuot, Delphine; Keely, Erin	2022	Journal of Telemedicine & Telecare	10.1177/1357633X20926779
Scale and spread of innovation in health and social care: Insights from the evaluation of the New Care Model/Vanguard programme in England	MacInnes, Julie; Billings, Jenny; Coleman, Anna; Mikelyte, Rasa; Croke, Sarah; Allen, Pauline; Checkland, Kath	2023	Journal of Health Services Research & Policy	10.1177/13558196221139548
Biolarviciding implementation in southern Tanzania: Scalability opportunities and challenges	Matindo, A. Y.; Meshi, E. B.; Kapologwe, N. A.; Kengia, J. T.; Kajange, S.; Chaki, P.; Munisi, D. Z.	2022	PLoS ONE	10.1371/journal.pone.0273490
Evaluating Digital Program Support for the Physical Activity 4 Everyone (PA4E1) School Program: Mixed Methods Study	McLaughlin, Matthew; Duff, Jed; McKenzie, Tom; Campbell, Elizabeth; Sutherland, Rachel; Wiggers, John; Wolfenden, Luke	2021	JMIR Pediatrics & Parenting	10.2196/26690
Developing a comprehensive school-based physical activity program with flexible design - from pilot to national program	Mooses, Kerli; Vihalemm, Triin; Uibu, Marko; Mägi, Katrin; Korp, Leene; Kalma, Maarja; Mäestu, Evelin; Kull, Merike	2021	BMC Public Health	10.1186/s12889-020-10111-x
Effectiveness of participatory women's groups scaled up by the public health system to improve birth outcomes in Jharkhand, eastern India: A pragmatic cluster non-randomised controlled trial	Nair, N.; Tripathy, P. K.; Gope, R.; Rath, S.; Pradhan, H.; Rath, S.; Kumar, A.; Nath, V.; Basu, P.; Ojha, A.; Copas, A.; Houweling, T. A. J.; Haghparast-Bidgoli, H.; Minz, A.; Baskey, P.; Ahmed, M.; Chakravarthy, V.; Mahanta, R.; Prost, A.	2021	BMJ global health	10.1136/bmjgh-2021-005066
Perceptions of COVID-19 self-testing and recommendations for implementation and scale-up among Black/African Americans: implications for the COVID-19 STEP project	Nwaozuru, Ucheoma; Obiezu-Umeh, Chisom; Diallo, Hassim; Graham, Danielle; Whembolua, Guy-Lucien; Bourgeau, Marie Janeeca; Ritchwood, Tiarney D.; Nelson, LaRon E.; Shato, Thembekile; Mathews, Allison; Moise, Rhoda; Ward, Maranda C.; Raude, Jocelyn; Ahonkhai, Aima A.; Young, Diane J.; Conserve, Donaldson F.	2022	BMC Public Health	10.1186/s12889-022-13619-6
Facilitators and barriers to seasonal malaria chemoprevention (SMC) uptake in Nigeria: a qualitative approach	Ogbulafor, Nnenna; Uhomoibhi, Perpetua; Shekarau, Emmanuel; Nikau, Jamilu; Okoronkwo, Chukwu; Fanou, Nadia M. L.; Mbaye, Ibrahima Marietou; Ndiaye, Jean-Louis; Tchouatieu, Andre-Marie; Poku-Awuku, Abena; Merle, Corinne; Scott, Susana; Milligan, Paul; Ali, Aminu; Yusuf, Hauwa'u Evelyn; Oguche, Stephen; Dahiru, Tukur	2023	Malaria Journal	10.1186/s12936-023-04547-w
Building Capacity for Patient-Oriented Research: Utilizing Decision Aids to Translate Evidence into Practice, Policy and Outcomes	Parry, Monica; Richards, Dawn P.; Wells, David; Najam, Adhiyat; Hemani, Salima; Marlin, Susan	2022	Healthcare Quarterly	10.12927/hcq.2022.26776
The Local Heroes Project: a youth-led pan-India hyperlocal crisis relief model during the COVID-19 pandemic	Patil, D.; Shah, P.; Rao, S.; Arora, A.; Shah, D.; Sarangi, P.; Gogoi, K.; Dutta, M.; Thakore, Y.; Pawar, S.; Zhang, Z.; Swaroop, M.	2023	Frontiers in public health	10.3389/fpubh.2023.1282289
Scaling out a palliative compassionate community innovation: Nav-CARE	Pesut, Barbara; Duggleby, Wendy; Warner, Grace; Ghosh, Sunita; Bruce, Paxton; Dunlop, Rowena; Puurveen, Gloria	2022	Palliative Care & Social Practice	10.1177/26323524221095102
Stakeholder perspectives on Nigeria's national sodium reduction program: Lessons for implementation and scale-up	Sanuade, O. A.; Alfa, V.; Yin, X.; Liu, H.; Ojo, A. E.; Shedul, G. L.; Ojji, D. B.; Huffman, M. D.; Orji, I. A.; Okoli, R. C. B.; Akor, B.; Ripiye, N. R.; Eze, H.; Okoro, C. E.; Van Horn, L.; Tripathi, P.; Ojo, T. M.; Trieu, K.; Neal, B.; Hirschhorn, L. R.	2023	PLoS ONE	10.1371/journal.pone.0280226

Achieving Spread, Scale Up and Sustainability of Video Consulting Services During the COVID-19 Pandemic? Findings From a Comparative Case Study of Policy Implementation in England, Wales, Scotland and Northern Ireland	Shaw, S. E.; Hughes, G.; Wherton, J.; Moore, L.; Rosen, R.; Papoutsi, C.; Rushforth, A.; Morris, J.; Wood, G. W.; Faulkner, S.; Greenhalgh, T.	2021	Frontiers in Digital Health	10.3389/fdgth.2021.754319
A Stakeholder-Centered mHealth Implementation Inquiry Within the Digital Health Innovation Ecosystem in South Africa: MomConnect as a Demonstration Case	Sibuyi, I. N.; de la Harpe, R.; Nyasulu, P.	2022	JMIR mHealth and uHealth	10.2196/18188
Expanding video consultation services at pace and scale in scotland during the covid-19 pandemic: national mixed methods case study	Wherton, J.; Greenhalgh, T.; Shaw, S. E.	2021	Journal of Medical Internet Research	10.2196/31374
Scalability of digital psychological innovations for refugees: A comparative analysis in Egypt, Germany, and Sweden	Woodward, A.; Burchert, S.; Barry, A. S.; Broerse, J. E. W.; Sondorp, E.; Bold, A.; Ruberl, A.; Hessling, J. M.; Knaevelsrud, C.; Roberts, B.; Fuhr, D. C.; Ventevogel, P.; Hosny, N.; Lindegaard, T.; Shahnavaz, S.; Sijbrandij, M.; Cuijpers, P.; McKee, M.; Dieleman, M. A.	2023	SSM - Mental Health	10.1016/j.ssmmh.2023.100231
Community perceptions about use of pre-exposure prophylaxis among adolescent girls and young women in Kenya	Escudero, Jaclyn N.; Dettinger, Julia C.; Pintye, Jillian; Kinuthia, John; Lagat, Harison; Abuna, Felix; Kohler, Pamela; Baeten, Jared M.; O'Malley, Gabrielle; John-Stewart, Grace C.; Beima-Sofie, Kristin M.	2020	JANAC: Journal of the Association of Nurses in AIDS Care	https://dx.doi.org/10.1097/JNC.0000000000000191
Implementation science to design, test and scale up effective Kangaroo Mother Care in Oromia region, Ethiopia	Estifanos, A. S.; Haile Mariam, D.; Fikre, A.; Kote, M.; Tariku, A.; Chan, G. J.	2023	Acta Paediatrica	https://dx.doi.org/10.1111/apa.16413
Hypertension in Guatemala's Public Primary Care System: A Needs Assessment Using the Health System Building Blocks Framework	Fort, M. P.; Mundo, W.; Paniagua-Avila, A.; Cardona, S.; Figueroa, J. C.; Hernández-Galdamez, D.; Mansilla, K.; Peralta-García, A.; Roche, D.; Palacios, E. A.; Glasgow, R. E.; Gulayin, P.; Irazola, V.; He, J.; Ramirez-Zea, M.	2021	BMC Health Services Research	10.1186/s12913-021-06889-0
Strategies for effective implementation and scale-up of a multi-level co-designed men's health initiative "Sheds for Life" in Irish Men's Sheds	McGrath, A.; Richardson, N.; Murphy, N.	2022	Frontiers in Health Services	10.3389/frhs.2022.940031
Scaling the Moments <i>That Matter</i>® early childhood development model: how communities' monitoring for change contributes to sustainable impact	Murdock, D. E.; Munsongo, K.; Nyamor, G.	2023	Frontiers in Public Health	10.3389/fpubh.2023.1165991
Family strengthening in the context of covid-19: Adapting a community-based intervention from kenya to the united states.PS - First Posting	Puffer, Eve S.; Johnson, Savannah L.; Quick, Kaitlin N.; Rieder, Amber D.; Mansoor, Mahgul; Proeschold-Bell, Rae Jean; Jones, Sierra; Moore-Lawrence, Shaneeka; Rasmussen, Justin D.; Cucuzzella, Cameron; Burwell, Francelia; Dowdy, Latoria; Moore, Florine; Rosales, Nancy; Sanyal, Ameya; Ramachandran, Preetha; Duerr, Emmy; Tice, Logan; Ayuku, David; Boone, Wanda J.	2022	Prevention Science	https://dx.doi.org/10.1007/s11121-022-01418-9
Pre-implementation adaptation of suicide safety planning intervention using peer support in rural areas	Woodward, E. N.; Lunsford, A.; Brown, R.; Downing, D.; Ball, I.; Gan-Kemp, J. M.; Smith, A.; Atkinson, O.; Graham, T.	2023	Frontiers in Health Services	10.3389/frhs.2023.1225171
Ethics and the treatment as prevention strategy among transgender women living with HIV in Argentina	Zalazar, Virginia; Aristegui, Ines; Socías, M. Eugenia; Cardozo, Nadir; Sued, Omar; Shannon, Kate; Duff, Putu	2021	Culture, Health & Sexuality	https://doi.org/10.1080/13691058.2020.1720821
