# Supplementary material for: Strategies for involving patients and the public in scaling initiatives in health and social services: A scoping review
Source: Health Expect. 2024 Jun 5;27(3):e14086. doi: 10.1111/hex.14086 (PMC11150745; doi:10.1111/hex.14086)
Supplement: Supplementary file 9 — Supporting information. [file HEX-27-e14086-s003.pdf]

Patient and public involvement in scaling in HSS. Coroa et al. 2024

Additional File 9 Excluded reports

Title	ID	exclusion reson
Diffusion of advanced medical imaging technology, CT, and MRI scanners, in Iran: A qualitative study of determinants	Abedini 2019	not PPI
A Participatory Regional Partnership Approach to Promote Nutrition and Physical Activity Through Environmental and Policy Change in Rural Missouri	Barnidge 2015	not scaling
A Matter of Balance: Sharing Front-Line Ownership for Quality and Safety with Patients and Families - Scaling and Spreading Local Improvements for Broader Impact	Davies 2017	not document
Scaling up community-based obesity prevention in Australia: background and evaluation design of the Health Promoting Communities: Being Active Eating Well initiative	deSilva-Sanigorski 2010	not document
Key Stakeholders' Perspectives on Implementation and Scale up of HIV Self-Testing in Rwanda	Dzinamarira 2020	not PPI
Scaling up antenatal syphilis screening in Mozambique: transforming policy to action	Gloyd 2007	not PPI
From scaling up to sustainability in HIV: potential lessons for moving forward	Hirschhorn 2013	not PPI
A hybrid design testing a 3-step implementation model for community scale-up of an HIV prevention intervention in rural Malawi: study protocol	Jere 2018	not document
Research Community Collaboration in Observational Implementation Research: Complementary Motivations and Concerns in Engaging in the Study of Implementation as Usual	Lau 2020	not PPI
Application of the i-PARIHS framework for enhancing understanding of interactive dissemination to achieve wide-scale improvement in Indigenous primary healthcare	Laycock 2018	not document
Scaling up a Mobile Telemedicine Solution in Botswana: Keys to Sustainability	Ndlovu 2014	not PPI
Disseminating evidence-based treatments for PTSD in organizational settings: A high priority focus area	Ruzek 2009	not PPI
Implementation and adoption of nationwide electronic health records in secondary care in England: final qualitative results from prospective national evaluation in "early adopter" hospitals	Sheikh 2011	not PPI
Collaboration, negotiation, and coalescence for interagency-collaborative teams to scale-up evidence-based practice	Aarons 2014	not PPI
Health technology diffusion in developing countries: a case study of CT scanners in Brazil	Silva 2011	not PPI
An analysis of stakeholder networks to support the breastfeeding scale-up environment in Mexico	Buccini 2020	not outome
Anything but engaged: user involvement in the context of a national electronic health record implementation	Cresswell 2011	not scaling
Engagement of community stakeholders to develop a framework to guide research dissemination to communities	Cunningham-Erves 2020	not scaling
An innovative toolkit: increasing the role and value of patient and public involvement in the dissemination of research findings	McNichol 2014	not scaling
Adopting a population-level approach to parenting and family support interventions	Prinz 2007	not scaling
Scaling up research-for-development innovations in food and agricultural systems	Shilomboleni 2019	pas HSS
A protocol paper: community engagement interventions for cardiovascular disease prevention in socially disadvantaged populations in the UK: an implementation research study	Nahar 2020	not HSS

Online randomized controlled experiments at scale: lessons and extensions to medicine	Kohavi 2020	not PPI
The successful scale-up of direct-acting antiviral hepatitis C treatments will benefit from concerted investments in implementation science	Knight 2019	not document
Scaling Up Teleophthalmology for Diabetic Eye Screening: Opportunities for Widespread Implementation in the USA	Liu 2019	not PPI
Pre-exposure prophylaxis rollout in a national public sector program: the Kenyan case study	Masyuko 2018	not PPI
'Doing more with less': a qualitative investigation of perceptions of South African health service managers on implementation of health innovations	Brooke-Sumner 2019	not PPI
Effective strategies for scaling up evidence-based practices in primary care: a systematic review	BenCharif 2017	not PPI
National Scale-Up of Results-Based Financing in Primary Health Care: The Case of Armenia	Petrosyan 2017	not PPI
Development and Pilot Evaluation of a Tablet-Based Application to Improve Quality of Care in Child Mental Health Treatment	Ruggiero 2015	not PPI
Development of a National Physical Activity Plan for the United States	Bornstein 2014	not PPI
Recommendations for scale-up of community-based misoprostol distribution programs	Robinson 2014	not PPI
Public-private interactions on health in South Africa: opportunities for scaling up	Kula 2014	not PPI
Dissemination and implementation of comparative effectiveness evidence: key informant interviews with Clinical and Translational Science Award institutions	Morrato 2013	not PPI
'FAN the SUN brighter': fortifying Africa nutritionally (FAN) - the role of public private partnership in scaling up nutrition (SUN) in West Africa	Sablah 2013	not PPI
Scaling-up and rooting-down: a case study of North-South partnerships for health from Tanzania	Corbin 2012	not PPI
The NIHR Collaborations for Leadership in Applied Health Research and Care (CLAHRC) for Greater Manchester: combining empirical, theoretical and experiential evidence to design and evaluate a large-scale implementation strategy	Harvey 2011	not PPI
Tackling health workforce shortages during antiretroviral treatment scale-up--experiences from Ethiopia and Malawi	Rasschaert 2011	not PPI
Design, implementation and scaling up of the balanced scorecard for hospitals in Lebanon: policy coherence and application lessons for low and middle income countries	El-Jardali 2011	not PPI
Evaluating expansion of traditional home care agencies	Williams 1990	not document
Scaling Sukh best practice, resource team and engaging key stakeholders	UlHasan 2017	not document
A New Framework and Practice Center for Adapting, Translating, and Scaling Evidence-Based Health/Wellness Programs for People With Disabilities	Rimmer 2016	not scaling
Using peer educators to scale-up HIV oral self-testing among female sex workers: An implementation science approach from Kenya	Chesang 2016	not document

Scaling up safe abortion services under global comprehensive abortion care initiative in ten districts of Nepal-impact and effect of innovative approaches on the uptake of safe abortion services & post abortion contraception	Thapa 2015	not document
Scaling-up community-based programmes (CBPs) for childhood obesity prevention: First results following the methodology appraisal of 13 CBPs	Mantziki 2015	not document
SCALING UP: Participatory health research through healthy universities	Brito 2012	not document
Assessment of a national monitoring and evaluation system for rapid expansion of antiretroviral treatment in Malawi	Lowrance 2007	not scaling
Scaling Up Diabetes Prevention Programs in North Carolina: Perceptions of Demand From Potential Program Recipients and Providers	Thomas 2019	not scaling
Key players in conservation diffusion: Using social network analysis to identify critical injection points	Mbaru 2017	not scaling
A Framework for Evaluating Implementation of Community College Workforce Education Partnerships and Programs	Yarnall 2016	not HSS
Implementing SDG 15: Can large-scale public programs help deliver biodiversity conservation, restoration and management, while assisting human development?	Bridgewater 2015	not HSS
Integrating participatory community mobilization processes to improve dengue prevention: an eco-bio-social scaling up of local success in Machala, Ecuador (vol 109, pg 126, 2015)	Mitchell-Foster 2015	not HSS
Perils of scaling up: Effects of expanding a nutrition programme in Madagascar	Weber 2019	not PPI
They would never receive you without a husband': Paradoxical barriers to antenatal care scale-up in Rwanda	PÃ¥fs 2015	not scaling
SSP 2014: Engaging With Stakeholder Perspectives	Oâ€™Neill 2014	not document
Assessment of Private Sector Physicians' Willingness to Participate in Family Physician Program Using the Diffusion of Innovation Model, Qom, 2012, Iran	Aligol 2014	not HSS
Healthy lifestyle programs in out-of-home care: Implementing preventative trauma-informed approaches at scale	Pizzirani 2020	not PPI
Scaling as an argumentative resource in television talk shows	Lee 2019	not HSS
Statewide scale-up of group prenatal care in South Carolina	VanDeGriend 2016	not PPI
Building a Local Curricular Diffusion Model Based on a Gamified Homework Platform in First Year Engineering: A Case Study	Streiner 2019	not PPI
Getting to Scale: Evidence, Professionalism, and Community	Slavin 2016	not HSS
Supporting Statewide Implementation of the Learning School Initiative. Catalyst Schools Research Study Report	Hammer 2016	not HSS
The LEARNING WISDOM Phase II Scale up Project	Nct 2019	not PPI
Going to Scale: A Case Study of an Indian Educational NGO	Guha 2019	not HSS
Participation and diffusion effects of a peer-intervention for HIV prevention among adults in rural Malawi	Crittenden 2015	not PPI
The Effect of Peer Support in the Implementation of Evidence Based Practices	Wesp 2012	not PPI

Engagement in Practice: Scaling Community-based Design Experiences	Oakes 2018	not HSS
Prevention of mother-to-child HIV transmission in resource-limited settings: assessment of 99 Viramune Donation Programmes in 34 countries, 2000-2011	Ladner 2013	not PPI
Building District Capacity for System-Wide Instructional Improvement in Erie Public Schools. Working Paper. GE Foundation "Developing Futures"™ in Education Evaluation Series	Riggan 2013	not HSS
A qualitative evaluation of a simplified cardiovascular management program in Tibet, China	Tian 2018	not scaling
Translating evidence-based interventions into practice: the design and development of the Merck Childhood Asthma Network, Inc. (MCAN)	Viswanathan 2011	not scaling
Evidence-based dialogue with communities for district health systems' performance improvement	Kaseje 2010	not scaling
A community-directed strategy for sustainable malaria elimination on islands: short-term MDA integrated with ITNs and robust surveillance	Kaneko 2010	not scaling
Designing and delivering Educational Therapeutic Program training kit for HAE patients across France	Boccon-Gibod 2019	not document
Using liver cancer prevention messages to scale up the diagnosis and treatment of people living with hepatitis B	E.-.-Y. Adamson, N.-//-Schreiber, E.	not document
A national program to assess and improve healthcare humanization in hospital through a partnership between citizens and healthcare professionals	Caracci 2018	not document
Program science and the mobilization of knowledge and communities: Towards an integrated model for HIV prevention and health promotion	Otis 2015	not document
Assessing partnership linkages for health workforce and research capacity building in Kenya; Lessons learned	Gathatwa 2015	not HSS
Policy implication of qualitative exploration of stakeholders perspectives about integration of essential nutrition actions/ interventions into nigeria health system	Anjorin 2013	not document
Rapid assessment: an international review of diffusion, practice and outcomes in the substance use field	Fitch 2004	wrong type of document
Scaling up complex interventions: Insights from a realist synthesis	Willis 2016	wrong type of document
Scaling up pre-exposure prophylaxis in sub-Saharan Africa	Ahmed 2019	wrong type of document
Scaling up a community-based program for maternal and child nutrition in Thailand	Winichagoon 2014	wrong type of document
Factors influencing the scale-up of public health interventions in low- and middle-income countries: a qualitative systematic literature review	Bulthuis 2020	wrong type of document
Keeping the spirit of community partnerships alive in the scale up of HIV/AIDS prevention: critical reflections on the roll out of DEBI (Diffusion of Effective Behavioral Interventions)	Dworkin 2008	wrong type of document
Consumer engagement and the development, evaluation, and dissemination of evidence-based parenting programs	Sanders 2012	wrong type of document

Scaling up HIV prevention efforts targeting people who inject drugs in Central Asia: a review of key challenges and ways forward	Boltaev 2013	wrong type of document
A five-step approach for developing and implementing a Rural Primary Health Care Model for Dementia: a community-academic partnership	Morgan 2019	not scaling
Recently acquired and early chronic hepatitis C in MSM: Recommendations from the European treatment network for HIV, hepatitis and global infectious diseases consensus panel		wrong type of document
A Report on an Evaluation of Mother Tongue Based Early Learning and Parents + (MTELP+) Programme		not HSS
Scaling Up School and Community Partnerships: The Community Schools Strategy. Building Capacity for Community Schools Series		not HSS
New Antenatal Model in Africa and India (NAMAI) study: implementation research to improve antenatal care using WHO recommendations		not scaling
Socio-demographic trends in malaria knowledge and implications for behaviour change interventions in Zanzibar	Abbas 2023	not PPI
Considerations for community-based mHealth initiatives: insights from three Beacon Communities	Abebe 2013	not scaling
Post introduction evaluation of the malaria vaccine implementation programme in Ghana, 2021	Adjei 2023	not scaling
Defining the Enablers and Barriers to the Implementation of Large-scale, Health Care-Related Mobile Technology: Qualitative Case Study in a Tertiary Hospital Setting	Aggarwal 2022	not PPI
Model for regional collaboration: Successful strategy to implement a pediatric early warning system in 36 pediatric oncology centers in Latin America	Agulnik 2022	not PPI
Patient Navigators: an innovative approach to improve hepatitis c case finding leveraging existing human immunodeficiency virus service delivery models to reach last mile patients in Nasarawa State, Nigeria	Agwuocha 2022	wrong type of document
Identifying pathways for large-scale implementation of a school-based mental health programme in the Eastern Mediterranean Region: a theory-driven approach	Alonge 2020	not scaling
A rapid increase in coverage of COVID-19 vaccination, Central African Republic	Amani 2023	not scaling
Stepping towards integrated supports for family caregivers: Engaging Multilevel Interdisciplinary Stakeholders in Co-design of Competency-Based Education	Anderson 2022	wrong type of document
Effectiveness of community mobilisation models in improving HIV testing services uptake among women and children in Nigeria: A quasi-experimental study	Arije 2023	not scaling
Factors affecting successful scale-up of health-related pilot projects	Ashraf 2021	not PPI
Lively Minds: improving health and development through play-a randomised controlled trial evaluation of a comprehensive ECCE programme at scale in Ghana	Augsburg 2022	wrong type of document
Barriers and facilitators to implementing a Pharmacist, Physician, and Patient Navigator-Collaborative Care Model (PPP-CCM) to treat hepatitis C among people who inject drugs	Austin 2023	not PPI
Learning health care network heart failure: Towards a better heart failure care in Belgium	Baldewijns 2021	wrong type of document

Role of Co-creation for Large-Scale Sustainable Adoption of Digitally Supported Integrated Care: Prehabilitation as Use Case	Baltaxe 2022	not PPI
Tools for assessing the scalability of innovations in health: a systematic review	BenCharif 2022	not PPI
Changes in a Digital Type 2 Diabetes Self-management Intervention During National Rollout: mixed Methods Study of Fidelity	Benton 2022	not PPI
Scaling Family Voices and Engagement to Measure and Improve Systems Performance and Whole Child Health: Progress and Lessons from the Child and Adolescent Health Measurement Initiative	Bethell 2023	wrong type of document
Evidence-based interventions for improvement of maternal and child nutrition: What can be done and at what cost?	Bhutta 2013	not scaling
Uptake and Acceptability of MyChoices: Results of a Pilot RCT of a Mobile App Designed to Increase HIV Testing and PrEP Uptake Among Young American MSM	Biello 2022	not scaling
Active case-finding policy development, implementation and scale-up in high-burden countries: A mixed-methods survey with National Tuberculosis Programme managers and document review	Biermann 2020	not PPI

Multi-level stakeholders' perspectives on implementation and scaling up community-based health promotion in Germany	Birkholz 2023	not PPIRoberta Corôa (2024-02-17 09:47:31)(Select): See bellow to identify stakeholders at the community level: alors, aucun patient ou public engagée<br><br>Appendix 2: Information about participants Workshop with stakeholders at the community level A total of 172 people registered for the workshop with stakeholders at the community level, of which 161 also attended the workshop. Of the attendees, 86 were from the urban
Process evaluation of a primary care-based type 2 diabetes remission project in the North East of England	Boocock 2023	not PPI
Leveraging the ExpandNet framework and operational partnerships to scale-up brief Cognitive Behavioral Therapy in VA primary care clinics	Boykin 2022	not PPI
Aligning quality improvement efforts and policy goals in a national integrated health system	Braganza 2022	not scaling
How to select interventions for promoting physical activity in schools? Combining preferences of stakeholders and scientists	Brandes 2023	not PPI
Implementing a Learning Collaborative for Population-Based Physical and Behavioral Health Integration	Brar 2021	not scaling
Strategies to support the provincial scaling-up of eConsult in Canada	Breton 2020	wrong type of document
California's COVID-19 Virtual Training Academy: Rapid Scale-Up of a Statewide Contact Tracing and Case Investigation Workforce Training Program	Brickley 2021	not PPI

Scale-up of a novel vital signs alert device to improve maternity care in Sierra Leone: a mixed methods evaluation of adoption	Bright 2023	not PPI
Becoming breastfeeding friendly in Wales: Recommendations for scaling up breastfeeding support	Brown 2023	not PPI
Exploring the economics of public health intervention scale-up: a case study of the Supporting Healthy Image, Nutrition and Exercise (SHINE) cluster randomised controlled trial	Brown 2022	not PPI
Adapting digital health interventions for the evolving HIV landscape: Examples to support prevention and treatment research	Budhwani 2022	not PPI
Assessing the scalability of a health management-strengthening intervention at the district level: a qualitative study in Ghana, Malawi and Uganda	Bulthuis 2022	not PPI
How to scale-up: a comparative case study of scaling up a district health management strengthening intervention in Ghana, Malawi and Uganda	Bulthuis 2023	not PPI
Transitional Care Management Quality Improvement Methods That Reduced Readmissions in a Rural, Primary Care System	Burdick 2022	not scaling
What Is the Role of Civil Society in Multisectoral Nutrition Governance Systems? A Multicountry Review	Busse 2020	not PPI
Assessing the scalability of an integrated falls prevention service for community-dwelling older people: a mixed methods study	Calnan 2022	not PPI
Enhancing the scalability of the collaborative care model for depression using mobile technology	Carleton 2020	not scaling
A Paramedic-based Program Across Canada Supports More Palliative Care Patients to Spend their Last Days at Home	Carter 2023	wrong type of document
A model for community-led peer-facilitated advance care planning workshops for the public	Carter 2023	not scaling
Implementation and scale-uping of text messages for primary care patients using Warfarin: A real life study	Chagas 2022	wrong type of document
Multi-stakeholder perspectives regarding preferred modalities for mental health intervention delivered in the orthopedic clinic: a qualitative analysis	Cheng 2023	not scaling
Stanford Vax Crew: A Model for Agile, Community-Centered Vaccination Campaigns	Cheng 2023	not PPI
Exploring the acceptability of a WHO school-based mental health program in Egypt: A qualitative study	Chiumento 2022	not PPI
Defining a Vision and Laying the Foundation for Integrated Care of Older Adults in Algoma	Chlebus 2022	wrong type of document
Project design and technology trade-offs for implementing a large-scale sexual and reproductive health mHealth intervention: Lessons from Sierra Leone	Chukwu 2023	not PPI
Scaling up public health interventions: case study of the polio immunization program in Indonesia	Cintyamena 2021	not PPI
Scaling up Rapid Linkage to HIV Treatment in Los Angeles County Using a Learning Collaborative Approach	Cohen 2023	wrong type of document
Establishing a Standardized Surveillance System for Health Care-Associated Infections in Vietnam	Coker 2022	not PPI

Mixed-methods process evaluation of the injury prevention Warming-up Hockey programme and its implementation	Cornelissen 2023	not PPI
Implementation conditions leading to the scale-up of an innovation involving the optimal use of antipsychotics in long-term care centers: The Optimizing Practices, Use, Care and Services-Antipsychotics (OPUS-AP) program	Couturier 2022	not PPI
Scaling up hepatitis C community-based treatment services to address healthcare inequalities in Sussex (U.K.)	Crofton-Biwer 2020	wrong type of document
Neonatal inpatient dataset for small and sick newborn care in low- and middle-income countries: systematic development and multi-country operationalisation with NEST360	Cross 2023	not scaling
Using Quality Improvement Methods to Pilot Test and Scale a Program of Serious Illness Conversations in an Oncology Learning Health System	Cullinan 2022	wrong type of document
Increasing access and uptake of SARS-CoV-2 at-home tests using a community-engaged approach	D'Agostino 2022	not scaling
‘All Aboriginal and Torres Strait Islander children should have access to the ASQ-TRAK’: Shared vision of an implementation support model for the ASQ-TRAK developmental screener	D'Aprano 2023	not scaling
What Makes Integration of Chronic Care so Difficult? A Macro-Level Analysis of Barriers and Facilitators in Belgium	Danhieux 2021	not scaling
Engaging Stakeholders to Adapt an Evidence-Based Family Healthy Weight Program	Darling 2023	not scaling
Implementation of the JOIN for ME Program for Families from Low-Income Backgrounds: The Use of Theory-Driven Formative Evaluation: Rhode Island CORD 3.0	Darling 2021	not scaling
Exploring Successful Implementation of Team-Based Care in Chronic Disease Management: A Case Study	daSilva 2022	wrong type of document
Promoting Self-Determination in Community Contexts: Experiences With Implementing the Self-Determined Learning Model of Instruction	Dean 2022	not scaling
TEC to support independent living	Dix 2022	wrong type of document
Building capacities for implementation of Integrated Care in Moldova...22nd International Conference on Integrated Care, May 23-25, 2022, Odense, Denmark	Dnestrean 2022	wrong type of document
Accessibility and utilization of birth companions in public facilities: lessons from implementation of birth companionship in Kigoma and Katavi region	Dominico 2022	wrong type of document
From an interventional study to a national scale-up: lessons learned from the Malakit strategy at the French Guiana–Suriname border	Douine 2023	not PPI
Het bevorderen van het implementatieproces van Krachtvoer, een lesprogramma over gezonde voeding voor het vmbo	Driessen-Willems 2023	not PPI
Considerations for Increasing Racial, Ethnic, Gender, and Sexual Diversity in HIV Cure-Related Research with Analytical Treatment Interruptions: A Qualitative Inquiry	Dubé 2022	not scaling

Developing a Culture of Health: Combining Research and Education to Develop Sustainable and Scalable Wellness Programs for Head Start Educators	Economos 2023	wrong type of document
Trial collaborators' perceptions of the process of delivering Healthy Beginnings advice via telephone calls or text messages	Ekambareshwar 2022	not scaling
“Endless Opportunities”: A qualitative exploration of facilitators and barriers to scale-up of two-way texting follow-up after voluntary medical male circumcision in Zimbabwe	Elkins 2023	not PPI
Defining the Scope of Knowledge Translation Within a National, Patient-Oriented Kidney Research Network	Elliott 2021	wrong type of document
Implementation of the Community-based Health Planning and Services (CHPS) in rural and urban Ghana: a history and systematic review of what works, for whom and why	Elsey 2023	not PPI
Barriers and drivers to capacity-building in global mental health projects	Endale 2020	not scaling
Enhancing the quality of psychological interventions delivered by telephone in mental health services: increasing the likelihood of successful implementation using a theory of change	Faija 2023	not scaling
Scaling Up a Strengthened Youth-Friendly Service Delivery Model to Include Long-Acting Reversible Contraceptives in Ethiopia: A Mixed Methods Retrospective Assessment	Fikree 2020	not PPI
A chair at the table: a scoping review of the participation of refugees in community-based participatory research in healthcare	Filler 2021	not scaling
Recommendations for Implementing Hepatitis C Virus Care in Homeless Shelters: The Stakeholder Perspective	Fokuo 2020	not PPI
Scaling Up Delivery of Biofortified Staple Food Crops Globally: Paths to Nourishing Millions	Foley 2021	not HSS
Developing a whole-school mental health and wellbeing intervention through pragmatic formative process evaluation: a case-study of innovative local practice within The School Health Research network	Gobat 2021	not scaling
SCIROCCO Exchange Knowledge Transfer Programme - Citizens' Empowerment in Integrated Care	Gociu 2022	wrong type of document
Development of a Culturally Sensitive Chatbot to Inform Living Donor Candidates of African Ancestry About Apol1 Genetic Testing	Gordon 2022	wrong type of document
Engaging People in Tobacco Prevention and Cessation: Reflecting Back Over 20 Years Since the Master Settlement Agreement	Graham 2020	not scaling
Adoptin of healthy baby toolkit in feeding children in fragile environments of Uganda	Grant 2023	wrong type of document
Global perspectives of determinants influencing HPV vaccine introduction and scale-up in low- and middle-income countries	Guillaume 2024	not PPI
Scaling up of peri-operative quality-improvement interventions in low and middle income countries: A systematic scoping review of implementation strategies and implementation effectiveness	Hadi 2021	wrong type of document

PUBLIC AND PATIENT INVOLVEMENT AND ENGAGEMENT (PPIE) OF OLDER PATIENTS IN CODESIGN OF RESEARCH INTO PERIOPERATIVE SERVICES	Hall 2023	wrong type of document
Scaling-up school mental health services in low resource public schools of rural Pakistan: the Theory of Change (ToC) approach	Hamdani 2021	not PPI
Telemonitoring at scale for hypertension in primary care: An implementation study	Hammersley 2020	not scaling
Practical Strategies for Improving Sustainability and Scale-up of Noncommunicable Disease-related Public Health Interventions: Lessons from the Better Health Program in Southeast Asia	Haregu 2023	not PPI
Mobile Team Vaccine - Standing up an Integrated Health Delivery System in 48 hours	Hay 2022	wrong type of document
Scaling-up through piloting: dual-track provider payment reforms in China's health system	He 2023	not PPI
Stakeholder perspectives on scaling up medical device reprocessing: A qualitative study	Hennein 2022	not PPI
Scaling up a community-based exercise program for women in difficult life situations in Germany—The BIG project as a case-study	Herbert-Maul 2021	not PPI
Planned adaptation and implementation of the Community Guide recommendations for increasing physical activity in rural community settings: A qualitative study	Hess 2023	not PPI
Rapidly scaling video visits during COVID-19: The ethos of virtual care at Yale Medicine	Hoffman 2020	not PPI
Scalability of effective adherence interventions for patients using cardiovascular disease medication: A realist synthesis-inspired systematic review	Hogervorst 2023	wrong type of document
Adoption of an Evidence-Based Intervention for Mammography Screening Adherence in Safety Net Clinics	Holcomb 2021	not PPI
Doing Things Differently: Transforming the Health and Care System and Model in Wales through Innovation	Howsen 2022	wrong type of document
Healthcare workers perspectives on client volumes and workload with differentiated service delivery models in the Kingdom of Eswatini	Hughey 2020	wrong type of document
Implementation of evidence-based multiple focus integrated intensified TB screening to end TB (EXIT-TB) package in East Africa: a qualitative study	Isangula 2023	not PPI
Identifying, replicating, and spreading health care innovations across a nation-wide health care system: VHA diffusion of excellence	Jackson 2020	wrong type of document
Post-intervention acceptability of multicomponent intervention for management of hypertension in rural Bangladesh, Pakistan, and Sri Lanka- a qualitative study	Jafar 2023	not scaling
Enabling factors and barriers to the sustainability and scale-up of drowning reduction interventions in Vietnam: a qualitative assessment	Jagnoor 2020	not PPI
Barriers to the implementation, uptake and scaling up of the healthy plate model among regular street food consumers: a qualitative inquiry in Dar-es-Salaam city, Tanzania	Kagaruki 2022	not scaling
The influence of maximum isometric muscle force scaling on estimated muscle forces from musculoskeletal models of children with cerebral palsy	Kainz 2018	not scaling

Care coordination in primary healthcare for patients with complex needs: A comparative case study	Karam 2022	wrong type of document
Developing a strategy to scale up place-based arts initiatives that support mental health and wellbeing: A realist evaluation of 'Arts for the Blues'	Karkou 2024	not PPI
A chair at the table: A scoping review of the participation of refugee adults and youth in healthcare research and policy design	Kaur 2021	wrong type of document
Leveraging low-cost mobile technologies to increase community participation and sustainability of the HIV response: The case of OVC care in eastern and northern Uganda	Kazibwe 2020	wrong type of document
A self-directed co-creation process for developing a care pathway for patients on oral anticancer therapy: A qualitative process evaluation	Kenis 2023	not PPI
A process for developing a sustainable and scalable approach to community engagement: community dialogue approach for addressing the drivers of antibiotic resistance in Bangladesh	King 2020	not scaling
Using a theory of change in monitoring, evaluating and steering scale-up of a district-level health management strengthening intervention in Ghana, Malawi, and Uganda - lessons from the PERFORM2Scale consortium	Kok 2022	not PPI
Social AU : Please note that there is adiscrepancy between the article Innovation for Health Research (SIFHR): Development of the SIFHR Checklist	Kpokiri 2021	not scaling
Rapid-Cycle Evaluation in an Early Intervention Program for Children With Developmental Disabilities in South India: Optimizing Service Providers' Quality of Work-Life, Family Program Engagement, and School Enrollment	Krishna 2020	not scaling
Health & Wellness Centers to Strengthen Primary Health Care in India: Concept, Progress and Ways Forward	Lahariya 2020	not scaling
Evolution of the monitoring and evaluation strategies to support the World Health Organization's Global Programme to Eliminate Lymphatic Filariasis	Lammie 2021	not PPI
Mastering stakeholders' engagement to reach national scale, sustainability and wide adoption of digital health initiatives: Lessons learnt from Burkina Faso	Lampariello 2021	no outcome
Innovative process to support the sustainability and scale-up of anxiety prevention programs in schools in Quebec, Canada	Lane 2022	wrong type of document
A pilot study of participatory and rapid implementation approaches to increase depression screening in primary care	Last 2021	not scaling
Development and Application of the Scale-Up Reflection Guide (SRG)	Lee 2023	not PPI
Exploring the key differences between the delivery of local quality improvement projects and multisite a € scaling up' programmes: Learning from kidney services	Lee 2021	no outcome
The feasibility and acceptability of collaborative learning in improving health worker performance on adolescent health: findings from implementation research in Moldova	Lesco 2019	not scaling
Crowdsourcing to design a marketing package to promote a WHO digital mental health intervention among Chinese young adults	Ling 2021	not scaling

Using Stakeholder Perceptions to Inform Future Efforts to Implement Mental Health First Aid Training in China: A Qualitative Study	Lu 2021	not PPI
Implementation Strategies Used to Increase Human Papillomavirus Vaccination Uptake by Adolescent Girls in Sub-Saharan Africa: A Scoping Review	Lubeya 2023	not scaling
Scale up of syphilis screening and treatment among pregnant women in Zimbabwe, 2021-2022	Machiha 2024	wrong type of document
Navigating Complex Implementation Contexts: Overcoming Barriers and Achieving Outcomes in a National Initiative to Scale Out Housing First in Canada	Macnaughton 2018	not HSS
Progress on the scaling up of HIV testing in South Africa through varied distribution models using the oral HIV self-test kit	Majam 2020	wrong type of document
Development and implementation of National External Quality Assurance Programs in a One Health approach: The Armenian experience	Manjengwa 2021	not scaling
A case study of digital health bridging health facilities and community health systems; The D-tree model	Mapunda 2023	wrong type of document
Macro-level barriers to scaling up integrated care in three countries: Belgium, Slovenia and Cambodia	Martens 2022	wrong type of document
Using the multiple streams model to elicit an initial programme theory: From policy dialogues to a roadmap for scaling up integrated care	Martens 2023	not PPI
Implementation evaluation of an Irish secondary-level whole school programme: a qualitative inquiry	McHale 2022	not scaling
What is the 'voltage drop' when an effective health promoting intervention for older adults—Choose to Move (Phase 3)—Is implemented at broad scale?	McKay 2023	not scaling
Scaling up public mental health care in Sub-Saharan Africa: Insights from infectious disease	Meffert 2021	wrong type of document
Implementers' experiences of delivering a parenting programme to reduce violence against children in Tanzania: Lessons learned from the scale-up	Mgunga 2022	not available
Digital Health Implementation Strategies Coproduced With Adults With Acquired Brain Injury, Their Close Others, and Clinicians: Mixed Methods Study With Collaborative Autoethnography and Network Analysis	Miao 2023	not scaling
Implementation of Web-Based Psychosocial Interventions for Adults With Acquired Brain Injury and Their Caregivers: Systematic Review	Miao 2022	not PPI
Digitalizing a public-private systems approach for combatting COVID-19 in Kisumu, Kenya	Milimo 2022	not scaling
Barriers to the utilization of community-based child and newborn health services in Ethiopia: a scoping review	Miller 2021	not scaling
Sharing Value And Vision Among The Integrated Care Chain: How Puglia Region Fosters The Collaborative Capacity Of Different Stakeholders Starting From Integrated Care Maturity Assessment	Mingolla 2022	not scaling

Integrating participatory community mobilization processes to improve dengue prevention: An eco-bio-social scaling up of local success in Machala, Ecuador	Mitchell-Foster 2015	not scaling
A five-step approach for developing and implementing a Rural Primary Health Care Model for Dementia: a community-academic partnership	Morgan 2019	not scaling
Scale-up of the Accrual to Clinical Trials (ACT) network across the Clinical and Translational Science Award Consortium: A mixed-methods evaluation of the first 18 months	Morrato 2020	not PPI
Navigate Your Health: A Case Study of Organisational Learnings from an Integrated Care Pilot for Children and Young People in Care	Moss 2021	no outcome
EAT WALK ENGAGE: MOVING FORWARDS WITH DELIRIUM PREVENTION	Mudge 2022	wrong type of document
From research to international scale-up: stakeholder engagement essential in successful design, evaluation and implementation of paediatric HIV testing intervention	Mugo 2020	not PPI
"It is still very little; I cannot pass it on": a qualitative study of experiences of diagnosis and treatment of acute HIV infection in Eswatini	Mukooza 2023	not scaling
Scaling up community-based health insurance in Ethiopia: a qualitative study of the benefits and challenges	Mulat 2022	not PPI
TRANSFORMING TRAINING STRATEGY FOR GLOBAL SCALE OF A QUALITY IMPROVEMENT (QI) INTERVENTION	Muniz-Talavera 2022	wrong type of document
Factors related to the implementation and scale-up of physical activity interventions in Ireland: a qualitative study with policy makers, funders, researchers and practitioners	Murphy 2023	not PPI
Adaptation of a model for integration of interventions for alcohol use disorders in primary health care in Tanzania	Mushi 2023	not PPI
Mapping the functioning and identifying the needs of the baby friendly community Initiative in Kenya using a systems approach	Mutoro 2023	wrong type of document
Implementation strategies, facilitators, and barriers to scaling up and sustaining demand generation in family planning, a mixed-methods systematic review	Nabhan 2023	wrong type of document
A public health approach to cervical cancer screening in Africa through community-based self-administered HPV testing and mobile treatment provision	Nakalembe 2020	not scaling
A Digital Health Approach to Facilitate Access to and Continuity of Palliative Care for People with Advanced Cancer in the Bidi Bidi Refugee Settlement in Uganda	Namisango 2023	wrong type of document
Role of community antiretroviral therapy champions in scaling up community refill service among PLWHIV in Geita region; Afya jumuishi project	Ndamugoba 2023	wrong type of document
Setting the global research agenda for community-based HIV service delivery through the faith sector	Ndlovu-Teijema 2021	not scaling
Challenges and perceptions of implementing mass testing, treatment and tracking in malaria control: a qualitative study in Pakro sub-district of Ghana	Ndong 2019	not scaling

Strengthening community and stakeholder participation in the implementation of integrated vector management for malaria control in western Kenya: a case study	Ng'ang'a 2021	not scaling
HIV SELF-TESTING IN VIETNAM: FROM PILOT TO SCALE UP	Ngoc 2023	wrong type of document
Using mHealth to Improve Timeliness and Quality of Maternal and Newborn Health in the Primary Health Care System in Ethiopia	Nigussie 2021	not PPI
TXTXT Implementation Preparation: Identifying Contextual Barriers and Facilitators to HIV Medication Adherence Among Youth Across 12 Clinics Nationwide	Nikolajuk 2024	wrong type of document
Complexity of implementing harm reduction services in community hospitals: A two-phase qualitative study	Nimsakul 2022	not PPI
That's how we roll! Using human-centred design to allow the community voice to design an educational campaign, social media and direct-to-consumer communication for PrEP rollout in Zambia	Njelesani 2020	wrong type of document
Role of digital health insurance management systems in scaling health insurance coverage in low- and Middle-Income Countries: A case study from Nigeria	Okuzu 2022	not PPI
PRE-IMPLEMENTATION PLANNING AND STAKEHOLDER ENGAGEMENT TO DEVELOP THE PEWS ADAPTATION TO SUPPORT HOSPITALS IN AFRICA/ASIA (PASHA) PROJECT	Omotola 2022	wrong type of document
Towards achieving the family planning targets in the African region: a rapid review of task sharing policies	Ouedraogo 2021	not scaling
Scirocco exchange knowledge transfer programme for capacity-building in integrated care	Pacevicius 2021	wrong type of document
Saving and Empowering young lives in PAKistan (SEPAK): an Exploratory Cluster Randomized Controlled Trial (cRCT)	Panagioti 2023	wrong type of document
Development and Introduction of the Filariasis Test Strip: A New Diagnostic Test for the Global Program to Eliminate Lymphatic Filariasis	Pantelias 2022	not PPI
Putting the social back into sociotechnical: Case studies of co-design in digital health	Papoutsi 2021	not scaling
Rethinking the scale up of Integrated Management of Childhood Illness	Patel 2018	wrong type of document
The Integrated Tracking, Referral, and Electronic Decision Support, and Care Coordination (I-TREC) program: scalable strategies for the management of hypertension and diabetes within the government healthcare system of India	Patel 2020	wrong type of document
Including the voice of older people in the co-design of perioperative pathways of care	Paveley 2023	not scaling
Scaling up integrated primary mental health in six low- And middle-income countries: Obstacles, synergies and implications for systems reform	Petersen 2019	not PPI
National Scale-Up of Results-Based Financing in Primary Health Care: The Case of Armenia	Petrosyan 2017	not PPI
Autopsy of a failed trial part 1: A qualitative investigation of clinician's views on and experiences of the implementation of the DAISIES trial in UK-based intensive eating disorder services	Phillips 2023	not PPI

Promoting equity through inclusive learning, planning and implementing: lessons from Nigeria's mass drug administration programme for neglected tropical diseases	Piotrowski 2023	not PPI
On the Accuracy and Scalability of Probabilistic Data Linkage over the Brazilian 114 Million Cohort	Pita 2018	not PPI
Using participatory action research to empower district hospital staff to deliver quality-assured essential surgery to rural populations in Malawi, Zambia, and Tanzania	Pittalis 2023	not PPI
Evaluation of an Implementation Package to Deliver the COPD CARE Service	Portillo 2023	wrong type of document
Towards an Implementation-STakeholder Engagement Model (I-STEM) for improving health and social care services	Potthoff 2023	not PPI
Hepatitis C screening, diagnosis, and treatment scale-up among people who use drugs: micro-elimination in an Iranian city…Iranian Congress of Gastroenterology and Hepatology (Virtual), November 16-19, 2021	Poustchi 2021	wrong type of document
Cost-effectiveness of a Community-based Hypertension Improvement Project (ComHIP) in Ghana: results from a modelling study	Pozo-Martin 2021	not scaling
Treatment goals for rheumatoid arthritis: patient engagement and goal collection	Predmore 2023	not scaling
Partnered innovation to implement timely and personalized care: A case study	Proctor 2021	not scaling
Success Factors for Scaling Up the Adoption of Digital Therapeutics Towards the Realization of P5 Medicine	Prodan 2022	not PPI
Identifying Public Healthcare Priorities in Virtual Care for Older Adults: A Participatory Research Study	Pu 2023	not PPI
Scaling and sustaining COVID-19 vaccination through meaningful community engagement and care coordination for underserved communities: hybrid type 3 effectiveness-implementation sequential multiple assignment randomized trial	Rabin 2023	wrong type of document
IDF21-0181 Leveraging Community Health Workers (CHWs) in scaling up Diabetic Retinopathy screening in Fiji: a qualitative study	Ram 2022	wrong type of document
Prioritising and planning scale-up research projects targeting non-communicable diseases: A mixed-method study by the Global Alliance for Chronic Diseases upscaling working group	Ramani-Chander 2023	not PPI
Considering treatment-as-prevention scale-up for Australian prisons: a qualitative sub-study of expert stakeholders from the Australian 'surveillance and treatment of prisoners with hepatitis C' project (SToP-C)	Rance 2021	not PPI
How Can We Meet the Needs of People Living With HIV/AIDS in a Lowand Middle Country for Tobacco Smoking Cessation? Steps Taken Through a Feasibility Study in Brazil	Ribeiro 2023	wrong type of document
Piloting scale up of CRADLE into routine maternity care in Sierra Leone	Ridout 2022	wrong type of document
Packaging and Scaling the Stanford Pediatric Weight Control Program: A Family-Based, Group, Behavioral Weight Management Program for Children with Obesity and Their Families: Background, Rationale, and Design of the Stanford CORD 3.0 Project	Robinson 2021	wrong type of document
Scaling-up Principles Leading to a Persistent Health Ecosystem, by a Coherent Sequence of Integrated Care Programmes	RossiMori 2022	wrong type of document

Describing, analysing and understanding the effects of the introduction of HIV self-testing in West Africa through the ATLAS programme in Côte d'Ivoire, Mali and Senegal	Rouveau 2021	wrong type of document
A Roadmap to Inform the Implementation of Evidence-Based Collaborative Care Interventions in Communities: Insights From the Michigan Mental Health Integration Partnership	Rusch 2021	not PPI
Hurdles to developing and scaling remote patients' health management tools and systems: a scoping review	Ruyobeza 2022	not scaling
Scaling up the "24/7 BHU" strategy to provide round-the-clock maternity care in Punjab, Pakistan: a theory-driven, coproduced implementation study	Salway 2022	wrong type of document
The complexity of scaling up an mHealth intervention: the case of SMS for Life in Tanzania from a health systems integration perspective	SantFruchtman 2021	not PPI
Post-ICU Care Is Transitional Care: A Qualitative Content Analysis of Stakeholder Perspectives on Barriers and Facilitators of Quality Post- ICU Care Delivery	Scheunemann 2022	wrong type of document
Facilitators of and Barriers to integrating telemonitoring in ALS care: A nationwide implementation study of ALS Home Monitoring & Coaching	Schmidt 2023	wrong type of document
Comparing the German enabling environment for nationwide Water Safety Plan implementation with international experiences: Are we still thinking big or already scaling up?	Schmiege 2020	not PPI
Development of self-management tip sheets for medical oncology and surgical patients electronically reporting symptoms in the home-care recovery setting	Schrag 2020	wrong type of document
A Population-Based Intervention to Improve Care Cascades of Patients With Hepatitis C Virus Infection	Scott 2021	not PPI
A community intervention increased HCV screening and treatment in King County, WA	Scott 2019	wrong type of document
Integrating a Sport-Based Trauma-Sensitive Program in a National Youth-Serving Organization	Shaikh 2021	not scaling
Innovating to increase access to diabetes care in Kenya: an evaluation of Novo Nordisk's base of the pyramid project	Shannon 2019	not scaling
INFORMING INTERVENTION DESIGN IN COGNITIVELY IMPAIRED POPULATIONS: LESSONS LEARNED FROM THE OPTIMIZE DEPRESCRIBING INTERVENTION	Sheehan 2022	wrong type of document
Scaling up community-delivered mental health support and care: A landscape analysis	Siddiqui 2022	not PPI
Facilitating community participation in family planning and contraceptive services provision and uptake: Community and health provider perspectives	Silumbwe 2020	not scaling
A POSITIVE SNOWBALL EFFECT: QUALITY IMPROVEMENT (QI) LAUNCH - FROM ONE SCHOOL OF PAEDIATRICS TO ANOTHER	Singh 2022	wrong type of document
Implementation Science of Mindfulness in Intellectual and Developmental Disabilities	Singh 2020	not scaling
A tale of 'politics and stars aligning': Analysing the sustainability of scaled up digital tools for front-line health workers in India	Singh 2021	not PPI
Development and Impact of Helping Babies Breathe Educational Methodology	Singhal 2020	not scaling

"If We Got a Win–Win, You Can Sell It to Everybody": A Qualitative Study Employing Normalization Process Theory to Identify Critical Factors for eHealth Implementation and Scale-up in Primary Care	Sivakumar 2022	not PPI
Rapid evaluation of service innovations in health and social care: key considerations	Smith 2023	not scaling
AddREssing Social Determinants to pRevent hypErtension (The RESTORE Network): Overview of the Health Equity Research Network to Prevent Hypertension	Spruill 2023	not scaling
Fitting Health Financing Reforms to Context: Examining the Evolution of Results-Based Financing Models and the Slow National Scale-Up in Uganda (2003-2015)	Ssennyonjo 2021	not PPI
Scaling up a community-based intervention for people affectedby dementia: What is the value?	Stephens 2021	wrong type of document
How Group-Based Interventions Can Improve Services for People with Severe Obesity	Swancutt 2019	not scaling
Exploring contextual adaptations in caregiver interventions for families raising children with developmental disabilities	Szlamka 2022	not scaling
Scale-up and sustainability of a personalized end-of-life care intervention: a longitudinal mixed-methods study	Takaoka 2021	not scaling
Participatory approaches to delivering clinical sexually transmitted infections services: a narrative review	Tan 2022	wrong type of document
Menstrual product choice and uptake among young women in Zimbabwe: a pilot study	Tembo 2020	not scaling
Key stakeholder perspectives on the development and real-world implementation of a home-based physical activity program for mothers at risk of postnatal depression: a qualitative study	Teychenne 2021	not PPI
Factors relating to sustainability and scalability of the 'Food, Move, Sleep (FOMOS) for Postnatal Mental Health' program: Qualitative perspectives from key stakeholders across Australia	Teychenne 2023	not PPI
Formative research to scale up a handwashing with soap and water treatment intervention for household members of diarrhea patients in health facilities in Dhaka, Bangladesh (CHoBI7 program)	Thomas 2020	not scaling
Cystic Fibrosis Learning Network Learning Structure for Multicenter Spread of Co-Production and Timely Patient Registry Data Entry Practices	Thomas 2022	wrong type of document
'We are the bridge': an implementation research study of SEWA Shakti Kendras to improve community engagement in publicly funded health insurance in Gujarat, India	Thomas 2022	not scaling
Crowdsourcing strategies to improve access to HIV pre-exposure prophylaxis (PrEP) in Australia, the Philippines, and China	Tieosapjaroen 2023	not scaling
Adolescent-Nutrition-and-Health: formative assessment of the School-Health Environment and Programs in Ethiopia, Sudan, and Tanzania	Tinkasimile 2022	wrong type of document
National Action towards a World Free of Cervical Cancer for All Women	Torode 2021	not PPI
Understanding maternity waiting home uptake and scale-up within low-income and middle-income countries: a programme theory from a realist review and synthesis	Uwamahoro 2022	not PPI
The role of partners in promoting self-care for misoprostol and subcutaneous DMPA in Pakistan	Uzma 2021	not scaling

Public-private partnership to rapidly strengthen and scale COVID-19 response in Western Kenya	VanDuijn 2021	not PPI
"It has to be better, otherwise we will get stuck." A Review of Novel Directions for Mental Health Reform and Introducing Pilot Work in the Netherlands	VanOs 2023	not scaling
Applying learning health systems thinking in codeveloping integrated tuberculosis interventions in the contexts of COVID-19	VanRensburg 2022	not PPI
Provider perspectives on service delivery modifications to maintain access to HIV pre-exposure prophylaxis during the COVID-19 pandemic: qualitative results from a PrEP implementation project in Kenya	Velloza 2023	not PPI
A narrative review of economic constructs in commonly used implementation and scale-up theories, frameworks and models	Vicki 2020	not PPI
Development of a facilitated cross-agency mentoring strategy to support scale-up and spread of a pediatric behavioral health integration model	Vieira 2020	wrong type of document
The eMEN Interreg Project - introduction and first results	Vlijter 2019	wrong type of document
Global HIV prevention, care and treatment services for children: A cross-sectional survey from the International Epidemiology Databases to Evaluate AIDS (IeDEA) consortium	Vreeman 2023	not PPI
Child health and the implementation of Community and District-management Empowerment for Scale-up (CODES) in Uganda: a randomised controlled trial	Waiswa 2021	not scaling
Guest Editorial: Psychologists Aim to HEAL the Opioid and Pain Crises	Wandner 2020	wrong type of document
A pilot study of Kangaroo mother care in early essential newborn care in resource-limited areas of China: the facilitators and barriers to implementation	Wang 2023	not PPI
Twenty years of capacity building across the cancer prevention and control research network	Wangen 2023	not scaling
A conceptual framework for effective dissemination and implementation of a policy on school health in rural Nigeria	Wankasi 2020	not scaling
ADAPTATION OF A BIDIRECTIONAL CRISIS AND EMERGENCY RISK COMMUNICATION FRAMEWORK BY COMMUNITY ENGAGED RESEARCH PARTNERSHIPS IN RURAL MISSISSIPPI DURING THE COVID-19 PANDEMIC	Washington 2023	wrong type of document
Survival probability and factors associated with time to loss to follow-up and mortality among patients on antiretroviral treatment in central Kenya	Wekesa 2022	not scaling
Scaling up Business Plans in Tajikistan: a qualitative study of the history, barriers, facilitators and lessons learnt	Werner 2021	wrong type of document
Barriers, opportunities, and potential costs of expanding HIV support services	Wheatley 2023	not PPI
Developments in scalable strategies for detecting early markers of cognitive decline	Whelan 2022	not scaling
A whole-of-health system approach to improving care of frail older persons	Whiting 2022	not PPI

TECHNOLOGY-ENABLED COLLABORATIVE CARE FOR DIABETES AND MENTAL HEALTH: LEARNING FROM STUDY FINDINGS AND COMMUNITY PROVIDERS TO ACHIEVE INTEGRATED DIABETES AND MENTAL HEALTH CARE	Whitmore 2023	wrong type of document
A process for converting an in-person training to increase church capacity to implement physical activity and healthy eating practices and policies to an online format	Wilcox 2023	not scaling
'What works here doesn't work there': The significance of local context for a sustainable and replicable asset-based community intervention aimed at promoting social interaction in later life	Wildman 2019	not HSS
Co-creation and Evaluation of Nationwide Remote Training Service for Mental Health Education of Community Health Workers in Rwanda	Willems 2021	not scaling
COVID-19 Conversations Within Black/Brown Minority Communities: A Stakeholder and Psychoeducation Approach Using Zoom/Facebook Live	Williams 2022	not scaling
Results-based financing as a strategic purchasing intervention: some progress but much further to go in Zimbabwe?	Witter 2020	not PPI
Stakeholder perspectives towards implementing the national framework on palliative care in Canada	Xiao 2022	not scaling
Opportunities and barriers to the uptake of a digitalised breast cancer Family History Assessment Pathway	Youngs 2023	wrong type of document
Financial Incentives for Pediatric HIV Testing (FIT): Caregiver Insights on Incentive Mechanisms, Focus Populations, and Acceptability for Programmatic Scale Up	Zhang 2021	not PPI
The one-dose schedule opens the door to rapid scale-up of HPV vaccination	Zou 2023	not scaling
Promoting Integrated Care through a Global Treatment Budget A Qualitative Study in German Mental Health Care using Rogers' Diffusion of Innovation Theory	Afraz 2021	not scaling
“Improved access, delayed accreditation, low recognition”: perspectives of mental health educators, preceptors and students on the Kintampo Project in Ghana	Agyekum 2023	not scaling
Scaled, citizen-led, and public qualitative research: A framework for citizen social science	Amirah 2023	not scaling
A qualitative analysis of rural syringe service program fidelity in Appalachian Kentucky: Staff and participant perspectives	Batty 2023	not scaling
Prevention Research with Indigenous Communities to Expedite Dissemination and Implementation Efforts	BlueBirdJernigan 2020	not scaling
Community-Supported Agriculture Networks in Wales and Central Germany: Scaling Up, Out, and Deep through Local Collaboration	Bonfert 2022	not HSS
Scaling Early Childhood Evidence-Based Interventions through RPPs	Brotman 2021	not PPI
Mobilising volunteers to deliver a school-based arts-in-nature practice to support children's mental health and wellbeing: a modified e-Delphi Study with primary school staff	Bungay 2024	not HSS
Learning to Enhance Community-Responsiveness in an Out-of-School Club Program	Burke 2023	not HSS
Implementing essential interventions for cardiovascular disease risk management in primary healthcare: lessons from Eastern Europe and Central Asia	Collins 2020	wrong type of document

Setting foot in private spaces: extending the hepatitis C cascade of care to automatic needle/syringe dispensing machines, a mixed methods study	Coupland 2022	not scaling
Mental Health Spillover Effects Among Cohabitating Caregivers of Sierra Leonean Youth Receiving a Cognitive Behavioral Therapy-Based Intervention	Desrosiers 2021	wrong type of document
Implementation of Community-Wide Initiatives Designed to Reduce Teen Pregnancy: Measuring Progress in a 5-Year Project in 10 Communities	Duane 2022	not scaling
Applying User-Centered Design and Implementation Science to the Early-Stage Development of a Telehealth-Enhanced Hybrid Cardiac Rehabilitation Program: Quality Improvement Study	Duran 2023	not scaling
Integration of Mental Health into Emergency Preparedness and Response Planning for the Monsoon Season in Bangladesh	Elshazly 2022	not scaling
From implementation to sustainment: A large-scale adverse event disclosure support program generated through embedded research in the Veterans Health Administration	Elwy 2021	not scaling
"Stopping the itch": mass drug administration for scabies outbreak control covered for over nine million people in Ethiopia	Enbiale 2020	not scaling
Handbook of school mental health: Innovations in science and practice., 3rd ed.ST - Issues in clinical child psychology	Evans 2023	wrong type of document
Community-Based Conversations about Advance Care Planning for Underserved Populations Using Lay Patient Navigators	Fink 2020	not scaling
System approaches to childhood obesity prevention: ground up experience of adaptation and real-world context	Fraser 2023	wrong type of document
A rapid mixed-methods evaluation of remote home monitoring models during the COVID-19 pandemic in England	Fulop 2023	not scaling
Engaging stakeholders to inform national implementation of critical time intervention in a program serving homeless-experienced Veterans	Gabrielian 2022	not scaling
Using Technology to Scale up Youth-Led Participatory Action Research: A Systematic Review	Gibbs 2020	not PPI
Frayme: Building the structure to support the international spread of integrated youth services	Halsall 2020	not scaling
"do I want prep or do I want a roof?": Social determinants of health and hiv prevention in the southern united states.PS - First Posting	Harrison 2022	Exclusion reason: No outcome;
Transferring a Community-Based Participatory Research Project to Promote Physical Activity Among Socially Disadvantaged Women-Experiences From 15 Years of BIG	Herbert-Maul 2020	not PPI
CASH TRANSFERS AND CAREGIVERS: WORKING TOGETHER TO REDUCE VULNERABILITY AND HIV RISK AMONG ADOLESCENT GIRLS IN JOHANNESBURG, SOUTH AFRICA	Hill 2021	not scaling
The feasibility of a Child and Family Hub within Victorian Community Health Services: a qualitative study	Honisett 2022	not PPI
Advancing the Large-Scale Implementation of Applied Behavior Analysis	Horner 2021	not PPI
Engagement of stakeholders in the design, evaluation, and implementation of complex interventions	Hudson 2020	not scaling

Integrated digital system for community engagement and community-based surveillance during the 2014-2016 Ebola outbreak in Sierra Leone: lessons for future health emergencies	Jalloh 2020	wrong type of document
Comprehensive primary health care and non-communicable diseases management: a case study of El Salvador	JimenezCarrillo 2020	not scaling
How central support built capacity to deliver a health-promoting intervention for older adults in Canada	Joanie 2022	not PPI
Rapid Scaling Up of Telehealth Treatment for Tobacco-Dependent Cancer Patients During the COVID-19 Outbreak in New York City	Kotsen 2021	not PPI
Research Community Collaboration in Observational Implementation Research: Complementary Motivations and Concerns in Engaging in the Study of Implementation as Usual	Lau 2020	not scaling
Tailoring implementation strategies for scale-up: Preparing to take the Med-South Lifestyle program to scale statewide	Leeman 2022	not PPI
Dissemination of a successful dementia care program: Lessons from early adopters	LeesHaggerty 2022	not PPI
Perceived benefits and barriers of mhealth mindfulness use for caregivers of older adults with cognitive impairment: A qualitative exploration	Llaneza 2022	not scaling
Implementing the patient care collaborative model in three general internal medicine units: a mixed-methods healthcare improvement initiative	LoPresti 2020	not PPI
Indigenous Youth Mentorship Program: a descriptive case study of implementation in Alberta, Canada	Lopresti 2020	not HSS
Barriers and facilitators to scaling up <i>Healthy Choices</i>, a motivational interviewing intervention for youth living with HIV	MacDonell 2022	not PPI
Voices from the Youth in Kenya Addressing Mental Health Gaps and Recommendations	Memiah 2022	not scaling
Effect and feasibility of district level scale up of maternal, newborn and child health interventions in Pakistan: a quasi-experimental study	Memon 2020	wrong type of document
Moving psychiatric deinstitutionalization forward: A scoping review of barriers and facilitators-Corrigendum	Montenegro 2023	not scaling
‘If there is joy… I think it can work well’: a qualitative study investigating relationship factors impacting HIV self-testing acceptability among pregnant women and male partners in Uganda	Naughton 2023	not scaling
Implementing Integrated Early Childhood Mental Health Services in Primary Care: Relationships, Vision, and Sustainability	Nayak 2023	not scaling
Qualitative Analysis of Community Support to Methadone Access in Kenya	Ndimbii 2021	not scaling
Impact of Technology Driven Mental Health Task-shifting for Accredited Social Health Activists (ASHAs): Results from a Randomised Controlled Trial of Two Methods of Training	Nirisha 2023	not scaling
Facilitators, barriers and considerations for the implementation of healthcare innovation: A qualitative rapid systematic review	Parmar 2022	not PPI
Systematic evaluation of the QualityRights programme in public mental health facilities in Gujarat, India	Pathare 2021	not scaling
Gearing to success with national breastfeeding programmes: The Becoming Breastfeeding Friendly (BBF) initiative experience	Pérez-Escamilla 2023	no outcome

Initial Feasibility of the "Families Moving Forward Connect" Mobile Health Intervention for Caregivers of Children With Fetal Alcohol Spectrum Disorders: Mixed Method Evaluation Within a Systematic User-Centered Design Approach	Petrenko 2021	not scaling
Successful scaling of Edible City Solutions to promote food citizenship and sustainability in food system transitions	Plassnig 2022	not HSS
The NIMH global mental health research community and COVID-19	Rahman 2020	wrong type of document
An early phase trial testing the proof of concept for a gamified smartphone app in manipulating automatic evaluations of exercise	Rasera 2022	not scaling
Evidence on Scaling in Health and Social Care: An Umbrella Review	RobertaDeCarvalho 2023	not PPI
The IPBES Global Assessment: Pathways to Action	Ruckelshaus 2020	not HSS
A Medical Translation Assistant for Non-English-Speaking Caregivers of Children With Special Health Care Needs: Proposal for a Scalable and Interoperable Mobile App	Sezgin 2020	wrong type of document
Delivering a complex mental health intervention in low-resource settings: Lessons from the implementation of the PRIME mental healthcare plan in primary care in Sehore district, Madhya Pradesh, India	Shidhaye 2019	not PPI
Scalability of Adaptation strategies to drought stress: the case of drought tolerant maize varieties in Kenya	Simtowe 2021	not HSS
Assessing fidelity to evidence-based quality improvement as an implementation strategy for patient-centered medical home transformation in the Veterans Health Administration	Stockdale 2020	not scaling
Avoiding pitfalls: Key insights and lessons learnt from customizing and rolling out a national web-based system in Tanzania	Sukums 2021	not PPI
Community-based health extension policy implementation in Ethiopia: a policy experience to scale-up	Tefera 2022	not scaling
Leveraging an Implementation Science Framework to Measure the Impact of Efforts to Scale Out a Total Worker Health^R Intervention to Employers	Tenney 2022	not PPI
A framework for Science Shop processes: Results of a modified Delphi study	Urias 2020	not scaling
TIPS for Scaling up Research in Upper Limb Prosthetics	vanderSluis 2020	wrong type of document
Implementation determinants and strategies in integration of PrEP into maternal and child health and family planning services: experiences of frontline healthcare workers in Kenya	Wagner 2023	not scaling
Undertaking rapid evaluations during the COVID-19 pandemic: Lessons from evaluating COVID-19 remote home monitoring services in England	Walton 2023	not scaling
Advancing School Behavioral Health at Multiple Levels of Scale	Weist 2022	not HSS
Realist review of community coalitions and outreach interventions to increase access to primary care for vulnerable populations: a realist review	Welch 2023	not scaling
Sdg platforms as strategic innovation through partnerships	Williams 2022	not HSS

Scaling a Behavioral Health Home Delivery Model to Special Populations	Williams 2023	not scaling
Scaling strategies, organizational capabilities and scaling social impact: An investigation of social enterprises in China	Yu 2023	not HSS
Facilitators and Barriers to Scaling-Up Integrated Care for Arterial Hypertension and Type 2 Diabetes in Slovenia: Qualitative Study	Zavrnik 2024	wrong type of document
