# Supplementary material for: Strategies for involving patients and the public in scaling initiatives in health and social services: A scoping review
Source: Health Expect. 2024 Jun 5;27(3):e14086. doi: 10.1111/hex.14086 (PMC11150745; doi:10.1111/hex.14086)
Supplement: Supplementary file 10 — Supporting information. [file HEX-27-e14086-s012.pdf]

Patient and public involvement in scaling in HSS Corôa et al. 2024
Additional File 10 - GRIPP2

	Background to paper		Aims of paper	Methods		Study results	Discussion and conclusions	
	Definition	Theoretical underpinnings	Aim	Design	People involved	Outcomes of PPI	Outcomes	Reflections/critical perspective
**Study ID**	Report the definition of PPI used in the study and how it links to comparable studies	Report the theoretical rationale and any theoretical influences relating to PPI in the study	Report the aim of the study	Provide a clear description of methods by which patients and the public were involved	Provide a description of patients, carers, and the public involved with the PPI activity in the study	Report the results of PPI in the study, including both positive and negative outcomes	Comment on how PPI influenced the study overall. Describe positive and negative effects	Comment critically on the study, reflecting on the things that went well and those that did not, so that others can learn from this study
Ashraf 2015	Yes	Yes	Yes	Yes	Yes	Yes	Yes	Yes
Awoonor-Williams 2013 (related to WHO-	Yes	Yes	Yes	Yes	Yes	Yes	Yes	Yes
Barber 2019	Yes	Yes	Yes	Yes	Yes	Yes	Yes	Yes
Basso 2017	No	Yes	Yes	Yes	Yes	Yes	Yes	Yes
Bennett 2017	No	Yes	Yes	Yes	Yes	Yes	Yes	Yes
Bradley 2012 (Related to WHO-13)	No	No	Yes	Yes	Yes	Yes	Yes	Yes
Callaghan-Koru 2020	No	Yes	Yes	Yes	Yes	Yes	Yes	Yes
Carnell 2014	Yes	Yes	Yes	Yes	Yes	Yes	Yes	Yes
CFHI-4 2021	No	No	Yes	Yes	Yes	Yes	Yes	No
CHFI-86 2017	No	Yes	Yes	Yes	Yes	Yes	Yes	Yes
Chandrashekar 2014	No	No	Yes	Yes	Yes	Yes	Yes	Yes
Chibanda 2017 (related to Chibanda 201(	Yes	Yes	No	Yes	Yes	Yes	Yes	Yes
Cislaghi 2019	No	Yes	Yes	Yes	Yes	Yes	Yes	Yes
Colom 2018	No	No	Yes	Yes	Yes	Yes	Yes	Yes
Desclaux 2010	No	Yes	Yes	Yes	Yes	Yes	Yes	Yes
Fagg 2014	No	Yes	Yes	Yes	Yes	Yes	Yes	Yes
Fort 2019	No	Yes	Yes	Yes	Yes	Yes	Yes	Yes
Fuhr 2020	No	Yes	Yes	Yes	Yes	Yes	Yes	Yes
Gaitonde 2020	No	Yes	Yes	Yes	Yes	Yes	Yes	Yes
Ghiron 2014	No	Yes	Yes	Yes	Yes	Yes	Yes	Yes
Google-18 2018 - REPORT	No	Yes	Yes	Yes	Yes	Yes	Yes	Yes
Google-180 2020 - REPORT	No	Yes	Yes	Yes	Yes	Yes	Yes	Yes
Google-182 2020 - GUIDANCE NOTE	No	Yes	Yes	Yes	Yes	Yes	Yes	No
Google-190	No	Yes	Yes	Yes	Yes	Yes	Yes	No
Google-202 2020	Yes	No	Yes	Yes	Yes	Yes	Yes	Yes
Google-234 2022	Yes	Yes	Yes	Yes	Yes	Yes	Yes	Yes
Google-28 2016	Yes	Yes	Yes	Yes	Yes	Yes	Yes	Yes
Google-29 2013	No	Yes	Yes	Yes	Yes	Yes	No	No
Google-381 2015	Yes	Yes	Yes	Yes	Yes	Yes	Yes	No
Google-40 2020	Yes	Yes	Yes	Yes	Yes	Yes	Yes	Yes
Google-57 2016	No	Yes	Yes	Yes	Yes	Yes	Yes	No
Google-62 2014	No	Yes	Yes	Yes	Yes	Yes	Yes	Yes
Held 2016	Yes	Yes	Yes	Yes	Yes	Yes	Yes	Yes
IHI-12 2020	No	Yes	Yes	Yes	Yes	Yes	Yes	Yes
IHI-4 2009	No	Yes	Yes	Yes	Yes	Yes	Yes	Yes
IHI-6 2015	No	No	Yes	Yes	Yes	Yes	Yes	Yes
Killingo 2017	No	Yes	Yes	Yes	Yes	Yes	Yes	Yes
King 2008	No	Yes	Yes	Yes	Yes	Yes	Yes	Yes
Koorts 2018	No	Yes	Yes	Yes	Yes	Yes	Yes	Yes
L'Engle 2017	No	Yes	Yes	Yes	Yes	Yes	Yes	Yes
Mai - 19 .	No	Yes	Yes	Yes	Yes	Yes	Yes	Yes
Mendel 2008	No	Yes	Yes	Yes	Yes	Yes	Yes	Yes
Moroz 2020	No	Yes	Yes	Yes	Yes	Yes	Yes	Yes
NICE-167 2016	No	No	Yes	Yes	Yes	Yes	Yes	Yes
NICE-221 2016	No	No	Yes	Yes	Yes	Yes	Yes	Yes
NICE-9 2021	No	Yes	Yes	Yes	Yes	Yes	Yes	Yes
NSW-6 2014 - Guide	No	No	Yes	Yes	Yes	Yes	Yes	No
Pinto 2015	No	Yes	Yes	Yes	Yes	Yes	Yes	Yes
Rhodes 2020	Yes	Yes	Yes	Yes	Yes	Yes	Yes	Yes
Soti-Ulberg 2020	No	No	Yes	Yes	Yes	Yes	Yes	Yes
Sperber 2008	No	Yes	Yes	Yes	Yes	Yes	Yes	Yes
Wagner 2007	No	No	Yes	Yes	Yes	Yes	Yes	Yes
Warren 2003	No	No	Yes	Yes	Yes	Yes	Yes	Yes
WHO-11 2003 - Report	No	Yes	Yes	Yes	Yes	Yes	Yes	Yes
WHO-120 2021 - GUIDE	No	No	Yes	Yes	Yes	Yes	Yes	Yes
WHO-169 2013 - Guide	No	Yes	Yes	Yes	Yes	Yes	Yes	Yes
WHO-179 2012 - guide	No	Yes	Yes	Yes	Yes	Yes	Yes	No
WHO-30 2014	No	No	Yes	Yes	Yes	Yes	Yes	Yes
WHO-34 2011 - guide	No	No	Yes	Yes	Yes	Yes	Yes	Yes
WHO-414 2018	No	No	Yes	Yes	Yes	Yes	Yes	Yes
WHO-553 2017	No	No	Yes	Yes	Yes	Yes	Yes	Yes
WHO-7 2015	No	Yes	Yes	Yes	Yes	Yes	Yes	Yes
WHO-8 2018	No	Yes	Yes	Yes	Yes	Yes	Yes	Yes
WHO-9 2020	No	Yes	Yes	Yes	Yes	Yes	Yes	No
Yamey 2011	No	No	Yes	Yes	Yes	Yes	Yes	Yes
Zalazar 2021	No	No	Yes	Yes	Yes	Yes	Yes	Yes
Woodward 2023	Not reported	Yes	Yes	Yes	Yes	Yes	Yes	Yes
Puffer 2022	No	No	Yes	Yes	Yes	Yes	Yes	Yes
Murdock 2023	No	Yes	Yes	Yes	Yes	Yes	Yes	No
McGrath 2022	No	Yes	Yes	Yes	Yes	Yes	Yes	Yes
Estifanos 2023	No	No	Yes	Yes	Yes	Yes	Yes	Yes
Escudero 2020	No	No	Yes	Yes	Yes	Yes	Yes	Yes
Woodward 2023	No	No	Yes	Yes	Yes	Yes	Yes	Yes
Sibuyi 2022	No	No	Yes	Yes	Yes	Yes	Yes	Yes

Shaw 2021	No	No	Yes	Yes	Yes	Yes	Yes	Yes
Sanuade 2023	No	Yes	Yes	Yes	Yes	Yes	Yes	Yes
Pesut 2022	No	Yes	Yes	Yes	Yes	Yes	Yes	Yes
Patil 2023	No	No	Yes	Yes	Yes	Yes	Yes	Yes
Parry 2022	Yes	Yes	Yes	Yes	Yes	Yes	Yes	Yes
Ogbulafor 2023	No	Yes	Yes	Yes	Yes	Yes	Yes	Yes
Nwaozuru 2022	No	No	Yes	Yes	Yes	Yes	Yes	Yes
Nair 2021	No	No	Yes	Yes	Yes	Yes	Yes	Yes
Mooses 2021	No	No	Yes	Yes	Yes	Yes	Yes	Yes
McLaughlin 2021	No	No	Yes	Yes	Yes	Yes	Yes	Yes
Matindo 2022	No	No	Yes	Yes	Yes	Yes	Yes	Yes
MacInnes 2023	No	No	Yes	Yes	Yes	Yes	Yes	Yes
Lenton 2021	No	Yes	Yes	Yes	Yes	Yes	Yes	Yes
Kumar 2023	No	No	Yes	Yes	Yes	Yes	No	Yes
Kodish 2022	No	No	Yes	Yes	Yes	Yes	No	No
Kiracho 2021	No	Yes	Yes	Yes	Yes	Yes	Yes	Yes
Jwanle 2023	No	No	Yes	Yes	Yes	Yes	Yes	Yes
Jayanna 2023	No	No	Yes	Yes	Yes	Yes	Yes	Yes
Gaber 2022	No	Yes	Yes	Yes	Yes	Yes	Yes	Yes
Flax 2023	No	Yes	Yes	Yes	Yes	Yes	Yes	Yes
Fiori 2023	No	No	Yes	Yes	Yes	Yes	No	Yes
ElJoueidi 2021	No	No	Yes	Yes	Yes	Yes	Yes	Yes
Dickson 2023	No	No	Yes	Yes	Yes	Yes	Yes	Yes
Dev 2021	No	Yes	Yes	Yes	Yes	Yes	Yes	Yes
Corches 2020	Yes	Yes	Yes	Yes	Yes	Yes	Yes	Yes
Chowdhary 2022	No	No	Yes	Yes	Yes	Yes	Yes	Yes
Chau 2021	No	No	Yes	Yes	Yes	Yes	Yes	Yes
Chamie 2022	No	No	Yes	Yes	Yes	Yes	Yes	Yes
Bharmal 2022	No	No	Yes	Yes	Yes	Yes	Yes	Yes
Berbakov 2023	No	Yes	Yes	Yes	Yes	Yes	Yes	Yes
Barker 2023	No	Yes	Yes	Yes	Yes	Yes	Yes	Yes
Balayah 2021	No	No	Yes	Yes	Yes	Yes	Yes	Yes
Azevedo 2022	No	Yes	Yes	Yes	Yes	Yes	Yes	Yes
AsamoahAmpofo 2022	No	No	Yes	Yes	Yes	Yes	Yes	Yes
Akter 2023	No	Yes	Yes	Yes	Yes	Yes	Yes	Yes
Akinyemi 2022	No	No	Yes	Yes	Yes	Yes	Yes	Yes
