# Supplementary material for: Strategies for involving patients and the public in scaling initiatives in health and social services: A scoping review
Source: Health Expect. 2024 Jun 5;27(3):e14086. doi: 10.1111/hex.14086 (PMC11150745; doi:10.1111/hex.14086)
Supplement: Supplementary file 11 — Supporting information. [file HEX-27-e14086-s001.pdf]

Patient and public involvement in scaling in HSS Corôa et al. 2024
Additional File 11 - PPI strategies and methods

STUDY ID	DIRECT CARE								
	Patient education	Behavioural change interventions	Personalized care planning	Self management supports	Shared decision making	Access to health records or portals	Patient navigation	Family supports	Peer supports
Ashraf 2015									
Awoonor-Williams 2013									
Barber 2019									
Basso 2017	Meetings and activities to share information and promote mobilization of the population								
Bennett 2017									
Bennett 2017									
Bennett 2017									
Bradley 2012									Involve user groups' releasing and spreading the innovation for its re-introduction in new user groups within their peer networks), this means introduction of the innovation from outside the user group to inside the user group via boundary spanners, translating the innovation so that user groups could assimilate the new information
Callaghan-Koru 2020	Comprehensive social and behavioural communications approach, involving mass media and facility- and community-level providers in both public and private sectors to educate families								
Carnell 2014	Community sensitization meetings, Volunteer training and supervision, Health extension workers were trained to sensitize community leaders and to assist in selecting, training, and supervising volunteers, Review meetings conducted three months after training								
CFHI-4 2021	Self-management education, Follow-up phone calls to track patient progress and reinforce education	Home visits with COPD patients and families	Opportunity to discuss advance care planning and complete a personal directive if desired						
CHFI-86 2017									
Chandrashekar 2014	Peer educators	Behavior change communication							Self-help groups
Chibanda 2017									
Cislaghi 2019- Intervention 1	Informal education program that makes use of participatory pedagogical strategies	Community Management Committee, a 17-person community group whose task is to implement the vision emerging from the classes in collaboration with the whole community							Class participants share their learning with peers and family members, and committee members raise awareness throughout their locality organizing community mobilization activities
Cislaghi 2019- Intervention 2	Curriculum delivered to 360 married couples via weekly facilitated Listening and Discussion Groups. Trainings for religious and community leaders	Radio program involving drama and discussion elements. Safe space for couples to critically reflect on existing harmful gender norms and renegotiate more mutually respectful relationships. Diffusion through community actions delivered by the LDGs.							Couples listening and Discussion Groups
Cislaghi 2019- Intervention 3	"Safe Space" courses	Branded communications campaign through radio discussions and dramas							
Colom 2018	During monthly visits, the loan officers introduce the healthcare initiative and provide education about the need for primary healthcar								
Desclaux 2010									
Fagg 2014	The intervention requires a parent or carer to attend sessions for activity through education and skills training	The intervention requires a parent or carer to attend sessions for motivational enhancement							
Fort 2019	Community health workers (CHW) led the home-based intervention and served as a source of education, motivation, and social support and as facilitators	Community health workers (CHW) were trained in facilitating behavioral change through monitoring, medication management, and lifestyle modification, Individualized messages (text messaging) to promote lifestyle change and reminders to reinforce medication adherence were sent out weekly							
Fuhr 2020									
Gaitonde 2020									
Ghiron 2014	Convertion of the innovation from a simple local training function into a "training of trainers" who would be in a position to expand the application of new knowledge beyond the initial project sites								
Google-18 2018									
Google-180 2020		Diary, Focus groups							
Google-182 2020									
Google-190						Care professionals, citizens and patients should be empowered to use the service, e.g. to access their health data through their personal health records			
Google-202 2020	Regular meetings for education, shared knowledge and mentorship, A website was developed to house central resources and collective knowledge								
Google-234 2022		Messaging and program content aimed at encouraging target-populagtion, Website included basic information on the approach, easy steps to begin, examples of the assessment, success stories, and a simple call to action, Dissemination strategies are a subset of implementation strategies that particularly attend to the natural communication channels of end-users and developing networks of individuals and champions, Sharing of success stories through interviews and online blogs, Video and written testimonials to share personal stories and successes							

Google-28 2016		Parent involvement and engagement events and workshops in partnership with community organizations and local businesses, Parent involvement plan, Monthly parent involvement calendar, Parent involvement log							
Google-29 2013									
Google-381 2015	Individual counselling and support focused on shift, Ensure careful selection, training, supervision and support of group facilitators,	Engage local opinion leaders strategically and purposefully through existing community structures, Locally tailored, sustained messaging		To promote greater self-efficacy individual client support should focus on skills building, problem solving and positive living					Participatory women groups
Google-40 2020	Citizen workshops								
Google-57 2016	Implementing public education programs	Enlisting prominent spokespersons or celebrities as advocates, Developing and popularizing images, slogans, and symbols, Mounting local, national, and international media, campaigns showcasing success, Sustained media coverage, Comparative scorecards							
Google-62 2014									
Held 2016	Active Dissemination: A research team member explained the study in waiting rooms in clinic settings and provided educational DVD to target population, Passive Dissemination: Along with an explanatory poster, unattended copies of the educational DVD were left in waiting rooms in clinic settings, a member of the research team monitored packets to see how many had been taken, Healthcare Provider-Mediated Dissemination: Healthcare providers of many types from clinic settings were asked to provide the educational DVD to patients in the context of their routine clinical visit, Peer-mediated Dissemination: A community-based peer support and outreach group staffed by individuals from target population offered the educational DVD to clients they interacted with by phone, in person, or at group outreach events.								
IHI-12 2020	Sharing the advantages of partner testing at gatherings and venues								A male leader sensitizing men on the advantages of HIV counseling and testing among couples, and sending invitation letters to male partners
IHI-4 2009									
IHI-6 2015									
Killingo 2017	Organizing mobilization workshops for knowledge building						Developing a treatment education toolkit,		
King 2008	Safer sex gatherings	Stage based encounters, Role model stories, Community network, Small group activities							Peer networks
Koorts 2018									
L'Engle 2017									
Mai 2019		Selection of CBDs (community-based distributors (CBDs) o) and community champions, Behavioral change training, including the training of trainers, Community outreach events							
Mendel 2008									
Moroz 2020									
NICE-167 2016									
NICE-221 2016	Support the inclusion of comprehensive sexuality education with strong gender equality components in both primary and secondary schools, Promote and provide training for educators and in- and out-of-school adolescents and young people	Focus on reaching target population with innovative service delivery methods in workplaces, places of worship, sports gatherings, schools and other community venues, Use communication and advocacy tools, social media and other media outlets (including through worker and employers unions, faith based networks, and sports, entertainment and traditional leadership structures) innovatively to challenge harmful gender norms.							
NICE-9 2021 - Intervention 1									Stories to help diagnose the wider symptoms
NICE-9 2021 - Intervention 2									
NICE-9 2021 - Intervention 3									Digital platform enables individuals to recognise and applaud each others efforts, with the intention of creating a fun, open, and supportive community not only for those receiving support but those giving it, Peer to peer leadership as healthy living champions
NICE-9 2021 - Intervention 4									Narrative focuses on the simple fact that our lives are better when they are shared, Shared Lives Week, annually celebrate and shine a spotlight on the work, helping to elevate these approaches with policy-makers, commissioners and ministers as well as reaching the general public.
NICE-9 2021 - Intervention 5									
NSW-6 2014									

Pinto 2015										Exercise coaches
Rhodes 2020										Train and support volunteers from soccer teams through the use of video vignettes
Soti-Ulberg 2020		Identify local and global champions, train and involve champions in breastfeeding-related activities, and share success stories with the community via different media outlets (e.g., TV, documentary, Radio and/or Social Media/Facebook)								
Sperber 2008									Opportunity for peers to come together in a multiple family group structure and, in so doing, to provide culturally acceptable community-level resources for one another.	
Wagner 2007	Community outreach to educate the community, Educating the community about HIV and ART and getting active support and cooperation from community leaders, Media advertising (billboards, radio advertisements), Scheduling pre-ART education sessions to coincide with medical clinic visits, rather than requiring separate visits									Trained peers/community members (in this case, market vendors) to spread HIV/ART education and mobilize the community to seek HIV testing by providing referrals to the testing center, Motivate members of the community to plan and implement ways to educate the community and mobilize their peers (e.g., community forums, town hall meetings, support groups, door-to-door campaigns, Organize clients to serve as a safety net for each other, providing support to clients who fail to show for an appointment to assure that these clients return to and are maintained in care, Clients could form support groups according to location so that when a client defaults, other clients in their group can check in on them and support them in getting back into treatment
Warren 2003	Face-to-face communication to equip potential users with information and skills, Training of public health providers, pharmacists in the private sector and local members of women's organisations as providers Involvement of local women's organisations to ensure a strong community focus and the development of an extensive network of peer educators to give a clear message from women to women	Social marketing campaign initiated by a successful social marketing organization								
WHO-11 2003										
WHO-120 2021										
WHO-169 2013	Training community health workers, Training of community members, Social marketing and demand creation									Peer support for health services and healthy behaviours
WHO-179 2012										
WHO-30 2014	Empower women through improved health education and information sharing among women and their community members	Establish or strengthen behaviour change communication initiatives including the availability of key messages in local languages, Use media and other outreach tools to communicate messages and improving participation (eg, community radio and mobile applications), discussion in community forums, Engage communities and leaders in sensitisation meetings such as town hall meetings or focus group discussions								
WHO-34 2011										
WHO-414 2018 - Intervention 1	Short training period									
WHO-414 2018 - Intervention 2										
WHO-414 2018 - Intervention 3										
WHO-553 2017										
WHO-7 2015										
WHO-8 2018		Mass media behavioural change campaigns and social marketing methods								
WHO-9 2020										
Yamey 2011										Participatory groups
Zalazar 2021										
Woodward 2023										
Puffer 2022										
Murdock 2023		Volunteers and faith leaders are trained as promoters								

McGrath 2022					Sheds were encouraged through shared decision making					
Estifanos 2023									Family discussions	Champion mothers
Escudero 2020										
Woodward 2023										
Sibuyi 2022						Subscription of users and beneficiaries to the mobile service. SMS text messaging. and service rating				
Shaw 2021										
Sanuade 2023	Education in schools	Community mobilization. and public health campaigns								
Pesut 2022							Volunteers trained to identify the day-to-day challenges persons are experiencing			
Patil 2023										
Parry 2022										
Ogbulafor 2023		Focus group discussions								
Nwaozuru 2022										
Nair 2021										
Mooses 2021	Development of students training									
McLaughlin 2021										
Matindo 2022										
MacInnes 2023										
Lenton 2021										
Kumar 2023										
Kodish 2022		Social and behavior change communication (SBCC) campaign								
Kiracho 2021										
Jwanle 2023	Community teams were deployed to conduct HIV education									
Jayanna 2023										
Gaber 2022			Trained volunteers use structured surveys to ask about clients' health needs, goals, and social context			Client responses are entered into a web-based application using a tablet computer.				
Flax 2023		Home visits. groups discussions and forums. orientations with stakeholders. community sensitization sessions. video shows in media-dark areas. village theatre. mass media and other campaign<br>materials (e.g., TV spots, radio spots, hand washing sticker mobile phone counselling sticker, newspaper inserts, child feeding bowls) Community mobilization								
Fiori 2023						Integrating the screening tool within the online patient portal	Community Health Workers (CHWs) to connect patients to essential social services			
ElJoueidi 2021						Digital health communication tool that allows patients to communicate with healthcare providers via short message service (SMS), voice and video call				
Dickson 2023										
Dev 2021										
Corches 2020		Workbook. music video. brochures. action plan cards. posters. peer-led workshops. digital, social, and broadcast media campaigns. mailers sent to residential addresses with information letter explaining the program. and social media campaign with radio stations and spoken public service announcements								
Chowdhary 2022	"Girls Groups" (GG) who received customized sexual and reproductive health (SRH) curriculum, economic empowerment (EE) curriculum, and life skills and communication training.									Girls Groups were led by trained and supported peer facilitators, not educators but learners themselves
Chau 2021										
Chamie 2022										
Bharmal 2022	The community-based sessions delivered nutrition and lifestyle-related education, provided participants with educational tools	Participants were provided customized shopping lists for cooking and menu options for eating out. a cooking demonstration session								
Berbakov 2023										
Barker 2023										
Balayah 2021										
Azevedo 2022										
AsamoahAmpofo 2022										
Akter 2023										
Akinyemi 2022										

STUDY ID	ORGANIZATION OF HEALTHCARE				
	Information campaigns & platforms	Service user needs assessment	Quality & safety assessment	Organizational advisory groups	Co-leadership in quality and safety improvement
Ashraf 2015					
Awoonor-Williams 2013					
Barber 2019					Steering group/ monthly meetings, Patient participation group—GP practice and local community - Chair of a Patient Participation Group (PPG)
Basso 2017					
Bennett 2017					
Bennett 2017					
Bennett 2017					
Bradley 2012					
Callaghan-Koru 2020					
Carnell 2014					
CFHI-4 2021		Regional roundtables	A phone number that patients and families can call to have their COPD related questions and/or concerns answered	Regional roundtables	
CHFI-86 2017				Steering committee, policy roundtable, evaluation after the roundtable	
Chandrashekar 2014					
Chibanda 2017		Needs assessment involving key stakeholders and community members, and identifying key priority areas			
Cislaghi 2019- Intervention 1					
Cislaghi 2019- Intervention 2					
Cislaghi 2019- Intervention 3					
Colom 2018		Focus groups and client surveys			
Desclaux 2010					

Fagg 2014					
Fort 2019					
Fuhr 2020					
Gaitonde 2020					
Ghiron 2014		Multiple group and individual interviews with a variety of community-based groups, to gather perspectives about whether the proposed interventions were relevant to their settings and how to ensure successful implementation			
Google-18 2018			Workshops with patient representatives, Testing of different approaches to patient/carer involvement, Inclusion of patient/carer involvement questions in post-embedded questionnaires, group interviews and second interviews or questionnaires with stakeholders		
Google-180 2020					
Google-182 2020					
Google-190			Patients (and carers) opinions during the process of procuring a service, in terms of detailing specifications and assessing equipment	Participatory meetings and events, Use the expertise of each partner in the ecosystem to analyse the local needs, Formalised agreements between parties to consolidate their common undertaking, A Joint Governing Board, or oversight group, comprising all the key stakeholders, Set-up of a representative forum of older people (or older people champions) to guarantee that the voices of older people, as well as their families and carers, are heard [Wales], a Council of Elderly People encompassing regional authorities, associations of older people and the third sector, to facilitate social participation of older people into political life of the region	
Google-202 2020				A project advisory group was formed to guide the project and the group provided advice on project implementation, evaluation, and interpretation of results	
Google-234 2022					
Google-28 2016		Family need assessment, Asset mapping,			Collaborative leadership team
Google-29 2013					
Google-381 2015					
Google-40 2020					

Google-57 2016					
Google-62 2014					
Held 2016					
IHI-12 2020					
IHI-4 2009					
IHI-6 2015				Steering Committee at the beginning of the project and less often (monthly or as needed) when implementation progressed to discuss design, decide on key operational issues, and drive implementation, Many informal communication channels to foster trust and harmony, Map out roles, responsibilities, reporting lines, etc., through formal Memoranda of Understanding, Discuss issues informally and built consensus to avert contentious emails quoting legal obligations of stakeholders	Actively included community members in Quality Improvement (QI) teams
Killingo 2017					
King 2008					
Koorts 2018					
L'Engle 2017					
Mai 2019					
Mendel 2008					
Moroz 2020					
NICE-167 2016					
NICE-221 2016					

NICE-9 2021 - Intervention 1					
NICE-9 2021 - Intervention 2					
NICE-9 2021 - Intervention 3					
NICE-9 2021 - Intervention 4					
NICE-9 2021 - Intervention 5		Consulting with volunteers and designed a digital data capture application that collects live membership data from volunteers leading the groups, Based on volunteer feedback, they also added a register function, so volunteers can track who is attending the group and reach out to those who are not Through the app, they can also get a picture of what type of external expertise groups are inviting in to support their members, for example sessions with choir facilitators or cookery instructors			
NSW-6 2014	Mobilising grass roots campaigns, Tailored stakeholder engagement, social marketing, and public relations campaigns targeting these, Use of champions to add weight to these efforts, Policy briefs, engaging the support of opinion leaders and champions to act as spokespersons for scaling up the intervention				
Pinto 2015					
Rhodes 2020					
Soti-Ulberg 2020					
Sperber 2008					
Wagner 2007					
Warren 2003					
WHO-11 2003	Well-designed communications program	Information, education, and communication activities to meet awareness and learning needs, as also process monitoring needs			

WHO-120 2021					
WHO-169 2013					
WHO-179 2012					
WHO-30 2014					
WHO-34 2011					
WHO-414 2018 - Intervention 1					
WHO-414 2018 - Intervention 2					
WHO-414 2018 - Intervention 3					
WHO-553 2017			Patient feedback captured during the 3-month (and later, the 12-month) evaluation		
WHO-7 2015					
WHO-8 2018					
WHO-9 2020					
Yamey 2011					

Zalazar 2021					
Woodward 2023					
Puffer 2022					
Murdock 2023					
McGrath 2022					
Estifanos 2023					
Escudero 2020					
Woodward 2023					
Sibuyi 2022					
Shaw 2021					
Sanuade 2023					
Pesut 2022					
Patil 2023					
Parry 2022					
Ogbulafor 2023					
Nwaozuru 2022					
Nair 2021					
Mooses 2021					
McLaughlin 2021					
Matindo 2022					
MacInnes 2023					
Lenton 2021					
Kumar 2023					
Kodish 2022					
Kiracho 2021					
Jwanle 2023					
Jayanna 2023	Champions spreading awareness through Community fora (ex. fairs). animated videos on local TV cable. radio interviews. and digital and paper media				
Gaber 2022					
Flax 2023					
Fiori 2023					
ElJoueidi 2021					
Dickson 2023					
Dev 2021					
Corches 2020					
Chowdhary 2022					
Chau 2021					
Chamie 2022					

Bharmal 2022					
Berbakov 2023		Interviews			
Barker 2023					
Balayah 2021					
Azevedo 2022			Interviews		
AsamoahAmpofo 2022					
Akter 2023					
Akinyemi 2022					

STUDY ID	PROFESSIONAL TRAINING				
	Use of patient data in training	Testimonials	Simulated patients	Patient trainers	Co-design of educational or training activities
Ashraf 2015					
Awoonor-Williams 2013					
Barber 2019				A series of training workshops were agreed and delivered	Creation of training materials containing advice on how the innovation could be used that were used in workshops and appeared on websites and in newsletters
Basso 2017					
Bennett 2017					
Bennett 2017					
Bennett 2017					
Bradley 2012					
Callaghan-Koru 2020					
Carnell 2014					
CFHI-4 2021					
CHFI-86 2017					
Chandrashekar 2014					
Chibanda 2017					
Cislaghi 2019- Intervention 1					
Cislaghi 2019- Intervention 2					
Cislaghi 2019- Intervention 3					
Colom 2018					
Desclaux 2010					

Fagg 2014					
Fort 2019					
Fuhr 2020					
Gaitonde 2020					
Ghiron 2014					
Google-18 2018					
Google-180 2020					
Google-182 2020					
Google-190					
Google-202 2020					
Google-234 2022					
Google-28 2016					
Google-29 2013					
Google-381 2015					
Google-40 2020					

Google-57 2016					
Google-62 2014					
Held 2016					
IHI-12 2020					
IHI-4 2009					
IHI-6 2015					
Killingo 2017					
King 2008					
Koorts 2018					
L'Engle 2017					
Mai 2019					
Mendel 2008					
Moroz 2020					
NICE-167 2016					
NICE-221 2016					

NICE-9 2021 - Intervention 1					
NICE-9 2021 - Intervention 2					
NICE-9 2021 - Intervention 3					
NICE-9 2021 - Intervention 4					
NICE-9 2021 - Intervention 5					
NSW-6 2014					
Pinto 2015					
Rhodes 2020					Video vignettes developed by the partnership and focused on men lived experiences were rewritten and produced for broad use within the community
Soti-Ulberg 2020					
Sperber 2008					
Wagner 2007					
Warren 2003					
WHO-11 2003					

WHO-120 2021					
WHO-169 2013					
WHO-179 2012					
WHO-30 2014					
WHO-34 2011					
WHO-414 2018 - Intervention 1					
WHO-414 2018 - Intervention 2					
WHO-414 2018 - Intervention 3					
WHO-553 2017					
WHO-7 2015					
WHO-8 2018					
WHO-9 2020					
Yamey 2011					

Zalazar 2021					
Woodward 2023					
Puffer 2022					
Murdock 2023					Committee meeting
McGrath 2022					
Estifanos 2023					
Escudero 2020					
Woodward 2023					
Sibuyi 2022					
Shaw 2021					
Sanuade 2023					
Pesut 2022					
Patil 2023					
Parry 2022					
Ogbulafor 2023					
Nwaozuru 2022					
Nair 2021					
Mooses 2021					
McLaughlin 2021					
Matindo 2022					
MacInnes 2023					
Lenton 2021					
Kumar 2023					
Kodish 2022					
Kiracho 2021					
Jwanle 2023					
Jayanna 2023					
Gaber 2022					
Flax 2023					
Fiori 2023					
ElJoueidi 2021					
Dickson 2023					
Dev 2021					
Corches 2020					
Chowdhary 2022					
Chau 2021					
Chamie 2022					

Bharmal 2022					
Berbakov 2023					
Barker 2023					
Balayah 2021					Workshops. and individual interviews
Azevedo 2022					
AsamoahAmpofo 2022					
Akter 2023					
Akinyemi 2022					

STUDY ID	RESEARCH				
	Lay scientific communications	Patient consultations	Involvement in study phases	Research advisory groups	Co-leadership in research activities
Ashraf 2015					
Awoonor-Williams 2013			Community mapping and enumeration, Outreach to traditional leaders, The team consulted chiefs and elders, married women and men, and health care providers about appropriate strategies for implementing, managing, and sustaining community-engaged primary health care, Community entry, which includes conducting meetings and diplomacy with village leaders, convening public gatherings for communicating plans and activities to communities, and constituting health liaison committees for providing daily support to the programme, Community leadership training, The project equipped volunteers with bicycles and startup kits of essential drugs, conducted training on service management, and set up revolving accounts so that the community financed and sustained the flow of supplies		
Barber 2019					
Basso 2017					
Bennett 2017			Identification and measurement of quality and performance indicators, collective development of work plans, qualitative evaluation using focus group discussions		
Bennett 2017			surveys of informal healthcare, exit interviews, household survey		
Bennett 2017			Designed as participatory action research, involving stakeholders in Susman's action research cycle; draws on routine monitoring data (from health service, project documents), household and facility surveys, and qualitative methods, e.g. focus group discussions, in-depth interviews		
Bradley 2012					
Callaghan-Koru 2020					
Carnell 2014					
CFHI-4 2021					
CHFI-86 2017					
Chandrashekar 2014					
Chibanda 2017			Engaging key stakeholders in participatory planning of mental health services through workshops, Validation of screening tools in which included translation/back-translation of tools, reviewing the gold standard, protocol training and deciding on a cut off score based on sensitivity and specific; formal and informal meetings with both communities and policymakers		
Cislaghi 2019- Intervention 1					
Cislaghi 2019- Intervention 2					
Cislaghi 2019- Intervention 3					
Colom 2018					
Desclaux 2010			Advisory committee, 4-day meeting, Field studies conducted by a team of qualified interviewers, Workshops to dissemination that encouraged the sharing of experiences and lessons learned, as well as discussion of particular cases		

Fagg 2014					
Fort 2019		Consultation workshops	Interviews and focus group discussions with different stakeholders (health area and district staff, doctors, nurses, auxiliary nurses, community members, patients, and family members)	Community advisory board	
Fuhr 2020		Patients have been interviewed in the formative research phase and findings of these qualitative interviews informed the development of the intervention			
Gaitonde 2020					
Ghiron 2014					
Google-18 2018					
Google-180 2020		Feedback form/short survey			
Google-182 2020					
Google-190					
Google-202 2020					
Google-234 2022		Focus groups and key informant interviews			
Google-28 2016					
Google-29 2013					
Google-381 2015				Working group would to be a permanent public sector unit to oversee and coordinate community-level activity	
Google-40 2020					Members of the preliminary steering committee discussed and approved the aim of the citizen workshops, Applications for the workshop were evaluated bycommittee member, Committee members participated in writing and approving the script for the workshops, Committee members were involved in all stages of the implementation, including workshop observation, data collection, and outcomes evaluation.

Google-57 2016					
Google-62 2014					
Held 2016			Focus groups was to assess awareness, knowledge and perceptions, to pilot test the educational DVD content, and obtain feedback on the various dissemination techniques used, as well as other possible strategies		
IHI-12 2020					
IHI-4 2009					
IHI-6 2015					
Killingo 2017					
King 2008					
Koorts 2018		Focus groups	Focus groups with target population to explore barriers and facilitators to intervention design at scale and fostering sustained participation, Needs assessments to address current gaps between what is available versus what is required by target population, Focus groups to assess implementation-related outcomes including barriers and facilitators		Target relevant stakeholders that have shared values and/or misson, If a lack of engagement, identify reasons and modify strategy or approach alternate groups, Engage stakeholders in a participatory research process, Identify formal or informal opportunities to gain feedback from user advisory groups
L'Engle 2017		Assessing the target audiences, information needs and		Establish mobile messaging technical working group	
Mai 2019					
Mendel 2008		Assess perceived community health needs, priorities	Personal narratives (written, video, and audio) of participation in the activities that convey the impact and methods of community engagement in ways not possible through the questionnaire surveys of participants alone		
Moroz 2020					
NICE-167 2016					
NICE-221 2016					

NICE-9 2021 - Intervention 1					
NICE-9 2021 - Intervention 2					
NICE-9 2021 - Intervention 3					
NICE-9 2021 - Intervention 4					
NICE-9 2021 - Intervention 5					
NSW-6 2014					
Pinto 2015					
Rhodes 2020			The partnership refined and validated findings (from interviews) using an approach that included three empowerment based meetings to review themes and their placement within scale-up and spread constructs, discussion, refinement, and interpretation		
Soti-Ulberg 2020				Country committee	
Sperber 2008			Focus groups that sought to identify pressing community and family needs, and to define acceptable research projects or service innovations, Community Collaborative Board, which would participate in, and share power over, the design, delivery and evaluation of the programme, Members of the community joined with programme staff to conduct semi-structured interviews with youth and caregivers, A Community Collaborative Board of influential community leaders, In the implementation phase of programme translation, the Community Board worked with investigators to tailor the curriculum to local values and needs identified in phase	Community Collaborative Board of influential community leaders, community health workers, teachers and caregivers, Community Board and staff worked with investigators to tailor the curriculum to local values and needs identified	
Wagner 2007					
Warren 2003					
WHO-11 2003					

WHO-120 2021					
WHO-169 2013					
WHO-179 2012			Identify and prioritize stakeholders, Identify opportunities to obtain stakeholder input at each stage of research while developing the research question, when assessing the overall relevance of the research, while developing the research protocol, during research fieldwork, when interpreting research findings, and as part of the dissemination and advocacy of findings, Communicate with stakeholders early in the research process to determine their interest in the research, to formulate stakeholder roles and to develop strategies for engaging stakeholders and obtaining support, Plan research budgets and timelines to accommodate stakeholder input, Organize participatory face-to-face meetings with the target audience, for example, hold a dissemination workshop where research findings are presented and stakeholders participate in interpreting the findings and developing specific recommendations or action plans for their use, Present the research findings in a way that considers the needs of different audiences, which may include the development of targeted, actionable messages in nonacademic language, Messages should be tailored to each stakeholder based on the types of decisions they make and the environments in which they work, Consider using champions to disseminate research results or evidence-based practices, Enhance the experiences of certain audiences by organizing site tours or using film and other media to convey information, Involve champions in developing an advocacy work plan, and		
WHO-30 2014					
WHO-34 2011					
WHO-414 2018 - Intervention 1					
WHO-414 2018 - Intervention 2			Use of locally relevant visual and audio material for acceptance by communities		
WHO-414 2018 - Intervention 3			Conducting formative research with community leaders, and the innovations were designed accordingly, Involve working with community'opinion leaders', individuals and organisations able to precipitate in the diffusion of ideas including community groups, women'sgroups, traditional and religious leaders, churches and mosques, Stimulating the diffusion of innovations through mass and local media and by word of mouth, Community mobilisation teams to improve relationships between communities and health professionals, or trained role models to spread ideas		
WHO-553 2017					
WHO-7 2015			Cultivating champions in different partner organizations	Establishment of effective governance structures involving a representative steering committee or decision-making board, with a regular schedule of meetings, and mechanisms for transparency and accountability	
WHO-8 2018				Securing community ownership for intervention, Work with local grassroots organisations, Understanding contextual factors and involving local stakeholders, Involving multiple stakeholders to mobilise communities and foster sustainability, Place community representatives at the forefront	
WHO-9 2020		Consult with different user groups and potential beneficiaries before defining the research question(s)	Involve stakeholders appropriately in data collection, analysis, synthesis and interpretation, Communicating research results using existing structures, Consider which strategies will facilitate stakeholders to take action based upon the research findings, Consider what results could be useful or influential to a broader range of stakeholders		
Yamey 2011					

Zalazar 2021					
Woodward 2023		Open-ended or "fill in the blank, then elaborate" questions. one-time anonymous survey			Workgroup meeting
Puffer 2022		Semi-structured focus group discussions. post-session surveys online			Virtual Intervention Development Workshop
Murdock 2023					
McGrath 2022		Focus groups. interviews. observations. questionnaires. and administrative data		Participatory Research Approach	
Estifanos 2023					
Escudero 2020		Focus groups discussions			
Woodward 2023					
Sibuyi 2022					
Shaw 2021					
Sanuade 2023					
Pesut 2022					
Patil 2023					
Parry 2022					Identifying research priorities. designing the project for wider effects and reach. developing the funded grant proposal, co-designing. co-delivering all webinar presentations. collecting, analyzing and interpreting interview data. disseminating results through a presentation and a publication. and making recommendations for further refinements to the design of each decision aid.
Ogbulafor 2023					
Nwaozuru 2022					
Nair 2021					
Mooses 2021		Focus group interviews			
McLaughlin 2021		Think-aloud survey. and usability test			
Matindo 2022		Interviews			
MacInnes 2023		Interviews. and focus groups			
Lenton 2021					
Kumar 2023					
Kodish 2022					
Kiracho 2021		Community level assessment	Cluster consolidation meeting. interface meeting. focus group discussions	Meetings	
Jwanle 2023					
Jayanna 2023					
Gaber 2022					
Flax 2023		Key informant interviews			
Fiori 2023					
ElJoueidi 2021					
Dickson 2023		Interviews. and focus groups			
Dev 2021					
Corches 2020		Survey. and focus groups			Community advisory board (CAB), co-chaired by the academic and community principal investigators (PIs)
Chowdhary 2022					
Chau 2021			The social collaborators were trained as part of the MAC-FI study to deliver SSM		
Chamie 2022					Community-academic "Latino COVID-19 Collaborative" (LCC) that met monthly to discuss barriers and facilitators and to plan mass testing events. Mass testing events with LCC input

Bharmal 2022		Focus groups and interviews. and surveys			
Berbakov 2023			Meetings		
Barker 2023					
Balayah 2021					
Azevedo 2022				Meetings	
AsamoahAmpofo 2022					
Akter 2023		Interviews. and focus groups			
Akinyemi 2022					

STUDY ID	POLICYMAKING		
	Population consultations	Policy advisory groups	Co-leadership in policymaking
Ashraf 2015		Interviews, Input in the meeting agenda, Multi-stakeholder dialogues (MSD) day-long	
Awoonor-Williams 2013			
Barber 2019			
Basso 2017	Survey		
Bennett 2017			
Bennett 2017			
Bennett 2017			
Bradley 2012			
Callaghan-Koru 2020		Working Committee	
Carnell 2014		Health extension workers led monthly meetings with volunteers in their communities to review efforts and add new themes	
CFHI-4 2021			
CHFI-86 2017			
Chandrashekar 2014			

Chibanda 2017			
Cislaghi 2019- Intervention 1			
Cislaghi 2019- Intervention 2			
Cislaghi 2019- Intervention 3			
Colom 2018			
Desclaux 2010			
Fagg 2014			
Fort 2019			
Fuhr 2020		Theory of Change (ToC) workshop	
Gaitonde 2020		Committee meetings, regular training, and six-monthly monitoring and planning activities	
Ghiron 2014	Interviews that gave the team insight into areas where community members' knowledge and capacity could be mobilized	Multi-sectoral steering committees comprised of representatives of the line ministries, district officials representing health and the environment, NGOs, universities and others	
Google-18 2018			
Google-180 2020			

Google-182 2020	Consultation forums, Key-informants, Needs assessments with the participation of the targeted groups, Surveys that include open-ended questions at the end formore critical feedback, Provision of anonymous feedback mechanisms, Consultation of target population to assess risks and identify possible solutions	Regular follow-up meetings, Focus groups discussion and participatory assemblies to review the different stages of the intervention	Community steering committees included in the decision-making processes of the project before it starts, Information and involvement of targeted group leaders to ensure community participation regarding the implementation and monitoring of the scaling, Definition of expected outcomes by the target groups and evaluation of the results
Google-190			
Google-202 2020			
Google-234 2022			
Google-28 2016			
Google-29 2013	Check if any baseline studies (such as household surveys) have been conducted among the target populations and localities to identify target population need, Regularly report/update collected data to local and national bodies, communities/beneficiaries and partners		
Google-381 2015	Assess the local context to inform a relevant involvement strategy, Conduct participatory formative research to ensure that interventions and messages are relevant for addressing local barriers, Adopt a participatory approach to define indicators of success and establish monitoring systems before implementing a community leader intervention.		

Google-40 2020			
Google-57 2016	Citizen oversight panels, Market research studies, Open-access websites	Enlisting prominent spokespersons or celebrities as advocates, Developing and popularizing images, slogans, and symbols, Mounting local, national, and international media, campaigns showcasing success, Sustained media coverage, Comparative scorecards	
Google-62 2014		Engagement in the beginning, Ongoing delivery of project management and communications, Iterative approaches to technology design and program design, Invite stakeholders to dialogues	
Held 2016			
IHI-12 2020		Engagement of community groups to support the work of home-based care volunteers and to constitute a community improvement team that identified barriers to retention and developed and tested locally feasible strategies to bridge gaps	
IHI-4 2009		Model of primary health care that maximized community involvement in planning and implementation, included a mechanism for volunteer contributions and made use of decentralized resources.	
IHI-6 2015			
Killingo 2017			

King 2008			
Koorts 2018			
L'Engle 2017			
Mai 2019	Development of assessment tools for a predominantly qualitative and participatory approach, In-depth interviews, Focus group discussions with members of the community		
Mendel 2008			
Moroz 2020	Identify local needs through wait time studies and engagement of the patient and provider community to identify priority areas specific to that region	Policy Forum with small group sessions and patient panel reflections, Clear governance in order to decide what team members or groups would be responsible for making different decisions and oversee strategic and operational components of the service, Patient partners	
NICE-167 2016	Patient groups to provide feedback on programme performance, these groups will increase knowledge and access to improved health service delivery	Advisory committee to develop the draft action plan, which was reviewed at a consultation meeting with representatives	
NICE-221 2016			Build collaboration at all levels and provide leadership and support for engaging men and adolescent boys to challenge harmful gender norms and improve their health-seeking behaviours.
NICE-9 2021 - Intervention 1			
NICE-9 2021 - Intervention 2			Following evaluation and learning from the first stage of replication, the team looked to establish a much greater role for co-production and community leadership to guide the approach
NICE-9 2021 - Intervention 3			
NICE-9 2021 - Intervention 4			

NICE-9 2021 - Intervention 5			
NSW-6 2014	Formative evaluation prior to scale up to test the appropriateness and acceptability of the intervention with the target audience and other stakeholders	Consult with stakeholders, this can be done by organising stakeholder dialogues, working through peak bodies, non-government organisations, or social institutions, Input into policy and budgetary, Establishing commissions and advisory boards made up of key influencers, processes	
Pinto 2015			
Rhodes 2020			
Soti-Ulberg 2020			
Sperber 2008			
Wagner 2007			

Warren 2003	Quantitative and qualitative data from a sample of sites to examine the relationship between characteristics of women using female condoms and use patterns, and to assess the influence of the service delivery system on female condom uptake, Acceptability study		
WHO-11 2003			Participatory appraisal and planning; Participatory workshops; Particpatory implementation, operation and maintenance; co-financing
WHO-120 2021	Worksheets as templates for collecting data and generating insights about the actions (many ways to use these tools, individually, in pairs, as a group, in a workshop, in person, virtually, and so on)		
WHO-169 2013		Community boards and structures to oversee and manage,	Community-managed services, Community partnerships and co-management, Community-owned services
WHO-179 2012			
WHO-30 2014		Involve community members and leaders in planning and implementation of activities, Use existing community systems, structures, and initiatives (village health committees, community health committees, traditional leaders), Workshops	

WHO-34 2011	Field visits and stakeholder interviews to understand the local context and local perspectives, Discussions with providers, program managers, community members and clients to understand how the project is implemented on the ground	Two-day strategy development workshop	
WHO-414 2018 - Intervention 1			
WHO-414 2018 - Intervention 2			
WHO-414 2018 - Intervention 3			
WHO-553 2017			
WHO-7 2015			
WHO-8 2018			
WHO-9 2020			
Yamey 2011		Committee	
Zalazar 2021	Interviews		
Woodward 2023			
Puffer 2022			
Murdock 2023			Committees
McGrath 2022			
Estifanos 2023			
Escudero 2020			

Woodward 2023	Semi-structured discussion guide with open-ended questions		
Sibuyi 2022	Semistructured face-to-face interviews. focus group discussion	Task team meetings	
Shaw 2021	Interviews. focus groups		
Sanuade 2023	Semi-structured interviews		
Pesut 2022			
Patil 2023			A channel of communication was established through platforms such as WhatsApp and regular Zoom meetings
Parry 2022			
Ogbulafor 2023	In-depth Interviews		
Nwaozuru 2022	Cross-sectional qualitative survey		
Nair 2021			Group meetings cycle. semi-structured interviews. focus group discussions
Mooses 2021			
McLaughlin 2021			
Matindo 2022			
MacInnes 2023			
Lenton 2021	Face-to-face interviews. telephone interviews. and focus groups		
Kumar 2023	Focus group discussions		
Kodish 2022		Volunteers group	
Kiracho 2021			
Jwanle 2023		Consortium	
Jayanna 2023	Focus group discussions. and in-depth interviews	Consultations. meetings. and workshops	
Gaber 2022	Focus groups. Interviews. and narratives from key informants		
Flax 2023			
Fiori 2023			
ElJoueidi 2021	Survey. focus group discussions. and semi-structured informal interviews		
Dickson 2023			

Dev 2021	Qualitative interviews		
Corches 2020			
Chowdhary 2022	Semi-structured focus group discussions. in-depth interviews. field notes. and daily summaries		
Chau 2021	Semi-structured interviews		
Chamie 2022			
Bharmal 2022			
Berbakov 2023			
Barker 2023			Advisory committee
Balayah 2021			
Azevedo 2022			
AsamoahAmpofo 2022	Face to face in-depth interviews		
Akter 2023			
Akinyemi 2022	Interviews		
