# Supplementary material for: Strategies for involving patients and the public in scaling initiatives in health and social services: A scoping review
Source: Health Expect. 2024 Jun 5;27(3):e14086. doi: 10.1111/hex.14086 (PMC11150745; doi:10.1111/hex.14086)
Supplement: Supplementary file 12 — Supporting information. [file HEX-27-e14086-s010.docx]

**Additional File 12 – Patient and public profiles described**

**1. Patients**

Representatives

Patients

Families

**2. Public**

**2. 1 Targeted-groups and/or beneficiary populations**

Parents or carers

Students

Teachers

Key-informants

Business leaders, small business owners, employees, chambers of commerce, insurers, brokers

Women

Librarians

Veterans

Male partners

Vulnerable populations

Traditional Birth Attendants

Private transport operators

Young women

Women and men who practise high risk sexual

Men and adolescent boys

Adolescents and young people

Poor populations

Smallholder farmers

**2.2 Community**

Community members

Local people

Community workers

**2.3 Civil society organizations**

Non-governmental organization working with communities or citizens

Advocacy groups

Not specified

Representative organizations

Non-profit organizations

Non-partisan monitoring organizations

**2.4 Leaders**

Civil society leaders

Opinion leaders

Gender activists

Youth leader

Champions

Community leaders

**2.5. Users**

Clients

Service users

Target user

Individual Consumers

**2.6 Citizens**

Any individual described as citizen

**2.7 Volunteers**

Any individual described as volunteer
