# Supplementary material for: Strategies for involving patients and the public in scaling initiatives in health and social services: A scoping review
Source: Health Expect. 2024 Jun 5;27(3):e14086. doi: 10.1111/hex.14086 (PMC11150745; doi:10.1111/hex.14086)
Supplement: Supplementary file 13 — Supporting information. [file HEX-27-e14086-s002.docx]

Additional File 12 – Involvement Framework

| **Study** | **Involvement Framework** | |
| --- | --- | --- |
| Awoonor-Williams 2013 | Traditional forms of governance, consensus building, and volunteerism | Awoonor-Williams JK, Sory EK, Nyonator FK, Phillips JF, Wang C, Schmitt ML. Lessons learned from scaling up a community-based health program in the Upper East Region of northern Ghana. Glob Health Sci Pract. 2013 Mar 21;1(1):117-33. doi: 10.9745/GHSP-D-12-00012. PMID: 25276522; PMCID: PMC4168550. |
| Basso 2017 | Framework of an Intersectoral Approach with and without community participation | Basso, C., García da Rosa, E., Lairihoy, R., Caffera, R. M., Roche, I., González, C., da Rosa, R., Gularte, A., Alfonso-Sierra, E., Petzold, M., Kroeger, A., & Sommerfeld, J. (2017). Scaling Up of an Innovative Intervention to Reduce Risk of Dengue, Chikungunya, and Zika Transmission in Uruguay in the Framework of an Intersectoral Approach with and without Community Participation. The American journal of tropical medicine and hygiene, 97(5), 1428–1436. https://doi.org/10.4269/ajtmh.17-0061 |
| Bradley 2012 | AIDED model | Bradley EH, Curry LA, Taylor LA, et al. A model for scale up of family health innovations in lowincome and middle-income settings: a mixed methods study. BMJ Open 2012;2: e000987.doi:10.1136/bmjopen-2012-000987. |
| Callaghan-Koru 2020 | WHO/ExpandNet Model | ExpandNet, World Health Organization. Nine steps for developing a scaling-up strategy. Geneva: WHO; 2010. |
| Carnell 2014 | ‘Three Pillars’ Approach to Accelerating MDG 4 Progress in Ethiopia | Carnell, M. A., Dougherty, L., Pomeroy, A. M., Karim, A. M., Mekonnen, Y. M., & Mulligan, B. E. (2014). Effectiveness of scaling up the 'three pillars' approach to accelerating MDG 4 progress in Ethiopia. Journal of health, population, and nutrition, 32(4), 549–563. |
| Chibanda 2017 | Theory of change (ToC) approach | De Silva MJ, Breuer E, Lee L, Asher L, Chowdhary N, Lund C, Patel V (2014). Theory of change: a theory-driven approach to enhance the Medical Research Council’s framework for complex interventions. Trials 15, 267. |
| Gaitonde 2020 | Atun’s framework | Atun R, De Jongh T, Secci F, Ohiri K, Adeyi O. Integration of targeted health interventions into health systems: a conceptual framework for analysis. Health Policy Plan. 2010;25:104–11. |
| Ghiron 2014 | WHO/ExpandNet Model | ExpandNet, World Health Organization. Nine steps for developing a scaling-up strategy. Geneva: WHO; 2010. |
| Google-180 2020 | SISCODe co-design for society in innovation and science | SISCODE. SISCODe co-design for society in innovation and science: Assessment Report. Ares: European Union’s Horizon 2020; 2021 Apr. |
| Google-182 2020 | Equity and Inclusion in Policy Processes (EquIPP) | Huss, T., & MacLachlan, M. (2016). Equity and Inclusion in Policy Processes (EquIPP): A framework to support equity & inclusion in the process of policy development, implementation and evaluation. Dublin: Global Health Press. |
| Google-190 | Adapted from an original INTERREG IVC Programme table | INTERREG IVC, Study on Exchange of Experience Processes, Final Report January 2013 |
| Google-202 2020 | Consolidated framework for implementation research (CFIR framework) | Damschroder LJ, Aron DC, Keith RE et al.Fostering implementation of health services research findings into practice: a consolidated framework for advancing implementation science. Implementation Sci 2009;4(50). doi:10.1186/1748-5908-4-50 |
| Google-28 2016 | ChildParent Center Preschool model (CPC model) | Hayakawa M, Reynolds A. Strategies for Scaling Up: Promoting Parent Involvement through Family-School-Community Partnerships. Voices in Urban Education, n44 p45-52. 2016. |
| Google-29 2013 | Setting Up a Monitoring and Evaluation System: A Sample Checklist for Scaling Up Programmes | United Nations Development Programme. Guidance Note: Scaling Up Development Programmes. 2013 |
| Google-381 2015 | Conceptual Model for the Promising Practices | UNICEF. Community-facility linkages to support the scale up of lifelong ART for pregnant and breastfeeding women living with HIV: A conceptual framework, compendium of promising practices and key operational considerations. 2015 |
| Google-40 2020 | Integrated knowledge translation approach (iKT) | Bowen S.J., Graham I.D. 2013. “Integrated Knowledge Translation.” In Straus S.E., Tetroe J., Graham I.D. (Eds.) pp. 14–23, Knowledge Translation in Health Care: Moving from Evidence to Practice. Chichester, UK: John Wiley & Sons; pp. 14–23. |
| Google-57 2016 | Scaling Up Management (SUM) Framework | Management Systems International. Scaling Up-From Vision to Large-Scale Change: A Management Framework for Practitioners. Third Edition, 2016 |
| Google-62 2014 | Vital Wave Consulting | The mHealth Alliance and Vital Wave Consulting. Sustainable Financing for Mobile Health (mHealth): Options and opportunities for mHealth financial models in low- and middle-income countries. Washington, DC: The mHealth Alliance and Vital Wave Consulting; February 2013. |
| IHI-12 2020 | Community Health System Strengthening model | USAID ASSIST Project. 2020. USAID Applying Science to Strengthen and Improve Systems (ASSIST) Project. Final Report. Published by the USAID ASSIST Project. Chevy Chase, MD: University Research Co., LLC (URC). |
| Killingo 2017 | Continuum of Prevention, Care, and Treatment (CoPCT) Cascade | USAID – Linkages Across the Continuum of HIV Services for Key Populations. HIV cascade framework for key populations. 2015 |
| King 2008 | Transtheoretical model of behavior change (known as stages of change); Diffusion of innovations Theory; Social learning theory | Prochaska JO, DiClemente CC. Stages and processes of selfchange of smoking: Toward an integrative model of change. J Consult Clin Psychol 1983;51:390. ;  Rogers EM. Diffusion of innovations. New York: The Free Press, 1962. ;  Rogers EM. Diffusion of innovations, 5th ed. New York: Simon & Schuster Adult Publishing Group, 2003;  Bandura A. Social learning theory. New York: General Learning Press, 1977. |
| Koorts 2018 | PRACTIS guide (PRACTical planning for Implementation and Scale-up) | Koorts, H., Eakin, E., Estabrooks, P. et al. Implementation and scale up of population physical activity interventions for clinical and community settings: the PRACTIS guide. Int J Behav Nutr Phys Act 15, 51 (2018). https://doi.org/10.1186/s12966-018-0678-0 |
| L'Engle 2017 | mHealth Adaptation Model | L'Engle, K., Plourde, K. F., & Zan, T. (2017). Evidence-based adaptation and scale-up of a mobile phone health information service. mHealth, 3, 11. https://doi.org/10.21037/mhealth.2017.02.06 |
| Mai 2019. | WHO/ExpandNet Model | World Health Organization & ExpandNet. (‎2011)‎. Beginning with the end in mind: planning pilot projects and other programmatic research for successful scaling up. World Health Organization. https://apps.who.int/iris/handle/10665/44708 |
| Mendel 2008 | Framework of Dissemination in Health Services Intervention Research | Mendel P, Meredith LS, Schoenbaum M, Sherbourne CD, Wells KB. Interventions in Organizational and Community Context: A Framework for Building Evidence on Dissemination and Implementation in Health Services Research. Administration and Policy in Mental Health and Mental Health Services Research. 2007 Nov 8;35(1-2):21–37. |
| NSW-6 2014 | Increasing the scale of population health interventions: A Guide | Centre for Epidemiology and Evidence. Milat AJ, Newson R, and King L. Increasing the scale of population health interventions: A guide. Evidence and Evaluation Guidance Series, Population and Public Health Division. Sydney: NSW Ministry of Health, 2014. |
| Soti-Ulberg 2020 | Becoming Breastfeeding Friendly (BBF) Toolbox | Pérez-Escamilla R, Hromi-Fiedler AJ, Gubert MB, Doucet K, Meyers S, Dos Santos Buccini G. Becoming Breastfeeding Friendly Index: Development and application for scaling-up breastfeeding programmes globally. Matern Child Nutr. 2018;14(3):e12596. ;  Pérez-Escamilla R, Curry L, Minhas D, Taylor L, Bradley E. Scaling up of breastfeeding promotion programs in low- and middle-income countries: the “breastfeeding gear” model. Adv Nutr. 2012;3(6):790 –800. ;  Hromi-Fiedler AJ, Dos Santos BG, Gubert MB, Doucet K, Perez-Escamilla R. Development and pretesting of “Becoming Breastfeeding Friendly”: Empowering governments for global scaling up of breastfeeding programmes. Matern Child Nutr. 2018;15:e12659. |
| Sperber 2008 | CHAMP (Collaborative HIV Prevention & Adolescent Mental Health Project) approach | Elizabeth Sperber, Mary M. MCKay , Carl C. Bell , Inge Petersen , Arvin Bhana & Roberta Paikoff (2008) Adapting and disseminating a community-collaborative, evidencebased HIV/AIDS prevention programme: Lessons from the history of CHAMP, Vulnerable Children and Youth Studies, 3:2, 150-158, DOI: 10.1080/17450120701867561 |
| Warren 2003 | The Female Condom: A Guide for Planning and Programming | World Health Organization/ UNAIDS. The Female Condom: A Guide for Planning and Programming. Geneva: UNAIDS/WHO, 2000 |
| WHO-11 2003 | Scaling Up Community-Driven Development: Theoretical Underpinningsand Program Design Implication | P. Binswanger H, S. Azyar S. Scaling Up Community-Driven Development: Theoretical Underpinnings and Program Design Implications. The World Bank Africa Regional Office Office of the Vice President; 2003. |
| WHO-120 2021 | Tools from INSPIRE Guide | INSPIRE Working Group. (2021) INSPIRE Guide to Adaptation and Scale-Up. New York: INSPIRE Working Group; 2021 |
| WHO-179 2012 | Eight Strategies for Research to Practice | Eight Strategies for Research to Practice. MOVING EVIDENCE INTO ACTION. FHI 360. 2012. |
| WHO-34 2011 | WHO/ExpandNet Model | ExpandNet, World Health Organization. Nine steps for developing a scaling-up strategy. Geneva: WHO; 2010 |
| WHO-8 2018 | WHO/ExpandNet Model | ExpandNet, World Health Organization. Nine steps for developing a scaling-up strategy. Geneva: WHO; 2010 |
| WHO-9 2020 | Scaling playbook: a practical guide for researchers | Price-Kelly, van Haeren & McLean (2020) The Scaling Playbook. International Development Research Centre, Ottawa, Canada. |
| Yamey 2011 | Scaling Up Global Health Interventions: A Proposed Framework for Success | Yamey G (2011) Scaling Up Global Health Interventions: A Proposed Framework for Success. PLoS Med 8(6): e1001049. doi:10.1371/journal.pmed.1001049 |
| Woodward 2023 | ENGAGED for CHANGE framework | Rhodes S. Engaged for change: a community-engaged process for developing interventions to reduce health disparities. AIDS Educ Prev. (2017) 29(6):491. doi: 10.1521/aeap.2017.29.6.49 |
| McGrath 2022 | PRACTical planning for Implementation and Scale- up guide (PRACTIS) | Koorts H, Eakin E, Estabrooks P, Timperio A, Salmon J, Bauman A. Implementation and scale up of population physical activity interventions for clinical and community settings: the PRACTIS guide. Int J Behav Nutr Phys Act. (2018) 15:51. doi: 10.1186/s12966-018-0678-0 |
| Estifanos 2023 | Optimised Kangaroo Mother Care (KMC) scale-up  model | Estifanos AS, Haile Mariam D, Fikre A, Kote M, Tariku A, Chan GJ. Implementation science to design, test and scale up effective Kangaroo Mother Care in Oromia region, Ethiopia. Acta Paediatr. 2023;112(Suppl. 473):56–64. https://doi.org/10.1111/apa.16413 |
| Pesut 2022 | A practical  program to support a public health/compassion- ate community (PHCCA) approach to care | Kellehear A. Compassionate communities: end- of-life care as everyone’s responsibility. QJM 2013; 106: 1071–1075. |
| Parry 2022 | Strategy for Patient-Oriented Research (SPOR) capacity  development framework | CIHR 2015a) and the SPOR Patient  Engagement Framework (CIHR 2015b). |
| Ogbulafor 2023 | Health belief model | Janz NK, Becker MH. The health belief model: a decade later. Health Educ  Q. 1984;11:1–47. |
| Matindo 2022 | Spider gram model of participation | Chilaka MA. Ascribing quantitative value to community participation: A case study of the Roll Back Malaria (RBM) initiative in five African countries. Public Health. 2005; 119: 987–994. https://doi.org/10. 1016/j.puhe.2005.08.010 PMID: 16188287 |
| Kiracho 2021 | Theory of change; Expand Net framework; FHS project institutionalization framework | (see Fig. 2), whose development was guided by previ- ously published scaling up frameworks [17, 18, 30–33], in particular the [34], the FHS project institutionalization framework [35] as well as project wide discussions |
| Gaber 2022 | Health TAPESTRY | . ngin D, Lamarche L, Oliver D, Bomze S, Borhan S, Browne T, et al.  Health TAPESTRY Ontario: protocol for a randomized controlled trial to  test reproducibility and implementation. Trials. 2020;21(1):714. 6. Dolovich L, Oliver D, Lamarche L, Thabane L, Valaitis R, Agarwal G, et al.  Combining volunteers and primary care teamwork to support health  goals and needs of older adults: a pragmatic randomized controlled trial. |
| Flax 2023 | A&T programme conceptual model for improving IYCF practices | Flax, V. L., Bose, S., Escobar‐DeMarco, J., & Frongillo, E. A. (2023). Changing maternal, infant and young child nutrition practices through social and behaviour change interventions implemented at scale: Lessons learned from Alive & Thrive. Maternal & Child Nutrition, e13559. https://doi.org/10.1111/mcn.13559 |
| ElJoueidi 2021 | Consolidated framework for implementation  research (mCFIR) | Damschroder LJ, Aron DC, Keith RE, Kirsh SR, Alexander JA, Lowery JC. Fostering implementation of health services research findings into prac- tice: a consolidated framework for advancing implementation science. Implement Sci. 2009;4(1):50. |
| Corches 2020 | Social Cognitive Theory (SCT) | Israel BA, Schulz AJ, Parker EA, Becker AB. Review of community-based research: assessing  partnership approaches to improve public health.; e focused on the Social Cognitive Theory (SCT) construct of  self-efficacy, which is an individual’s belief in their ability to perform specific behaviors. |
| Chowdhary 2022 | CARE’s Village Savings and Loan Association (VSLA) model | Chowdhary, P., Mekuria, F.T., Tewahido, D. et al. Building sustainable and scalable peer-based programming: promising approaches from TESFA in Ethiopia. Reprod Health 19 (Suppl 1), 55 (2022). https://doi.org/10.1186/s12978-021-01304-7 |
| Berbakov 2023 | Participatory design apporach | Berbakov ME, Hoffins EL, Stone JA, Gilson AM, Chladek JS, Watterson TL, Lehnbom EC, Moon J, Holden RJ, Jacobson N, Shiyanbola OO, Welch LL, Walker KD, Gollhardt JD, Chui MA. Adapting a community pharmacy intervention to improve medication safety. J Am Pharm Assoc (2003). 2024 Jan-Feb;64(1):159-168. doi: 10.1016/j.japh.2023.11.009. Epub 2023 Nov 7. PMID: 37940099; PMCID: PMC108726 |
| Barker 2023 | Theory of Change. | The Learning Collaborative to Advance Normative Change. Identifying and Describing Approaches and Attributes of Norms- Shifting Interventions. Georgetown University, Institute for Reproductive Health; 2017. |
| Azevedo 2022 | Pine;  models for Participatory Action Research in Organizations;  Farmer Participatory Research | Selener D. Farmer-to-farmer extension: lessons from the field. Quito: IIRR (1997). ; Pine G. Teacher action research: building knowledge democracies. California: Sage (2009). |
| Akter 2023 | Participatory Learning and Action (PLA) groups | Gram L, Desai S, Prost A. Classroom, club or collective? Three types of community‐based group intervention and why they matter for health. BMJ Glob Health. 2020;5(12): e003302. |
