# Supplementary material for: Strategies for involving patients and the public in scaling initiatives in health and social services: A scoping review
Source: Health Expect. 2024 Jun 5;27(3):e14086. doi: 10.1111/hex.14086 (PMC11150745; doi:10.1111/hex.14086)
Supplement: Supplementary file 14 — Supporting information. [file HEX-27-e14086-s011.pdf]

Patient and public involvement in scaling in HSS Corôa et al. 2024
Additional File 14 - PROGRESS

Study ID	Place of	Race/ethnicit	Occupation	Gender	Sex at birth	Religion	Education	Socioecono	Social capital	Age	Disability	Sexual
Ashraf 2015	Not reported	Yes - specify	Not reported	Woman	Not reported	Not reported	Not reported	Not reported	Not reported	Not reported	Not reported	Not reported
Awoonor-Williams 2013 (related to WHO-189, WHO-03, Krumholz 2015)	Rural	Not reported	Not reported	Not reported	Not reported	Not reported	Not reported	Not reported	Not reported	Not reported	Not reported	Not reported
Barber 2019	Not reported	Yes - specify	Not reported	Woman	Not reported	Not reported	Not reported	Not reported	Not reported	Not reported	Not reported	Not reported
Basso 2017	Urban	Not reported	Not reported	Not reported	Not reported	Not reported	Not reported	Yes - specify	Not reported	Not reported	Not reported	Not reported
Bennett 2017	Lower	Yes - specify	Not reported	Not reported	Not reported	Not reported	Not reported	Not reported	Not reported	Not reported	Not reported	Not reported
Bradley 2012 (Related to WHO-13)	Not reported	Yes - specify	Yes - specify	Woman	female	Not reported	Not reported	Not reported	Not reported	Not reported	Not reported	Not reported
Callaghan-Koru 2020	Lower	Yes - specify	Not reported	Not reported	Not reported	Not reported	Not reported	Not reported	Not reported	Not reported	Not reported	Not reported
Carnell 2014	Urban	Not reported	Not reported	Not reported	Not reported	Not reported	Not reported	Not reported	Not reported	Not reported	Not reported	Not reported
CFHI-4 2021	Not reported	Yes - specify	Not reported	Not reported	Not reported	Not reported	Not reported	Not reported	Not reported	Not reported	Not reported	Not reported
CHFI-86 2017	Not reported	Yes - specify	Not reported	Not reported	Not reported	Not reported	Not reported	Not reported	Not reported	Not reported	Not reported	Not reported
Chandrashekar 2014	Not reported	Yes - specify	Not reported	Not reported	Not reported	Not reported	Not reported	Not reported	Not reported	Not reported	Not reported	Not reported
Chibanda 2017 (related to Chibanda 2016)	Rural	Yes - specify	Not reported	Woman	Not reported	Not reported	Not reported	Not reported	Not reported	Not reported	Not reported	Not reported
Cislaghi 2019	Not reported	Yes - specify	Not reported	Not reported	Not reported	Not reported	Not reported	Not reported	Not reported	Not reported	Not reported	Not reported
Colom 2018	Rural	Yes - specify	Not reported	Not reported	Not reported	Not reported	Not reported	Not reported	Not reported	Not reported	Not reported	Not reported
Desclaux 2010	Lower	Yes - specify	Not reported	Not reported	Not reported	Not reported	Not reported	Not reported	Not reported	Not reported	Not reported	Not reported
Fagg 2014	Urban and	Not reported	Yes - specify	Not reported	Not reported	Not reported	Not reported	Not reported	Not reported	Not reported	Not reported	Not reported
Fort 2019	Lower	Yes - specify	Not reported	Not reported	Not reported	Not reported	Not reported	Yes - specify	Not reported	Not reported	Not reported	Not reported
Fuhr 2020	Urban	Yes - specify	Not reported	Woman	female	Not reported	Not reported	Not reported	Not reported	Not reported	Not reported	Not reported
Gaitonde 2020	Not reported	Yes - specify	Not reported	Not reported	Not reported	Not reported	Not reported	Not reported	Not reported	Not reported	Not reported	Not reported
Ghiron 2014	Not reported	Yes - specify	Not reported	Not reported	Not reported	Not reported	Not reported	Not reported	Not reported	Not reported	Not reported	Not reported
Google-18 2018	Not reported	Yes - specify	Yes - specify	Not reported	Not reported	Not reported	Not reported	Not reported	Not reported	Not reported	Not reported	Not reported
Google-180 2020	Rural	Yes - specify	Not reported	Not reported	Not reported	Not reported	Not reported	Not reported	Not reported	Not reported	Not reported	Not reported
Google-182 2020	Not reported	Yes - specify	Not reported	Woman	Not reported	Not reported	Not reported	Not reported	Not reported	Not reported	Not reported	Heterosexual
Google-190	Not reported	Yes - specify	Not reported	Not reported	Not reported	Not reported	Not reported	Not reported	Not reported	Not reported	Not reported	Not reported
Google-202 2020	Lower	Yes - specify	Not reported	Not reported	Not reported	Not reported	Not reported	Not reported	Not reported	Not reported	Not reported	Not reported
Google-234 2022	Not reported	Not reported	Not reported	Woman	Not reported	Not reported	Not reported	Not reported	Not reported	Not reported	Not reported	Not reported
Google-28 2016	Not reported	Yes - specify	Not reported	Woman	Not reported	Not reported	Not reported	Not reported	Not reported	Youth (15-24	Not reported	Not reported
Google-29 2013	Lower	Yes - specify	Not reported	Woman	Not reported	Not reported	Not reported	Not reported	Not reported	Not reported	Not reported	Not reported
Google-381 2015	Lower	Not reported	Not reported	Not reported	Not reported	Not reported	Not reported	Not reported	Not reported	Not reported	Not reported	Not reported
Google-40 2020	Urban	Yes - specify	Not reported	Not reported	Not reported	Not reported	Not reported	Yes - specify	Yes - specify	Not reported	Not reported	Not reported
Google-57 2016	Not reported	Yes - specify	Not reported	Woman	Not reported	Not reported	Not reported	Not reported	Not reported	Not reported	Not reported	Not reported
Google-62 2014	Not reported	Yes - specify	Not reported	Not reported	Not reported	Not reported	Not reported	Not reported	Not reported	Not reported	Not reported	Not reported
Held 2016	Not reported	Yes - specify	Not reported	Not reported	Not reported	Not reported	Not reported	Yes - specify	Not reported	Not reported	Not reported	Not reported
IHI-12 2020	Rural	Yes - specify	Yes - specify	Not reported	Not reported	Not reported	Not reported	Not reported	Not reported	Not reported	Not reported	Not reported
IHI-4 2009	Not reported	Yes - specify	Not reported	Not reported	Not reported	Not reported	Not reported	Not reported	Not reported	Not reported	Not reported	Not reported
IHI-6 2015	Urban	Yes - specify	Not reported	Not reported	Not reported	Not reported	Not reported	Not reported	Not reported	Not reported	Not reported	Not reported
King 2008	Not reported	Yes - specify	Yes - specify	Woman	Not reported	Not reported	Not reported	Not reported	Not reported	Not reported	Not reported	Not reported
Killingo 2017	Lower	Yes - specify	Not reported	Not reported	Not reported	Not reported	Not reported	Not reported	Not reported	Not reported	Not reported	Not reported
Koorts 2018	Not reported	Not reported	Not reported	Not reported	Not reported	Not reported	Not reported	Yes - specify	Not reported	Not reported	Not reported	Not reported
L'Engle 2017	Lower	Not reported	Not reported	Not reported	Not reported	Not reported	Not reported	Not reported	Not reported	Not reported	Not reported	Not reported
Mai - 19 .	Urban	Not reported	Not reported	Not reported	Not reported	Not reported	Not reported	Not reported	Not reported	Not reported	Not reported	Not reported
Mendel 2008	Not reported	Yes - specify	Not reported	Not reported	Not reported	Not reported	Not reported	Not reported	Not reported	Not reported	Not reported	Not reported
Moroz 2020	Not reported	Yes - specify	Not reported	Not reported	Not reported	Not reported	Not reported	Not reported	Not reported	Not reported	Not reported	Not reported
NICE-167 2016	Not reported	Yes - specify	Not reported	Not reported	Not reported	Not reported	Not reported	Not reported	Not reported	Not reported	Not reported	Not reported
NICE-221 2016	Not reported	Yes - specify	Not reported	Not reported	Not reported	Not reported	Not reported	Not reported	Not reported	Not reported	Not reported	Not reported
NICE-9 2021	Urban	Not reported	Not reported	Not reported	Not reported	Not reported	Not reported	Not reported	Not reported	Not reported	Not reported	Not reported
NSW-6 2014	Not reported	Yes - specify	Not reported	Woman	Not reported	Not reported	Not reported	Not reported	Not reported	Not reported	Not reported	Not reported
Pinto 2015	Not reported	Yes - specify	Not reported	Not reported	Not reported	Not reported	Not reported	Not reported	Not reported	Not reported	Not reported	Not reported
Rhodes 2020	Not reported	Yes - specify	Yes - specify	Not reported	Not reported	Not reported	Not reported	Yes - specify	Not reported	Not reported	Not reported	Not reported
Soti-Ulberg 2020	Lower	Not reported	Not reported	Not reported	Not reported	Not reported	Not reported	Not reported	Not reported	Not reported	Not reported	Not reported
Sperber 2008	Urban	Not reported	Not reported	Not reported	Not reported	Not reported	Not reported	Not reported	Not reported	Not reported	Not reported	Not reported
Wagner 2007	Not reported	Not reported	Not reported	Not reported	Not reported	Not reported	Not reported	Not reported	Not reported	Not reported	Not reported	Not reported
Warren 2003	Not reported	Not reported	Not reported	Not reported	Not reported	Not reported	Not reported	Not reported	Not reported	Not reported	Not reported	Not reported
WHO-11 2003	Not reported	Not reported	Not reported	Not reported	Not reported	Not reported	Not reported	Not reported	Not reported	Not reported	Not reported	Not reported
WHO-120 2021	Not reported	Yes - specify	Yes - specify	Not reported	Not reported	Not reported	Not reported	Not reported	Not reported	Not reported	Not reported	Not reported
WHO-169 2013	Not reported	Not reported	Not reported	Not reported	Not reported	Not reported	Not reported	Not reported	Not reported	Not reported	Not reported	Not reported
WHO-179 2012	Not reported	Yes - specify	Yes - specify	Not reported	Not reported	Not reported	Not reported	Not reported	Not reported	Not reported	Not reported	Not reported
WHO-30 2014	Not reported	Not reported	Not reported	Not reported	Not reported	Not reported	Not reported	Yes - specify	Not reported	Not reported	Not reported	Not reported
WHO-34 2011	Not reported	Not reported	Not reported	Not reported	Not reported	Not reported	Not reported	Not reported	Not reported	Not reported	Not reported	Not reported
WHO-414 2018	Not reported	Not reported	Not reported	Woman	Not reported	Not reported	Not reported	Not reported	Not reported	Not reported	Not reported	Not reported
WHO-553 2017	Not reported	Yes - specify	Not reported	Not reported	Not reported	Not reported	Not reported	Not reported	Not reported	Not reported	Not reported	Not reported
WHO-7 2015	Not reported	Yes - specify	Not reported	Not reported	Not reported	Not reported	Not reported	Not reported	Not reported	Not reported	Not reported	Not reported

WHO-8 2018	Not reported	Yes - specify	Yes - specify	Not reported	Not reported	Christian and	Not reported	Not reported	Not reported	Not reported	Not reported	Not reported
WHO-9 2020	Not reported	Not reported	Yes - specify	Not reported	Not reported	Not reported	Not reported	Not reported	Not reported	Not reported	Not reported	Not reported
Yamey 2011	Urban	Not reported	Not reported	Not reported	Not reported	Not reported	Not reported	Not reported	Not reported	Not reported	Not reported	Not reported
Zalazar 2021	Not	Not reported	Occupation:	Other:	Sex at birth:	Religion:	Education:	Socioecono		Age: Not	Disability:	Sexual
Woodward 2023	Rural	Not reported	Occupation:	Gender:		Religion:	Education:	Socioecono		Age: Adults	Disability:	Sexual
Puffer 2022	Not	Not reported	Occupation:	Gender: Not	Sex at birth:	Religion:	Education:	Socioecono		Age: Not	Disability:	Sexual
Murdock 2023	Rural	Not reported	Occupation:	Gender: Not	Sex at birth:	Religion:	Education:	Socioecono		Age: Not	Disability:	Sexual
McGrath 2022	Rural	Not reported	Occupation:	Gender:	Sex at birth:	Religion:	Education:	Socioecono		Age: Not	Disability:	Sexual
Estifanos 2023	Not	Not reported	Occupation:	Gender: Not	Sex at birth:	Religion:	Education:	Socioecono		Age: Not	Disability:	Sexual
Escudero 2020	Not	Not reported	Occupation:	Gender: Not	Sex at birth:	Religion:	Education:	Socioecono		Age: Not	Disability:	Sexual
Woodward 2023	Not	Not reported	Occupation:	Gender: Not	Sex at birth:		Education:	Socioecono		Age: Not		Sexual
Sibuyi 2022	Not	Not reported	Occupation:	Gender: Not	Sex at birth:	Religion:	Education:	Socioecono		Age: Not	Disability:	Sexual
Shaw 2021	Not	Not reported	Occupation:	Gender: Not	Sex at birth:	Religion:	Education:	Socioecono		Age: Not	Disability:	Sexual
Sanuade 2023	Not	Not reported	Occupation:	Gender: Not	Sex at birth:	Religion:	Education:	Socioecono		Age: Adults	Disability:	Sexual
Pesut 2022	Rural	Not reported	Occupation:	Gender: Not	Sex at birth:	Religion:	Education:	Socioecono		Age: Youth	Disability:	Sexual
Patil 2023	Not	Not reported	Occupation:	Gender: Not	Sex at birth:	Religion:	Education:	Socioecono		Age: Not	Disability:	Sexual
Parry 2022	Not	Not reported	Occupation:	Gender: Not	Sex at birth:	Religion:	Education:	Socioecono		Age: Not	Disability:	Sexual
Ogbulafor 2023	Rural	Not reported	Occupation:	Gender: Not	Sex at birth:	Religion:	Education:	Socioecono		Age: Not	Disability:	Sexual
Nwaozuru 2022	Not	Yes, if yes	Occupation:	Gender: Not	Sex at birth:	Religion:	Education:			Age: Not	Disability:	Sexual
Nair 2021	Rural	Not reported	Occupation:	Gender:	Sex at birth:	Religion:	Education:	Socioecono		Age: Youth	Disability:	Sexual
Mooses 2021	Not	Not reported	Occupation:	Gender: Not	Sex at birth:	Religion:	Education:	Socioecono		Age:	Disability:	Sexual
McLaughlin 2021	Not	Not reported	Occupation:	Gender: Not	Sex at birth:	Religion:	Education:	Socioecono		Age: Not	Disability:	Sexual
Matindo 2022	Not	Not reported	Occupation:	Gender:	Sex at birth:	Religion:	Education:	Socioecono		Age:	Disability:	Sexual
MacInnes 2023	Not	Not reported	Occupation:	Gender: Not	Sex at birth:	Religion:	Education:	Socioecono		Age: Not	Disability:	Sexual
Lenton 2021	Not	Not reported		Gender: Not	Sex at birth:	Religion:	Education:	Socioecono		Age: Not	Disability:	Sexual
Kumar 2023	Rural	Not reported	Occupation:	Gender: Not	Sex at birth:	Religion:	Education:	Socioecono		Age: Youth	Disability:	Sexual
Kodish 2022	Not	Not reported	Occupation:	Gender: Not	Sex at birth:	Religion:	Education:	Socioecono		Age: Not	Disability:	Sexual
Kiracho 2021	Not	Not reported	Occupation:	Gender: Not	Sex at birth:	Religion:	Education:	Socioecono		Age: Not	Disability:	Sexual
Jwanle 2023	Not	Not reported	Occupation:	Gender: Not	Sex at birth:	Religion:	Education:	Socioecono		Age: Not	Disability:	Sexual
Jayanna 2023	Not	Not reported	Occupation:	Gender: Not	Sex at birth:	Religion:	Education:	Socioecono		Age: Not	Disability:	Sexual
Gaber 2022	Not	Not reported	Occupation:	Gender: Not	Sex at birth:	Religion:	Education:	Socioecono		Age: Not	Disability:	Sexual
Flax 2023	Not	Not reported	Occupation:	Gender: Not	Sex at birth:	Religion:	Education:	Socioecono		Age: Not	Disability:	Sexual
Fiori 2023	Not	Not reported	Occupation:	Gender: Not	Sex at birth:	Religion:	Education:	Socioecono		Age: Not	Disability:	Sexual
ElJoueidi 2021	Not	Not reported	Occupation:	Gender: Not	Sex at birth:	Religion:	Education:	Socioecono		Age: Not	Disability:	Sexual
Dickson 2023	Not	Yes, if yes	Occupation:	Gender:	Sex at birth:	Religion:	Education:	Socioecono		Age: Adults	Disability:	Sexual
Dev 2021	Not	Not reported	Occupation:	Gender:	Sex at birth:	Religion:	Education:	Socioecono		Age: Youth	Disability:	Sexual
Corches 2020	Not	Not reported	Occupation:	Gender: Not	Sex at birth:	Religion:	Education:	Socioecono		Age: Adults	Disability:	Sexual
Chowdhary 2022	Not	Not reported	Occupation:	Gender:	Sex at birth:	Religion:	Education:	Socioecono		Age: Not	Disability:	Sexual
Chau 2021	Not	Not reported	Occupation:	Gender:	Sex at birth:	Religion:	Education:	Socioecono		Age: Adults	Disability:	Sexual
Chamie 2022	Rural	; Yes.	Occupation:	Gender: Not	Sex at birth:	Religion:	Education:	Socioecono		Age: Adults	Disability:	Sexual
Bharmal 2022	Not	Not reported	Occupation:		Sex at birth:	Religion:	Education:	Socioecono		Age: Youth	Disability:	Sexual
Berbakov 2023	Not	Not reported	Occupation:	Gender: Not	Sex at birth:	Religion:	Education:	Socioecono		Age: Seniors	Disability:	Sexual
Barker 2023	Not	Not reported	Occupation:	Gender: Not	Sex at birth:	Religion:	Education:	Socioecono		Age: Not	Disability:	Sexual
Balayah 2021	Not	Not reported	Occupation:	Gender: Not	Sex at birth:	Religion:	Education:	Socioecono		Age: Not	Disability:	Sexual
Azevedo 2022	Not	Not reported	Occupation:	Gender: Not	Sex at birth:	Religion:	Education:	Socioecono		Age: Youth	Disability:	Sexual
AsamoahAmpofo 2022	Not	Not reported	Occupation:	Gender:	Sex at birth:	Religion:	Education:	Socioecono		Age: Youth	Disability:	Sexual
Akter 2023	Not	Not reported	Occupation:	Gender: Not	Sex at birth:	Religion:	Education:	Socioecono		Age: Not	Disability:	Sexual
Akinyemi 2022	Not	Not reported	Occupation:	Gender:	Sex at birth:	Religion:	Education:	Socioecono		Age: Not	Disability:	Sexual
